# Protecting‐Group‐Free Amidation of Amino Acids using Lewis Acid Catalysts

**DOI:** 10.1002/chem.201800372

**Published:** 2018-04-30

**Authors:** Marco T. Sabatini, Valerija Karaluka, Rachel M. Lanigan, Lee T. Boulton, Matthew Badland, Tom D. Sheppard

**Affiliations:** ^1^ Department of Chemistry University College London, Christopher Ingold Laboratories 20 Gordon Street London WC1H 0AJ UK; ^2^ GlaxoSmithKline Medicines Research Centre GunnelsWood Road Stevenage, Herts SG12NY UK; ^3^ Pfizer Global Pharmaceutical Sciences, Discovery Park Ramsgate Road Sandwich UK

**Keywords:** amides, amino acids, boron, catalysis, green chemistry

## Abstract

Amidation of unprotected amino acids has been investigated using a variety of ‘classical“ coupling reagents, stoichiometric or catalytic group(IV) metal salts, and boron Lewis acids. The scope of the reaction was explored through the attempted synthesis of amides derived from twenty natural, and several unnatural, amino acids, as well as a wide selection of primary and secondary amines. The study also examines the synthesis of medicinally relevant compounds, and the scalability of this direct amidation approach. Finally, we provide insight into the chemoselectivity observed in these reactions.

## Introduction

**Scheme 1:**
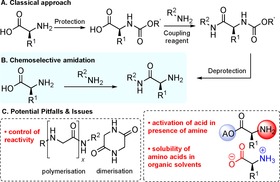
Chemoselective amidation of unprotected amino acids: A) Classical approach to the synthesis of amino amides B) Chemoselective amidation of an unprotected amino acid. C) Potential pitfalls & Issues.

Chemical reactions for the formation of amide bonds are among the most commonly employed transformations in organic chemistry; amides featuring in 25 % of industrially important pharmaceuticals as well as a wide selection of bioactive natural products and polymeric materials.^1^ The synthesis of amides derived from amino acids constitutes a major application of amide bond formation chemistry. While the synthesis of amino amides by the assembly of N‐protected α‐amino acids and amines is a well‐known and accepted methodology,^2^ the synthesis of amides directly derived from unprotected amino acids (Scheme 1 B) is rare, despite shortening the synthetic sequence by two steps. This class of amidation reaction presents issues relating to control of reactivity, but the lack of research effort in this area is probably due to the preconception that such a reaction simply would not work (Scheme 1 C). Nevertheless, a small number of methods for direct amidation of unprotected amino acids have been developed.

**Scheme 2:**
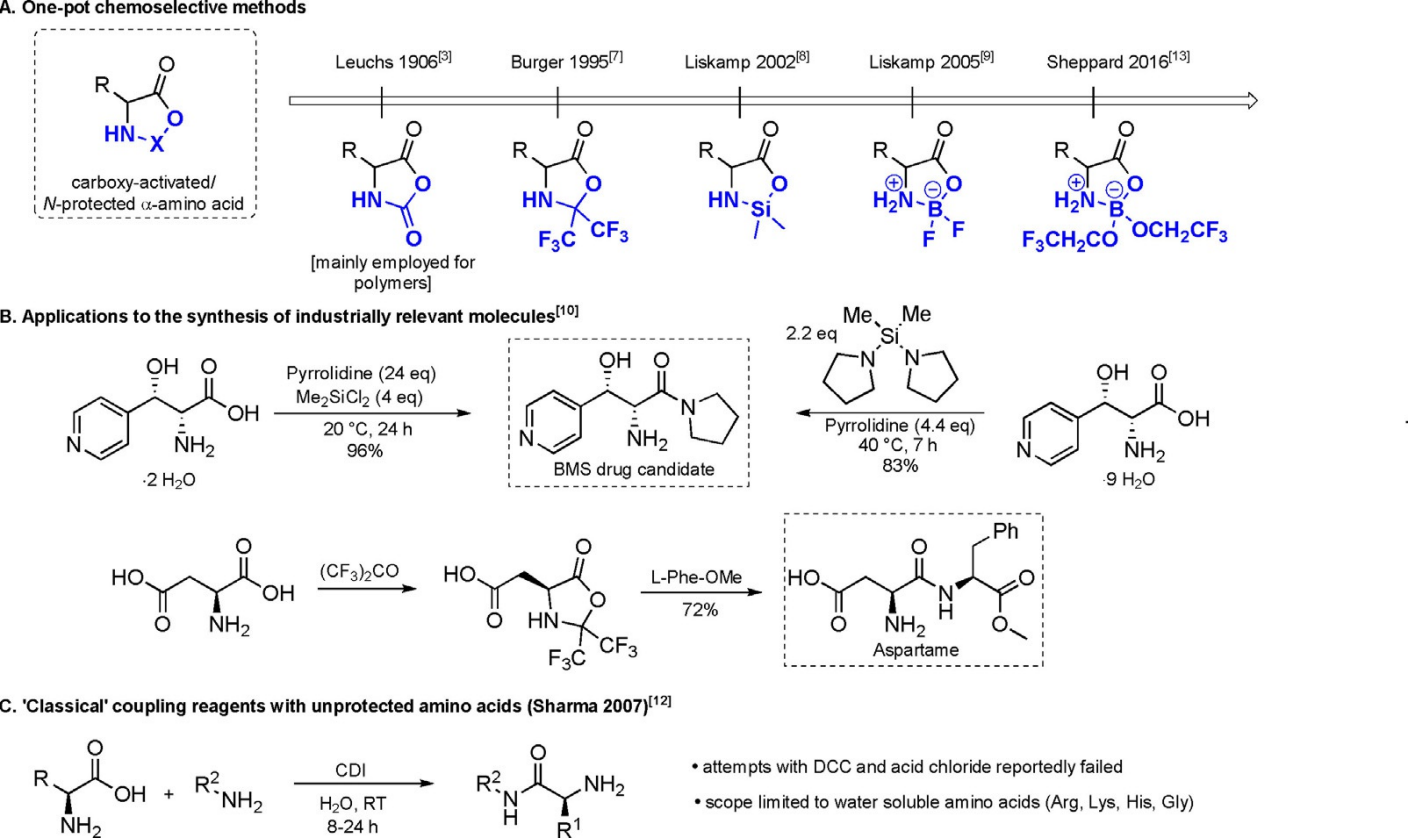
Chemoselective amidation of unprotected amino acids: A) One‐pot chemoselective methods.^3^, ^4^, ^5^, ^6^, ^7^, ^8^, ^9^, ^13^ B) Applications to the synthesis of drug/natural products.^6^, ^10^ C) “Classical” coupling reagents with unprotected amino acids.^12^

The first known method for the chemoselective amidation of unprotected amino acids was developed by Leuchs, and employed phosgene or derivatives thereof to generate reactive *N*‐carboxyanhydrides (NCAs) for subsequent reaction with amines (Scheme 2).^3^ However the activation occurs to such an extent that it leads to polymerization^4^ and the amino acid still requires protection for a fully selective monoacylation.^5^

Since then, there have been several reports of methods for the synthesis of amides derived from amino acids employing a transient activating group for the carboxylic acid that simultaneously protects the amine (Scheme 2 A). Burger et al. reported the use of gaseous hexafluoroacetone (HFA) for the formation of activated oxazolidinones,^6^, ^7^ which are reactive towards amines. The applicability of this method was demonstrated through the shortest synthesis of aspartame to date (Scheme 2 B).^6^ Later, similar approaches were exploited by Liskamp et al. using dichloroalkyl silanes^8^ or boron trifluoride^9^ to produce activated Lewis acid conjugates in which the amino acid was proposed to coordinate in a bidentate fashion. Remarkably, Bristol–Meyers–Squibb have employed the silane‐based methodology for the synthesis of an amino amide drug candidate,^10^ demonstrating benefits with regard to both cost‐effectiveness and atom economy (Scheme 2 B).^11^ More recently, Sharma et al.^12^ reported the amidation of some unprotected amino acids employing a “classical” coupling reagent, 1′‐carbonyldiimidazole (CDI), in water. Nevertheless, the method displayed serious drawbacks with regard to scope, only succeeding in the amidation of four amino acids (Scheme 2 C).

We have recently reported that the borate ester B(OCH_2_CF_3_)_3_ is an effective reagent for the direct synthesis of α‐amino amides from unprotected amino acids and amines.^13^, ^14^ In this study, we outline the full scope of the direct amidation of unprotected amino acids employing both stoichiometric and catalytic quantities of a variety of boron Lewis acids as well as group(IV) metal salts and “classical” coupling reagents.

## Results and Discussion

### Classical coupling reagents

The acylation of amines with activated carboxylic acids is the most common way to make an amide, as a consequence of the widespread availability and high stability of both of these building blocks. In fact, based on literature surveys, the most commonly used methods for amidation involve the formation of intermediary acid chlorides, *O*‐acyl ureas or (mixed) anhydrides.^15^ We therefore began our investigation by looking at the efficiency of classical coupling reagents for the chemoselective amidation of unprotected phenylalanine with benzylamine. Using polar solvents with the potential to partially solubilize unprotected amino acids (H_2_O, EtOH), biphasic systems (CH_2_Cl_2_/H_2_O) as well as non‐protic solvents (DMF, CH_2_Cl_2_) we looked at the competency of ‘classical“ coupling reagents in amidation. In most cases, little or no amino amide was produced (Table 1, entries 1–6). In the case of amidation employing CDI in water (entry 7), the formation of the desired amino amide was observed in small quantities, accompanied by the formation of three other amide species, highlighting issues relating to control of reactivity. Overall, we believe that the lack of reactivity, as suggested previously,^12^ is mainly due to the poor solubility of zwitterionic α‐amino acids both in organic solvents and in aqueous solution.

**Table 1:** “Classical” coupling reagents in amidation of amino acids.

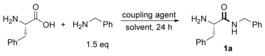			
Entry	Coupling agent	Solvents	Yield [%]^[a]^
1	EDC/HOBt	CH_2_Cl_2_/H_2_O	<5
2	EDC**⋅**HCl/HOBt	NMM, H_2_O	<5
3	EDC**⋅**HCl/HOBt	NMM, EtOH	<5
4	T3P	DMF	0
5	HATU	DMF	0
6	SOCl_2_	Et_3_N/CH_2_Cl_2_	0
7	CDI	H_2_O	<10

[a] 1,4‐dimethoxybenzene was used as an internal standard. Complete reaction conditions and spectra of crude reaction mixtures as can be found in the Supporting Information. EDC⋅HCl=*N*‐(3‐dimethylaminopropyl)‐*N*′‐ethylcarbodiimide hydrochloride; HATU=(2‐(1*H*‐benzotriazol‐1‐yl)‐1,1,3,3‐tetramethyluronium‐hexafluorophosphate); HOBt=1‐hydroxybenzotriazole; NMM=*N*‐methylmorpholine.

### Stoichiometric amidation with Lewis acids

We have recently reported the use of B(OCH_2_CF_3_)_3_ as an effective reagent for the direct synthesis of α‐amino amides from unprotected amino acids and excess amine. In most cases, the pure amino amide could be obtained using a solid phase workup to remove amino acid and boron compounds, followed by evaporation of the volatile components.13b All 20 common proteogenic amino acids (Table 2, entries 1–20) as well as six unnatural amino acids (entries 21–26) were evaluated in the reaction. Although polar amino acids (namely arginine (**2 b**), asparagine (**2 c**) and glutamine (**2 f**)) did not efficiently undergo amidation, the method was extremely successful with the less polar amino acids (**2 a**, **2 g**–**2 h**, **2 j**–**2 k, 2 m**–**2 o, 2 q**–**2 t**). Overall, 60 % of the natural amino acids and 5/6 of the unnatural amino acids (**2 u**–**2 z**) underwent amidation to give amides in good yields and with high enantiomeric ratio (e.r.) (>60 % yield and >90:10 e.r.).

**Table 2:** B(OCH_2_CF_3_)_3_‐mediated amidation of free amino acids with propylamine.13b Sarcosine (Sar), α‐aminoisobutyric acid (AiB); Homoserine (Hse); l‐2‐Aminobutyric acid (AABA). e.r. could not be determined for **2 c**, **2 e**, **2 f**, **2 i** or **2 l**.

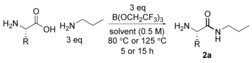
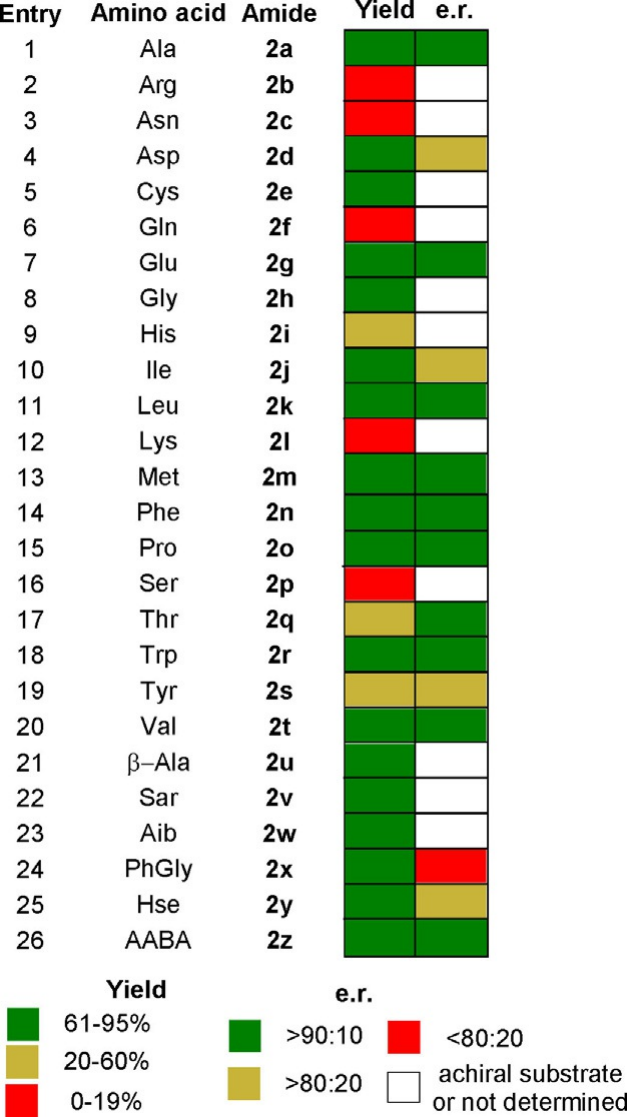

Following this study, we went on to look at the reaction conditions in more detail in order to minimize the levels of racemization seen for some examples. The reaction of tryptophan with propylamine was examined with various conditions, and it was found that racemization could be minimized by shortening the reaction time and/or by adding the borate reagent dropwise (Table 3).

**Table 3:** Reducing racemization through the dropwise addition of B(OCH_2_CF_3_)_3_ and by decreasing the reaction time.

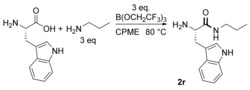				
Entry	Reaction time [h]	Borate added dropwise	Yield [%]	e.r.
1	15	N	93	80:20
2	5	N	87	83:17
3	15	Y	93	84:16
4	5	Y	83	91:9

**Figure 1:**
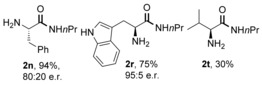
Ti(O*i*Pr)_4_‐mediated amidation of free amino acids with propylamine.

We have also attempted the reaction to synthesise **1 a** under similar conditions with a variety of group (IV) metal salts (Table 4), as these types of species have been reported to be active catalysts for amide bond formation in recent years.^16^, ^17^ While reactions employing zirconium‐based reagents solidified and produced only minor quantities of amide **1 a**, Ti(O*i*Pr)_4_ was identified as a suitable alternative amidation reagent. The reactions were also attempted in the presence of molecular sieves but the resulting yields were slightly lower. A selection of amino amides were synthesised using 1 equiv of Ti(O*i*Pr)_4_ (Figure 1). Amide **2 n** was synthesised in high yield but with significant racemisation. Although lower yields were seen for the synthesis of **2 r** and **2 t**, the use of Ti(O*i*Pr)_4_ furnished products with higher enantiopurity (≥95:5 e.r.) than the reactions mediated by B(OCH_2_CF_3_)_3_. Hence, Ti(O*i*Pr)_4_ represents an easily accessible and lower cost alternative to our borate ester, especially for more reactive amino acids.

**Table 4:** Ti/Zr in amidation of amino acids.

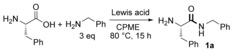			
Entry	Lewis acid	Equiv	Yield [%]
1	B(OCH_2_CF_3_)_3_	3	85
2	[Cp_2_ZrCl_2_]	1	0
3	ZrCl_4_	1	32
4	Ti(O*i*Pr)_4_	1	85
5	Ti(O*i*Pr)_4_^[a]^	1	75

[a] Reaction run with mol. sieves. Cp=1,5‐cyclopentadiene.

### Catalytic amidation employing Lewis acids

**Scheme 3:**
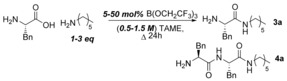
DoE study on the direct amidation reaction to form **3 a**. For further details, please see the Supporting Information.

Having recently developed a method for general amidation employing catalytic B(OCH_2_CF_3_)_3_,^14^ we wished to explore the applicability of this approach in the amidation of unprotected amino acids. In the optimisation of the general catalytic amidation reaction, we screened a wide selection of organic solvents under Dean–Stark conditions and identified *tert*‐amyl methyl ether (TAME) as a suitable alternative to CPME and PhMe, which crucially allows for azeotropic removal of water to be conducted at lower temperatures (86 °C).^14^ Design of Experiments (DoE) reaction optimisation^18^ was then conducted to understand the factors playing a role in the amidation of an unprotected amino acid, and to maximise the yield of product **3 a** and minimise the formation of diamide byproduct **4 a**, the product of amidation of the amino acid with the desired amino amide product. The catalyst loading, amine loading and volume of solvent used were investigated as factors (Scheme 3).

Good quality models for predicting the yield of both products were obtained from the DoE study. The results indicated that only the amine loading and catalyst loading had a significant effect on the yield of the desired amino amide. Unsurprisingly, we found that excess amine was beneficial for minimising byproduct formation. Increasing the catalyst loading led to increased formation of both products. As a result of this study, we were able to identify conditions to obtain high yields of the desired amino amide by running the reactions with only 1.5 equivalents of amine and 20 mol % B(OCH_2_CF_3_)_3_ catalyst. Lowering the amounts of amine further led to increased formation of diamino amide **4 a**, although less than statistically expected (usually ranging between 1–8 %, separable during purification).

With effective conditions in hand for the use of catalytic B(OCH_2_CF_3_)_3_ for the direct amidation of an unprotected amino acid, we then went on to explore the use of alternative Lewis acid catalysts under these conditions. The use of 2‐Cl and 3,4,5‐trifluorophenylboronic acids (Table 5, entries 2–3, 9) has been previously reported to be effective for amidation under dehydrative conditions.^19^, ^20^ Such catalysts are generally unsuccessful even for amidation of protected amino acids, and, to the best of our knowledge, they have never been explored with unprotected amino acids.^19^, ^20^, ^21^ However, to our surprise, these boronic acid catalysts were reasonably effective for the amidation of Phe with benzylamine under Dean–Stark conditions. Group (IV) metals (entries 4–5) were, as with the stoichiometric conditions, suitable catalysts for this transformation, with Ti(O*i*Pr)_4_ a particularly cost‐effective alternative to our borate ester providing amide **1 a** in excellent yield. When employing ZrCl_4_, isolation of the product was problematic (product lost in workup), most likely due to the formation of complexes between amino amides and Zr and the formation of hydrochloride salts of the amines present in the reaction mixture.

**Table 5:** Lewis acids in the catalytic amidation of unprotected amino acids.^[a]^

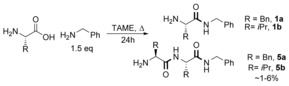			
Entry	R	Catalyst	Yield **4 a** [%]^[b]^
1	Bn	B(OCH_2_CF_3_)_3_	90
2	Bn	*o‐*ClC_6_H_4_B(OH)_2_	63
3	Bn	3,4,5‐F_3_C_6_H_2_B(OH)_2_	71
4	Bn	Ti(O*i*Pr)_4_	85
5	Bn	Zr(Cl)_4_	41
6	*i*Pr	B(OCH_2_CF_3_)_3_	40
7	*i*Pr	Zr(Cl)_4_	43
8	*i*Pr	B(OMe)_3_	8
9	*i*Pr	*o‐*ClC_6_H_4_B(OH)_2_	7
10	*i*Pr	Ti(O*i*Pr)_4_	13
11	*i*Pr	Hf(Cp_2_)(Cl)_2_	25

[a] Amino acid (5 mmol), benzylamine (7.5 mmol), 20 mol % catalyst, TAME (5 mL), Dean–Stark. [b] 1,4‐dimethoxybenzene was used as an internal standard.

The high levels of conversion to **1 a** in these reactions prevents accurate differentiation of the reactivity of the various catalysts, so we went on to screen selected catalysts for the direct amidation of valine, a much less reactive substrate. Again, the amidation could be achieved chemoselectively with both group(IV) catalysts and boron‐based catalysts. Although amidation employing ZrCl_4_ gave the highest level of conversion, attempts at isolation failed due to issues relating to adduct/salt formation (vide supra, for use of stoichiometric ZrCl_4_). B(OCH_2_CF_3_)_3_ was therefore identified as the catalyst of choice, but Ti(O*i*Pr)_4_ is a suitable alternative for more reactive amino acids.

### Substrate scope of borate‐catalysed amidation

**Scheme 4:**
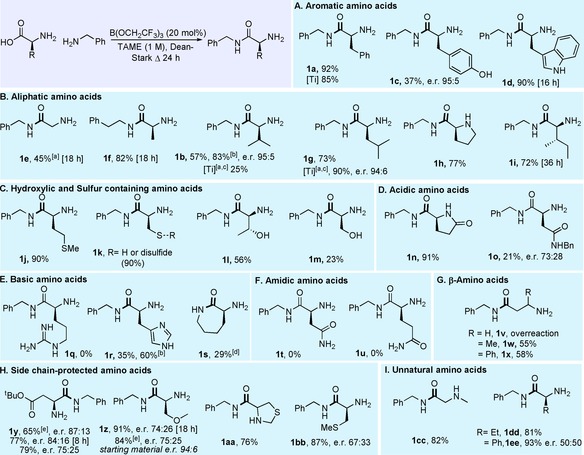
Scope of amidation reaction with unprotected amino acids. A) Aromatic amino acids B) Aliphatic amino acids. C) Hydroxylic and sulphur‐containing amino acids D) Acidic amino acids. E) Basic amino acids. F) Amidic amino acids. G) β‐amino acids. H) Side‐chain‐protected amino acids. I) Unnatural amino acids. enantiomeric/diastereomeric ratios >95:5, unless otherwise stated. e.r. for **1 k**, **1 m** and **1 r** could not be determined. Yields in parentheses are calculated against an internal standard (see ESI). [a] 3 equiv amine used. [b] 30 mol % B(OCH_2_CF_3_)_3_. [c] Reaction conducted with Ti(O*i*Pr)_4_ (15 mol %) in CPME. [d] No benzylamine used. [e] 10 mol % B(OCH_2_CF_3_)_3_.

With B(OCH_2_CF_3_)_3_ as the catalyst, we explored the scope of the amino acid component with benzylamine as the amine (Scheme 4). All 20 common proteinogenic, as well as nine non‐proteinogenic amino acids were tested. In general, the less polar amino acids were good substrates for the reaction, with two aromatic (**1 a**, **1 d**) and most aliphatic (**1 f**–**i**) amino acids yielding the corresponding amide in good to excellent yields (Scheme 4 A, B). The amide derived from alanine and benzylamine could not be readily separated from unreacted benzylamine during purification, so a reaction was, therefore, performed with phenethylamine to give amide **1 f** in excellent yield. The reaction with glycine led to the formation of a mixture of the desired amide **1 e** and its diamido counterpart **5 e** under the standard conditions, so a larger excess of amine had to be employed (3 equiv) and only a moderate yield of **1 e** was obtained. We were also able to use catalytic quantities of Ti(O*i*Pr)_4_ for the preparation of amino amides derived from more reactive amino acids (e.g. **1 a**, **1 g**). This catalyst was much less effective for more hindered amino acids, however (e.g. **1 b**). Pleasingly, by using B(OCH_2_CF_3_)_3_ we were also able to access amides of more polar amino acids with hydroxyl (**1 l**–**m**) and sulfur (**1 j**,**1 k**) moieties present, and good conversions were observed (Scheme 4 C). Amide **1 k** (from cysteine) was partially oxidized to the corresponding disulfide over the course of the reaction and upon exposure to air.

Acidic amino acids underwent amidation effectively (Scheme 4 D), with glutamic acid cyclizing intramolecularly to give pyroglutamide **1 n** and aspartic acid undergoing double amidation to give a diamide (**1 o**). The increased degree of racemization for **1 o** is probably due to a competing dehydrative mechanism involving a 5‐membered acid anhydride, known to have a propensity for racemization.^24^ The basic (**1 q**–**s**) and amidic (**1 t**–**u**) amino acids were somewhat less reactive (Scheme 4 E, F), with only histidine giving an amide derived from benzylamine (**1 r**). As expected, lysine spontaneously cyclized to form α‐aminocaprolactam **1 s**. β‐Amino acids generally worked less well than their α‐amino counterparts (Scheme 4 G). As with glycine, β‐alanine underwent extensive over‐reaction (**1 v**), and even with three equivalents of amine did not give a clean amino amide product. However, β‐amino acids could be coupled successfully to give amides **1 w**–**x**, albeit in lower yields relative to their α‐amino acid counterparts.

Amino acids with protected side chains were also tested (Scheme 4 H). Ester‐protected glutamic acid and the methyl‐ether derivative of serine gave amides **1 y** and **1 z**, respectively, in good yields, despite showing signs of significant racemization. Protected cysteine formed desired amide **1 aa** along with minor amounts of a rearranged product (see ESI); *S*‐methyl cysteine yielded amide **1 bb** in excellent yield, but with significant racemization. Sarcosine, l‐α‐aminobutyric acid and phenylglycine all underwent successful amidation (Scheme 4 I), although the latter (**1 ee**) underwent complete racemization, as expected based on its known propensity to racemise even under mild conditions (e.g. Cbz‐PhGly+NH_**4**_Cl yields amide in 43 % yield, 46 % *ee* using ethyl chloroformate, 5° C, 1 h).^25^ Overall, the method showed similar trends in reactivity to the stoichiometric approach (vide supra, Table 2). The relevance of this chemistry can easily be exemplified by the fact that most of the amides synthesized in Scheme 4 (or their enantiomers) are members of a well‐documented class of potent anticonvulsants and agents for neuropathic pain treatment, commonly referred to as primary amino acid derivatives (PAADs).^22^, ^23^

**Scheme 5:**
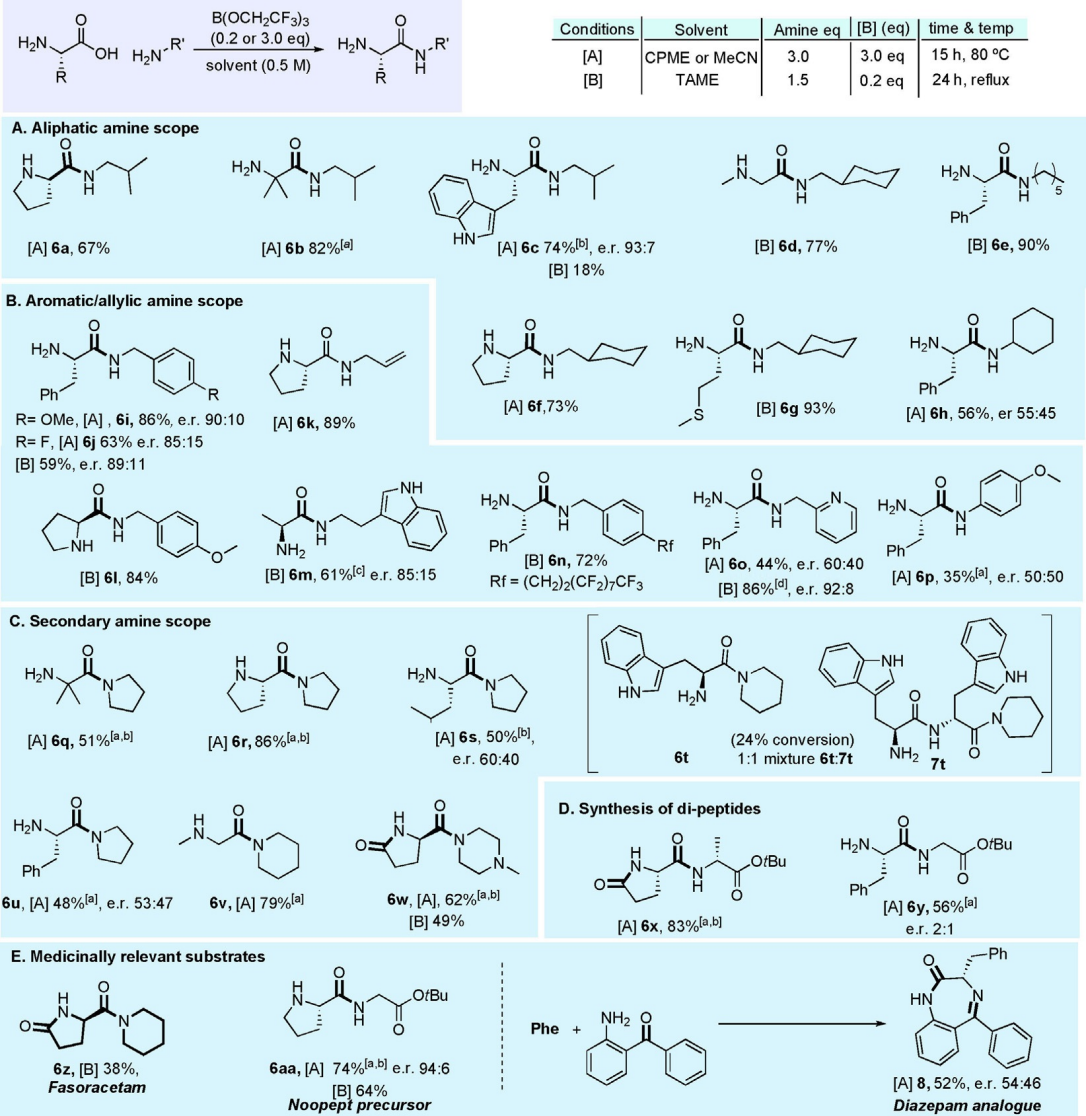
Scope of amine component for amidation of unprotected amino acids. A) Aliphatic amine scope. B) Aromatic/allylic amine scope. C) Secondary amine scope. D) Synthesis of dipeptides. E) Medicinally relevant substrates. Enantiomeric/diastereomeric ratios >95:5, unless otherwise stated. [a] Reaction run at 125 °C. [b] B(OCH_2_CF_3_)_3_ added dropwise. [c] 2 equiv of amine used. [d] 3 equiv of amine used. [e] 30 mol % B(OCH_2_CF_3_)_3_.

The scope of the reaction with regard to the amine component was investigated next through the preparation of amides derived from a selection of different amines employing both stoichiometric B(OCH_2_CF_3_)_3_ (denoted as [A]) and the catalytic reaction conditions (denoted as [B], Scheme 5).

Simple aliphatic amines worked well under both sets of conditions (**6 a**–**g**, Scheme 5 A). However, the reaction of isobutylamine with tryptophan under catalytic conditions resulted in a low yield of amide most likely due to the volatility of the amine, which is probably removed into the side arm of the Dean–Stark apparatus. Benzylic and allylic amines were also successful (Scheme 5 B), with substituted benzylamines bearing a methoxy group (**6 i**, **6 l**), fluoride (**6 j**) or polyfluoroalkane (**6 n**) giving good yields of the corresponding α‐amino amides under both sets of conditions. Heterocyclic tryptamine (**6 m**) and 2‐picolylamine (**6 o**) also underwent amidation successfully, with significant amounts of racemization in the latter case when stoichiometric B(OCH_2_CF_3_)_3_ was used. Pleasingly, the degree of racemization was significantly reduced under catalytic conditions.

Amino amides could also be prepared from reactive secondary amines in good yield (**6 q**–**s**, **6 u**–**w**, Scheme 5 C), but only with the stoichiometric method. The reactions with leucine and phenylalanine with pyrrolidine required forcing reaction conditions which led to significant levels of racemization (**6 s**, **6 u**). When employing catalytic B(OCH_2_CF_3_)_3_ for the synthesis of a tertiary amide, even with a large excess of amine (3 equiv) the reaction was not selective and led to further reaction of **6 t** to give diamide **7 t**. Under the catalytic set of conditions, glutamic acid was the only amino acid to show selectivity for monoamidation with a secondary amine, due to intramolecular cyclisation. Indeed, it was possible to synthesise **6 w** and the pharmaceutical Fasoracetam **6 z** (Scheme 5 E) in one step from glutamic acid.

It was also possible to prepare dipeptide derivatives using glycine and alanine *tert*‐butyl esters as the nucleophiles (Scheme 5 D, E). Reaction of both Phe (**6 y**) and Pro (**6 aa**) was attempted with O*t*Bu Gly, although only the latter gave a good yield and enantiopurity, with phenylalanine undergoing significant racemization (**6 y**). Conveniently, **6 aa** is a precursor to a marketed nootropic, Noopept, which can be accessed through a further condensation employing our catalytic amidation conditions.^14^ Glutamic acid also underwent successful cyclisation/amidation with Ala‐O*t*Bu to give **6 x** in good yield. We were also able to synthesize a benzodiazepine derivative **8**, which belongs to a class of anti‐anxiolytic drugs, from 2‐aminobenzophenone and l‐Phe in 52 % yield, albeit under forcing conditions, which again led to a near full racemisation of the final product.

**Scheme 6:**
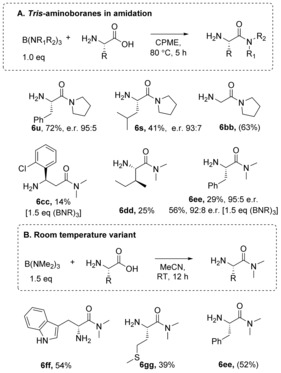
Tris(amino)boranes in amidation. Enantiomeric/diastereomeric ratios >95:5, unless otherwise stated.

As the synthesis of tertiary amides was problematic in most cases, we set out to explore an alternative protocol using aminoboranes (Scheme 6), which have been previously demonstrated to promote amidation of carboxylic acids^26^, ^27^ and esters.^28^ Commercially available tris(amino) boranes were found to be effective for direct amidation of unprotected amino acids, affording amides in moderate yield and enantiopurity. Due to the volatility of amines, such as dimethylamine, these amides would be difficult to prepare using the borate‐mediated reactions outlined above. Using equimolar amounts of aminoborane under conditions analogous to the stoichiometric borate reaction, amino amides **6 u**, **6 s** and **6 bb**–**gg** were synthesized in low to good yields and with little sign of racemization. In acetonitrile, the remarkable reactivity of these aminoboranes enabled the synthesis of amides **6 ff**–**gg** and **6 ee** under very mild conditions at room temperature. This method is, however, limited by the requirement to employ an excess of amine in the synthesis of the tris(amino) borane, something which is only likely to be economically viable with low‐cost readily available amines.

### Sequential amidation reactions

With a method to provide direct access to amino amide derivatives, we reasoned that we could use the free amine group in further transformations to access useful compounds. We envisaged that the direct synthesis of free amino amides could be combined with our previously reported amidation processes^13^ to provide access to relatively complex α‐amido amides in a simple operation.

We started by exploring sequential amidation reactions with our stoichiometric reaction conditions. To this end, direct amidation of a free amino acid with propylamine was followed by a filtration workup to remove unreacted amino acid and boron residues and give the crude α‐amino amide. This was then subjected to direct borate‐mediated amidation with a carboxylic acid to give an α‐amido amide, which was purified by a second filtration workup (Table 6). The diamides **10 a**–**e** were obtained efficiently over the two‐step sequence in all cases, with no requirement for chromatographic purification.

**Table 6:** Sequential double amidation reactions.

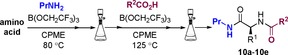		
Amide	Step 1 yield [%]	Step 2 yield [%] (e.r.)
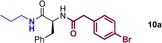	95	57 (87:13)
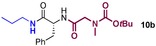	94	64 (98:2)
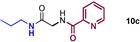	71	57
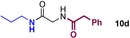	71	77
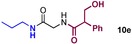	71	63

We then went on to explore an analogous transformation employing the catalytic conditions in a one‐pot procedure (Table 7). Following the standard protocol for direct amidation of a free amino acid, a solution of carboxylic acid in TAME was added to enable a second amidation reaction to take place. In general these double amidation reactions gave high conversions, although in the case of chiral substrates, significant epimerization took place most likely due to the extended reaction times. Purification for diamides **11** employing column chromatography was particularly difficult, due to the formation of amide side products from the reaction of the reactant amine with the second carboxylic acid coupling partner. Nonetheless, the one‐pot synthesis of Lacosamide was successful with this method, yielding desired amide **11 d**, albeit with a low enantiopurity. It is worth noting, however, that this substrate is particularly prone to racemization,^29^, ^30^ and that the first step of the amidation reaction (**1 z**, Scheme 4) also showed erosion of enantiopurity (vide supra).

**Table 7:** Sequential catalytic double amidation reactions (one‐pot).

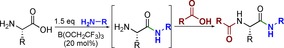	
Amide	Yield [%]
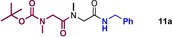	80
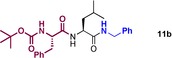	61 (85:15 d.r.)
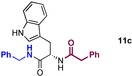	69 (98:2 e.r.) (recryst)
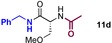	73 (66:34 e.r.) (SM e.r. 94:6)

### Sequential condensation reactions

**Scheme 7:**
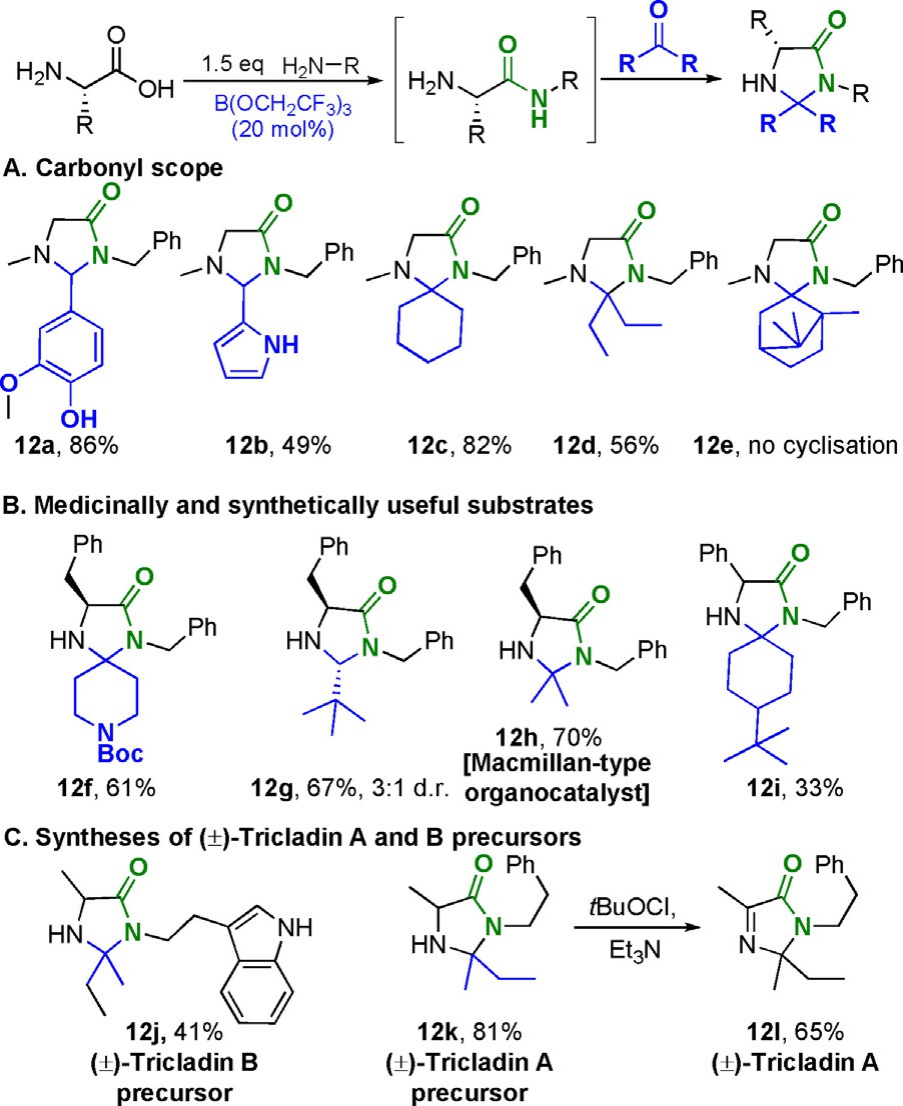
Sequential condensation reactions for the synthesis of imidazolidinones. Enantiomeric ratios >95:5, unless otherwise stated. The e.r. of **12 f** could not be determined.

Given that B(OCH_2_CF_3_)_3_ has previously been shown to promote imine formation when used stoichiometrically,^31^ we also explored a one‐pot unprotected amino acid amidation/condensation reaction to provide access to imidazolidinones in a single step (Scheme 7). In this case the cyclisation reaction was much quicker (1–2 h), and yielded products with very little or no signs of racemization. Imidazolidinones derived from a selection of amino acids (Phe, Ala and Sar) were cyclized with aliphatic (**12 c**,**d**, **12 g–i**, **12 k**), benzylic (**12 a**), heterocyclic (**12 b**, **12 j**) and hydroxyl containing (**12 a**) aldehydes or ketones in good to excellent yields. Only the reaction of camphor (**12 e**) failed to yield the desired heterocycle. We were also able to synthesise precursors to two natural products (±)‐Tricladin A and B from alanine in one step.^32^

### Scalability and green metrics

**Figure 2:**
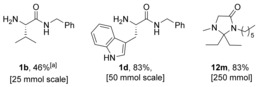
Scale‐up amidations and sequential condensations. See the Supporting Information for detailed procedures. [a] 40 mol % B(OCH_2_CF_3_)_3_.

As our goal was to develop a highly efficient and scalable amidation protocol, we sought to test the synthesis of a set of substrates on a larger scale. Both the chemoselective amidation protocol (25–50 mmol) and the sequential amidation/condensation procedures (250 mmol) were amenable to scale up to access multigram quantities of material, although in slightly lower yields than the smaller scale reactions in the case of **1 b** and **1 d** (Figure 2). This is likely due to the heterogeneity of the system, which makes adequate mixing more difficult on a larger scale.

**Scheme 8:**
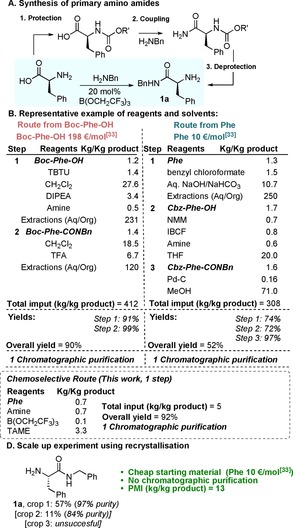
Efficiency and cost‐effectiveness of chemoselective amidation in comparison to regular amide bond‐formation processes.

Next we set out to demonstrate the efficiency and cost‐effectiveness of our method by benchmarking it against “classical” amide coupling approaches (Scheme 8). We examined the synthesis of amide **1 a** by using our method, and using literature approaches starting from either the free amino acid or the *tert*‐butyloxycarbonyl (Boc)‐protected derivative, which is also commercially available and commonly used as a starting material. However, despite Boc‐Phe‐OH being available at what is often considered a nominal cost, it is >20 times more expensive than the free amino acid (198 vs. 10 € mol^−1^)!^33^

It is clear on the basis of the total material input required in terms of reagents and solvents, that the direct chemoselecitve amidation route offers significant benefits. The literature route from the free amino acid requires a total material input of 308 Kg per Kg of amide product, and the approach from the Boc‐protected amino acid requires an even larger material input (412 Kg per Kg of amide product). In comparison, our chemoselective amidation method requires a material input of only 5 Kg per Kg of amide product obtained, ≈60–80 times more efficient. As these methods for the syntheses of amino amides employ column chromatographic purification, we are unable to calculate the process mass intensities (PMIs).^34^ However, the synthesis of amide **1 a** was carried out on a 25 mmol scale and crystallization was used to obtain the product with high purity, though in a lower yield (57 %) than from chromatographic purification. Nevertheless, this process proceeds with an impressive PMI of only 13, which compares very favourably to established amidation methods (Typical PMI values in the range of 150–300).^34^

### Origins of chemoselectivity

**Scheme 9:**
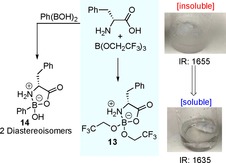
Interaction of boron Lewis acids with amino acids.

**Figure 3:**
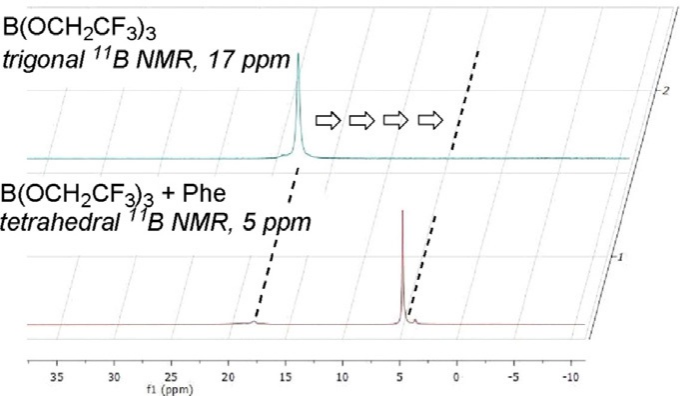
^11^B NMR of the interaction of B(OCH_2_CF_3_)_3_ with phenylalanine.

The interactions between amino acids and boron Lewis acids were investigated by NMR studies. The NMR spectra suggest that the Lewis acid coordinates to the amino acid to form a cyclic intermediate such as **13** or **14** (Scheme 9). A shift in the ^11^B NMR spectrum from the trigonal to the tetrahedral region suggests the formation of a structure such as **13** (Figure 3). The interaction between the borate species and the amino acid effectively solubilizes the amino acid in organic solvent, allowing it to react in an amidation reaction.

**Scheme 10:**
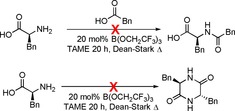
Reactivity of amino acids with carboxylic acids (top) and themselves (bottom).

Interestingly, amino acids do not react with an external carboxylic acid such as phenylacetic acid under our standard catalytic amidation conditions (Scheme 10). This suggests that complexes such as **13** are not reactive at the amine. Similarly, in the absence of an amine reaction partner self condensation of the amino acid to give diketopiperazine did not take place to any significant extent (Scheme 10). Of all the amino acids screened, only proline formed trace amounts of diketopiperazine when subjected to the standard catalytic amidation conditions (<5 % yield over 24 h).

**Scheme 11:**
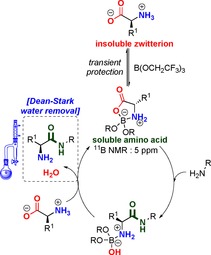
Solubilization of amino acid into solution and subsequent acylation.

From these observations, it can be deduced that, only small quantities of amino acid will be solubilized when employing catalytic amounts of Lewis acid, creating a system in which the amine is in large excess to the solubilised amino acid, which is not reactive as a nucleophile (Scheme 11). This explains how self‐condensation of the amino acid is prevented, and also explains the lack of reactivity of the amino acid with a carboxylic acid. The solubilized amino acid complex **13** is then able to undergo boron‐catalysed amidation with the amine to generate the amino amide product coordinated to the catalyst, which then undergoes exchange with another molecule of free amino acid to continue the cycle. On the basis of our recent studies,^35^ the amidation of the amino acid complex is likely to be mediated by interaction with a second catalyst molecule to form a species with a bridging acylboron unit.

## Conclusions

**Scheme 12:**
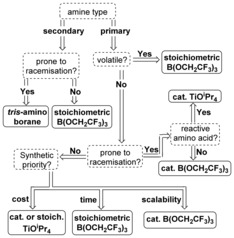
Method selection flowchart for chemoselective amidation of unprotected amino acids. For amides derived from His, Lys or Arg the method using CDI reported by Sharma et al. should be considered.^12^

In summary, we have identified effective methods for the direct amidation of unprotected amino acids with amines by using catalytic or stoichiometric quantities of boron or titanium Lewis acids. In this study, a detailed exploration of the scope of these reactions has been carried out, enabling the advantages and limitations of each approach to be identified. In Scheme 12, we provide a flowchart to enable the best method for a particular amidation reaction to be identified. We hope that this guide will prove useful in promoting the direct amidation of amino acids as a useful transformation for the chemistry community. With burgeoning interest in the development of novel catalytic methods for amide bond formation,^36^ we anticipate that other amidation catalysts may well be applicable to this reaction. We also anticipate that implementation of this synthetic strategy in the pharmaceutical sector can lead to improved cost‐effectiveness and reduced levels of waste in the synthesis of complex medicinally relevant compounds.

## Experimental Section

Experimental procedures,^1^H and ^13^C NMR spectra, and characterization data for all compounds are available in the Supporting Information.

## Conflict of interest

The authors declare no conflict of interest.

## Supporting information

As a service to our authors and readers, this journal provides supporting information supplied by the authors. Such materials are peer reviewed and may be re‐organized for online delivery, but are not copy‐edited or typeset. Technical support issues arising from supporting information (other than missing files) should be addressed to the authors.

Supplementary
