# Supplementary material for: Protecting‐Group‐Free Amidation of Amino Acids using Lewis Acid Catalysts
Source: Chemistry. 2018 Apr 30;24(27):7033–43. doi: 10.1002/chem.201800372 (PMC5969221; doi:10.1002/chem.201800372)
Supplement: Supplementary file 1 — Supplementary [file CHEM-24-7033-s001.pdf]

CHEMISTRY
A European Journal

Supporting Information

Protecting-Group-Free Amidation of Amino Acids using Lewis Acid Catalysts

Marco T. Sabatini,[a] Valerija Karaluka,[a] Rachel M. Lanigan,[a] Lee T. Boulton,[b]
Matthew Badland,[c] and Tom D. Sheppard*[a]

chem_201800372_sm_miscellaneous_information.pdf

Table of contents

1. General methods……………………………………………………………………….…..…2

2. Amidation reactions employing ‘classical’ coupling reagents …………………….….3

3. Calculations of waste generated from amidation procedures…………………………8

4. Procedures for unprotected amino acid amidation ………………..…………………10

4.1 Spectroscopic data ………………..…………………………………..………..14

5. Procedures for trisamino borane amidations ……………..………………………..…..35

5.1 Spectroscopic data ………………..…………………………………..………..36

6. Procedures for sequential condensations ………………..…………………………..….40

6.1 Spectroscopic data ………………..…………………………………..………..41

7. Origins of chemoselectivity ………………..………………………..……………….…..56

8. ^{1}H and ^{13}C NMR spectra …...………………..………………………..……………….…..58

9. ^{1}H and ^{13}C NMR spectra for enantiopurity and HPLC traces …………..…………….…..110

10. References…...………………..……………………..………………………………136

1. General methods

All reagents and solvents were purchased and used as supplied unless otherwise stated. All reactions were carried out at atmospheric pressure with stirring and under air atmosphere unless otherwise indicated. All resins were washed with EtOAc, Et_2O and CH_2Cl_2 and dried *in vacuo* prior to use. *In vacuo* is used to describe evaporation of solvent by Büchi rotary evaporator between 17 ºC and 70 ºC at a pressure of ~ 10 mmHg. All reactions were monitored by TLC or ^{1}H NMR. TLC plates used were pre-coated with silica gel 60 F254 on aluminium (Merck KGaA). The spotted TLCs were visualised by UV light (254 nm or 365 nm) or chemically stained ($KMnO_4$). Column chromatography purification was performed using silica gel (Merck silica gel, 40-60 μm). $[\alpha]_D$ values are given in 10^{-1} deg cm^2 g^{-1}, concentration (*c*) in g per 100 mL. ^{1}H NMR and ^{13}C NMR spectra were recorded at 300, 400, 500 MHz or 600 MHz (for ^{1}H) and 75, 100, 125 MHz or 150 MHz (for ^{13}C) on a Bruker AMX300, AMX400, AMX500 or AMX600 at ambient temperature, unless otherwise indicated. Deuterated solvents for NMR detection used were $CDCl_3$, MeOD or DMSO-*d6* as stated in the spectrum. Peaks are assigned as singlet (s), doublet (d), triplet (t) or multiplet (m). All shifts are reported in parts per million (ppm) and compared against residual solvent signals: $CDCl_3$ (δ = 7.26 ppm, s), DMSO (δ = 2.56 ppm, qn) or MeOD (δ = 4.87, s and 3.31, qn) as the internal standard. Coupling constants (*J*) are quoted in Hertz (Hz) to one decimal place. Mass spectrometry was performed on VG70 SE (EI, CI, ES- modes). Infra-red spectra were obtained using a Perkin-Elmer Spectrum 100 FTIR Spectrometer operating in ATR mode, all frequencies given in reciprocal centimetres (cm^{-1}). Melting points were measured with a Gallenkamp heating block and are uncorrected.

2. Amidation reactions employing ‘classical’ coupling reagents

Attempted amidation reactions of phenylalanine with benzylamine employing ‘classical’ coupling reagents (Table 1)

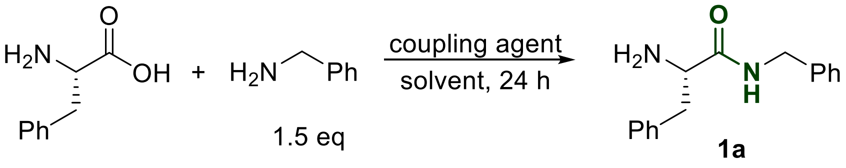

Based on a procedure by Pu *et al*.[1]

Benzylamine (1.5 mmol), HOBt (1.0 mmol), and phenylalanine (1.0 mmol) were suspended in 4.8 mL of water, and NMM (3.7 mmol) was added. The reaction was stirred at room temp. for 10 min and then cooled to 5 °C. EDC (1.1 mmol) was added and the temperature allowed to rise to 25 °C. After stirring for 24 h, the mixture was concentrated in vacuo. 1,4 dimethoxybenzene (1.0 mmol) was added to the flask, and the crude mixture dissolved in DMSO-d_6 and analysed by NMR.

The procedure was also conducted using ethanol instead of water as a solvent.

No desired amino amide **1a** was observed in either case.

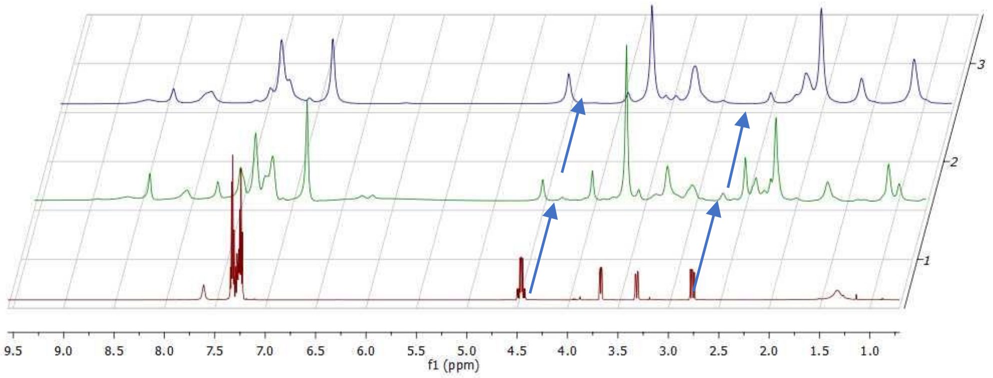

Figure SI.1: crude reaction mixture of amidation procedure in water and ethanol. Spectrum of pure amide is shown in red for comparison.

Based on a procedure by Ho *et al*.[2]

Water (20 mL), phenylalanine (2.0 mmol) and HOBt (2.0 mmol) were added to a solution of benzylamine (3.0 mmol) in CH_2Cl_2 (20 mL). The biphasic mixture was cooled to 5 °C and EDC (2.2 mmol) was added. After stirring for 24 h, the mixture was concentrated *in vacuo*. 1,4 dimethoxybenzene (1.0 mmol) was added to the flask, and the crude mixture dissolved in DMSO-d_6 and analysed by NMR.

The procedure was also conducted using ^{i}PrOAc instead of CH_2Cl_2 as the organic solvent.

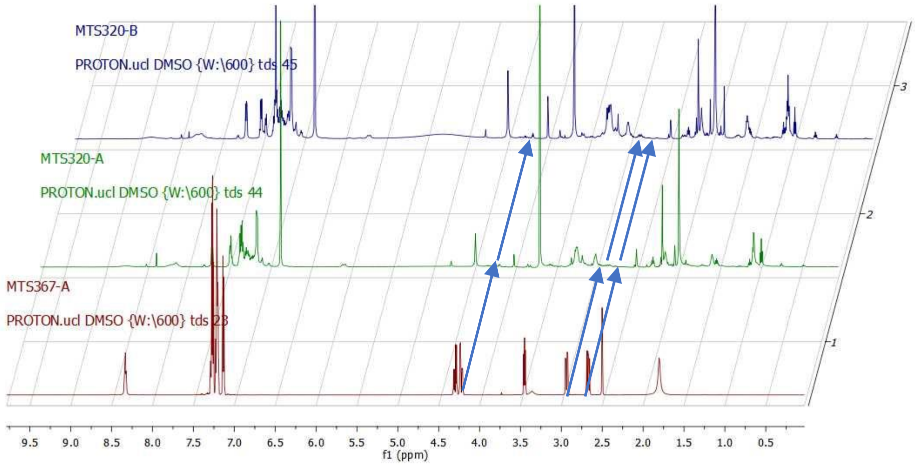

Figure SI.2: crude reaction mixture of amidation procedure in $^{i}PrOAc/H_2O$ and CH_2Cl_2/H_2O. Spectrum of pure amide is shown in red for comparison.

No desired amino amide **1a** was observed in either case.

Based on a procedure by Leggio *et al*.[3]

Phenylalanine (2.0 mmol) was added to a solution of benzylamine (3 mmol), triethylamine (9.0 mmol) and 1,4 dimethoxybenzene (1.0 mmol) in CH_2Cl_2 (10 mL). $SOCl_2$ (2.0 mmol) was added at room temp. and reaction mixture stirred for 24 h. The mixture was concentrated *in vacuo* and the crude mixture dissolved in $CDCl_3$ and analysed by NMR.

The procedure was also conducted without the use of Et_3N.

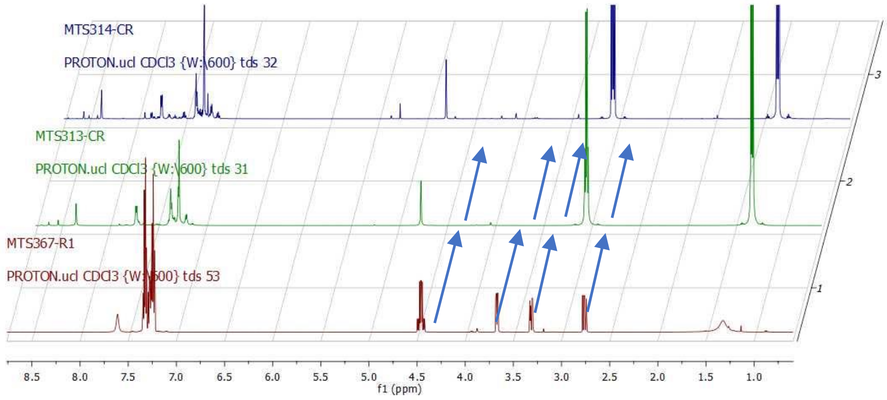

Figure SI.3: Crude reaction mixture of amidation procedure with and without Et_3N. Spectrum of pure amide is shown in red for comparison.

No desired amino amide **1a** was observed in either case.

HATU (2.0 mmol) was added to a suspension of benzylamine (3 mmol) and phenylalanine (2.0 mmol) and 1,4 dimethoxybenzene (1.0 mmol) in DMF (10 mL). The mixture was stirred for 24 h, concentrated *in vacuo* and analysed by NMR in $CDCl_3$.

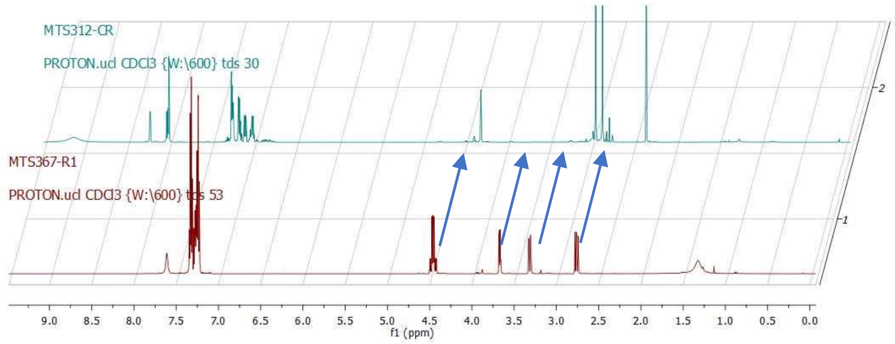

Figure SI.4: Crude reaction mixture of amidation procedure. Spectrum of pure amide is shown in red for comparison.

No desired amino amide **1a** was observed in either case.

T3P (50 w/w% in DMF, 1.5 mmol) was added to a suspension of benzylamine (1.5 mmol) and phenylalanine (1.0 mmol) and 1,4 dimethoxybenzene (1.0 mmol) in DMF (0.5 mL). The mixture was stirred for 24 h at 80 °C, concentrated *in vacuo,* dissolved in DMSO-d_6 and analysed by NMR.

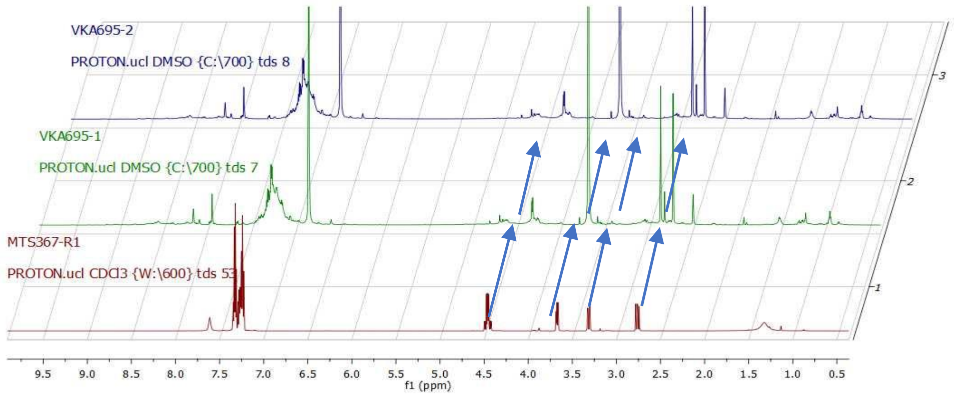

Figure SI.5: Crude reaction mixture of amidation procedure at room temp and at 80 °C. Spectrum of pure amide is shown in red for comparison.

No desired amino amide **1a** was observed in either case.

CDI amidation, based on a procedure by Sharma *et al.*[4]

To a solution of phenylalanine (1.73 mmol) in H_2O (4 mL), CDI (2.08 mmol) was added at ambient temperature and the reaction mixture was stirred for 30 min. benzylamine (2.08 mmol) was then added and the stirring continued for another 24 h at RT, concentrated *in vacuo,* dissolved in DMSO-d_6 and analysed by NMR.

Traces of desired amide (10%) were accompanied by formation of other amidic species (4 total).

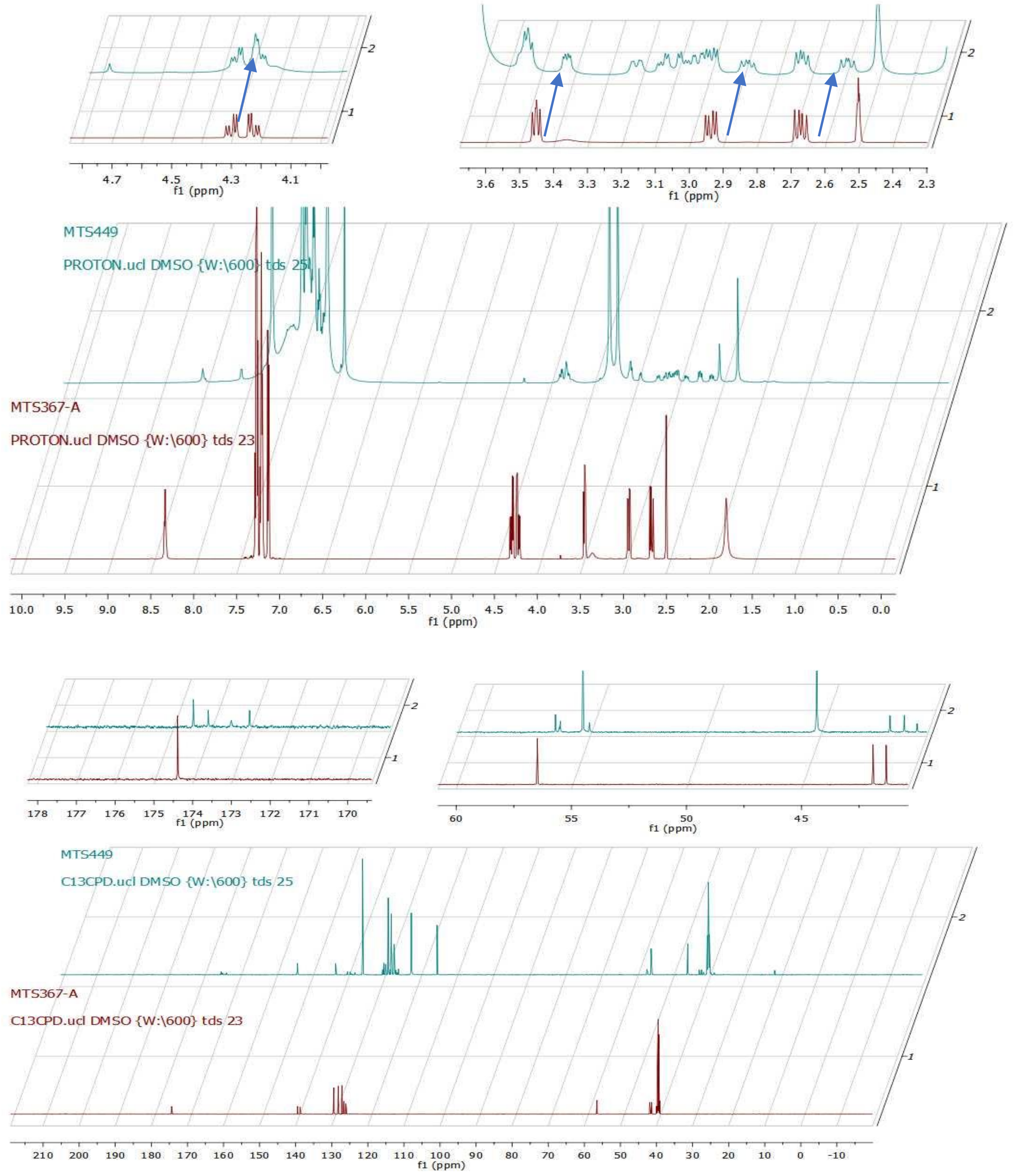

Figure SI.6: Crude reaction mixture of amidation procedure. Spectrum of pure amide is shown in red for comparison.

For comparative purposes, below, are shown ^{1}H NMR's of crude reaction mixtures performed over the course of the reaction optimisation using boron catalysts. The reactions show the predominant formation of desired amino amide over undesired diketopiperazine or dipeptides as was observed with classical coupling reagents (*vide supra*). Even with fewer equivalents of benzylamine (**SI7.3**) and excess amino acid (1.2 eq) relative to benzylamine, the reaction was chemoselective (**SI7.2**).

Superimposed spectra for catalytic reactions, respectively:

SI.7.1: 1.5 eq benzylamine, TAME (1 M), 3,4,5-Trifluorophenylboronic acid (20 mol%), 24 h (NMR in DMSO-d_6)

SI.7.2: 0.83 eq benzylamine, TAME (0.5 M), $B(OCH_2CF_3)_3$ (20 mol%), 24 h (NMR in $CDCl_3$)

SI.7.3: 1.1 eq benzylamine, TAME (0.5 M), $B(OCH_2CF_3)_3$ (20 mol%), 24 h (NMR in DMSO-d_6)

SI.7.4: 3.0 eq benzylamine, CPME (0.5 M), $B(OCH_2CF_3)_3$ (10 mol%), 24 h (NMR in DMSO-d_6)

SI.7.5*: Purified sample of amino amide* (NMR in DMSO-d_6)

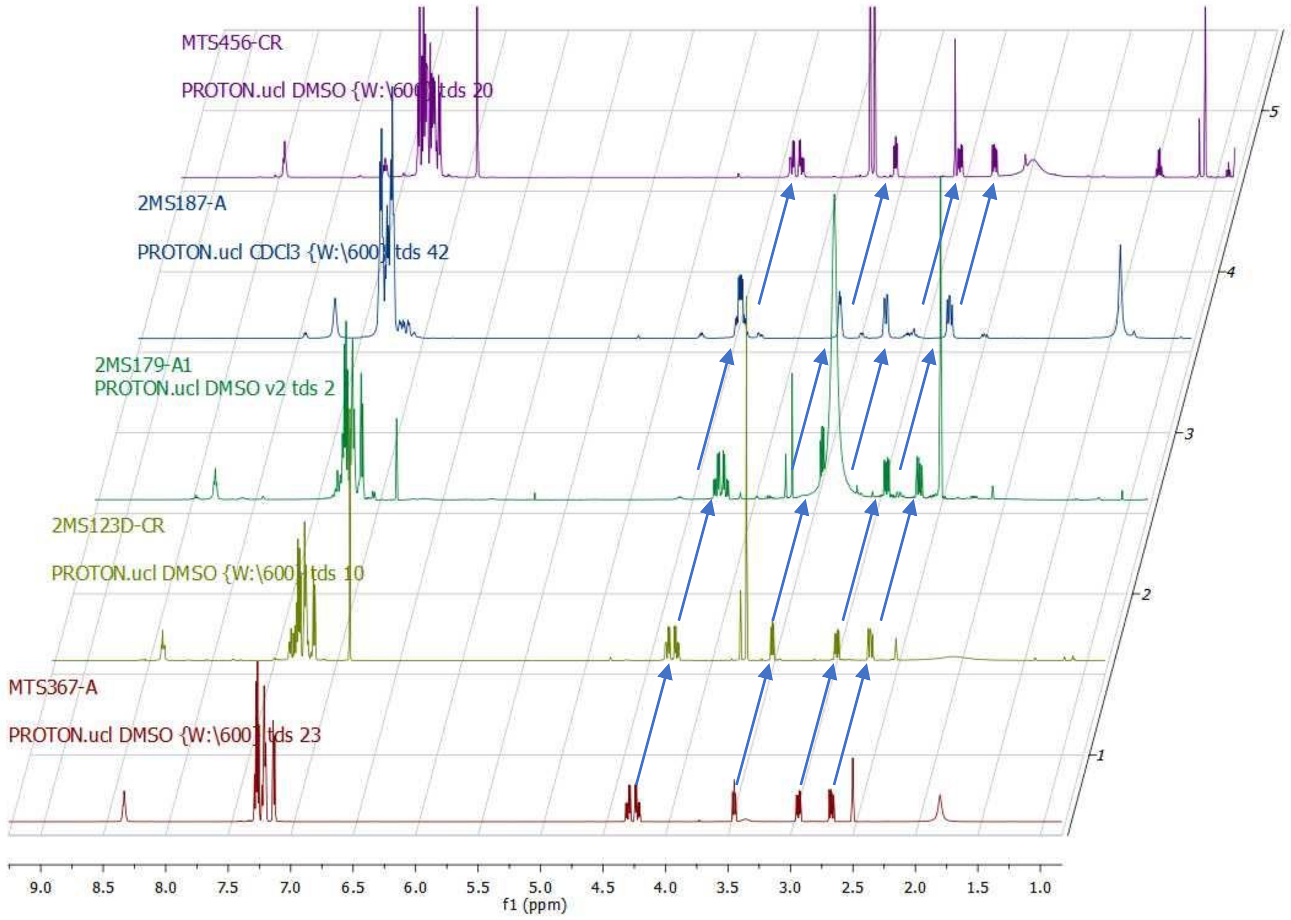

Figure SI.7: Crude reaction mixtures from borate-catalysed amidation reactions.

3. Calculations of waste generated from amidation procedures

Procedure by H. Kohn *et al*.[5]

Supporting info*:* http://pubs.acs.org/doi/suppl/10.1021/jm2004305

(*R*)-2-N-(Benzyloxycarbonyl)amino-3-phenylpropionic acid ((*R*)-92). Benzyl chloroformate (5.09 mL, 36.35 mmol) and aqueous 4 M NaOH (12 mL) were added simultaneously over a 30 min period to a vigorously stirred solution of D-phenylalanine (5.00 g, 30.29 mmol) dissolved in aqueous 4 M NaOH (10 mL)/aqueous 1 M $NaHCO_3$ (30 mL) at 0 °C. The mixture was then warmed to room temperature and stirred overnight (18 h). The reaction was washed with Et2O (2 × 100 mL) and then the aqueous mixture was added to a stirred mixture of aqueous 4 M HCl (66 mL) and EtOAc (100 mL). The aqueous layer was separated and then extracted with EtOAc (3 × 100 mL). All of the organic layers were combined, successively washed with H_2O (3 × 100 mL) and brine (2 × 100 mL), dried (Na_2SO_4), and then evaporated in vacuo to give the desired product (6.74 g, 74%).

30.29 mmol scale

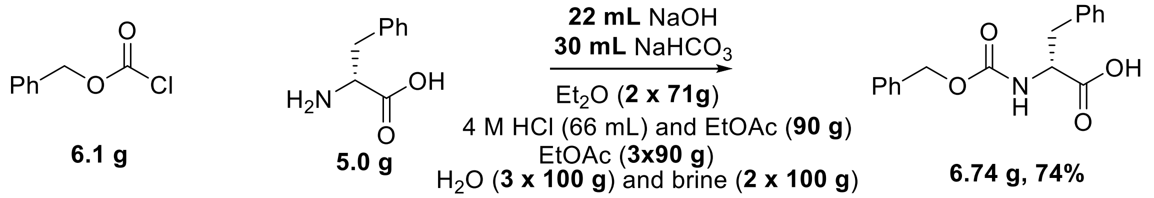

An anhydrous THF solution of carboxylic acid (0.5–2.0 M [middle point chosen for green metric studies]) was cooled to −78 °C in a dry ice/acetone bath under an inert atmosphere (Ar or N_2), and NMM (2.39 mL, 21.73 mmol) was added. After the mixture was stirred (2–10 min), IBCF (2.37 mL, 18.38 mmol), was added leading to the precipitation of a white solid. The reaction was allowed to proceed for an additional 15–25 min, and then benzylamine (1.92 mL, 17.55 mmol) was added at −78 °C. The reaction mixture was allowed to stir at room temperature (1.5 h), and then the insoluble salts were filtered. The organic layer was concentrated in vacuo, and recrystallized from hot EtOAc to give the desired compound (4.67 g, 72%) as an off-white solid.

16.7 mmol scale

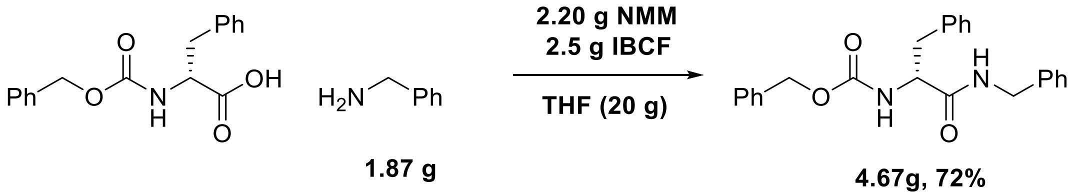

Using Pd Catalyzed Hydrogenation. A MeOH solution (90 mL) of Cbz-protected PAAD (3.50 g, 9.02 mmol)was hydrogenated (1 atm) in the presence of 10% Pd-C (0.35 g) at room temperature (3 h–7 d). The mixture was filtered through a bed of Celite®, the filtrate was evaporated in vacuo, and the product was purified by column chromatography (SiO_2; 1:10 $MeOH/CH_2Cl_2$) to give the desired compound (2.22 g, 97%) as a pale yellow solid.

9.02 mmol

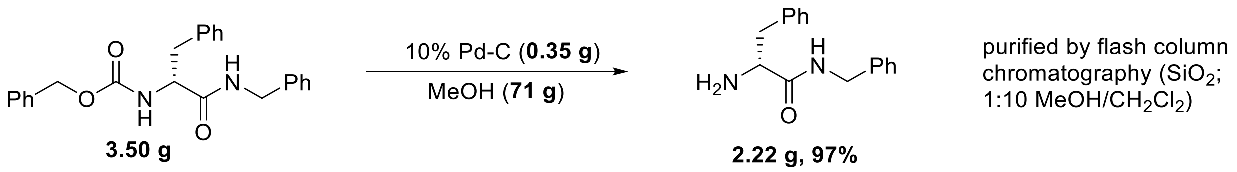

Procedure from B. Ramalingam *et al*.[6]

Full text: *DOI: 10.1055/s-2007-965897*

TBTU (6.78 g, 21.1 mmol) was added to a solution of Boc-protected amino acid (21.1 mmol) in CH_2Cl_2 (100 mL). After 5 min, DIPEA (22.0 mL, 128 mmol) and amine (21.1 mmol) were added sequentially and stirred overnight. The mixture was diluted with CH_2Cl_2 (200 mL), washed with H_2O (250 mL) and the organic layer was separated. The organic layer was washed with 1 M HCl (3 × 200 mL), separated and dried (Na_2SO_4). The crude product was purified by flash chromatography (silica gel) to give a white solid.

21.1 mmol scale

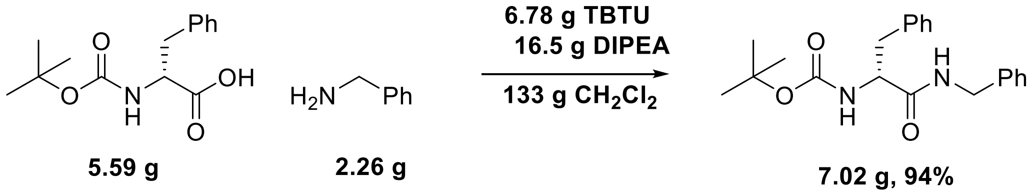

The Boc-protected amide 7 (14.3 mmol) was dissolved in CH_2Cl_2 (50 mL) and stirred for 2 h after the addition of TFA (16.5 mL, 215 mmol). The excess reagent and the solvent were removed under vacuum, redissolved in CH_2Cl_2 (100 mL) and cooled to 0 °C. Sat. Na_2CO_3 soln was added slowly and the resulting organic layer was washed with aq $NaHCO_3$ (3 × 100 mL). The separated organic phase was dried (Na_2SO_4) and concentrated to afford a white solid.

14.3 mmol scale

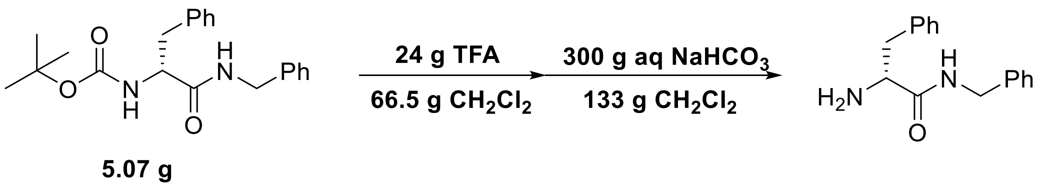

4. Procedures for unprotected amino acid amidation

Stoichiometric unprotected amino acid amidation

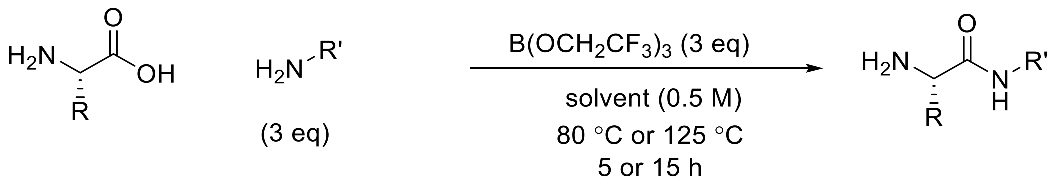

Method A: All reactions were performed on 0.5 or 1.0 mmol scale. An unprotected amino acid (1 eq) and amine (3 eq) were stirred at 80 °C or 125 °C in CPME (0.5 M, unless stated otherwise) with $B(OCH_2CF_3)_3$ (3 eq) for 5 or 15 h. Upon completion, the mixture was diluted with EtOAc or CH_2Cl_2 (3 mL) and water (0.5 mL). Amberlite IRA-743 and Amberlyst A-26(OH) were added and stirred for 30 min. The mixture was dried over $MgSO_4$ and then filtered. The solids were washed with EtOAc (3 × 20 mL) and the product concentrated *in vacuo* to give the amino amide. Amides derived from non-volatile amines were purified either by trituration (Et_2O) or column chromatography as stated below.

Method A [Dropwise addition of Borate]: A solution of $B(OCH_2CF_3)_3$ (3.0 mmol, 3.0 eq) in CPME (1 mL, unless stated otherwise) was added dropwise to a mixture of an unprotected amino acid (1.0 mmol, 1.0 eq) and propylamine (3.0 mmol, 3.0 eq) in CPME (1 mL) over 1 h at 80 °C or 125 °C. The resulting mixture was stirred for 5 or 15 h. Upon completion, the mixture was diluted with EtOAc or CH_2Cl_2 (3 mL) and water (0.5 mL). Amberlite IRA-743 and Amberlyst A-26(OH) were added and stirred for 30 min. The mixture was dried over $MgSO_4$ and then filtered. The solids were washed with EtOAc (3 × 20 mL) and the product concentrated *in vacuo.* The volatile propylamine was removed *in vacuo* by the addition of $CHCl_3$ to get the clean product. Amides with different amines were purified either by trituration (Et_2O) or column chromatography as stated.

Method A [(Ti(O^{i}Pr)$_4$]: As described above for Method A, but with 1 eq of $Ti(O^iPr)_4$ instead of $B(OCH_2CF_3)_3$.

Catalytic unprotected amino acid amidation

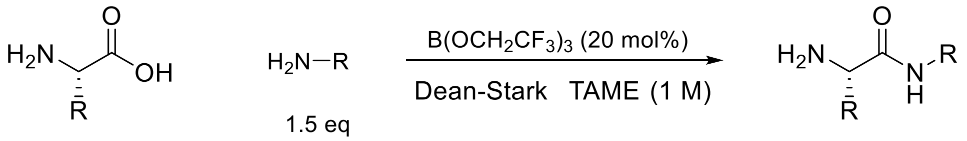

Method B: A stirred suspension of an amine (7.5 mmol) and unprotected amino acid (5 mmol) in TAME (2.5 mL) with a Dean-Stark was heated to reflux (bp, 86 °C) and $B(OCH_2CF_3)_3$ (1 mmol, 2.5 mL of a 0.4 M solution in TAME) was added through the Dean-Stark. An air condenser was fitted, and the reaction mixture stirred for 24 hours. Upon completion, the reaction mixture was concentrated *in vacuo* and dry loaded onto silica gel for column chromatography.

Method B [(Ti(O^{i}Pr)$_4$]: As described above for Method A, but with 20 mol% $Ti(O^iPr)_4$ instead of $B(OCH_2CF_3)_3$.

Note: Naturally occurring L-Amino acids were used, unless specified otherwise. Where amino acids were not available as fine powders, they were ground with mortar and pestle (for amino acids obtained from Alfa Aesar or Calbiochem: Valine, Leucine, Isoleucine, Sarcosine, Methionine, and Glutamic acid).

Design of experiments optimisation

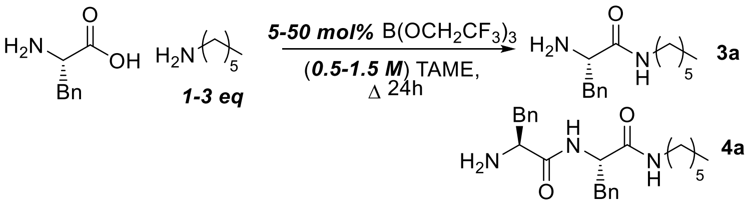

Following general procedure B, with a carousel 6 reaction station with integrated Dean-Stark traps. Yields were measured by HPLC against an internal standard (phenanthrene).

Amine Equiv	Catalyst mol %	Solvent vol	Mmols of amino acid	Yield after 24h	Yield of Di-amide after 24 hours
1	0.5	4	37.6	*56%*	6.9%
2	0.275	8	18.8	*49%*	4.6%
1	0.05	12	12.7	*32%*	3.3%
1	0.05	4	37.6	*22%*	1.5%
3	0.05	4	37.6	*42%*	2.0%
1	0.5	12	12.7	*47%*	6.9%
2	0.275	8	18.8	*47%*	4.5%
3	0.5	12	12.7	*68%*	5.1%
3	0.05	12	12.7	*47%*	2.4%
3	0.5	4	37.6	*59%*	4.0%

Note: water removal/stirring was inefficient, leading to lower yields relative to when reaction was conducted in a standard reaction set-up. All reaction flasks contain 25 mL of TAME

DoE study on the direct amidation reaction to form **3a**.

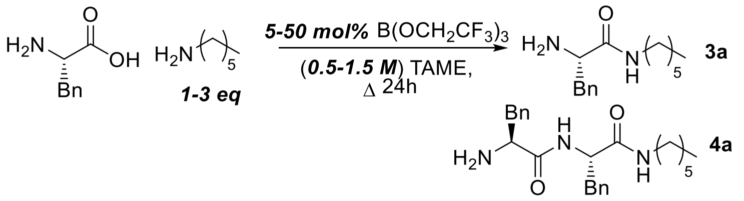

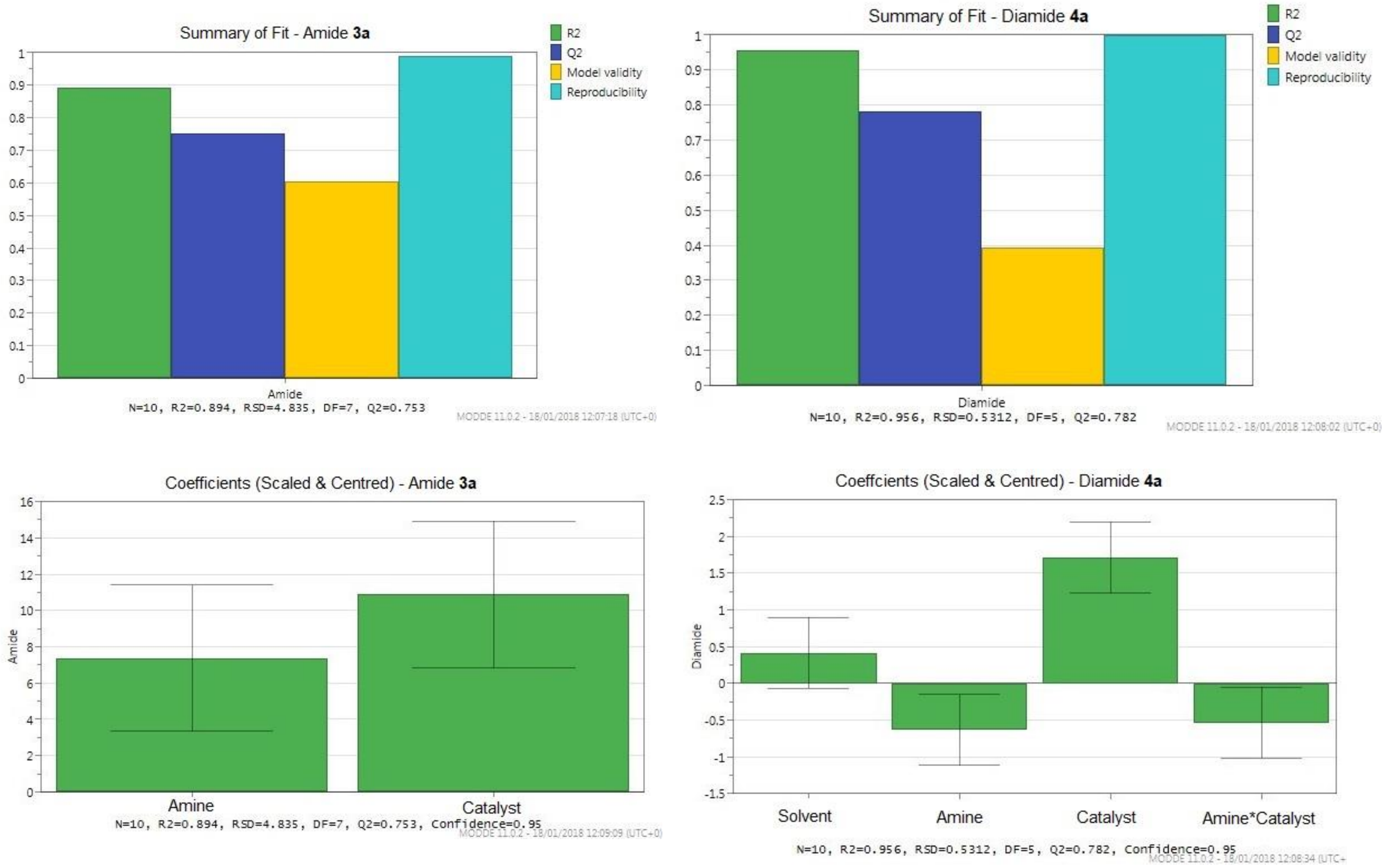

'Summary of Fit' and 'Coefficient' plots for amide **3a** and diamide **4a**. The 'Summary of Fit' plots demonstrate that the two models obtained from the DoE study provide a high quality fit to the data. The coefficient plots indicate the significant factors affecting the yield of each product, showing the average effect on the yield of a product when a factor is increased from the value at the design centre point to the upper limit of the design. Factors: Am (amine loading, 1-3 eq.); Cat (catalyst loading, 5-50 mol%); Sol (solvent: 4 to 12 volumes, equivalent to varying the reaction concentration from 1.5 M to 0.5 M).

Substrates from Tables 2-4: The synthesis of compounds **2a-2w** and **2z** is described in our previous report.[7] Where these compounds have been synthesised using a different method (i.e. $Ti(OiPr)_4$), we include the spectra for enantiopurity).

(*S*)-2-Amino-2-phenyl-*N*-propylacetamide (2x)

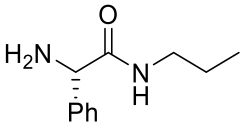

Prepared according to general procedure A from phenylglycine (151 mg, 1.0 mmol) and propylamine (177 mg, 3.0 mmol), at 80 °C for 15 h to yield a yellow oil (141 mg, 73%, er 55:45, determined using chiral HPLC, Chiralcel OD-H (n-Hex/PrOH; 97/3, 0.5 ml/min; 254 nm; R_t(S) = 15.79 min)).

$[\alpha]_D^{25}$ +3.1 (*c* 0.64, MeOH);

ν_{max} (film/cm^{-1}) 3294, 2961, 2929, 2873, 1647, 1522;

δ_H (300 MHz, $CDCl_3$) 7.43-7.25 (m, 5H, Ar*H*), 7.07 (s, 1H, N*H*), 4.51 (s, 1H, COC*H*), 3.22-3.21 (m, 2H, NHCH_2), 2.01 (s, 2H, NH_2),1.53-1.51 (m, 2H, C$H_2$$CH_3$), 0.89 (t, *J* = 7.4 Hz, 3H, CH_3);

δ_C (75 MHz, $CDCl_3$) 173.0, 141.3, 129.0, 128.1, 127.0, 60.0, 41.1, 23.0, 11.5;

LRMS (ES+): 193 ($[M+H]^+$, 100);

HRMS: Found (ES+): $[M+H]^+$ 193.1343 $C_{11}H_{17}N_2O$, requires 193.1341;

(*S*)-2-Amino-4-hydroxy-*N*-propylbutanamide (2y)

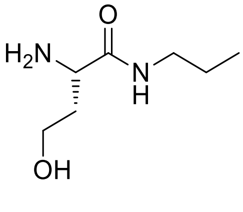

Prepared according to general procedure A, from homoserine (119 mg, 1.0 mmol) and propylamine (177 mg, 3 mmol), at 125 °C for 15 h to yield a yellow oil (111 mg, 69%, er 84:16, determined using Marfey's reagent);

$[\alpha]_D^{25}$ +12.1 (*c* 0.62, MeOH);

ν_{max} (film/cm^{-1}) 3287, 3260, 2966, 2836, 1645, 1542;

δ_H (300 MHz, $CDCl_3$) 7.27 (br s, 1H, N*H*), 3.83-3.80 (m, 2H, OHC*H*$_2$), 3.57 (t, *J* = 6.6 Hz, 1H, COC*H*), 3.23 (app. q, *J* = 6.5 Hz, 2H, NHC*H*$_2$), 2.17 (br s, 3H, N*H*$_2$ and O*H*), 1.94-1.92 (m, 1H, CHC*H*H),1.85-1.83 (m, 1H, CHC*H*H), 1.57-1.54 (m, 2H, C*H*$_2$CH$_3$), 0.93 (t, *J* = 7.4 Hz, 3H, CH$_3$);

δ_C (75 MHz, $CDCl_3$) 175.3, 61.0, 54.6, 41.0, 38.0, 22.9, 11.5;

LRMS (ES+) 161 ([M+H]$^+$, 100);

HRMS: Found (ES+): [M+H]$^+$ 161.1304, $C_7H_{17}N_2O_2$, requires 161.1290.

Substrates from Figure 2: The synthesis of compounds **1a, 1b, 1d, 1f, 1g-j, 1l,** and **1w** is described in our previous reports.[7,16] Where these compounds have been synthesised using a different method (Ti(OiPr)$_4$) we include the details for their synthesis and enantiopurity data.

(S)-2-amino-N-benzyl-3-phenylpropanamide (1a)

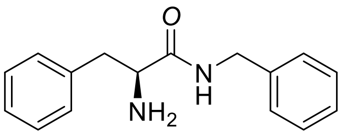

Synthesis using B(OCH_2CF_3)$_3$ described in our previous report.[16]

Prepared according to general procedure B [(Ti(O^{i}Pr)$_4$], from phenylalanine (0.825 g, 5.0 mmol) and benzylamine (0.818 mL, 7.5 mmol) with 20 mol% Ti(O^{i}Pr)$_4$ for 24 h and further purified by flash column chromatography (1.08 g, 85% er >95:5, determined using Marfey's reagent);

Spectroscopic data described in our previous report.[16]

(S)-2-amino-N-benzyl-3-methylbutanamide (1b)

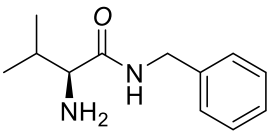

Synthesis using B(OCH_2CF_3)$_3$ described in our previous report.[16]

Prepared according to general procedure B [(Ti(O^{i}Pr)$_4$], from valine (0.234 g, 2.0 mmol) and excess benzylamine (0.655 mL, 6.0 mmol) with 15 mol% Ti(O^{i}Pr)$_4$ in CPME (5 mL) for 24 h

and further purified by flash column chromatography (101 mg, 25% er >95:5, determined using Marfey's reagent);

(S)-2-Amino-*N*-benzyl-3-(4-hydroxyphenyl)propanamide (1c)

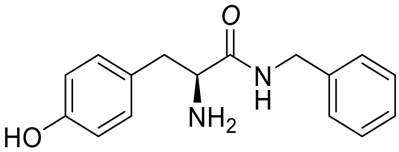

Prepared according to general procedure B, from Tyrosine (0.906 g, 5.0 mmol) and benzylamine (0.818 mL, 7.5 mmol) for 24 h. Product was purified by column chromatography (CH_2Cl_2:MeOH, 9:1) and recrystallised from hot MeOH, to yield a white solid (501 mg, 37% er 95:5, determined using chiral HPLC);

$[\alpha]_D^{20}$ +6.2 (*c* 1.0, MeOH);

mp 169-171 °C;

ν_{max} (solid/cm^{-1}) 3334, 2925, 1635, 1543, 1515;

δ_H (500 MHz, MeOD) 7.33-7.19 (m, 3H, Ar*H*), 7.07 (m, 2H, Ar*H*), 6.99 (m, 2H, Ar*H*), 6.89 (m, 2H, Ar*H*), 4.39 (dd, *J* = 15.0, 6.5 Hz, 1H, NHC*H*H), 4.23 (dd, *J* = 15.0, 6.8 Hz, 1H, NHCH*H*), 3.50 (m, 1H, C*H*CH_2), 2.84-2.87 (m, 1H, CHCH*H*), 2.76-2.79 (m, 1H, CHC*H*H);

δ_C (125 MHz, MeOD) 179.2, 160.0, 142.1, 134.0, 132.0, 131.9, 131.2, 130.7, 118.9, 60.7, 46.5, 44.4;

HRMS (ES+): Found (ES+): [M+H]+ 271.1448, $C_{16}H_{18}N_2O_2$+H, requires 271.1447;

Compound was determined to have an enantiopurity >95:5 by chiral HPLC (n-Hex/PrOH; 95/5, 0.7 ml/min; 218 nm; R_t(S) = 15.6 min.

2-Amino-*N*-benzylacetamide (1e)

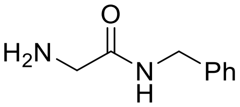

Prepared according to General Procedure B from Glycine (0.375 g, 5.0 mmol) and benzylamine (1.64 mL, 15 mmol), and further purified by flash column chromatography (6% MeOH:CH_2Cl_2) to yield an amide as a white solid (369 mg, 45%);

mp 140-143 °C [lit 142-144 °C][4]

ν_{max} (solid/cm^{-1}) 3348, 3269, 2910, 1680, 1634, 1534;

δ_H (600 MHz, MeOD) 7.34-7.27 (m, 4H, Ar*H*), 7.26-7.22 (m, 1H, Ar*H*), 4.40 (s, 2H, PhC*H*$_2$), 3.34 (s, 2H, NH$_2$C*H*$_2$);

δ_C (150 MHz, MeOD) 174.3, 139.8, 129.6, 128.6, 128.3, 44.7, 44.0;

LRMS (ES+): 165 ([M+H]$^+$, 100).

(S)-2-amino-N-benzyl-4-methylpentanamide (1g)

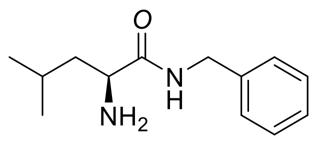

Synthesis using B(OCH$_2$CF$_3$)$_3$ described in our previous report.[16]

Prepared according to general procedure B [(Ti(O^{i}Pr)$_4$], from leucine (0.262 g, 2.0 mmol) and excess benzylamine (0.655 mL, 6.0 mmol) with 15 mol% Ti(O^{i}Pr)$_4$ in CPME (5 mL) for 24 h and further purified by flash column chromatography (399 mg, 90% er 94:6, determined using Marfey's reagent);

(*R*)-2-Amino-*N*-benzyl-3-mercaptopropanamide (1k)

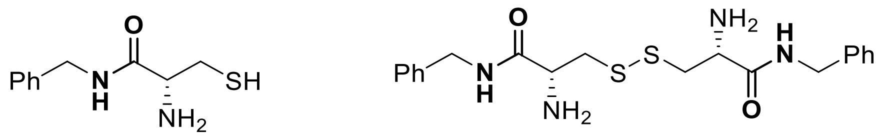

Prepared according to general procedure B, from Cysteine (0.605 g, 5.0 mmol) and benzylamine (0.818 mL, 7.5 mmol) for 24 h. Overall conversion of the reaction was calculated using 1,4 dimethoxybenzene as an internal standard (90%). Mixture was subsequently purified by column chromatography to give a mixture of amide (**1k**), and oxidised **1k** (with racemisation, diastereoisomers). NMR's of product mixture purified by column chromatography are provided below.

Analysis by mass spectrometry confirmed formation of **1k**.

ν_{max} (film/cm^{-1}) 3284, 1639, 1516, 1453;

LRMS (ES+): 419.2 ([M+H]$^+$, 100);

HRMS: Found (ES+): [M+H]$^+$ 211.0898 $C_{10}H_{15}N_2OS$, requires 211.0905;

HRMS: Found (ES+): [M+H]$^+$ 419.1533 $C_{20}H_{26}N_4O_2S_2$, requires 419.1575.

(*S*)-2-Amino-*N*-benzyl-3-hydroxypropanamide (1m)

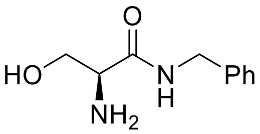

This compound was prepared according to general procedure B from Serine (420 mg, 4 mmol) and benzylamine (0.654 mL, 6 mmol) and purified by column chromatography (CH_2Cl_2:MeOH, 96:4) to yield a brown solid (176 mg, 23%).

$[\alpha]_D^{20}$ −2.0 (*c* 1.0, $CDCl_3$);

mp 51-53 °C;

ν_{max} (solid/cm^{-1}) 3273, 3087, 3030, 2846, 1638, 1527, 1497;

δ_H (500 MHz, $CDCl_3$) 7.83 (br s, 1H, N*H*), 7.24–7.33 (m, Ar*H*), 4.40 (m, 2H, CH_2NH), 3.84 (dd, *J* = 10.9, 5.3 Hz, 1H, C*H*HOH), 3.71 (dd, *J* = 10.9, 5.2 Hz, 1H, C*H*HOH), 3.45 (dd, *J* = 4.8, 4.9 Hz, C*H*), 2.40 (br s, 3H, O*H*, NH_2);

δ_C (125 MHz, $CDCl_3$) 173.8, 138.1, 128.8, 127.7, 127.5, 65.2, 56.1, 43.2;

LRMS (ES+): 195.1 ([M]$^+$, 100), 196.1 ([M+2H]$^+$, 10).

(*S*)-*N*-Benzyl-5-oxopyrrolidine-2-carboxamide (1n)

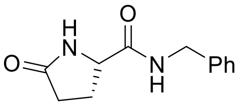

Prepared according to General Procedure B from Glutamic acid (735, 2 mmol) and benzylamine (327 µL, 3 mmol) for 24 hours to yield a white solid (398 mg, 91%, er > 99:1, determined using chiral shift reagent);

mp 134-135 °C [lit. 138.3 °C];[8]

$[\alpha]_D$ +218.1 (*c* 0.21, MeOH, 25 °C);

ν_{max} (solid/cm^{-1}) 3276, 3226, 3097, 1681, 1645, 1574, 1419;

δ_H (600 MHz, $CDCl_3$) 7.38 (s, 1H, NH), 7.29-7.19 (m, 5H, ArH), 4.37 (dd, *J* = 14.7, 5.7 Hz, 1H, PhC*H*H), 4.32 (dd, *J* = 14.7, 5.7 Hz, 1H, PhCH*H*), 4.09 (dd, *J* = 8.9, 4.5 Hz, 1H), 2.43-2.31 (m, 1H), 2.19 (m, 2H), 2.09 (m, 1H);

δ_C (150 MHz, $CDCl_3$) 179.8, 172.4, 138.1, 128.8, 127.9, 127.6, 57.3, 43.5, 29.4, 25.9;

LRMS (ES): 241 ([M+Na]$^+$, 100);

HRMS: Found (ES): $[M+Na]^+$ 241.0943 $C_{12}H_{14}N_2O_2Na$, requires 241.0953.

Data in accordance with the literature.[9]

(*S*)-2-Amino-*N*[1],*N*[4]-dibenzylsuccinamide (1o)

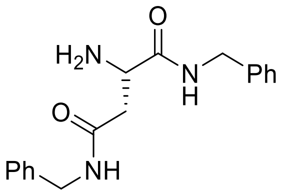

Prepared according to General Procedure B from Aspartic acid (665 mg, 5 mmol) and benzylamine (1.64 mL, 15 mmol), and further purified by flash column chromatography to yield amide as a white solid (326 mg, 21%, er 72:28, determined using Marfey's reagent);

mp 157-159 °C;

ν_{max} (solid/cm^{-1}) 3273, 3082, 3059, 3028, 1627, 1543;

δ_H (600 MHz, $CDCl_3$) 7.93 (t, *J* = 5.4 Hz, 1H, N*H*), 7.35-7.20 (m, 10H, Ar*H*), 7.05 (s, 1H, NH), 4.38-4.26 (m, 4H, 2 × PhCH_2), 3.67 (dd, *J* = 7.6, 4.2 Hz, 1H, C*H*), 2.69 (dd, *J* = 14.7, 4.2 Hz, 1H, CHC*H*H), 2.58 (dd, *J* = 14.7, 7.6 Hz, 1H, CHCH*H*), 1.93 (s, 2H, NH_2);

δ_C (150 MHz, $CDCl_3$) 174.1, 171.2, 138.4, 138.3, 128.8, 128.8, 127.8, 127.7, 127.5, 127.5, 52.8, 43.5, 43.3, 40.7;

LRMS (ES+): 312 ($[M+H]^+$, 100);

HRMS: Found (ES+): $[M+H]^+$ 312.1713 $C_{18}H_{22}N_3O_2$, requires 312.1712.

(*S*)-2-Amino-*N*-benzyl-3-(1*H*-imidazol-4-yl)propenamide (1r)

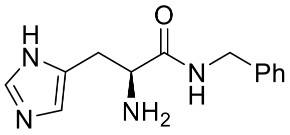

Prepared according to General Procedure B from Histidine (775 mg, 5 mmol) and benzylamine (0.818 mL, 7.5 mmol) for 24 hours to yield a yellow solid (430 mg, 35%)

Prepared according to General Procedure B from Histidine (775 mg, 5 mmol) and benzylamine (0.818 mL, 7.5 mmol) with 30 mol% $B(OCH_2CF_3)_3$ for 24 hours to yield a pale-yellow oil which solidified upon prolonged standing (728 mg, 60%)

$[\alpha]_D$ −1.2 (*c* 1.0, CH_3Cl, 25 °C);

mp 70-71 °C;

ν_{max} (solid/cm^{-1}) 3015, 2930, 2873, 2853, 1649, 1547, 1498;

δ_H (600 MHz, $CDCl_3$) 8.05 (t, *J* = 6.0 Hz, 1H, ArH), 7.44 (d, *J* = 0.9 Hz, 1H, ArH), 7.18-7.27 (m, 3H, ArH), 7.13 (d, *J* = 7.13 Hz, 2H, ArH), 6.74 (s, 1H, ArH), 4.66 (br s, 3H, N*H,* N*H₂*), 4.37 (dd, *J* = 15.0, 5.9 Hz, 1H, CONHC*H*H), 4.34 (dd, *J* = 15.0, 5.9 Hz, 1H, CONHC*H*H), 3.64 (dd, *J* = 7.9, 4.5 Hz, 1H, CH_2C*H)*, 3.02 (dd, *J* =14.6, 4.3 Hz, 1H, CH*H*CH), 2.84 (dd, *J* =14.6, 7.9 Hz, 1H, CH*H*CH);

δ_C (150 MHz, $CDCl_3$) 174.8, 138.3, 135.3, 132.5, 128.8, 127.6, 127.5, 119.8, 55.1, 43.2, 32.0;

LRMS (ESI+) 245.1 ($[M+H]^+$, 100), 489.3 ($[2M+H]^+$, 10).

Data in accordance with the literature.[10]

(*S*)-3-Aminoazepan-2-one (1s)

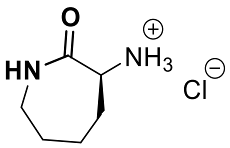

Prepared according to General Procedure B from Lysine (2.92 g, 20 mmol) for 24 hours, purified by reverse phase column chromatography (5:95 MeCN:H_2O, 0.1% NH_3) and further reacted with a solution of HCl in Et_2O to give aminocaprolactam.HCl **6s** as a white solid (740 mg, 29%, 80:20 er determined using Marfeys reagent).

ν_{max} (film/cm^{-1}) 3176, 3005, 1662, 1564;

δ_H (400 MHz, DMSO-d_6) 7.65 (br s, 1H), 3.47 (d, *J* = 10.8 Hz, 1H), 3.04-3.10 (m, 2H), 1.88-1.62 (m, 4H), 1.39-1.12 (m, 2H);

δ_C (100 MHz, DMSO-d_6) (75 MHz, DMSO-d6) 177.5, 52.8, 40.5, 33.9, 29.0, 27.8;

LRMS (ES+) 129.1 ($[M+H]^+$, 100);

Data in accordance with the literature.[11]

3-Amino-*N*-benzyl-3-phenylpropanamide (1x)

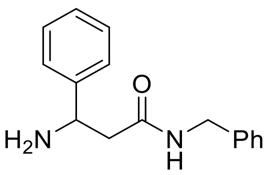

Prepared according to General Procedure B from DL-beta-Phenylalanine (825 mg, 5 mmol), and benzylamine (0.818 mL, 7.5 mmol) for 24 hours, purified by column chromatography (95:5 CH_2Cl_2:MeOH) and further purified by crystallisation from PhMe to yield a white solid (739 mg, 58%);

mp 74-75 °C;

ν_{max} (solid/cm^{-1}) 3291, 2875, 1641, 1522, 1452;

δ_H (600 MHz, $CDCl_3$) 7.35-7.25 (m, 9H, Ar*H*, N*H*), 7.23-7.21 (m, 2H, Ar*H*), 4.46 (dd, *J* = 14.8, 5.7 Hz, 1H, C*H*HPh), 4.42 (dd, *J* = 14.8, 5.7 Hz, 1H, C*H*HPh), 4.38 (dd, *J* = 8.7, 4.8 Hz, 1H, C*H*Ph), 2.57 (dd, *J* = 15.3, 4.8 Hz, 1H, C*H*HCON),), 2.57 (dd, *J* = 15.3, 8.7 Hz, 1H, C*H*HCON), 1.82 (br s, 2H, N*H*$_2$);

δ_H (150 MHz, $CDCl_3$) 171.3, 145.2, 138.5, 128.9, 128.8, 127.8, 127.5, 127.5, 126.0, 53.2, 45.5, 43.5;

LRMS (ES+) 255.1 ([M+H]$^+$, 100);

HRMS: Found (ES+): [M+H]$^+$ 255.1487 $C_{16}H_{19}N_2O$, requires 255.1497.

tert-Butyl (*S*)-3-amino-4-(benzylamino)-4-oxobutanoate (1y)

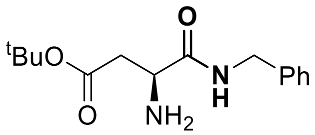

Prepared according to General Procedure B from O^{t}Bu-Aspartic acid (945 mg, 5 mmol) and benzylamine (0.818 mL, 7.5 mmol) with 20 mol% $B(OCH_2CF_3)_3$ for 24 hours and purified by column chromatography (95:5, CH_2Cl_2:MeOH) to yield a pale yellow oil (1.10 g, 79%, 75:25 er determined using Marfeys reagent)

Prepared according to General Procedure B from O^{t}Bu-Aspartic acid (945 mg, 5 mmol) and benzylamine (0.818 mL, 7.5 mmol) with 10 mol% $B(OCH_2CF_3)_3$ for 24 hours and purified by column chromatography (95:5, CH_2Cl_2:MeOH) to yield a pale yellow oil (897 mg, 65%, 87:13 er determined using Marfeys reagent)

Prepared according to General Procedure B from O^{t}Bu-Aspartic acid (945 mg, 5 mmol) and benzylamine (0.818 mL, 7.5 mmol) with 20 mol% $B(OCH_2CF_3)_3$ for 8 hours and purified by column chromatography (95:5, CH_2Cl_2:MeOH) to yield a pale yellow oil (1.07 g, 77%, 84:16 er determined using Marfeys reagent)

$[\alpha]_D$ −15.1 (*c* 1.0, CH_3Cl, 25 °C);

ν_{max} (film/cm^{-1}) 3314, 2976, 1723, 1670;

δ_H (400 MHz, $CDCl_3$) 7.74 (br s, 1H, N*H*), 7.34-7.25 (m, 5H, Ar*H*), 4.44 (d, *J* = 5.9 Hz, 2H, CH_2Ph), 3.68 (dd, *J* = 8.3, 3.9 Hz, 1H, CH*H*), 2.87 (dd, *J* = 16.6, 3.9 Hz, 1H, CH*H*), 2.59 (dd, *J* = 16.6, 8.3 Hz, 1H, C*H*), 1.73 (br s, 2H, NH_2), 1.45 (s, 9H, $(CH_3)_3$);

δ_H (100 MHz, $CDCl_3$) 173.4, 171.3, 138.4, 128.6, 127.7, 127.4, 81.2, 52.1, 42.2, 43.2, 40.6, 28.1;

LRMS (ES+) 223.1, 279.2 ([M-O^{t}Bu]$^+$, 100, [M+H]$^+$, 70);

HRMS: Found (ES+): [M+H]$^+$ 279.1704 $C_{15}H_{23}N_2O_3$, requires 279.1709.

(*S*)-2-Amino-*N*-benzyl-3-methoxypropanamide (1z)

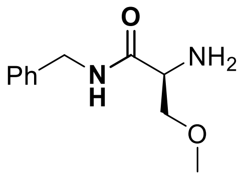

Prepared according to method B from OMe-Serine (595 mg, 5 mmol) and benzylamine (0.818 mL, 7.5 mmol) with 10 mol% $B(OCH_2CF_3)_3$ for 24 h to yield a colourless oil (870 mg, 84%, 75:25 er determined using Marfeys reagent);

Prepared according to method B, from OMe-Serine (595 mg, 5 mmol) and benzylamine (0.818 mL, 7.5 mmol) with 20 mol% $B(OCH_2CF_3)_3$ for 18 h to yield a colourless oil (946 mg, 91%, 74:26 er determined using Marfeys reagent);

δ_H (600 MHz, $CDCl_3$) 7.83 (br s, 1H, NH), 7.26-7.23 (m, 2H, ArH), 7.20-7.17 (m, 2H, ArH), 4.38 (dd, *J* = 15.0, 6.2 Hz, 1H, C*H*H), 4.34 (dd, *J* = 15.0, 6.0 Hz, 1H, C*H*HPh), 3.54 (dd, *J* = 9.2, 6.2 Hz, 1H, C*H*HPh), 3.52 (dd, *J* = 9.3, 4.1 Hz, 1H, C*H*H), 3.49-3.47 (m, 1H, C*H*), 3.28 (s, 3H, CH_3), 1.7 (br s, 2H, NH_2);

δ_C (150 MHz, $CDCl_3$) 172.9, 138.5, 128.7, 127.6, 127.4, 74.7, 58.9, 55.0, 43.1;

LRMS (ES+) 209 ([M+H]$^+$, 100);

Data in accordance with the literature.[12]

N-Benzylthiazolidine-4-carboxamide and 5-amino-3-benzyl-1,3-thiazinan-4-one (1aa)

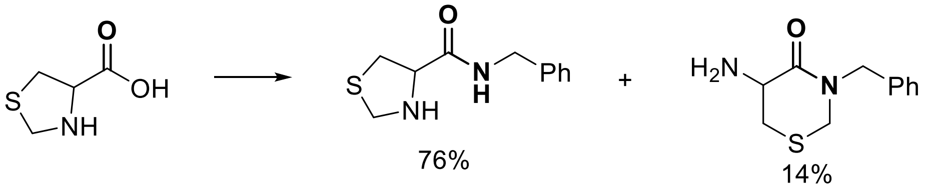

Prepared according to method B from Thioproline (665 mg, 5 mmol) and benzylamine (0.818 mL, 7.5 mmol) for 24 h and benzylamine removed *in vacuo* (10 mbar, 80 °C) to yield a mixture of products which were separated out by column chromatography (95:5, EtOAc:MeOH).

N-benzylthiazolidine-4-carboxamide (1aa)

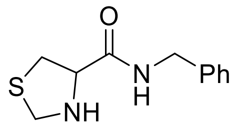

Yellow solid (840 mg, 76%);

mp 82-83 °C [lit. 80-82 °C];[13]

ν_{max} (solid/cm^{-1}) 3285, 3031, 1642, 1553, 1497;

δ_H (400 MHz, $CDCl_3$) 7.39 (br s, 1H, N*H*), 7.36-7.32 (m, 2H, Ar*H*), 7.30-7.25 (m, 3H, Ar*H*), 4.43 (m, 2H, CH_2Bn), 4.21 (d, *J* = 10.0 Hz, 1H, SC*H*HN), 4.16 (dd, *J* = 7.6, 4.3 Hz, 1H, C*H*), 3.94 (d, *J* = 10.0 Hz, 1H, SC*H*HN), 3.48 (dd, *J* = 10.8, 4.3 Hz, C*H*HCH), 3.13 (dd, *J* = 10.8, 7.6 Hz, 1H, C*H*HCH), 2.42 (br s, 1H, N*H*);

δ_C (150 MHz, $CDCl_3$) 170.7, 138.1, 128.8, 127.7, 127.6, 66.1, 53.6, 43.4, 35.4;

LRMS (ES+) 223.2 ($[M+H]^+$, 100);

HRMS: Found (ES+): $[M+H]^+$ 223.0911 $C_{11}H_{14}N_2OS$, requires 223.0905;

Data in accordance with the literature.[13]

5-Amino-3-benzyl-1,3-thiazinan-4-one (SI.1ab)

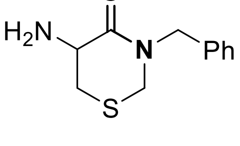

Pale yellow oil (154 mg, 14%);

ν_{max} (film/cm^{-1}) 3292, 2924, 1639, 1451;

δ_H (400 MHz, $CDCl_3$) 7.36-7.24 (m, 5H, Ar*H*), 4.86 (d, *J* = 14.9 Hz, 1H, CH*H*N), 4.53 (d, *J* = 14.9 Hz, 1H, CH*H*N), 4.43 (d, *J* = 13.2 Hz, 1H, CH*H*N), 4.04 (d, *J* = 13.2 Hz, 1H, CH*H*N), 3.77 (dd, *J* = 10.5, 6.1 Hz, 1H, C*H*HCH), 3.23 (dd, *J* = 11.7, 6.1 Hz, 1H, C*H*HCH), 2.74 (app t, *J* = 11 Hz, C*H*), 2.00 (br s, 1H, N*H*);

δ_C (100 MHz, $CDCl_3$) 172.8, 128.8, 128.0, 127.8, 52.3, 50.8, 46.0, 32.5;

LRMS (ES+) 223.2 ($[M+H]^+$, 100);

HRMS: Found (ES+): $[M+H]^+$ 223.0897 $C_{11}H_{14}N_2OS$, requires 223.0905.

(*S*)-2-Amino-*N*-benzyl-3-(methylthio)propanamide (1bb)

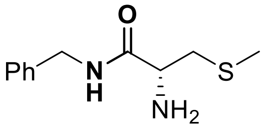

This compound was prepared according to method B from SMe-Cysteine (675 mg, 5 mmol) and benzylamine (0.818 mL, 7.5 mmol) and purified by column chromatography (CH_2Cl_2:MeOH, 95:5) to yield a pale yellow oil (969 mg, 87%, er 67:33);

$[\alpha]_D$ −10.5 (*c* 1.0, CH_3Cl, 25 °C);

ν_{max} (film/cm^{-1}) 3345, 1657, 1525, 1231;

δ_H (400 MHz, $CDCl_3$) 7.78 (br s, 1H, N*H*), 7.35-7.25 (m, 5H, Ar*H*), 4.45 (d, *J* = 6.0 Hz, 2H, CH_2Ph), 3.56 (dd, *J* = 8.7, 3.8 Hz, 1H, CH*H*), 3.05 (dd, *J* = 13.7, 3.8 Hz, 1H, CH*H*), 2.71 (dd, *J* = 13.7, 8.7 Hz, 1H), 2.10 (s, 3H, CH_3), 1.73 (br s, 2H, NH_2);

δ_C (100 MHz, $CDCl_3$) 173.6, 138.4, 128.8, 127.8, 127.6, 53.6, 43.3, 39.8, 15.5;

LRMS (ES+): 225.1 ($[M+H]^+$, 100);

HRMS (ES+): Found (ES+): [M+H]+ 225.1071, $[C_{11}H_{17}N_2OS+H]^+$, requires 225.1062.

***N*-Benzyl-2-(methylamino)acetamide (1cc)**

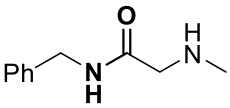

Prepared according to General Procedure B, from Sarcosine (0.445 g, 5 mmol) and benzylamine (0.818 mL, 7.5 mmol) and further purified by flash column chromatography (94:6 CH_2Cl_2:MeOH) to yield a yellow oil (730 mg, 82%);

δ_H (600 MHz, $CDCl_3$) 7.53 (s, 1H, $PhCH_2NH$), 7.38-7.31 (m, 2H, Ph), 7.31-7.27 (m, 3H, Ph), 4.49 (d, *J* = 6.0 Hz, 2H, $PhCH_2$), 3.30 (s, 2H, $COCH_2$), 2.42 (s, 3H, CH_3);

δ_C (150 MHz, $CDCl_3$) 171.5, 138.5, 128.8, 127.8, 127.5, 54.7, 43.1, 37.0;

LRMS (ES+): 179 ($[M+H]^+$, 100);

Data in accordance with the literature.[14]

(*S*)-2-Amino-N-benzyl-2-phenylacetamide (1ee)

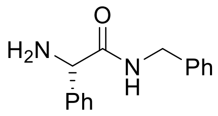

Prepared according to General Procedure B for 24 h from Phenylglycine (0.755 g, 5 mmol) and benzylamine (0.818 mL, 7.5 mmol) and further purified by flash column chromatography to yield a colourless oil (1124 mg, 93%, er 50:50, determined using Marfey's reagent);

ν_{max} (film/cm^{-1}) 3289, 2888, 1665, 1546;

δ_H (600 MHz, $CDCl_3$) 7.47-7.21 (m, 10H, Ar*H*), 4.59 (s, 1H, C*H*), 4.51-4.42 (m, 2H, $PhCH_2$);

δ_C (150 MHz, $CDCl_3$) 173.0, 141.1, 138.4, 129.0, 128.8, 128.2, 127.8, 127.6, 127.0, 60.1, 43.4;

Data in accordance with literature.[15]

Substrates from Figure 3

The synthesis of compounds **6a-f, 6k, 6q, 6r, 6v, 6w, 6x, 6z and 6aa** is described in our previous reports.[7,16] Where these compounds have been synthesised using a different method (catalytic borate or $Ti(OiPr)_4$, we include the spectra for enantiopurity.

(*S*)-2-Amino-*N*-(cyclohexylmethyl)-4-(methylthio)butanamide (6g)

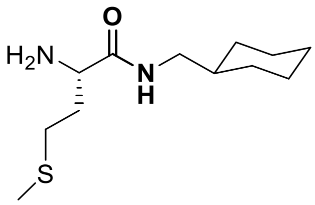

Prepared according to General Procedure B from Methionine (0.745 g, 5 mmol) and benzylamine (0.818 mL, 7.5 mmol) for 24 h and further purified by flash column chromatography to yield a white pasty solid (1.14 g, 94%, >95:5 er determined using Marfey's reagent); Note: 4% of di-amino amide was inseparable from the amino amide.

$[\alpha]_D^{20}$ −16.3 (*c* 1.0, MeOH);

ν_{max} (film/cm^{-1}) 3296, 2918, 1639, 1525;

δ_H (600 MHz, $CDCl_3$) 7.34 (br s, 1H, N*H*), 3.38-3.40 (m, 1H, C*H*CON), 2.98-3.00 (m, 2H, CH_2), 2.50-2.52 (m, 2H, CH_2), 2.07-2.04 (m, 1H, CH*H*), 2.01 (s, 3H, SCH_3) 1.68-1.66 (m, 1H, CH*H*), 1.61-1.63 (m, 4H, 2xCH_2), 1.58-1.56 (br d, 1H, C*H*H), 1.46 (br s, 2H, NH_2), 1.36-1.37 (m, 1H, C*H*), 1.14-1.10 (m, 2H, CH_2), 1.09-1.05 (br d, 1H, C*H*H), 0.85 (m, 2H, CH_2);

δ_C (150 MHz, $CDCl_3$) 174.6, 54.1, 45.4, 38.0, 34.3, 30.9, 30.8, 26.5, 25.9, 15.4;

HRMS: Found (ES+): $[M+H]^+$ 245.1688, $C_{12}H_{25}N_2OS$, requires 245.1688.

(*S*)-2-Amino-*N*-cyclohexyl-3-phenylpropanamide (6h)

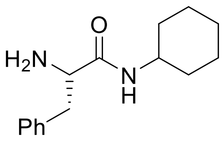

Prepared according to General Procedure A, at 80 °C for 15 h and further purified by flash column chromatography (2% MeOH:CH_2Cl_2) to yield a brown solid (147 mg, 56%, er 55:45, determined using Marfey's reagent);

mp 74-76 °C [lit.[17] 78-79 °C for DL-**6h**];

$[\alpha]_D^{25}$ −15.1 (*c* 0.20, $CHCl_3$, 25 °C);

δ_H (600 MHz, $CDCl_3$) 7.33-7.18 (m, 5H, Ph), 7.08 (br s, 1H, NH), 3.75 (m, 1H, CONHC*H*), 3.56 (dd, *J* = 9.1, 4.3 Hz, 1H, COC*H*), 3.23 (dd, *J* = 13.7, 4.3 Hz, 1H, PhCH*H*), 2.69 (dd, *J* = 13.7, 9.1 Hz, 1H, PhC*H*H), 1.89-1.79 (m, 2H, 2 × CyH), 1.73-1.64 (m, 2H, 2 × CyH), 1.62-1.55 (m, 1H, CyH), 1.42-1.33 (m, 3H, NH_2 and CyH), 1.20-1.07 (m, 3H, 3 × CyH);

δ_C (150 MHz, $CDCl_3$) 173.3, 138.1, 129.5, 128.8, 126.9, 56.5, 47.7, 41.2, 33.2, 33.1, 25.7, 24.9;

LRMS (ES+): 247 ($[M+H]^+$, 100).

(*S*)-2-Amino-*N*-(4-methoxybenzyl)-3-phenylpropanamide (6i)

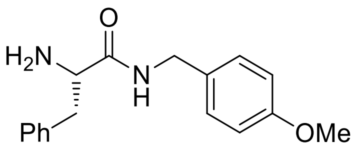

Prepared according to method A, at 80 °C for 15 h from phenylalanine (83 mg, 0.5 mmol) and 4-methoxybenzylamine (0.195 mL, 1.5 mmol) and further purified by trituration with Et_2O to yield a white solid (122 mg, 86%, er 90:10, determined using chiral HPLC, Chiralcel OD-1 n-Hex/PrOH; 9/1, 0.5 ml/min; 254 nm; R_t(S) = 27.90 min)

mp 78-80 °C;

$[\alpha]_D^{25}$ +3.9 (*c* 1.0, MeOH);

ν_{max} (solid/cm^{-1}) 3295, 2890, 1670;

δ_H (600 MHz, $CDCl_3$) 7.52 (br s, 1H, NH), 7.28-7.32 (m, 2H, ArH), 7.20-7.26 (m, 3H, ArH), 7.14-7.18 (m, 2H, ArH), 6.83-6.86 (m, 2H, ArH), 4.33-4.41 (m, 2H, CH_2NH), 3.79 (s, 3H, CH_3), 3.63 (dd, *J* = 9.2, 4.1 Hz, 1H, C*H*NH_2), 3.29 (dd, *J* = 13.7, 4.1 Hz, 1H, CH*H*Ph), 2.73 (dd *J* 9.2, 13.7 Hz, 1H, C*H*HPh), 1.42, (br s, 2H, NH_2);

δ_C (150 MHz, $CDCl_3$) 174.1, 159.0, 138.0, 130.6, 129.5, 129.2, 128.8, 126.9, 114.1, 56.6, 55.4, 42.7, 41.1;

Found (CI): $[M+H]^+$ 285.160994 $C_{17}H_{21}N_2O_2$, requires 285.16030.

(*S*)-2-Amino-*N*-(4-fluorobenzyl)-3-phenylpropanamide (6j)

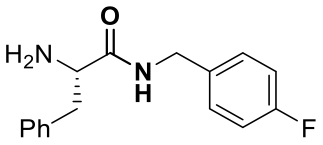

Prepared according to General Procedure A, at 80 °C for 15 h from phenylalanine (83 mg, 0.5 mmol) and 4-Fluorobenzylamine (0.171 mL, 1.5 mmol) and further purified by flash column chromatography (Petrol:EtOAc:Et_3N, 100:25:1) to yield a yellow oil (172 mg, 63 %, er 85:15, determined using Marfey's reagent);

$[\alpha]_D^{20}$ +8.0 (*c* 0.40, MeOH);

Prepared according to General Procedure B for 24 h from phenylalanine (825 mg, 5.0 mmol) and 4-F benzylamine (0.856 mL, 7.5 mmol) and further purified by flash column chromatography (Petrol:EtOAc:Et_3N, 100:25:1) to yield a yellow oil (807 mg, 59 %, er 89:11, determined using Marfey's reagent);

$[\alpha]_D^{20}$ −48.4 (*c* 1.0, $CHCl_3$);

ν_{max} (film/cm^{-1}) 3285, 3028, 2925, 1648, 1507;

δ_H (600 MHz, $CDCl_3$) 7.63 (br s, 1H, NH), 7.30-7.17 (m, 9H, Ar), 4.41 (dd, *J* = 14.8, 6.0 Hz, 1H, ArC*H*H), 4.38 (dd, *J* = 14.8, 6.0 Hz, 1H, ArCH*H*), 3.66 (m, 1H), 3.27 (dd, *J* = 13.7, 4.2 Hz, 1H, PhC*H*H), 2.76 (dd, *J* = 13.7, 9.0 Hz, 1H, PhCH*H*);

δ_C (150 MHz, $CDCl_3$) 174.2, 162.2 (d, J_{CF} = 245.5 Hz, Ar*C*F), 137.8, 134.3 (d, J_{CF} = 3.2 Hz, ArC), 129.5, 129.5, 128.9, 127.0, 115.6 (d, J_{CF} = 21.4 Hz, 2 × ArCH), 56.5, 42.5, 41.1;

LRMS (ES+): 273 ($[M+H]^+$, 100);

HRMS: Found (ES+): $[M+H]^+$ 273.1403, $C_{16}H_{18}N_2OF$, requires 273.1403.

(*S*)-*N*-(4-Methoxybenzyl)pyrrolidine-2-carboxamide (6l)

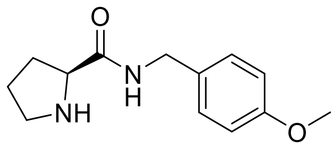

Prepared according to General Procedure B, from proline (575 mg, 5.0 mmol) and 4-methoxybenzylamine (0.980 mL, 7.5 mmol) and further purified by flash column

chromatography to yield a light brown oil (980 mg, 84%, er >95:5, determined using Marfey's reagent);

$[\alpha]_D^{20}$ −48.4 (*c* 1.0, $CHCl_3$);

ν_{max} (solid/cm^{-1}) 3303, 2965, 2869, 2832, 1648, 1610, 1508;

δ_H (600 MHz, $CDCl_3$) 7.9 (br s, 1H, CON*H*), 7.12 (d, *J* = 8.7 Hz, 2H, Ar*H*), 6.79 (d, *J* = 8.7 Hz, 2H, Ar*H*), 4.29 (d, *J* = 5.9 Hz, 2H, CH_2Ar), 3.71 (s, 3H, CH_3), 3.68 (dd, *J* = 9.0, 5.3, 1H, C*H*), 2.93-2.88 (m, 1H, CHNHC*H*H), 2.80-2.77 (m, 1H, CHNHCH*H*), 2.16 (br s, 1H, N*H*), 2.10-2.04 (m, 1H, CHC*H*H), 1.89-1.84 (m, 1H, 1 × CHC*H*H), 1.68-1.58 (m, 2H, $NHCH_2CH_2$);

δ_C (150 MHz, 100 mHz) 175.1, 158.9, 130.9, 128.9, 114.0, 60.7, 55.3, 47.3, 42.4, 30.9, 26.3;

LRMS (ES+): 235.1 ($[M+H]^+$, 100);

HRMS (ES+): Found (ES+): [M+H]+ 235.1450, ($[C_{13}H_{18}N_2O_2+H]^+$), requires 235.1447.

(*S*)-*N*-(2-(1*H*-Indol-3-yl)ethyl)-2-aminopropanamide (6m)

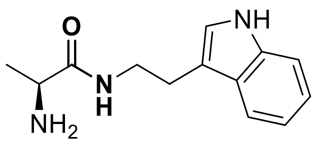

Prepared according to General Procedure B, from tryptamine (1.6 g, 10 mmol, 2 eq) and alanine (445 mg, 5 mmol) and further purified by flash column chromatography to yield a light brown solid (704 mg, 61%, er 85:15, determined using Marfey's reagent);

δ_H (600 MHz, $CDCl_3$) 8.6 (br s, 1H, N*H*), 7.61 (d, *J* = 7.9 Hz, 1H, Ar*H*), 7.37 (d, *J* = 8.2 Hz, 1H, Ar*H*), 7.34 (br s, 1H, N*H*), 7.19 (t, *J* = 7.6 Hz, 1H, Ar*H*), 7.12 (t, *J* = 7.8 Hz, 1H, Ar*H*), 7.00 (s, 1H, Ar*H*), 3.60 (m, 2H, CH_2), 3.43 (q, *J* = 7.0 Hz, 1H, C*H*), 2.98 (t, *J* = 6.9 Hz, 2H, CH_2), 1.46 (br s, 2H, NH_2), 1.29 (d, *J* = 7.0 Hz, 3H, CH_3);

δ_C (150 MHz, $CDCl_3$) 175.9, 136.5, 127.5, 122.2, 122.1, 119.4, 118.8, 113.0, 111.5, 50.9, 39.6, 25.5, 21.9;

LRMS (ES+) 232.1 ($[M+H]^+$, 100);

Data in accordance with the literature.[18]

(*S*)-2-Amino-*N*-(4-(3,3,4,4,5,5,6,6,7,7,8,8,9,9,10,10,10-heptadecafluorodecyl)benzyl)-3-phenylpropanamide (6n)

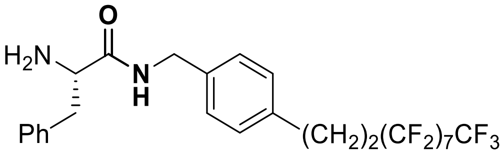

Prepared according to General Procedure B, from 4-perfluorodecyl benzylamine (2.07 g, 3.75 mmol) and phenylalanine (413 mg, 2.5 mmol) and further purified by flash column chromatography. The white solid was recrystallised from propanol to give a white solid (1.27g, 72%, er >95:5, determined using Marfey's reagent);

$[\alpha]_D^{25}$ – 24.5 (*c* 1.0, CH_3Cl, 25 °C);

ν_{max} (film/cm^{-1}) 3308, 1632, 1539, 1194;

δ_H (400 MHz, $CDCl_3$) 7.6 (br s, 1H, N*H*), 7.33-7.15 (m, 9H, Ar*H*), 4.45 (dd, *J* = 14.7, 5.9 Hz, 1H, NC*H*H), 4.38 (dd, *J* = 14.7, 5.6 Hz, 1H, NC*H*H), 3.66 (dd, *J* = 9.3, 4.2 Hz, 1H, C*H*), 3.30 (dd, *J* = 13.7, 3.9 Hz, 1H, CH*H*), 2.92-2.88 (m, 2H, R_FCH_2), 2.76 (dd, *J* = 13.7, 9.3 Hz, 1H, CH*H*), 2.42-2.29 (m, 2H, R_FCH_2), 1.41 (br s, 2H, NH_2);

δ_C (100 MHz, $CDCl_3$, not all carbons visible due to coupling with ^{19}F) 174.1, 138.3, 137.9, 136.9, 129.3, 128.7, 128.6, 128.2, 126.8, 56.5, 42.8, 41.0, 32.9 (t, *J* = 22.2 Hz), 26.1 (br t, *J* = 4.0 Hz);

δ_F (377 MHz, $CDCl_3$) −80.75 (t, *J* = 10.3 Hz), −114.6 (m), −121.96 (m), −121.9 (m), −122.7 (m), −123.4 (m), −126.1 (m);

LRMS (ES+): 701.12 ($[M+H]^+$, 100);

***(S)*-2-Amino-3-phenyl-*N*-(pyridin-2-ylmethyl)propanamide (6o)**

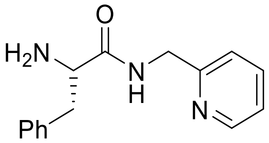

Prepared according to General Procedure A, at 125 °C for 15 h and further purified by flash column chromatography to yield a light brown solid (112 mg, 44%, er 60:40, determined using Marfey's reagent);

$[\alpha]_D^{25}$ −6.1 (*c* 1.0, MeOH);

Prepared according to General Procedure B from phenylalanine (825 mg, 5.0 mmol) and picolylamine (0.772 mL, 7.5 mmol) using 30 mol% $B(OCH_2CF_3)_3$, and further purified by flash column chromatography to yield a light brown solid (876 mg, 86%, er 92:8, determined using Marfey's reagent);

mp 109-110 °C;

$[\alpha]_D^{20}$ -41.7 (*c* 1.0, $CHCl_3$);

ν_{max} (solid/cm^{-1}) 3351 (N-H), 2872, 1640 (C=O);

δ_H (600 MHz, $CDCl_3$) 8.53 (d, *J* = 4.4 Hz, 1H, Ar), 8.17 (br s, 1H, NH), 7.64 (td, *J* = 7.7, 1.6 Hz, 1H, Ar), 7.27-7.31 (m, 2H, Ar), 7.20-7.25 (m, 4H, Ar), 7.18 (dd, *J* = 7.0, 5.2 Hz, 1H, Ar), 4.57 (d, *J* = 5.3 Hz, 2H, CONHC*H*$_2$), 3.68 (dd, *J* = 9.5, 4.0 Hz, 1H, C*H*CO), 3.30 (dd, *J* = 13.8, 4.0 Hz, 1H, PhCH*H*), 2.73 (dd, *J* = 13.8, 9.5 Hz, 1H, PhC*H*H), 1.63 (br s, 2H, NH_2);

δ_C (150 MHz, $CDCl_3$) 174.5, 157.0, 149.3, 138.1, 136.9, 129.4, 128.8, 126.9, 122.4, 122.1, 56.8, 44.5, 41.2;

HRMS: Found (CI): $[M+H]^+$ 256.144138 $C_{15}H_{18}N_3O$, requires 256.14499.

(*S*)-2-Amino-*N*-(4-methoxyphenyl)-3-phenylpropanamide (6p)

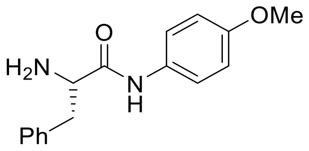

Prepared according to General Procedure A, at 125 °C for 24 h and further purified by flash column chromatography to yield a brown solid (95 mg, 35%, er 1:1, determined using Marfey's reagent);

mp 88-90 °C; [lit.[19] 89-90 °C for DL-**6p**)

ν_{max} (solid/cm^{-1}) 3381, 3253, 1642, 1536;

δ_H (600 MHz, $CDCl_3$) 9.29 (s, 1H, NH), 7.58-7.43 (m, 2H, 2 × ArH), 7.37-7.29 (m, 2H, Ph), 7.26-7.18 (m, 3H, Ph), 6.93-6.77 (m, 2H, 2 × ArH), 3.77 (s, 3H, CH_3), 3.69 (dd, *J* = 9.4, 4.0 Hz, 1H, CH), 3.34 (dd, *J* = 13.8, 4.0 Hz, 1H, CHC*H*H), 2.77 (dd, *J* = 13.8, 9.4 Hz, 1H, CHCH*H*);

δ_C (150 MHz, $CDCl_3$) 172.3, 156.4, 138.0, 131.1, 129.5, 128.9, 127.3, 121.3, 114.2, 56.9, 55.6, 40.9;

LRMS (ES+): 271 ($[M+H]^+$, 100).

Data in accordance with the literature.[20]

(*S*)-2-Amino-4-methyl-1-(pyrrolidin-1-yl)pentan-1-one (6s)

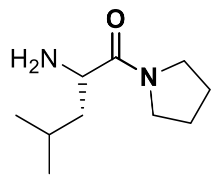

Prepared according to General Procedure A [dropwise], at 80 °C for 15 h and further purified by flash column chromatography (2% MeOH:CH_2Cl_2) to yield a brown oil (93 mg, 50%, er 60:40, determined using Marfey's reagent);

$[\alpha]_D^{20}$ −3.8 (*c* 1.0, $CHCl_3$);

ν_{max} (film/cm^{-1}) 3400, 3205, 1648, 1442

δ_H (600 MHz, $CDCl_3$) 3.56-3.49 (m, 3H, 2 × NCH*H*, C*H*NH_2), 3.45-3.36 (m, 2H, 2 × NCH*H*), 1.99-1.91 (m, 2H, 2 × NCH_2CH*H*), 1.88-1.82 (m, 4H, CH_3C*H*, 2 × NCH_2CH*H*, NH_2), 1.43-1.32 (m, 2H, CHCH_2), 0.90 (d, *J* = 2.4 Hz, 3H, CH_3), 0.88 (d, *J* = 2.2 Hz, 3H, CH_3);

δ_C (150 MHz, $CDCl_3$) 174.6, 51.5, 46.1, 44.5, 26.3, 24.8, 24.2, 23.8, 21.7;

LRMS (ES+): 245.1 ([M+H]$^+$, 100);

Data in accordance with the literature[21]

***(S)*-2-Amino-3-phenyl-1-(pyrrolidin-1-yl)propan-1-one (6u)**

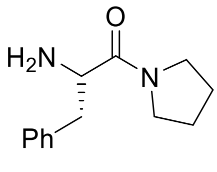

Prepared according to General Procedure A, at 125 °C for 15 h and further purified by flash column chromatography (2% MeOH:CH_2Cl_2) to yield a brown oil (105 mg, 48%, er 53:47, determined using Marfey's reagent);

$[\alpha]_D^{25}$ +6.1 (*c* 0.5, MeOH);

ν_{max} (film/cm^{-1}) 3033, 2969, 2884, 1673, 1586;

δ_H (600 MHz, $CDCl_3$) 7.28-7.26 (m, 2H, Ar), 7.22-7.18 (m, 3H, Ar), 3.71 (app t, *J* = 7.2 Hz, 1H, CH), 3.45 (dd, *J* = 13.0, 7.0 Hz, 1H, CHCH*H*), 3.39-3.30 (m, 2H, NCH_2), 2.93 (dd, *J* =

13.1, 7.4 Hz, 1H, CHC*H*H), 2.80-2.75 (m, 2H, NCH_2), 1.80-1.72 (m, 2H, NCH_2CH_2), 1.72-1.59 (m, 1H, NCH_2CH_2);

δ_C (150 MHz, $CDCl_3$) 173.1, 137.9, 129.4, 128.6, 126.8, 55.2, 46.1, 45.9, 43.0, 26.0, 24.1;

LRMS (ES+): 219 ($[M+H]^+$, 100);

HRMS: Found (ES+): $[M+H]^+$ 219.1491 $C_{13}H_{18}N_2O$, requires 219.1492.

tert-Butyl L-phenylalanylglycinate (6y)

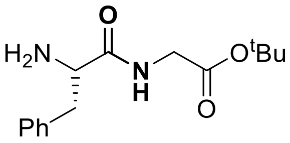

Prepared according to General Procedure A, at 125 °C for 15 h and further purified by flash column chromatography (2% MeOH:CH_2Cl_2) to yield a colourless oil (157 mg, 56%, er 2:1, determined using Marfey's reagent);

ν_{max} (film/cm^{-1}) 3303, 3026, 2974, 1736, 1667, 1517;

δ_H (600 MHz, $CDCl_3$) 7.77 (br s, 1H, NH), 7.32-7.27 (m, 2H, Ar), 7.24-7.18 (m, 3H, Ar), 3.96 (dd, *J* = 18.2, 5.4 Hz, 1H, C*H*H), 3.91 (dd, *J* = 18.2, 5.4 Hz, 1H, CH*H*), 3.62 (dd, *J* = 9.9, 3.9 Hz, 1H, CH), 3.29 (dd, *J* = 13.8, 3.9 Hz, 1H, CHCH*H*), 2.65 (dd, *J* = 13.8, 9.9 Hz, 1H, CHCH*H*), 1.56 (s, 2H, NH_2), 1.46 (s, 9H, 3 × CH_3);

δ_C (150 MHz, $CDCl_3$) 174.7, 169.2, 138.1, 129.4, 128.8, 126.9, 82.2, 56.6, 41.8, 41.0, 28.2;

LRMS (ES+): 279 ($[M+H]^+$, 85), 223 ($[M\text{-}^tBu+2H]^+$, 100);

HRMS: Found (ES+): $[M+H]^+$ 279.1702 $C_{15}H_{23}N_2O_3$, requires 279.1703.

(*S*)-3-Benzyl-5-phenyl-1,3-dihydro-2H-benzo[e][1,4]diazepin-2-one (8)

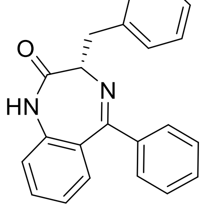

A mixture of 2-aminobenzophenone (197 mg, 1 mmol, 1 equiv.), L-Phe (165 mg, 1 equiv., 1 mmol) and $B(OCH_2CF_3)_3$ (0.64 mL, 3 mmol, 3 equiv.) in CPME was stirred at 125 °C for 4

days and further purified by flash column chromatography (Petrol:EtOAc 70:30) to give a white solid (170 mg, 52%, er 54:46, determined used chiral HPLC);

mp 189-190 °C;

$[\alpha]_D^{20}$ +13.8 (*c* 1.0, $CHCl_3$);

ν_{max} (solid/cm^{-1}) 3196, 2964, 1676 (C=O);

δ_H (600 MHz, $CDCl_3$) 9.36 (2 × br s, 1H, NH), 7.52-7.47 (m, 3H, ArH), 7.43-7.41 (m, 3H, ArH), 7.38-7.34 (m, 2H, ArH), 7.34-7.27 (m, 3H, ArH), 7.23 (t, *J* = 6.8 Hz, 1H, ArH), 7.19-7.17 (m, 1H, ArH), 7.13 (t, *J* = 7.6 Hz, 1H, ArH), 3.82 (t, *J* = 6.7 Hz, 1H, CH), 3.68-3.58 (m, 2H, CH_2);

δ_C (150 MHz, $CDCl_3$) 171.9, 169.4, 139.5, 139.4, 138.5, 131.8, 131.4, 130.4, 130.1, 130.0, 128.3, 128.3, 127.7, 126.3, 123.4, 121.3, 65.0, 37.8;

LRMS (ES+): 327 ($[M+H]^+$, 100);

HRMS: Found (ES+): $[M+H]^+$ 327.1498 $C_{22}H_{19}N_2O$, requires 327.1497.

Data in accordance with the literature.[22]

5. Trisamino borane amidations

General procedure C:

All reactions were performed on 0.5 or 1.0 mmol scale. An unprotected amino acid (1 eq) and trisaminoborane (1.0-1.5 eq) were stirred at 80 °C in CPME (0.5 M, unless stated otherwise) for 1-6 h. Upon completion, the mixture was diluted with EtOAc or CH_2Cl_2 (3 mL) and water (0.5 mL). Amberlite IRA-743 and Amberlyst A-26(OH) were added and stirred for 30 min. The mixture was dried over $MgSO_4$ and then filtered. The solids were washed with EtOAc (3 × 20 mL) and the product concentrated *in vacuo* to give the amino amide. Where necessary, amides were further purified by column chromatography.

General procedure C [room temp]:

All reactions were performed on 0.5 mmol scale. An unprotected amino acid (1 eq) and trisaminoborane (1.5 eq) were stirred at 21 °C in MeCN (0.5 M) for 12 h. Upon completion, the mixture was diluted with EtOAc or CH_2Cl_2 (3 mL) and water (0.5 mL). Amberlite IRA-743 and Amberlyst A-26(OH) were added and stirred for 30 min. The mixture was dried over $MgSO_4$ and then filtered. The solids were washed with EtOAc (3 × 20 mL) and the product concentrated *in vacuo* to give the amino amide. Where necessary, amides were further purified by column chromatography.

(S)-2-Amino-3-phenyl-1-(pyrrolidin-1-yl)propan-1-one (6u)

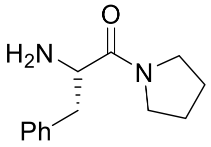

Prepared according to General Procedure C, at 80 °C for 6 h and further purified by flash column chromatography (2% MeOH:CH_2Cl_2) to yield a colourless oil (79 mg, 72%, er 95:5 determined using Marfey's reagent);

$[\alpha]_D^{20}$ +15.7 (*c* 1.0, $CHCl_3$);

Vide supra for spectroscopic data

(*S*)-2-Amino-4-methyl-1-(pyrrolidin-1-yl)pentan-1-one (6s)

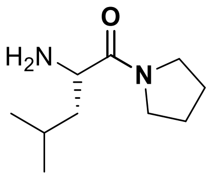

Prepared according to General Procedure C with 1.5 eq of trispyrrolidinoborane, at 80 °C for 5 h and further purified by flash column chromatography (2% MeOH:CH_2Cl_2) to yield a colourless oil (75 mg, 41%, er 93:7 determined using Marfey's reagent);

$[\alpha]_D^{20}$ −7.3 (*c* 1.0, $CHCl_3$);

Vide supra for spectroscopic data

(*R*)-3-Amino-3-(2-chlorophenyl)-*N,N*-dimethylpropanamide (6cc)

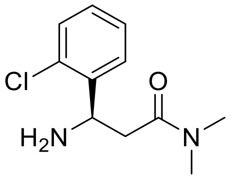

Prepared according to General Procedure C, at 80 °C for 7 h and further purified by flash column chromatography (2% MeOH:CH_2Cl_2) to yield a colourless oil (31 mg, 14%, er could not be determined);

δ_H (400 MHz, $CDCl_3$) 7.68 (dd, *J* = 7.7, 1.5 Hz, 1H, Ar*H*), 7.35 (dd, *J* = 7.9, 1.2 Hz, 1H, Ar*H*), 7.29 (td, *J* = 7.6, 1.1 Hz, 1H, Ar*H*), 7.20 (td, *J* = 7.7, 1.6 Hz, 1H, Ar*H*), 4.92 (dd, *J* = 9.7, 2.5

Hz, 1H, C*H*), 2.96 (s, 3H, CH_3), 2.94 (s, 3H, CH_3), 2.73 (dd, *J* = 16.2, 2.6 Hz, 1H, C*H*H), 2.53 (dd, *J* = 16.2, 9.7 Hz, 1H, C*H*H), 1.9 (br s, 2H, NH_2)

δ_C (100 MHz, $CDCl_3$) 171.3, 142.4, 132.6, 129.7, 128.3, 127.7, 127.3, 49.1, 41.2, 37.2, 35.5

HRMS: Found (ES+): $[M+H]^+$ 226.0868 $C_{11}H_{15}N_2OCl$, requires 226.0867.

(*S*)-2-Amino-*N,N*-4-trimethylpentanamide (6dd)

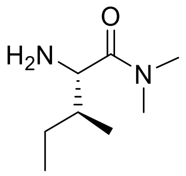

Prepared according to General Procedure C on a 5 mmol scale, at 80 °C for 5 h and further purified by flash column chromatography (3% MeOH:CH_2Cl_2) to yield a colourless oil (159 mg, 25%, dr 95:5);

$[\alpha]_D^{25}$ +36.1 (*c* 1.0, CH_3Cl, 25 °C);

ν_{max} (film/cm^{-1}) 3016, 1625, 1373;

δ_H (400 MHz, $CDCl_3$) 3.50 (d, *J* = 5.8 Hz, 1H, C*H*NH_2), 3.02 (s, 3H, NCH_3), 2.95 (s, 3H, NCH_3), 1.79 (br s, 2H, NH_2), 1.57-1.54 (m, 2H, CH_2), 1.13-1.0.7 (m, 1H, C*H*CH_2), 0.90 (d, *J* = 6.9 Hz, 3H, CHCH_3), 0.86 (t, *J* = 7.4 Hz, 3H, CH_2CH_3);

δ_C (100 MHz, $CDCl_3$) 175.5, 55.8, 39.1, 37.3, 35.9, 23.7, 16.3, 11.6;

LRMS (ES+) 159.1 ($[M+H]^+$, 100), 317.3 ($[2M+H]^+$, 70);

HRMS: Found (ES+): $[M+H]^+$ 159.1490, $C_8H_{19}N_2O$, requires 159.1497.

(*S*)-2-Amino-*N,N*-dimethyl-3-phenylpropanamide (6ee)

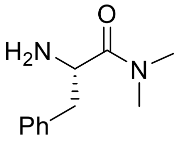

Prepared according to General Procedure C, at 80 °C for 5 h and further purified by flash column chromatography (2% MeOH:CH_2Cl_2) to yield a colourless oil (56 mg, 29%, er 95:5 determined by Marfeys reagent);

Prepared according to General Procedure C, at 80 °C for 5 h and further purified by flash column chromatography (2% MeOH:CH_2Cl_2) to yield a colourless oil (98 mg, 56%, er 92:8 determined by Marfeys reagent);

$[\alpha]_D$ +43.6 (*c* 1.0, CH_3Cl, 25 °C);

ν_{max} (film/cm^{-1}) 3359, 2926, 1624, 1494;

δ_H (400 MHz, $CDCl_3$) 7.29-7.26 (m, 2H, Ar*H*), 7.22 (t, J = 7.3 Hz, 1H, Ar*H*), 7.18-7.17 (m, 2H, Ar*H*), 3.92 (t, *J* = 7.1 Hz, 1H, C*H*), 2.92 (dd, *J* = 13.4, 6.8 Hz, 1H, C*H*H), 2.89 (s, 3H, CH_3), 2.75 (dd, *J* = 13.4, 7.5 Hz, 1H, C*H*H), 2.72 (s, 3H, CH_3), 1.9 (br s, 2H);

δ_C (100 MHz, $CDCl_3$) 174.8, 137.9, 129.4, 128.6, 126.9, 52.9, 43.0, 36.7, 35.8;

LRMS (ES+) 193.1 ($[M+H]^+$, 100)

HRMS: Found (ES+): $[M+H]^+$ 193.1335, $C_{11}H_{17}N_2O$, requires 193.1341

(*S*)-2-Amino-3-(1*H*-indol-3-yl)-*N,N*-dimethylpropanamide (6ff)

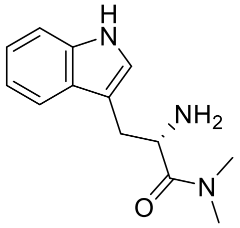

Prepared according to General Procedure C [room temp], and further purified by flash column chromatography (2% MeOH:CH_2Cl_2) to yield a brown oil (62 mg, 54%, er >95:5 determined using Marfey's reagent);

ν_{max} (film/cm^{-1}) 3351, 2920, 2849, 2815, 1638, 1503

δ_H (600 MHz, $CDCl_3$) 8.60 (br s, 1H, N*H*), 7.56 (d, *J* = 7.9 Hz, 1H, Ar*H*), 7.35 (d, *J* = 8.1 Hz, 1H, Ar*H*), 7.18 (t, *J* = 7.2 Hz, 1H, Ar*H*), 7.11 (t, *J* = 7.2 Hz, 1H, Ar*H*), 7.04 (d, *J* = 1.8 Hz, 1H, Ar*H*), 4.05 (dd, *J* = 7.5, 6.4 Hz, 1H, C*H)*, 3.12 (dd, *J* = 14.4, 6.2 Hz, 1H, CH*H*), 2.92 (dd, *J* = 14.4, 7.6 Hz, 1H, CH*H*), 2.90 (s, 3H, CH_3), 2.79 (s, 3H, CH_3), 2.05 (br s, 2H, NH_2);

δ_C (150 MHz, $CDCl_3$) 175.1, 136.4, 127.5, 123.2, 122.2, 119.6, 118.6, 111.6, 111.5, 51.8, 37.0, 35.9, 32.4;

HRMS: Found (ES+): $[M+H]^+$ 232.1445, $C_{13}H_{17}N_3O$, requires 232.1444.

(*S*)-2-Amino-*N,N*-dimethyl-4-(methylthio)butanamide (6gg)

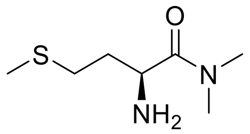

Prepared according to General Procedure C [room temp], and further purified by flash column chromatography (2% MeOH:CH_2Cl_2) to yield a brown oil (34 mg, 39%, er >95:5 determined using Marfey's reagent);

ν_{max} (film/cm^{-1}) 3351, 2918, 2848, 2814, 1638, 1503

δ_H (600 MHz, $CDCl_3$) 3.86 (dd, *J* = 8.9, 4.0 Hz, 1H, C*H*), 3.06 (s, 3H, C*H₃*), 2.96 (s, 3H, C*H₃*), 2.70 (ddd, *J* = 13.1, 8.3, 6.9 Hz, 1H, SCH*H*), 2.62 (ddd, *J* = 12.9, 6.9, 5.4 Hz, 1H, SCH*H*), 2.05 (s, 3H, SC*H₃*), 1.81 (m, 1H, C*H*HCH), 1.66-1.62 (m, 3H, 2 × NH_2, 1× C*H*HCH);

δ_C (150 MHz, $CDCl_3$) 175.5, 49.8, 36.8, 36.0, 34.5, 31.0, 15.8;

HRMS: Found (ES+): $[M+H]^+$ 177.1056, $C_7H_{16}N_2OS$, requires 177.1056.

6. Sequential amidation

Stoichiometric sequential amidation of unprotected amino acids

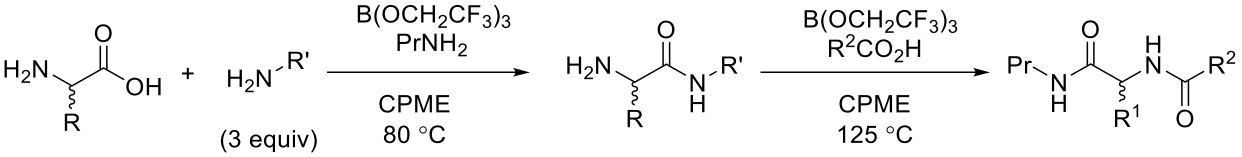

Method D: A solution of $B(OCH_2CF_3)_3$ (3.0 mmol, 3.0 eq) in CPME (1 mL) was added dropwise to a mixture of an unprotected amino acid (1.0 mmol, 1.0 eq) and propylamine (3.0 mmol, 3.0 eq) in CPME (1 mL) over 1 h at 80 °C. The resulting mixture was stirred for 15 h. Upon completion, the mixture was diluted with EtOAc (3 mL) and water (0.5 mL). Amberlite IRA-743 and Amberlyst A-26(OH) were added and stirred for 30 min. The mixture was dried over $MgSO_4$ and then filtered. The solids were washed with EtOAc (3 × 20 mL) and the product concentrated *in vacuo.* The resulting amino amide was then stirred with a carboxylic acid (2 mmol, 2 equiv.) at 125 °C in CPME (1 mL). $B(OCH_2CF_3)_3$ (3.0 mmol, 3.0 equiv.) in CPME (1 mL, unless stated otherwise) was added dropwise to the mixture over a period of 1 h, and the reaction subsequently left to stir for 5 h. Upon completion the mixture was diluted with EtOAc (4.0 mL) and water (0.5 mL). Amberlite IRA-743 (0.5 g), Amberlyst-15 (0.5 g) and Amberlyst A-26(OH) (1.0 g) resins were added, left to stir for 1 hour and the solution dried over $MgSO_4$. The solids were washed with EtOAc (3 × 20 mL) and the filtrate concentrated *in vacuo*. The crude product amide was recrystallised from hot EtOAc to yield a clean amide product.

Catalytic sequential amidations of unprotected amino acids

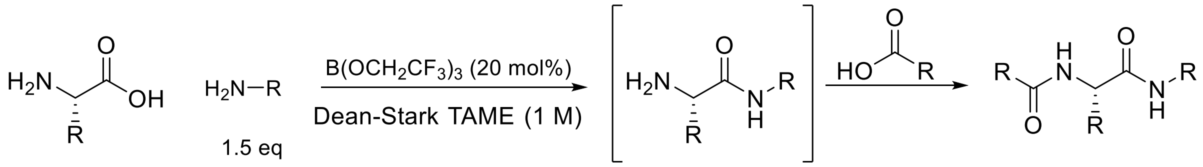

Method E: Following Method B, after heating to reflux for 24 h, a solution of carboxylic acid (10 mmol. 2 eq) in TAME (10 mL) was added dropwise over 10 min into the reaction mixture. The reaction was left to stir for 12-24 h. If the reaction was not complete within these time frames, as seen with the disappearance of the intermediate amino amide by TLC [revealed with ninhydrin stain] or HPLC, a further portion of carboxylic acid (5 mmol) in TAME (4 mL) was added dropwise over 5 min into the reaction mixture. Once complete, the reaction was cooled to room temperature and concentrated *in vacuo*. The product was purified by flash column chromatography.

Note: to achieve maximum yields it is advisable to use HPLC to monitor the reaction upon addition of the carbonyl, as the intermediate aminoamide can be hard to see on TLC.

Catalytic sequential condensations of unprotected amino acids

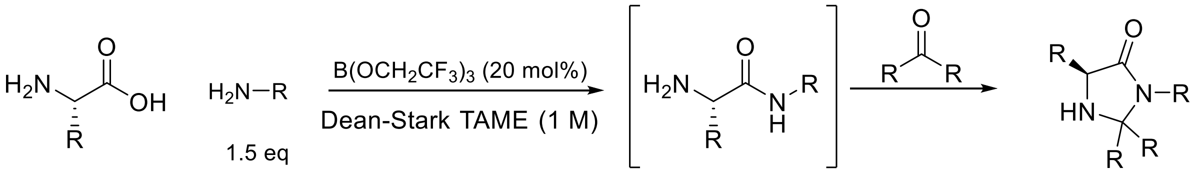

Method F: Following general procedure B, after heating to reflux for 24 h, a solution of aldehyde/ketone (10 mmol) in TAME (5 mL) was added dropwise over 10 min into the reaction mixture. The reaction was left to stir for 1-2 hour. If the reaction was not complete within these time frames, as seen with the disappearance of the intermediate amino amide by TLC [revealed with ninhydrin stain] or HPLC, a further portion of aldehyde/ketone (5 mmol) in TAME (2 mL) was added dropwise over 5 min into the reaction mixture. Once complete, the reaction was cooled to room temperature and concentrated *in vacuo*. The product was purified by flash column chromatography.

Note: to achieve maximum yields it is advisable to use HPLC to monitor the reaction upon addition of the carbonyl, as the intermediate aminoamide can be hard to see on TLC.

Stoichiometric sequential amidation

(*S*)-2-(2-(4-Bromophenyl)acetamido)-3-phenyl-*N*-propylpropanamide (10a)

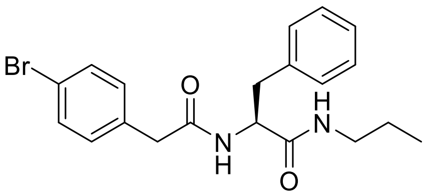

Prepared according to general procedure D to give **(*S*)-2n** in step 1 in 95% yield and **(*S*)-10a** as a white solid (229 mg, 57%, er 87:13, determined using chiral HPLC);

mp 181-182 °C;

$[\alpha]_D^{25}$ −7.8 (*c* 2.0, MeOH);

ν_{max} (solid/cm^{-1}) 3282, 3086, 2963, 1635, 1544;

δ_H (600 MHz, $CDCl_3$) 7.41 (d, *J* = 8.28 Hz, 2H, ArH), 7.28-7.20 (m, 3H, ArH), 7.12-7.07 (m, 2H, ArH), 7.03-6.98 (m, 2H, ArH), 6.50-6.42 (m, 1H, N*H*), 5.93 (br s, 1H, N*H*), 4.63-4.54 (m,

1H, CH), 3.45 (s, 2H, CH_2ArBr), 3.06-2.87 (m, 4H, CH_2Ar, CH_2CH$_2$CH$_3$), 1.40-1.29 (m, 2H, CH_2CH$_3$), 0.78 (t, J = 7.5 Hz, 3 H, CH_3);

δ_C (150 MHz, $CDCl_3$) 170.5, 170.3, 136.6, 133.4, 132.2, 131.1, 129.3, 128.8, 127.2, 121.5, 54.8, 43.0, 41.3, 38.5, 22.6, 11.4;

LRMS (ES+) 403, 405 (^{79}Br, ^{81}Br [M+H]$^+$, 100, 100);

HRMS: Found (ES+): [M+H]$^+$ 403.1028 $C_{20}H_{23}N_2O_2Br$, requires 403.1021.

tert-Butyl (_R_)-methyl(2-oxo-2-((1-oxo-3-phenyl-1-(propylamino)propan-2-yl)amino) ethyl)carbamate (10b)

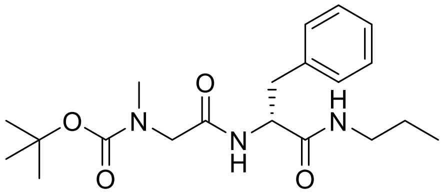

Prepared according to general procedure D to give **(R)-2n** in step 1 in 94% yield and **(R)-10b** as a white solid (256 mg, 68%, er 98:2, determined by chiral HPLC);

mp 133-134°C.

$[\alpha]_D^{20}$ +6.3 (c 1.0, MeOH);

ν_{max} (solid/cm^{-1}) 3281, 2967, 1702, 1644, 1554;

δ_H (400 MHz, $CDCl_3$, 60 °C) 7.35-7.20 (m, 5H, ArH), 6.5 (br s, 1H, NH), 5.9 (br s, 1H, NH), 4.64-4.59 (m, 1H, CHCH$_2$), 3.88 (d, J = 16.5 Hz, 1H, NCHH), 3.74 (d, J = 16.5 Hz, 1H, NCHH), 3.20-3.01 (m, 4H, CH_2Ar, CH_2CH$_2$CH$_3$), 2.84 (s, 3H, NCH$_3$), 1.45 (s, 9H, (CH_3)$_3$), 1.47-1.32 (m, 2H, CH_2CH$_3$), 0.83 (t, J = 7.3 Hz, 3H, CH_3);

δ_C (100 MHz, $CDCl_3$, 60 °C) 170.2, 169.1, 156.6, 136.7, 129.2, 128.6, 126.9, 80.7, 54.2, 53.2, 46.1, 41.3, 35.7, 28.2, 22.5, 11.1;

LRMS (ES+) 378, 777 ([M+H]$^+$, 100, 80);

HRMS: Found (ES+): [M+H]$^+$ 378.2398, $C_{20}H_{32}N_3O_4$, requires 378.2393.

N-(2-Oxo-2-(propylamino)ethyl)picolinamide (10c)

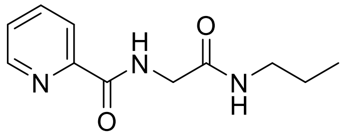

Prepared according to general procedure D to give **2h** in step 1 in 71% yield and **10c** as a white solid (86 mg, 57%);

mp 147-148 °C;

ν_{max} (solid/cm^{-1}) 3334, 3285, 3071, 2962, 1650, 1526;

δ_H (600 MHz, $CDCl_3$) 8.64 (br s, 1H, N*H*), 8.58-8.57 (m, 1H, ArH), 8.17 (d, *J* = 7.7 Hz, 1H, ArH), 7.86 (td, *J* = 7.7, 1.3 Hz, 1H, ArH), 7.46 (ddd, *J* = 7.6, 4.8, 1.3, 1H, ArH), 6.40 (br s, 1 H, N*H)*, 4.15 (d, *J* = 6.0 Hz, 2H, CH_2CON), 3.24 (dt, 2H, *J* = 7.3, 6.1 Hz C$H_2$$CH_2CH_3$), 1.53 (app sx, *J* = 7.3 Hz, 2H, C$H_2$$CH_3$), 0.90 (t, *J* = 7.4 Hz, 3H, CH_3);

δ_C (150 MHz, $CDCl_3$) 168.9, 165.2, 149.3, 148.5, 137.5, 126.7, 122.4, 43.7, 41.4, 22.9, 11.5;

HRMS: Found (ES+): $[M+H]^+$ 222.1237 $C_{11}H_{15}N_3O_2$, requires 222.1237.

N-(2-Oxo-2-(propylamino)ethyl)-2-phenylacetamide (10d)

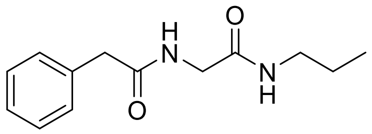

Prepared according to general procedure D to give **2h** in step 1 in 71% yield and **10d** as a white solid (180 mg, 77%);

mp 151-152 °C;

ν_{max} (solid/cm^{-1}) 3254, 3082, 2962, 2873, 1632, 1562;

δ_H (600 MHz, $CDCl_3$) 7.34-7.25 (m, 5H, ArH), 6.75 (br s, 1 H, N*H)*, 6.67 (br s, 1 H, N*H)*, 3.88 (d, *J* = 5.2 Hz, 2H, CH_2CON), 3.58 (s, 2H, ArCH_2), 3.13 (m, 2H, C$H_2$$CH_2CH_3$), 1.53 (app sx, *J* = 7.2 Hz, 2H, C$H_2$$CH_3$), 0.86 (t, *J* = 7.4 Hz, 3H, CH_3);

δ_C (150 MHz, $CDCl_3$) 171.9, 168.8, 134.7, 129.4, 129.1, 127.5, 43.6, 43.4, 41.3, 22.7, 11.4;

LRMS (ES+) 235.1 ($[M+H]^+$, 100);

HRMS: Found (ES+): $[M+H]^+$ 235.1442 $C_{20}H_{32}N_3O_4$, requires 235.1441.

3-Hydroxy-*N*-(2-Oxo-2-(propylamino)ethyl)-2-phenylpropanamide (10e)

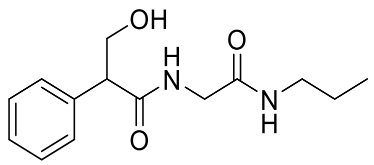

Prepared according to general procedure D to give **2h** in step 1 in 71% yield and **10e** as a white solid (236 mg, 63%);

mp 143-144 °C;

ν_{max} (solid/cm^{-1}) 3372, 3284, 3097, 2961, 2875, 1646, 1575, 1540;

δ_H (600 MHz, $CDCl_3$) δ 7.37-7.29 (m, 5 H, ArH), 6.49 (br s, 1 H, N*H)*, 6.10 (br s, 1 H, N*H)*, 4.17 (dd, *J* = 11.0, 8.8 Hz, 1H, CH*H*OH), 3.91 (dd, *J* = 16.4, 5.6 Hz, 1H CH*H*CON), 3.87 (dd, *J* = 16.4, 5.4 Hz, 1H C*H*HCON), 3.86-3.84 (m, 1H, CH*H*OH), 3.74 (dd, *J* = 8.7, 4.6 Hz, 1H, C*H*), 3.37 (br s, 1H, OH), 3.19-3.17 (m, 2H, $CH_2CH_2CH_3$), 1.53 (app sx, *J* = 7.2 Hz, 2H, CH_2CH_3), 0.89 (t, *J* = 7.5 Hz, 3H, CH_3);

δ_C (150 MHz, $CDCl_3$) 174.1, 168.5, 136.3, 129.3, 128.5, 128.2, 65.0, 54.5, 43.4, 41.4, 22.8, 11.4;

LRMS (ES+) 265.2, 287.1 ([M+H]$^+$, 30; [M+Na]$^+$, 100);

HRMS: Found (ES+): [M+H]$^+$ 265.1555 $C_{20}H_{32}N_3O_4$, requires 265.1552.

Catalytic sequential amidation

tert-Butyl (2-((2-(benzylamino)-2-oxoethyl)(methyl)amino)-2-oxoethyl)(methyl) carbamate (11a)

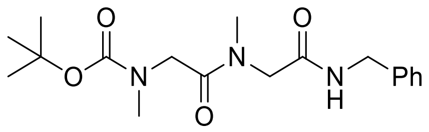

Prepared according to General Procedure E and purified by column chromatography (1:1 EtOAc:Pet) to give title compound as a white solid (1.4 g, 81%);

mp 104-105 °C;

ν_{max} (film/cm^{-1}) 3336, 3029, 2970, 1653, 1551;

δ_H (400 MHz, DMSO, 353 K) mixture of rotamers in a 3†:2‡ ratio: 8.4‡ (br s, 0.4H, N*H*), 8.1† (br s, 0.6H, N*H*), 7.33-7.22 (m, 5H, Ar*H*), 4.32‡† (d, *J* = 5.8 Hz, 2H, CH_2Bn), 4.05‡† (br s, 2H, CH_2), 3.99‡† (br s, 2H, CH_2),), 2.99† (br s, 2H, NCH_3), 2.85‡ (br s, 1H, NCH_3), 2.79‡† (br s, 3H, NCH_3), 1.38‡† (s, 9H, C(CH_3)$_3$);

δ_C (100 MHz, DMSO) 168.8 (C), 168.0 (br, C), 155.4 (C), 139.4 (br, C), 128.2 (CH), 127.2 (br, CH), 126.7 (br, CH), 78.6 (C), 51.6‡ (br, CH_2), 50.7† (br CH_2), 49.6 (br, CH_2), 42.2 (br, CH_2), 35.1 (br, CH_3), 35.1 (CH_3), 28.1 (CH_3);

LRMS (ES+): 350.3 ([M]$^+$, 30), 250.3 ([M-Boc]$^+$, 100);

HRMS: Found (ES+): [M+H]$^+$ 350.2062 $C_{18}H_{28}N_3O_4$,requires 350.2080.

tert-Butyl ((*S*)-1-(((*S*)-1-(benzylamino)-4-methyl-1-oxopentan-2-yl)amino)-1-oxo-3-phenylpropan-2-yl)carbamate (11b)

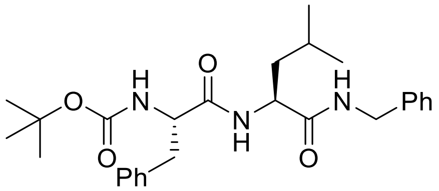

Prepared according to General Procedure E and purified by column chromatography (3:7 EtOAc:Pet) to give title compound as a white solid (1.42 g, 61% dr 85:15);

mp 133-134 °C

[α]$_D$ + 4.3 (*c* 1.0, CH_3Cl, 25 °C);

ν_{max} (solid/cm^{-1}) 3341, 3310, 3024, 2970, 1659, 1522;

^{1}H NMR (400 MHz, DMSO, 353 K, NMR contains 15% of diastereoisomer) δ 8.16-7.96 (m, 1H), 7.69 (d, *J* = 8.24 Hz, 1H), 7.31-7.14 (m, 11H), 4.37 (d, *J* = 7.63 Hz, 1H), 4.32-4.15 (m, 3H), 3.11-2.89 (m, 2H), 2.80 (d, *J* = 9.48 Hz, 1H), 1.62 (d, *J* = 6.77 Hz, 1H), 1.55-1.41 (m, 2H), 1.36-1.14 (m, 11H), 0.92-0.76 (m, 6H);

^{13}C NMR (100 MHz, DMSO, 353 K) δ 171.7, 171.2, 155.1, 139.3, 138.0, 129.1, 128.1, 127.9, 127.9, 127.1, 127.0, 126.6, 126.1, 78.3, 55.9, 51.4, 42.2, 41.4, 37.5, 28.1, 24.2, 22.9, 21.8,

LRMS (ES+): 468.4 ([M]$^+$, 100), 412.3 ([M-56]$^+$, 40);

HRMS: Found (ES+): [M+H]$^+$ 468.2822 $C_{27}H_{38}N_3O_4$,requires 468.2862

(*S*)-*N*-Benzyl-3-(1*H*-indol-3-yl)-2-(2-phenylacetamido)propenamide (11c)

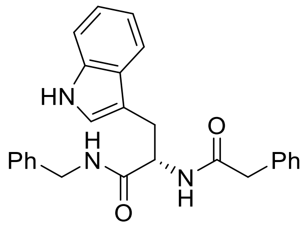

Prepared according to General Procedure E. Upon completion of the reaction, reaction mixture was concentrated in vacuo and extracted with 3 × 100 mL CH_2Cl_2 and 50 mL H_2O. Product was then recrystallised from hot acetone to yield product as a white solid (1.21 g, 69%, >95:5 er measured by chiral HPLC (n-Hex/PrOH; 85/15, 0.5 ml/min; 218 nm; R_t(S) = 50.9 min.));

mp 203-204 °C;

ν_{max} (film/cm^{-1}) 3413, 3292, 3029, 1635, 1539, 1496;

δ_H (600 MHz, $CDCl_3$) 10.9 (br s, 1H, N*H*), 8.55 (t, *J* = 5.8 Hz, 1H, Ar*H*), 8.34 (d, *J* = 8.2 Hz, 1H, Ar*H*), 7.62 (d, *J* = 7.9 Hz, 1H, Ar*H*), 7.35 (d, *J* = 8.0 Hz, 1H, Ar*H*), 7.26-7.16 (m, 6H, Ar*H,* N*H*), 7.12-7.11 (m, 5H, Ar*H*), 7.07 (t, *J* = 7.5 Hz, 1H, Ar*H*), 6.97 (t, *J* = 7.3 Hz, 1H, Ar*H*), 4.63-4.59 (m, 1H, C*H*), 4.30-4.23 (m, 2H, C*H*$_2$), 3.47-3.41 (m, 2H, C*H*$_2$), 3.15 (dd, *J* = 14.4, 5.7 Hz, 1H, C*H*H), 2.98 (dd, *J* = 14.4, 8.7 Hz, 1H, C*H*H);

δ_C (150 MHz, $CDCl_3$) 171.6, 170.0, 139.2, 136.4, 136.1, 129.0, 128.2, 128.1, 127.3, 127.0, 126.2, 123.8, 120.9, 118.6, 118.3, 111.3, 110.0, 53.7, 42.1, 42.0, 28.2

LRMS (ES+): 468.4 ([M+56], 100), 412.3 ([M+H]$^+$, 40);

HRMS: Found (ES+): [M+H]$^+$ 412.2011 $C_{26}H_{26}N_3O_2$,requires 412.2025

(*R*)-2-Acetamido-*N*-benzyl-3-methoxypropanamide (*Lacosamide) (11d)*

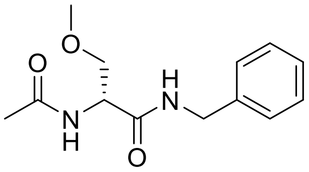

Prepared according to General Procedure E and purified by column chromatography (80:20 EtOAc:Petrol) to give diamide as a white solid (699 mg, 73%, 66:34 er by chiral HPLC (n-Hex/PrOH; 80/20, 0.5 ml/min; 218 nm; R_t(S) = 7.9 min)).

ν_{max} (solid/cm^{-1}) 3284 (br, NH), 3067, 2921, 2888, 2873, 2806, 1631 (C=O), 1539;

δ_H (600 MHz, $CDCl_3$) 7.34-7.23 (m, 5H, Ar*H*), 6.87 (br s, 1H, N*H*), 6.54 (br s, 1H, N*H*), 4.56 (dd, *J* = 7.4, 4.1 Hz, 1H, C*H*), 4.46 (d, *J* = 5.6 Hz, 2H, CONCH_2), 3.78 (dd, *J* = 9.2, 4.1 Hz, 1H, CH*H*), 3.44 (dd, *J* = 9.2, 7.4 Hz, 1H, CH*H*), 3.36 (s, 3H, CH_3O), 2.01 (s, 3H, CH_3CO),

δ_C (150 MHz, $CDCl_3$) 170.5, 170.1, 138.0, 128.8, 127.6, 127.6, 71.9, 59.2, 52.5, 43.7, 23.3;

LRMS (ES+) 251 ([M+H]$^+$, 100);

HRMS: Found (ES+): [M+H]$^+$ 251.1393 $C_{13}H_{18}N_2O_3$, requires 251.1390.

Data in agreement with the literature.[7]

Imidazolidinone cyclisations

3-Benzyl-2-(4-hydroxy-3-methoxyphenyl)-1-methylimidazolidin-4-one (12a)

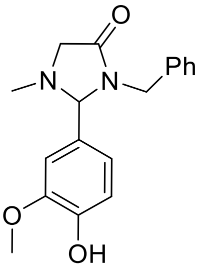

Prepared according to General Procedure F and purified by column chromatography (1:1 EtOAc:Pet) to give an orange oil (1.36 g, 86%);

ν_{max} (film/cm^{-1}) 3013, 2948, 2788, 1687, 1601, 1515;

δ_H (600 MHz, $CDCl_3$) 7.27-7.23 (m, 3H, Ar*H*), 7.03-7.01 (m, 2H, Ar*H*), 6.87 (d, *J* = 7.9 Hz, 1H, Ar*H*), 6.78 (d, *J* = 1.7 Hz, 1H, Ar*H*), 6.70 (dd, *J* = 7.9, 1.7 Hz, 1H, Ar*H*), 5.95 (br s, 1H, O*H*), 4.82 (d, *J* = 14.8 Hz, C*H*H), 4.41 (s, 1H, C*H*), 3.81 (s, 3H, OC*H*$_3$), 3.79 (d, *J* = 14.5 Hz, 1H, C*H*H), 3.55 (d, *J* = 14.8 Hz, 1H, C*H*H), 3.18 (dd, *J* = 14.3, 2.2 Hz, 1H, C*H*H), 2.24 (s, 3H, C*H*$_3$);

δ_C (150 MHz, $CDCl_3$) 171.3, 147.3, 147.0, 136.5, 128.6, 128.6, 128.4, 127.6, 122.7, 113.8, 110.1, 83.4, 57.6, 56.0, 43.9, 38.6;

LRMS (ES+): 313.2 ([M]$^+$, 90), 625.3 ([2M+H]$^+$, 100);

HRMS: Found (ES+): [M+H]$^+$ 313.1563 $C_{18}H_{21}N_2O_3$, requires 313.1552.

3-Benzyl-1-methyl-2-(1*H*-pyrrol-2-yl)imidazolidin-4-one (12b)

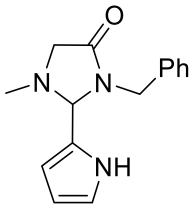

Prepared according to General Procedure F and purified by column chromatography (1:1 EtOAc:Pet) to give a brown oil (0.624 g, 49%);

ν_{max} (film/cm^{-1}) 3246, 1677, 1409;

δ_H (600 MHz, $CDCl_3$) 8.94 (br s, 1H, N*H*), 7.30-7.25 (m, 3H, Ar*H*), 7.12-7.11 (m, 2H, Ar*H*), 6.84 (br m, 1H, Ar*H*), 6.23-6.22 (br m, 1H, Ar*H*), 6.17-6.15 (app q, *J* = 2.7 Hz, 1H, Ar*H*), 4.78 (d, *J* = 14.7, 1H, C*H*H), 4.65 (s, 1H, C*H*), 3.72 (d, *J* = 14.3 Hz, 1H, C*H*H), 3.58 (d, *J* = 14.7 Hz, 1H, C*H*H), 3.72 (dd, *J* = 14.2, 2.5 Hz, 1H, C*H*H), 2.29 (s, 3H, CH_3);

δ_C (150 MHz, $CDCl_3$) 170.8, 136.6, 128.6, 128.6, 127.6, 126.1, 119.9, 111.5, 108.1, 76.7, 57.5, 43.9, 38.8;

LRMS (ES+): 256.1 ($[M]^+$, 40), 419.1 ($[M+163]^+$, 100);

HRMS: Found (ES+): $[M+H]^+$ 256.1443 $C_{15}H_{18}N_3O$, requires 256.1450.

1-Benzyl-4-methyl-1,4-diazaspiro[4.5]decan-2-one (12c)

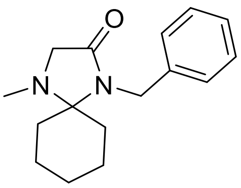

Prepared according to General Procedure F and purified by column chromatography (1:2 EtOAc:Pet) to give a clear oil (1.053 g, 82%);

Rf: product 0.6 (CH_2Cl_2:MeOH, 96:4) [Rf of intermediate: 0.5]

ν_{max} (film/cm^{-1}) 3345, 2931, 2856, 2790, 1687, 1605

^{1}H NMR ($CDCl_3$, 400 mHz) δ 7.31-7.24 (m, 5H, Ar*H*) 4.38 (2H, CH_2Bn), 3.39 (s, 2H, CH_2NMe), 2.32 (s, 3H, CH_3), 1.49-1.68 (m, 7H, Cy*H*), 1.83-1.40 (m, 2H, Cy*H*), 1.09-1.13 (m, 1H, Cy*H*)

^{13}C NMR (150 MHz, $CDCl_3$) δ 173.6, 138.7, 128.5, 127.3, 127.2, 83.7, 58.0, 43.1, 32.1, 39.1, 25.1, 22.8

LRMS (ES+) 259.2 ($[M+H]^+$, 100), 516.4 ($[2\times M+H]^+$, 40)

HRMS (ES+): Found (ES+): [M+H]+ 259.1815, ($[C_{16}H_{22}N_2O+H]^+$), requires 259.1810

3-Benzyl-2,2-diethyl-1-methylimidazolidin-4-one (12d)

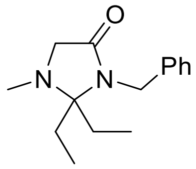

Prepared according to General Procedure F and purified by column chromatography (1:2 EtOAc:Pet) to give a yellow oil (0.692 g, 56%);

ν_{max} (film/cm^{-1}) 3289, 2970, 2789, 1687, 1415;

δ_H (600 MHz, $CDCl_3$) 7.42-7.40 (m, 2H, Ar*H*), 7.29-7.23 (m, 3H, Ar*H*), 4.36 (s, 2H, CONC*H*$_2$), 3.56 (s, 2H, NC*H*$_2$), 2.38 (s, 3H, NC*H*$_3$), 1.62-1.55 (m, 2H, 2 × CH*H*CH_3), 1.52-1.46 (m, 2H, 2 × CH*H*CH_3), 0.53 (t, *J* = 7.3 Hz, 6H, 2 × C*H*$_3$);

δ_C (150 MHz, $CDCl_3$) 172.5, 137.9, 129.0, 128.4, 127.5, 84.5, 56.7, 43.7, 32.5, 29.2, 7.6;

LRMS (ES+): 493.3 ($[2M+H]^+$, 100), 247.2 ($[M+H]^+$, 20);

HRMS: Found (ES+): $[M+H]^+$ 247.1803 $C_{26}H_{34}N_3O_3$, requires 247.1810

tert-Butyl (*S*)-1,3-dibenzyl-2-oxo-1,4,8-triazaspiro[4.5]decane-8-carboxylate (12f)

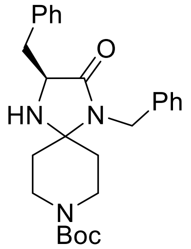

Prepared according to General Procedure F and purified by column chromatography (3:2 EtOAc:Pet) to give a clear oil (1.343 g, 61%);

ν_{max} (film/cm^{-1}); 2979, 2930, 1736, 1682, 1414;

δ_H (600 MHz, $CDCl_3$) δ 7.33-7.27 (m, 5H, Ar*H*), 7.22-7.20 (m, 3H, Ar*H*), 7.04-7.02 (m, 2H, Ar*H*), 4.68 (d, *J* = 15.8 Hz, 1H, NC*H*H), 4.06 (d, *J* = 15.8 Hz, 1H, NC*H*H), 4.0 (br s, 1H, N*H*), 3.86 (t, *J* = 5.0 Hz, C*H*), 3.26 (dd, *J* = 14.1, 5.4 Hz, C*H*HPh), 3.12 (dd, *J* = 14.1, 4.7 Hz, C*H*HPh), 3.02 (br t, *J* = 10.3 Hz, 1H, C*H*H), 2.80 (br t, *J* = 10.7 Hz, 1H, C*H*H), 1.67-1.58 (m, 4H, C*H*$_2$), 1.41 (s, 9H, 3 × C*H*$_3$), 1.39-1.36 (m, 1H, CH*H*), 0.70-0.67 (m, 1H, CH*H*);

δ_C (150 MHz, $CDCl_3$) 174.6, 154.6, 137.9, 136.6, 130.1, 128.7, 128.5, 127.3, 127.2, 127.1, 79.9, 58.6, 43.4, 40.1, 37.0, 36.7, 33.6, 28.4;

LRMS (ES+): 436.3 ($[M]^+$, 60), 871.5 ($[2M+H]^+$, 100);

HRMS: Found (ES+): $[M+H]^+$ 436.2587 $C_{26}H_{34}N_3O_3$, requires 436.2600

1-Benzyl-8-(tert-butyl)-3-phenyl-1,4-diazaspiro[4.5]decan-2-one (12i)

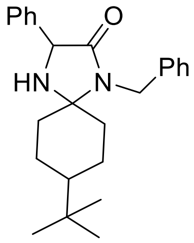

Prepared according to General Procedure F and purified by column chromatography (1:3 EtOAc:Pet) to give a clear oil (626 mg, 33%);

mp 109-110 °C;

ν_{max} (film/cm^{-1}) 3306, 2957, 1665, 1409, 1352;

δ_H (400 MHz, $CDCl_3$) 7.54-7.52 (m, 2H, Ar*H*), 7.41-7.37 (m, 2H, Ar*H*), 7.33-7.30 (m, 5H, Ar*H*), 7.27-7.25 (m, 1H, Ar*H*), 4.69 (s, 1H, C*H*), 4.66 (d, *J* = 15.5 Hz, 1H, C*H*H), 4.32 (d, *J* = 15.5 Hz, 1H, C*H*H), 2.00 (br s, 1H, *NH*), 1.75-1.62 (m, 5H, Cy*H*), 1.48-1.26 (m, 3H, Cy*H*), 1.00-0.90 (m, 1H, Cy*H*), 0.85 (s, 9H, $(CH_3)_3$)

δ_C (100 MHz, $CDCl_3$) 173.7, 139.0, 138.7, 128.7, 128.5, 127.9, 127.6, 127.5, 127.2, 78.4, 61.6, 47.0, 43.6, 38.0, 35.1, 32.3, 23.8, 23.2;

LRMS (ES+): 377.3 ($[M+H]^+$, 100);

HRMS: Found (ES+): $[M+H]^+$ 377.2576, $C_{25}H_{33}N_2O$, requires 37702593

3-(2-(1*H*-Indol-3-yl)ethyl)-2-ethyl-2,5-dimethylimidazolidin-4-one (12j)

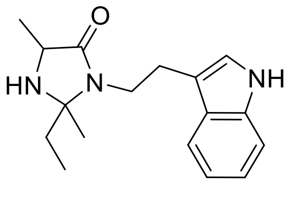

Prepared according to General Procedure F and purified by column chromatography (1:2 EtOAc:Pet) to give a brown oil (590 mg, 41%);

ν_{max} (film/cm^{-1}) 3278, 3971, 2877, 1667, 1620, 1455;

δ_H (600 MHz, $CDCl_3$, mixture of diastereoisomers) 8.7 (br s, 1H, N*H*), 7.71 (t, *J* = 8.4 Hz, 1H, Ar*H*), 7.37 (d, *J* = 8.7 Hz, 1H, Ar*H*), 7.22-7.19 (m, 1H, Ar*H*), 7.16-7.13 (m, 1H, Ar*H*), 7.03 (br s, 1H, N*H*), 3.64† (q, *J* = 7.0 Hz, 0.5 H), 3.64-3.60‡ (m, 0.5H), 3.58‡ (q, *J* = 6.8 Hz, 0.5H), 3.47−3.36† (m, 1H), 3.26−3.21‡ (m, 0.5H), 3.15-3.06‡† (m, 2H), 1.76-1.55‡† (m, 2H), 1.40‡† (d, *J* = 6.8 Hz, 3H), 1.31† (s, 1.5H, CH_3), 1.28‡ (s, 1.4H, CH_3), 0.90† (t, *J* = 7.3 Hz, 1.5 H), 0.89‡ (t, *J* = 7.3 Hz, 1.4 H);

δ_C (150 MHz, $CDCl_3$) 176.2‡, 176.1†, 136.3‡†, 127.5‡, 127.4†, 122.4‡, 122.3†, 122.1‡†, 119.4†, 119.4 ‡, 118.9‡, 118.9†, 113.0‡, 112.9†, 111.5‡†,79.0‡, 78.8†, 54.6†, 53.7‡, 41.9‡, 41.8†, 32.4†, 32.2‡, 27.1†, 25.3‡, 24.9†, 24.7‡, 18.7†, 17.8‡, 8.5†, 7.6‡

LRMS (ES+): 286.2 ($[M+H]^+$, 100), 593.3 ($[2M+H]^+$, 100);

HRMS: Found (ES+): $[M+H]^+$ 286.1914 $C_{17}H_{23}N_3O$, requires 286.1919

Scale up procedures:

*Procedures for **1b** (using 40 mol% $B(OCH_2CF_3)_3$) and **1d** following general procedure B at 1.5 M concentration.*

2,2-Diethyl-3-hexyl-1-methylimidazolidin-4-one (12m)

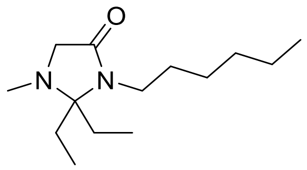

Scale up: A stirred suspension of hexylamine (49.3 mL, 0.375 mol) and sarcosine (22.3 g, 0.250 mol) in TAME (40 mL) with a Dean-Stark was heated to reflux (bp, 86 °C) and $B(OCH_2CF_3)_3$ (50 mmol, 10.1 mL in 43 mL TAME) was added through the Dean-Stark. An air condenser was fitted, and the reaction mixture stirred for 24 hours at which point a solution of 3-pentanone (52 mL in 42 mL TAME) was added dropwise over 1 h into the reaction mixture. The reaction was left to stir for 2 hours and a further portion of 3-pentanone (26 mL in 21 mL TAME) was added dropwise over 30 min, and left to stir for 4 hours.

The reaction mixture was reduced to half of the reaction volume by opening the dean stark arm trap. 40 mL of PhMe was added to the reaction mixture and the reaction mixture heated to reflux (for azeotropic removal of hexylamine). After 30 min, the side arm of the Dean-Stark was again opened and drained. Another 40 mL portion of PhMe was added and the process repeated. At this point, any remaining solvents were removed *in vacuo* and the resulting oil (~110 g) were purified by column chromatography (Petrol:EtOAc) to yield imidazolidinone as a brown oil (49.7g, 83%).

ν_{max} (film/cm^{-1}); 2968, 2930, 1736, 1730, 1460;

δ_H (600 MHz, $CDCl_3$) 3.45 (s, 2H, CH_2), 3.06 (t, J = 8.3 Hz, 2H, NCH_2CH$_2$), 2.41 (s, 3H, CH_3), 1.69-1.63 (m, 2H, 2 ×CCH*H*CH$_3$), 1.63-1.58 (m, 2H, NCH$_2$CH_2), 1.55-1.50 (m, 2H, 2 × CCH*H*CH$_3$), 1.34-1.29 (m, NCH$_2$CH$_2$CH_2CH_2CH_2), 0.88 (t, J = 6.6 Hz, 3H, CH$_2$CH$_2$CH_3), 0.75 (t, J = 7.3 Hz, 2 × CCH$_2$CH_3);

δ_C (150 MHz, $CDCl_3$) 172.2, 84.2, 56.8, 40.6, 32.6, 31.5, 29.3, 28.4, 27.2, 22.6, 14.0, 8.0;

LRMS (ES+): 241.3 ($[M+H]^+$, 50), 413.3 ($[2M+H]^+$, 100),

HRMS: Found (ES+): $[M+H]^+$ 241.2287 $C_{14}H_{29}N_2O$, requires 241.2280

(*S*)-2-Amino-*N*-benzyl-3-phenylpropanamide (1a)

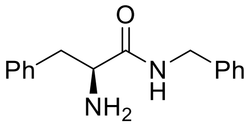

Scale up: A stirred suspension of benzylamine (10.0 mL, 90 mol) and phenylalanine (10.0 g, 60 mmol) in TAME (25 mL) with a Dean-Stark was heated to reflux (bp, 86 °C) and $B(OCH_2CF_3)_3$ (6 mmol, 2.6 mL in 20 mL TAME) was added through the Dean-Stark. An air condenser was fitted, and the reaction mixture stirred for 24 hours, at which point the reaction mixture was filtered (hot vacuum filtration at ~60 °C) and washed with 2 portions of hot TAME (2 × 10 mL). The reaction mixture was left to stand under refrigeration at ~5 °C for 72 h, filtered and washed with 6 portions of 10 mL heptane (crop 1, 8.65 g, 56.7%). The filtrate was concentrated in vacuo, and re-dissolved in 20 mL of TAME. The solution was left to stand under refrigeration at ~5 °C for 72 h, filtered and washed with 2 portions of 10 mL heptane (crop 2, 1.62 g, 11%, at 84% purity levels, as determined by ^{1}H NMR). Enantiopurity was determined to be >95:5, using Marfey's reagent.

7. Origins of chemoselectivity

Interaction of phenylalanine with Lewis-acids.

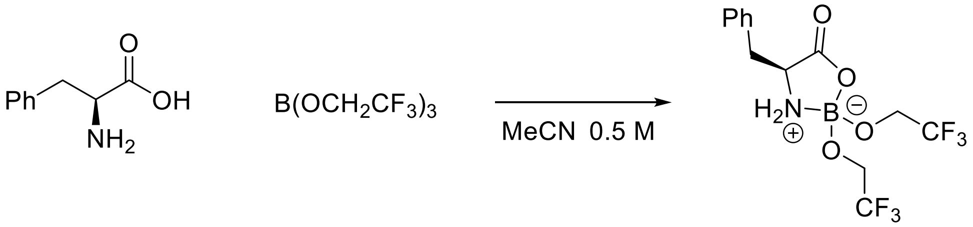

Procedure:

$B(OCH_2CF_3)_3$ (307 mg, 1 mmol) was added to a suspension of phenylalanine (165 mg, 1 mmol) in 2 mL CD_3CN and sonicated at 40 °C for 30 min. A 0.6 mL aliquot was taken and subjected to spectroscopic analysis.
Reaction was also conducted in DMSO-d_6, but is less informative as ^{11}B NMR of $B(OCH_2CF_3)_3$ is tetrahedral in DMSO-d_6 (from Me2S^+−O^- interaction with borate), and thus shows no shift.

^{1}H and ^{13}C NMRs for the CD_3CN experiment are shown below (^{11}B NMR in the main paper)

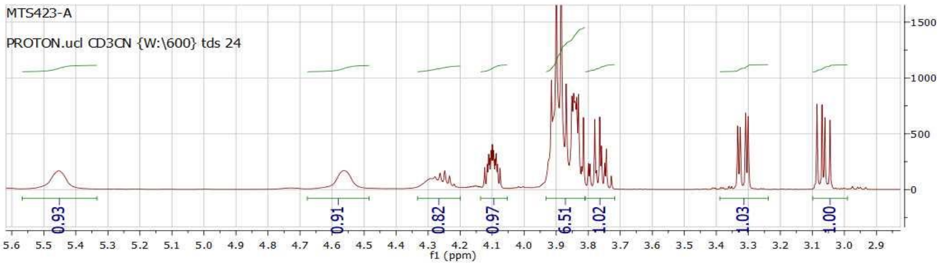

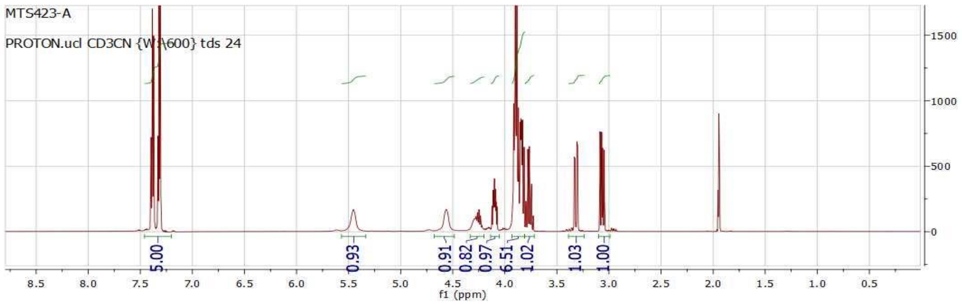

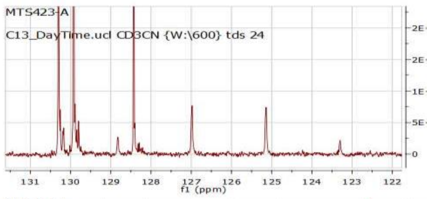

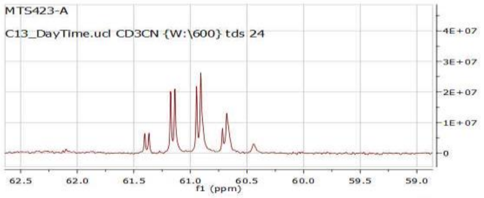

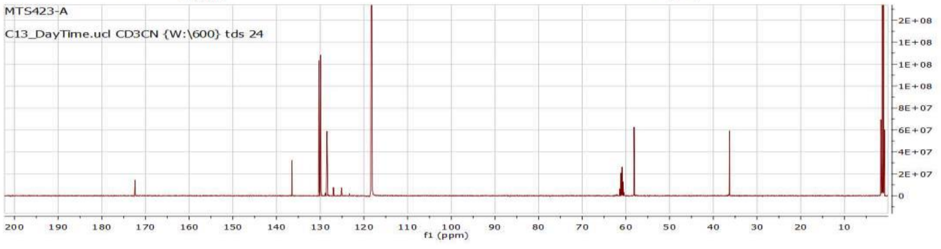

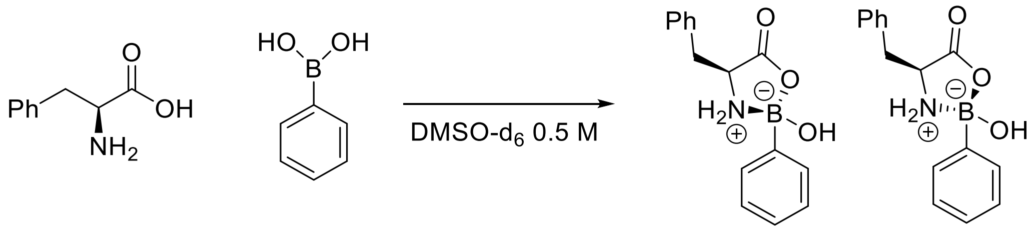

Procedure:

Phenylboronic acid (166 mg, 1 mmol) was added to a suspension of phenylalanine (165 mg, 1 mmol) in 2 mL DMSO-d_6 and sonicated at 40 °C for 30 min. A 0.6 mL aliquot was taken and subjected to spectroscopic analysis.

^{1}H, ^{13}C and ^{11}B NMRs are shown below. As can be seen from ^{1}H and ^{13}C NMR, 2 sets of signals appear corresponding to diastereoisomers, in a 6:4 ratio.

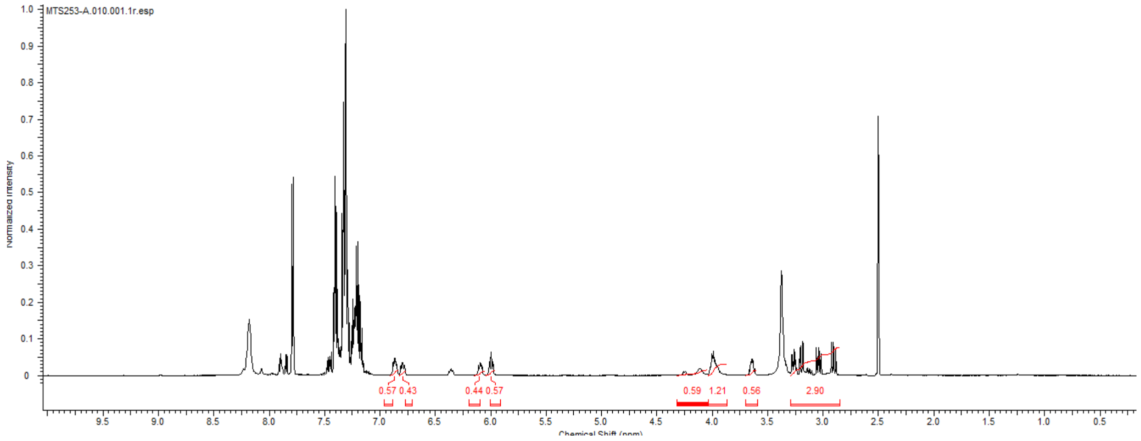

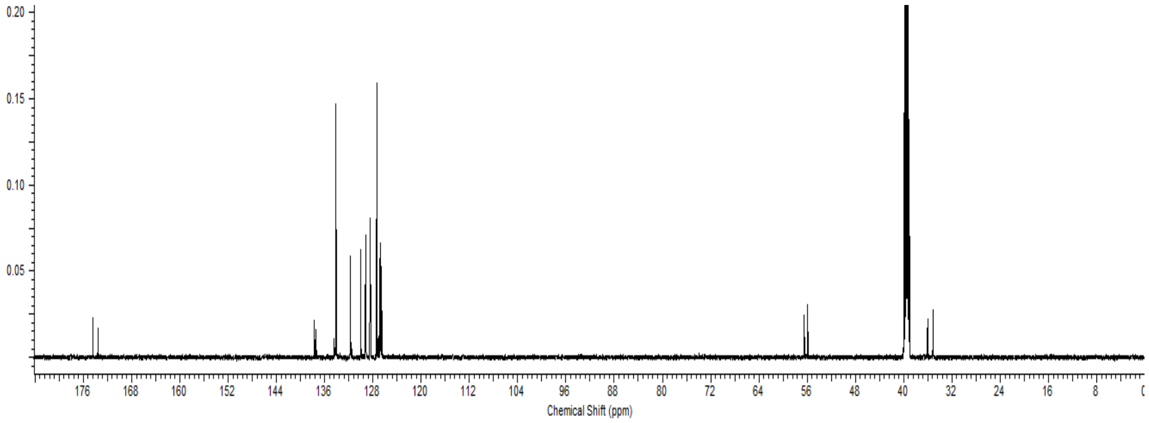

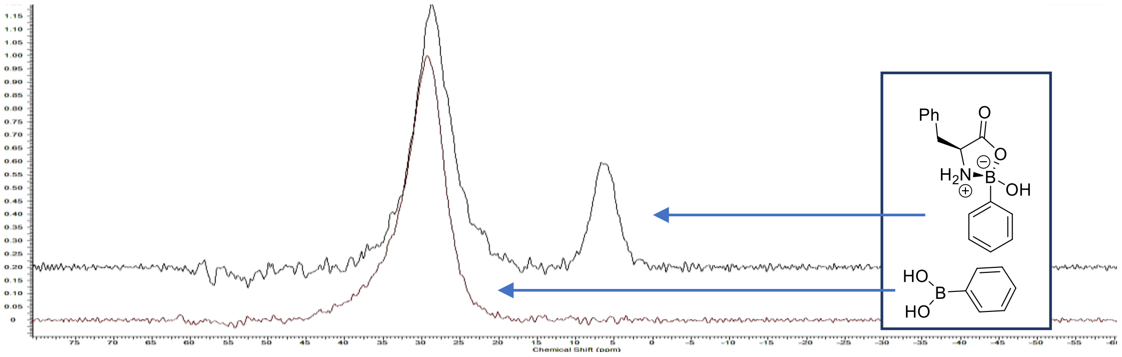

8. ^{1}H and ^{13}C NMR spectra

The spectra for compounds **2a-2w**, **2z, 6a, 6b, 6c, 6f, 6k, 6q, 6r, 6v, 6x** and **6aa** can be found in our previous report.[7]

The spectra for compounds **1a, 1b, 1d, 1f, 1g-j, 1l, 1w, 1dd, 6d, 6e, 6w, 6z, 6aa, 12c, 12g, 12h, 12k, and 12l** can be found in our previous report.[16]

(*S*)-2-Amino-2-phenyl-*N*-propylacetamide (2x)

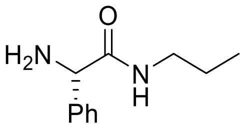

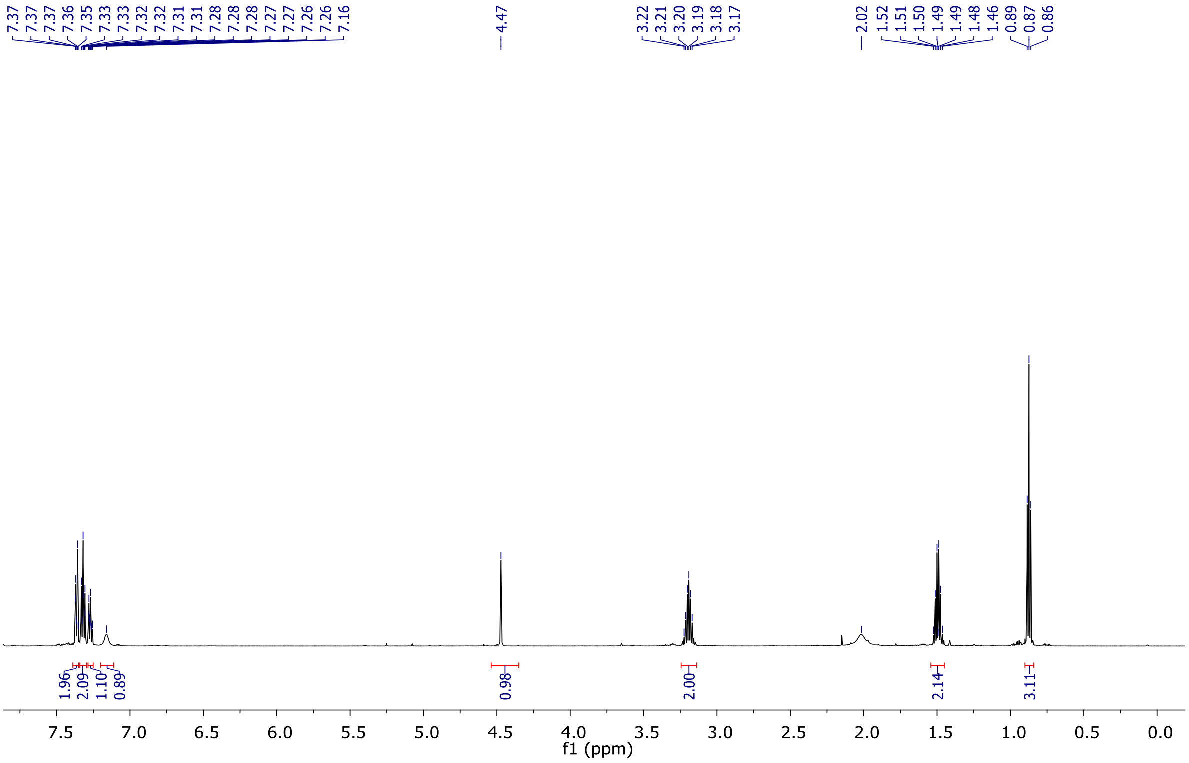

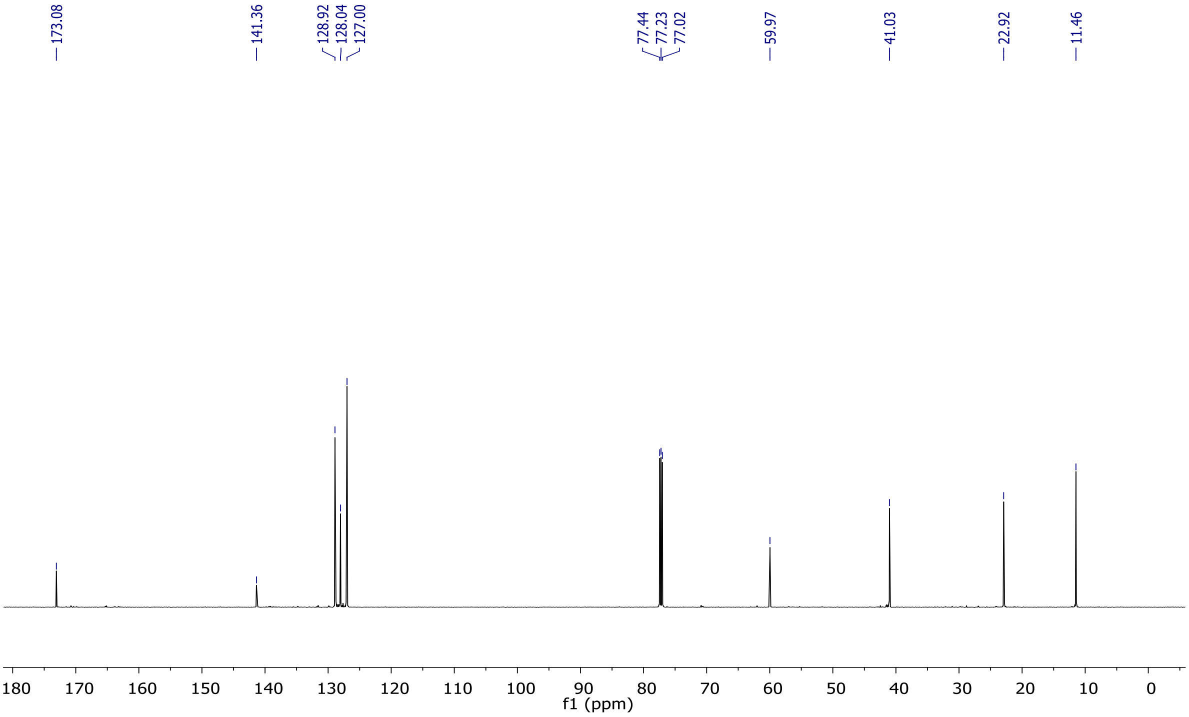

(*S*)-2-Amino-4-hydroxy-*N*-propylbutanamide (2y)

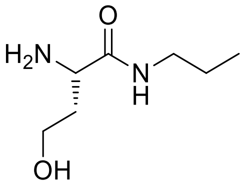

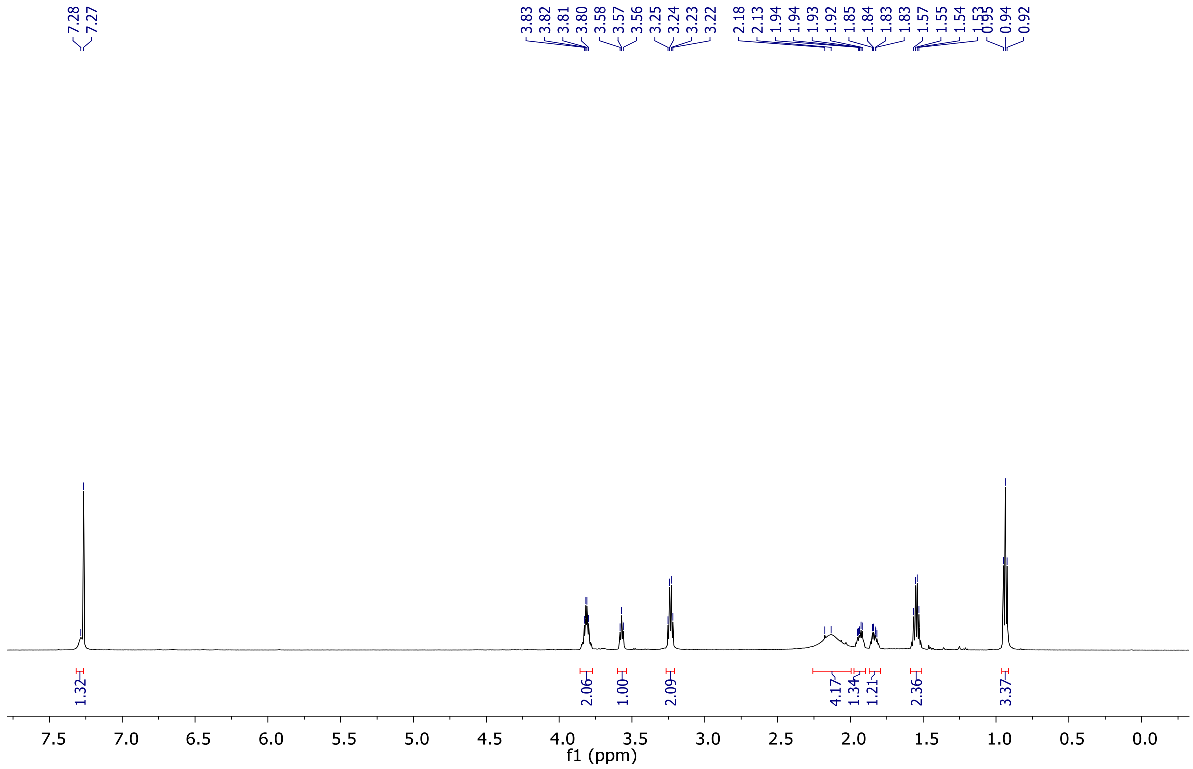

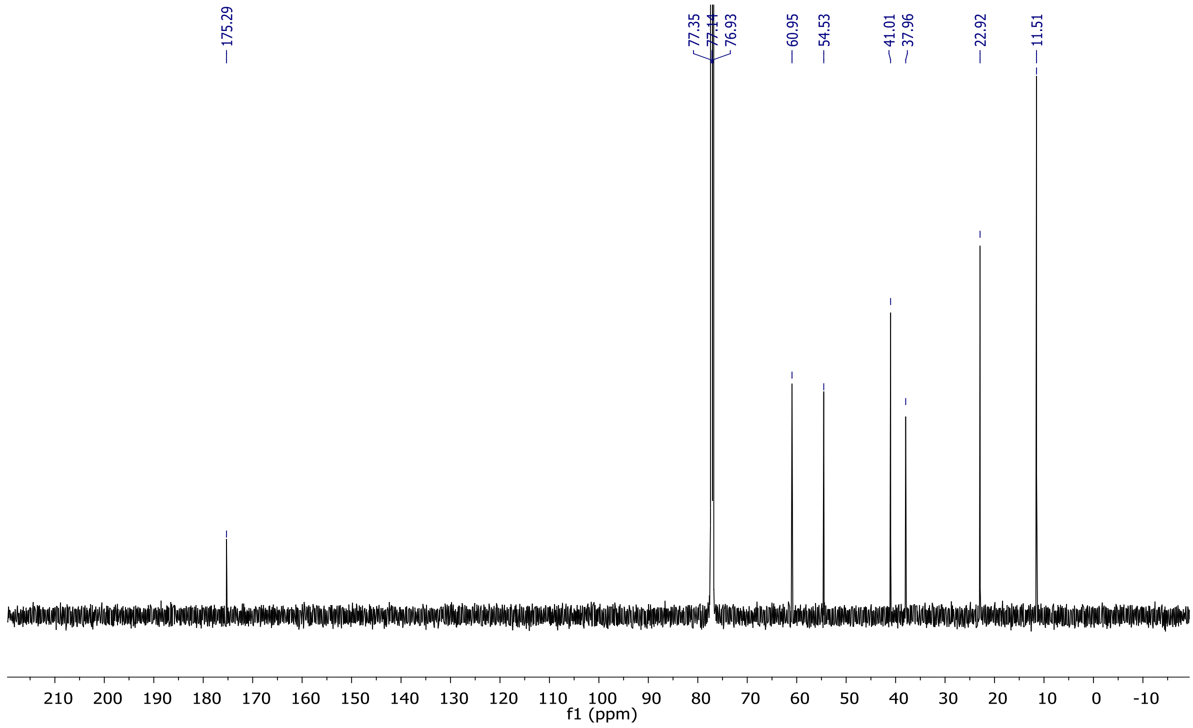

(S)-2-Amino-*N*-benzyl-3-(4-hydroxyphenyl)propanamide (1c)

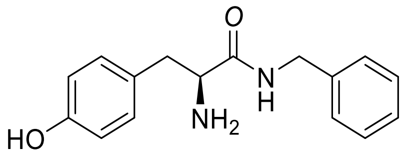

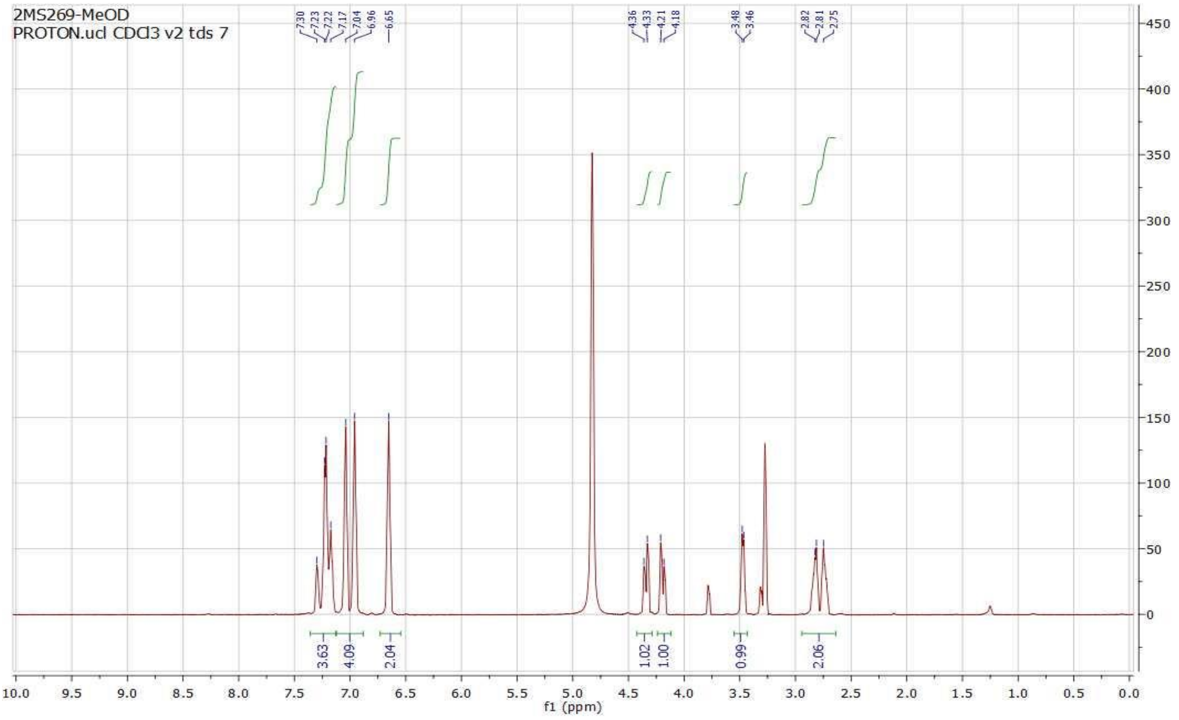

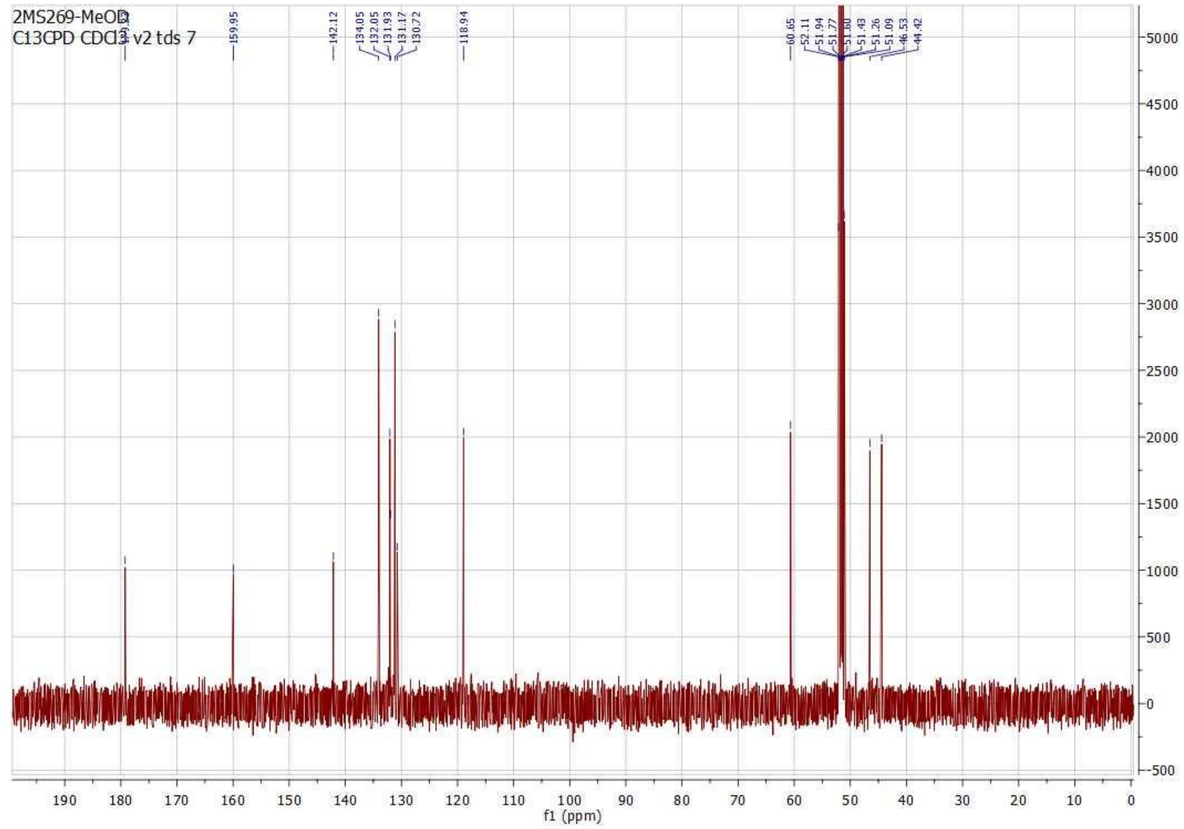

2-Amino-*N*-benzylacetamide (1e)

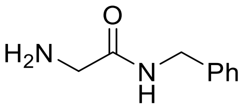

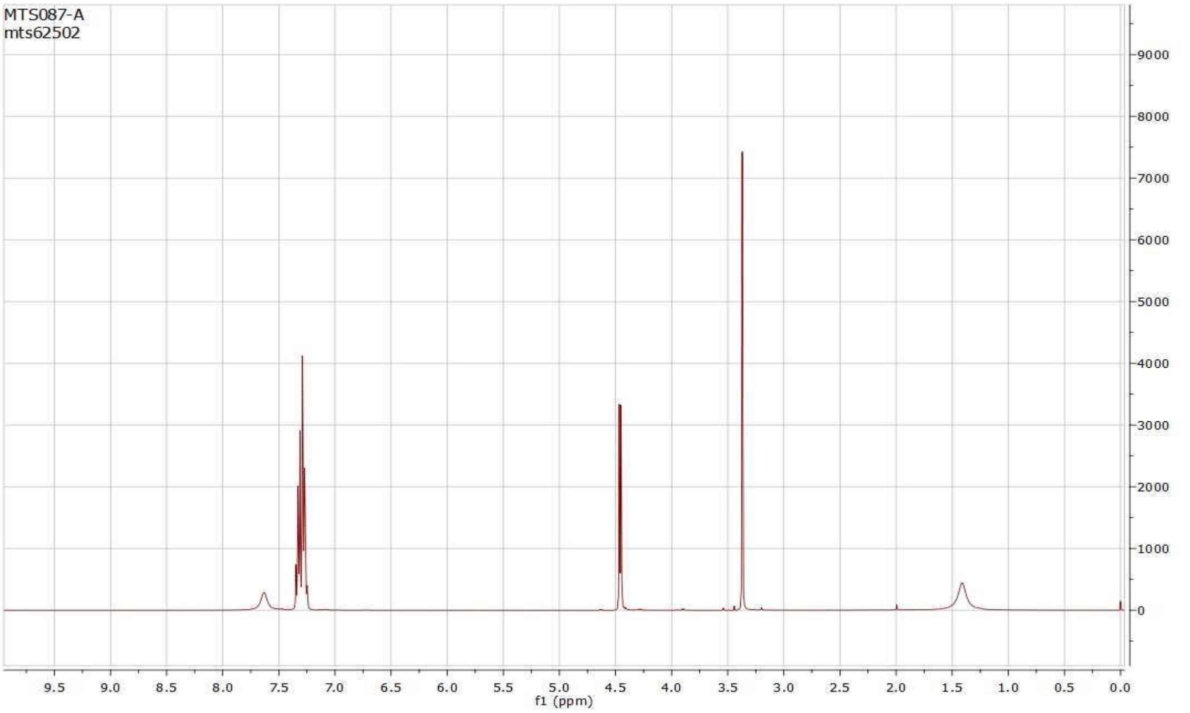

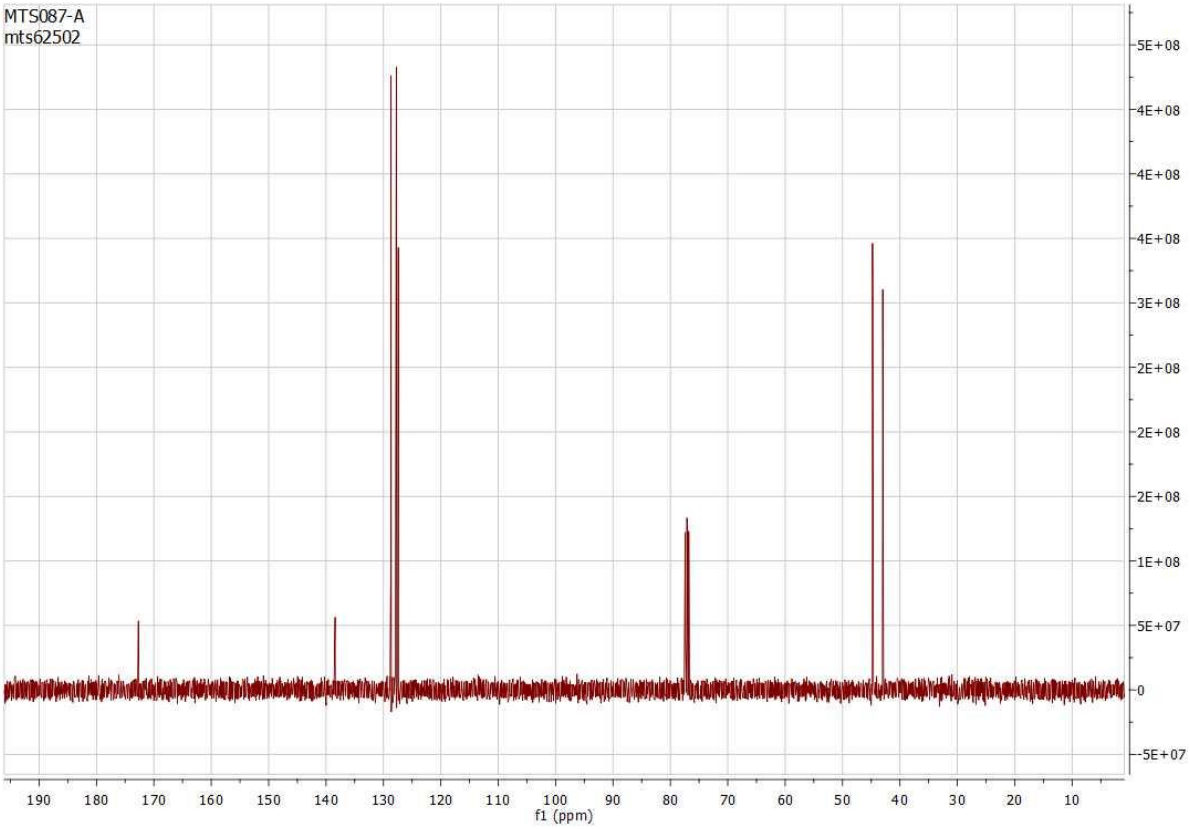

2-Amino-*N*-benzyl-3-mercaptopropanamide (1k)

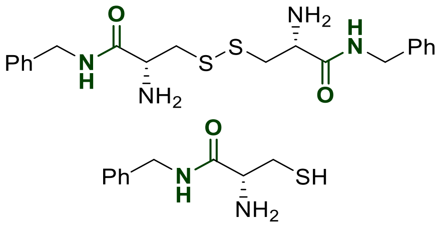

Crude reaction mixture, with internal standard (1,4 dimethoxybenzene, δ 6.8, 3.7 ppm). CH_2Ph, δ 4.4 ppm integrates to 1.81, hence divided by 2 = 0.90.

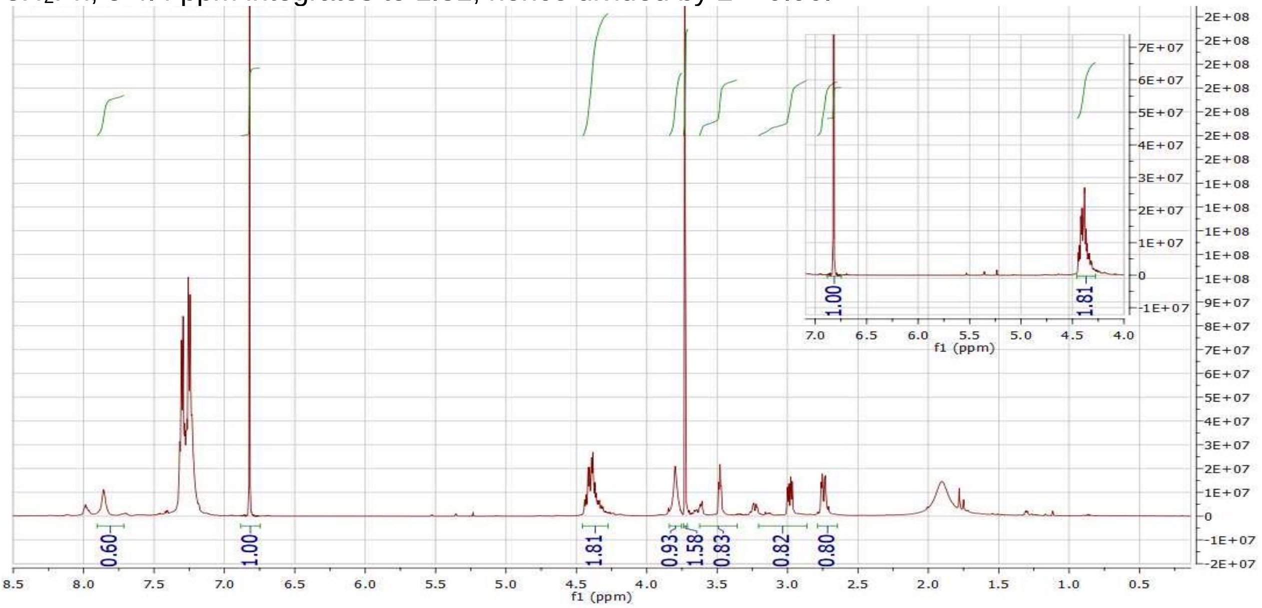

Mixture of products following chromatographic purification:

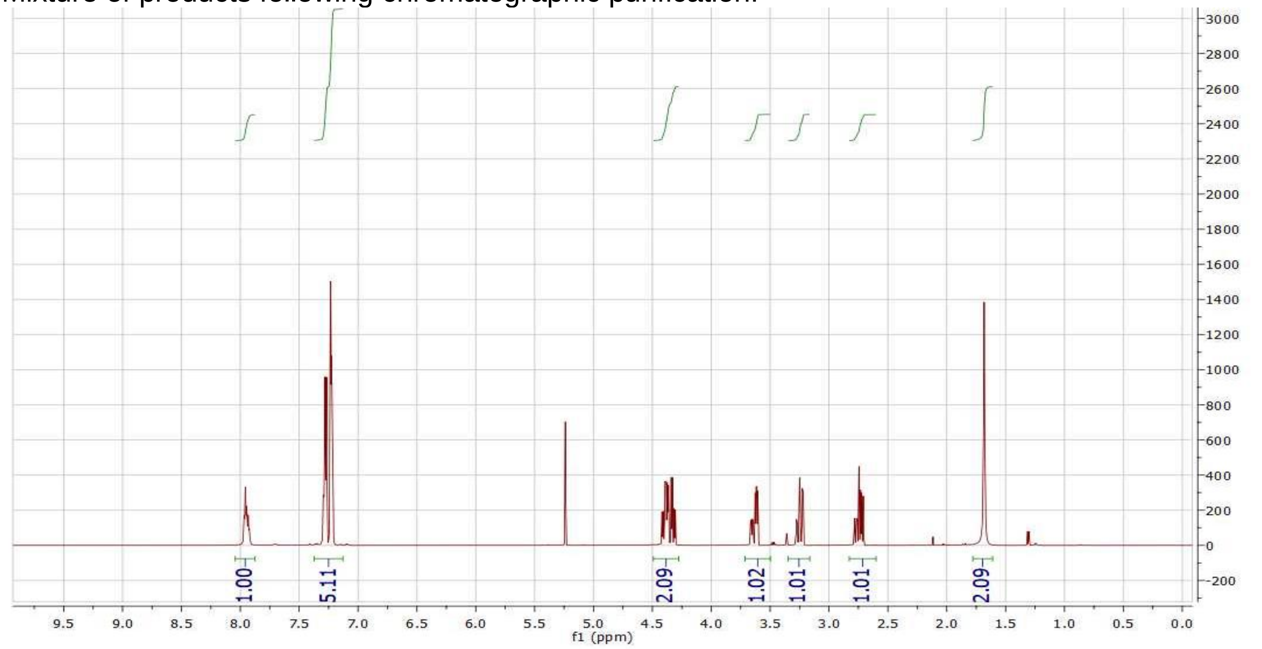

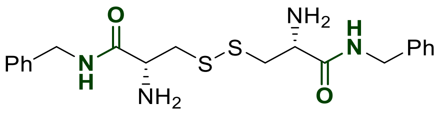

2-Amino-*N*-benzyl-3-mercaptopropanamide (1k)

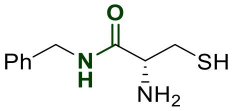

Mixture of products following chromatographic purification:

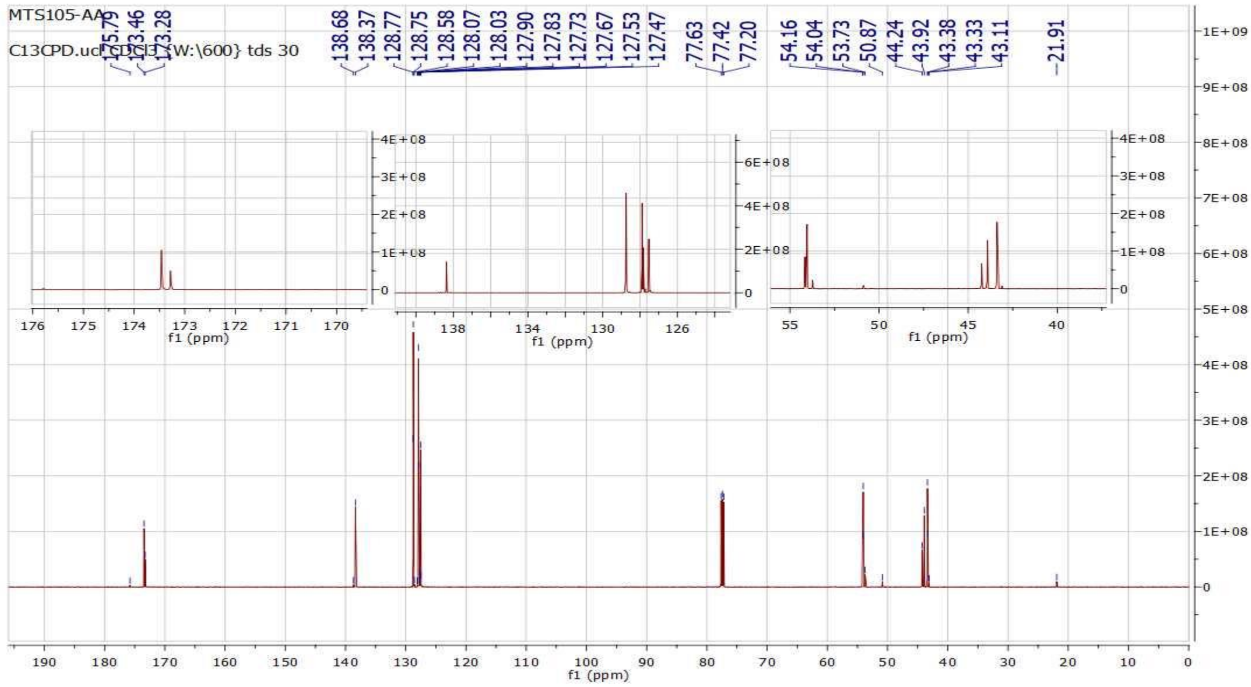

(*S*)-2-Amino-*N*-benzyl-3-hydroxypropanamide (1m)

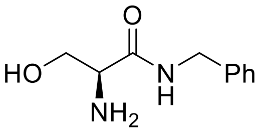

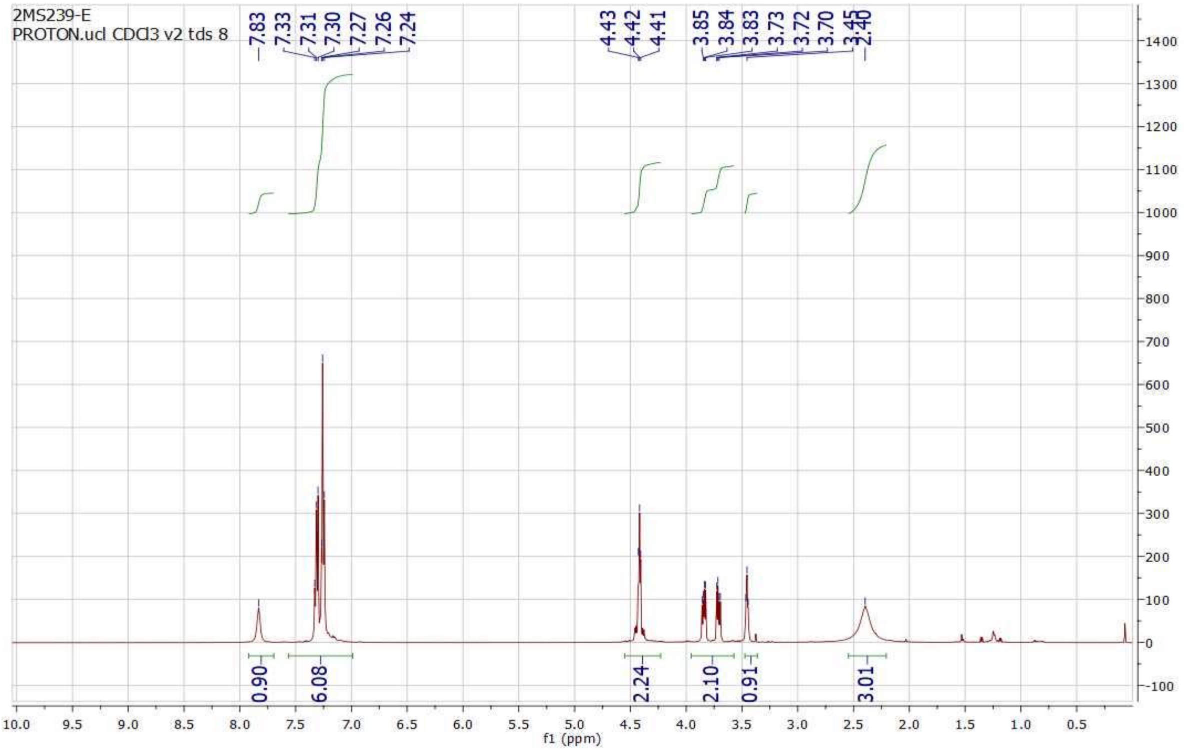

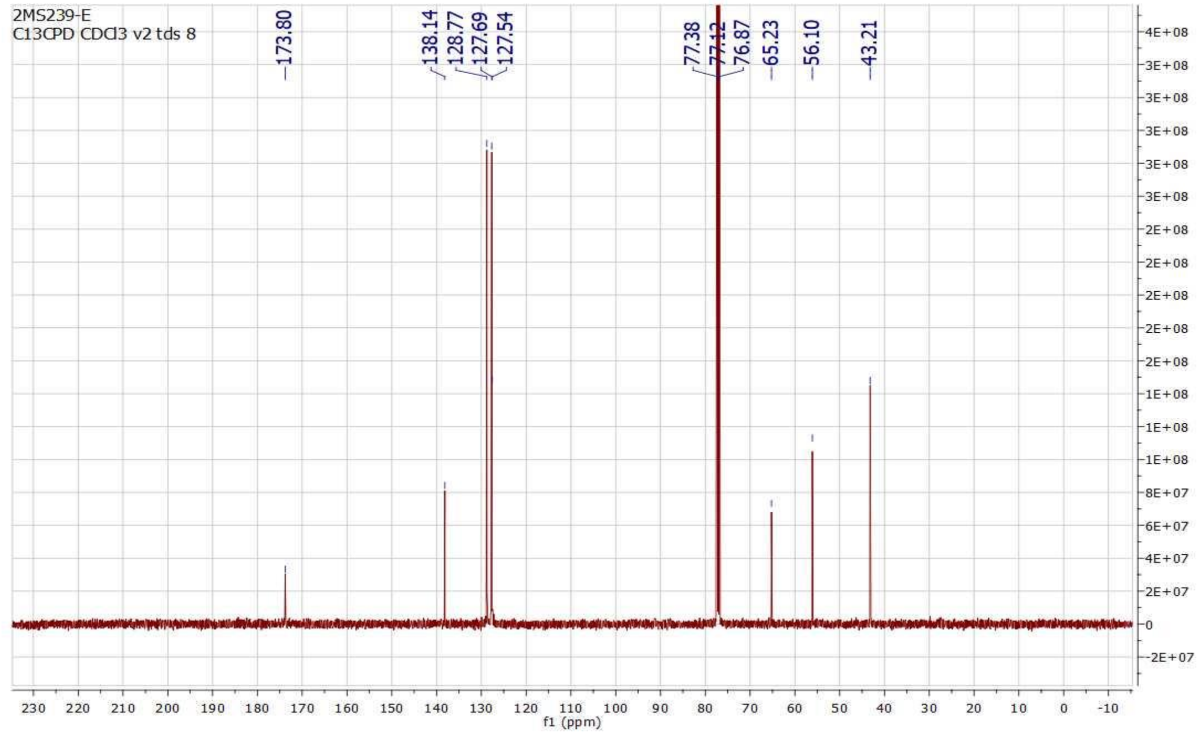

(*S*)-2-Amino-*N*[1],*N*[4]-dibenzylsuccinamide (1o)

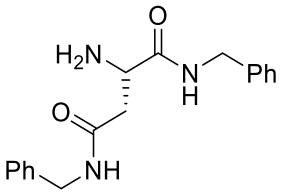

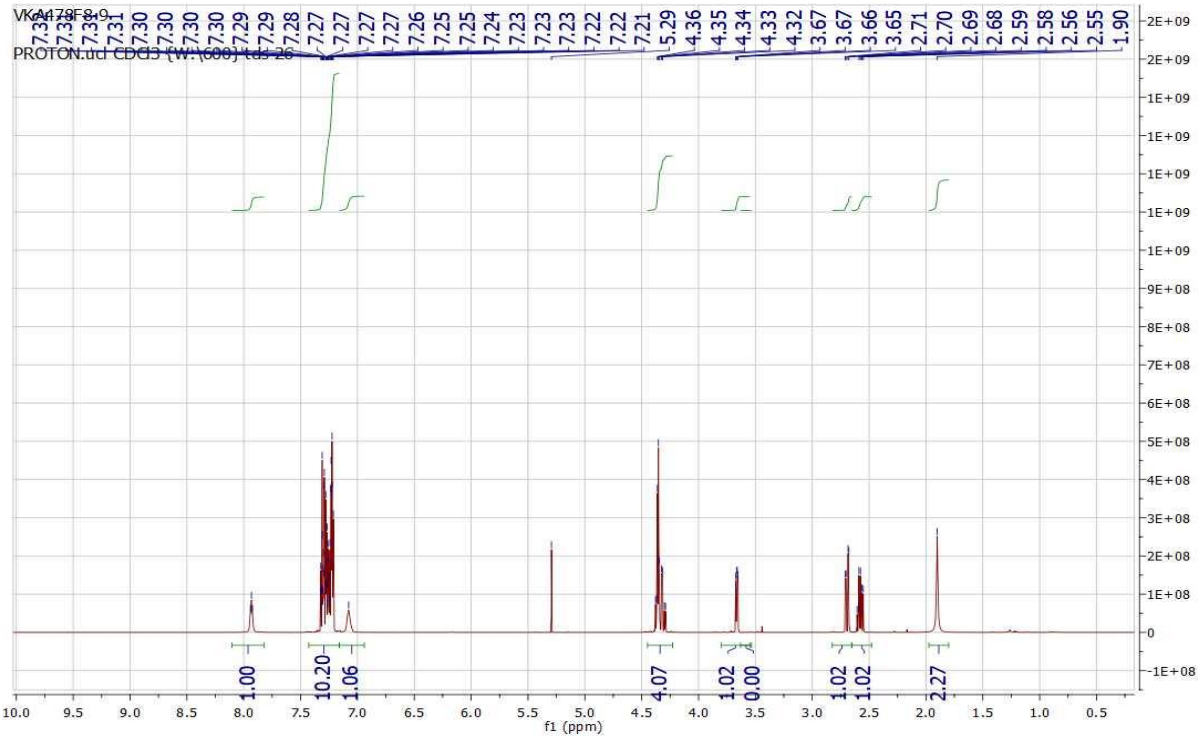

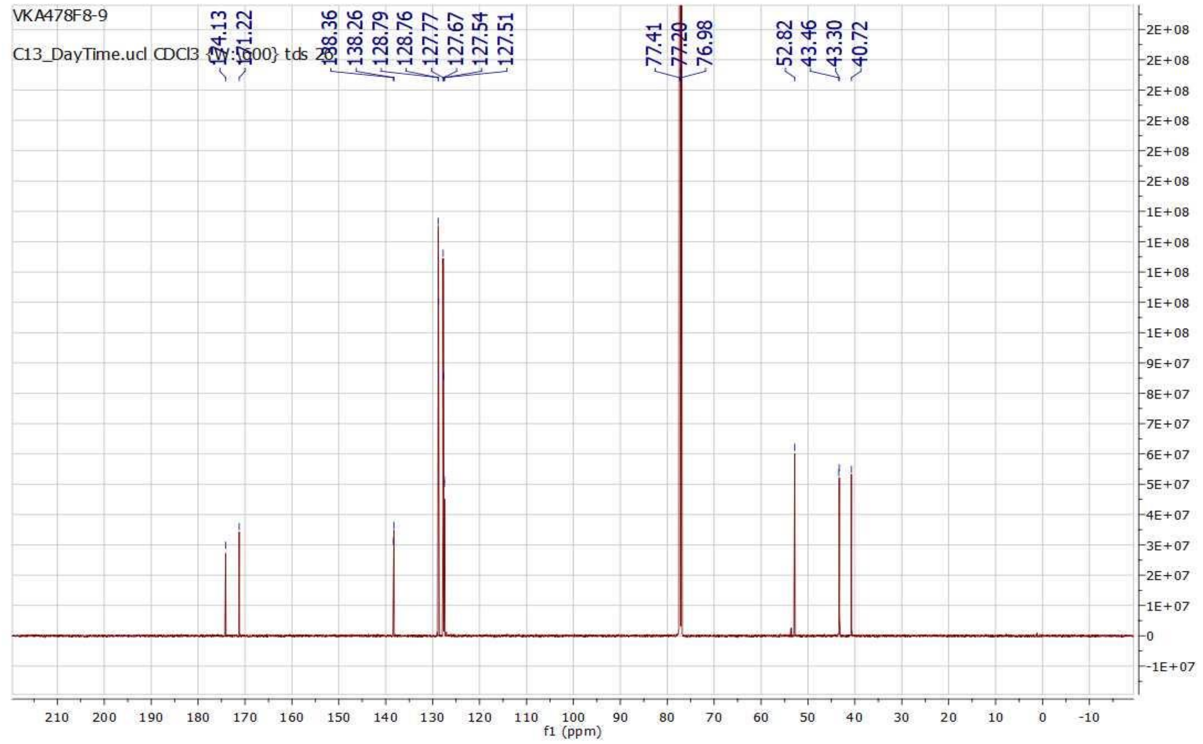

(*S*)-2-Amino-*N*-benzyl-3-(1*H*-imidazol-4-yl)propenamide (1r)

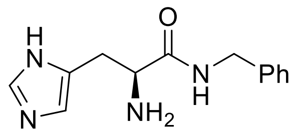

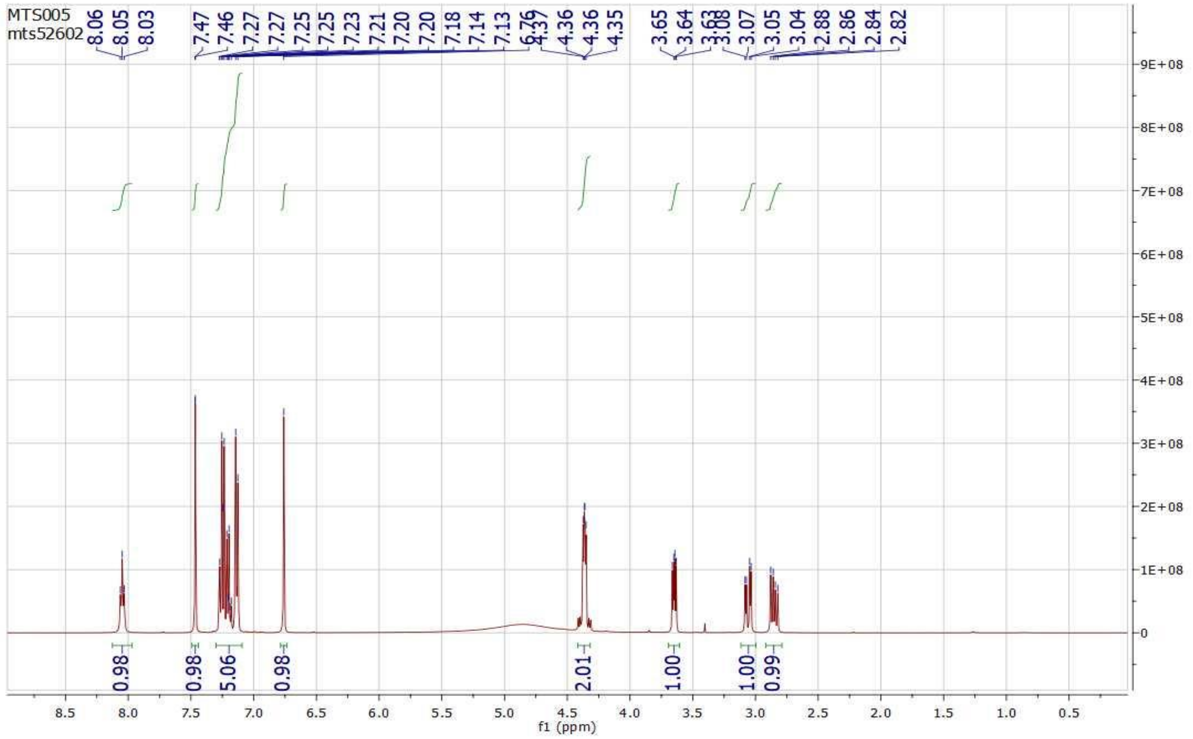

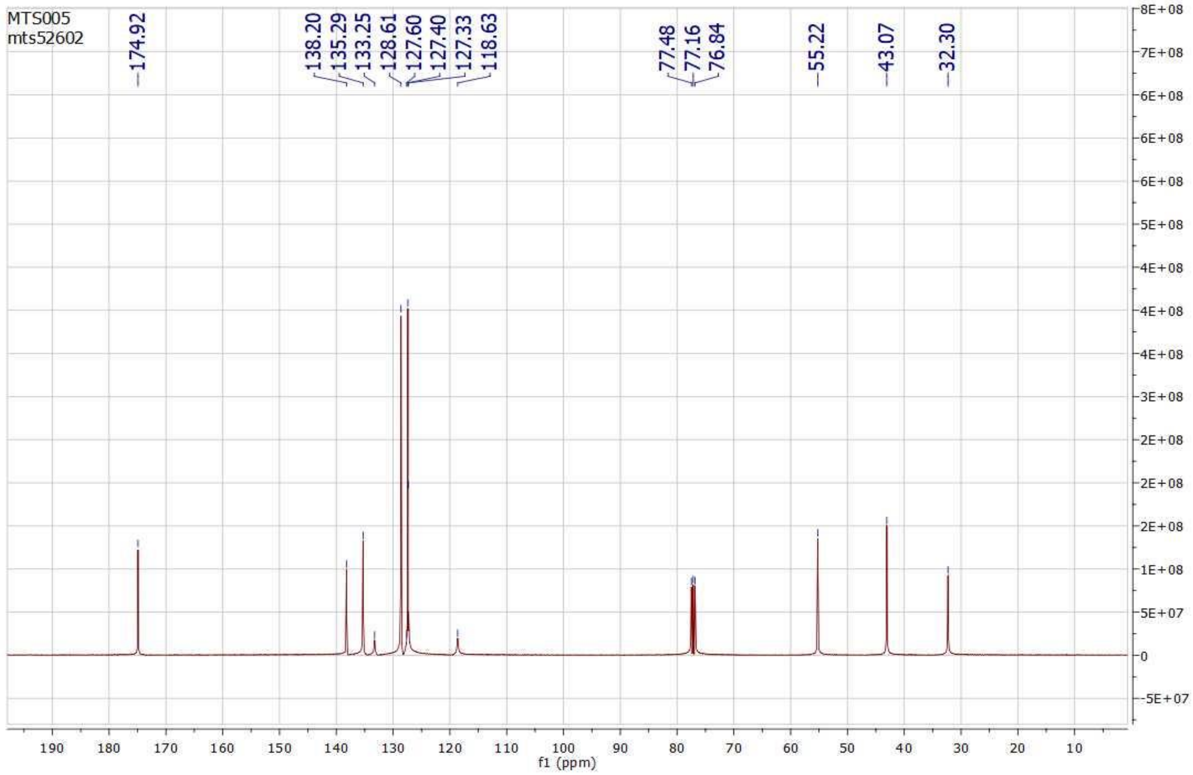

3-Amino-*N*-benzyl-3-phenylpropanamide (1x)

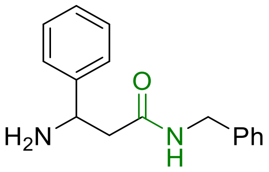

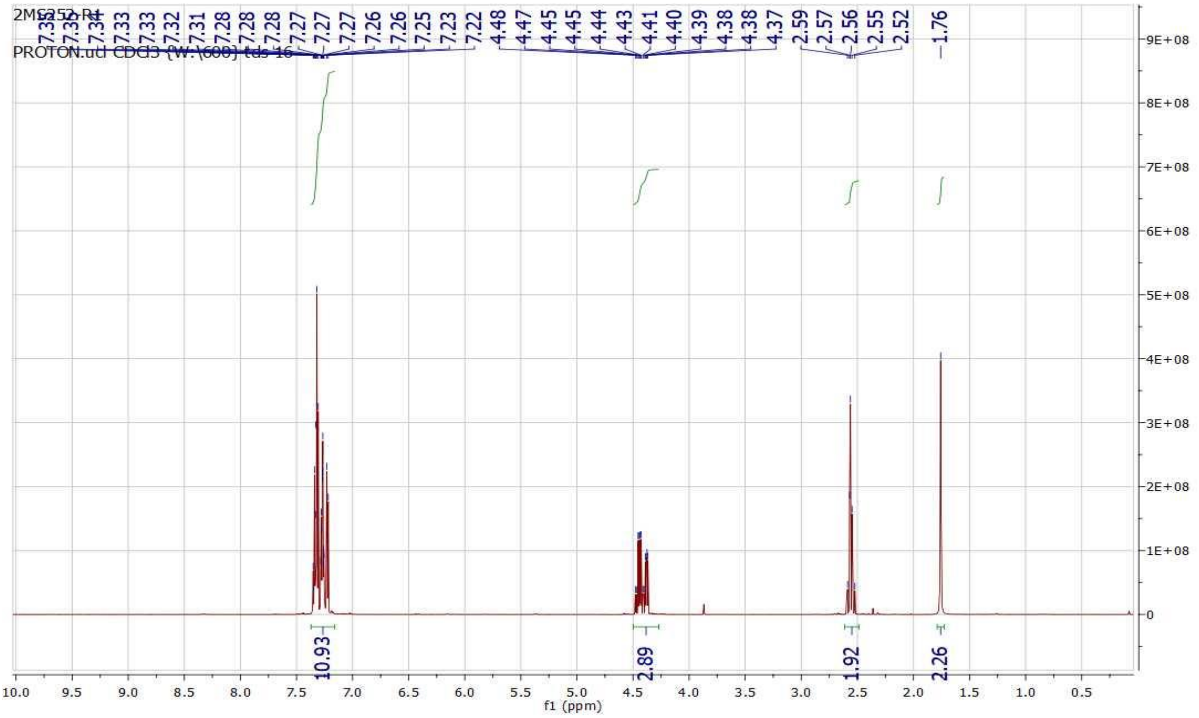

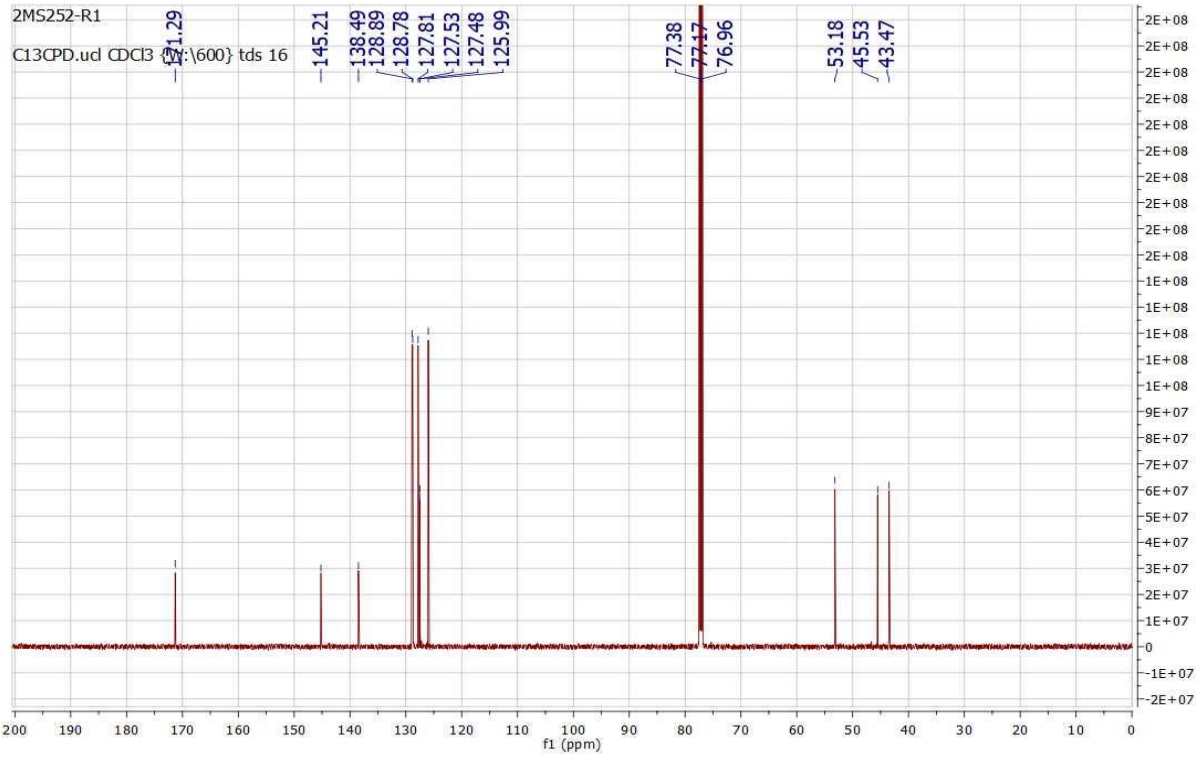

tert-Butyl (*S*)-3-amino-4-(benzylamino)-4-oxobutanoate (1y)

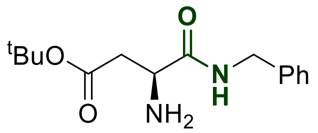

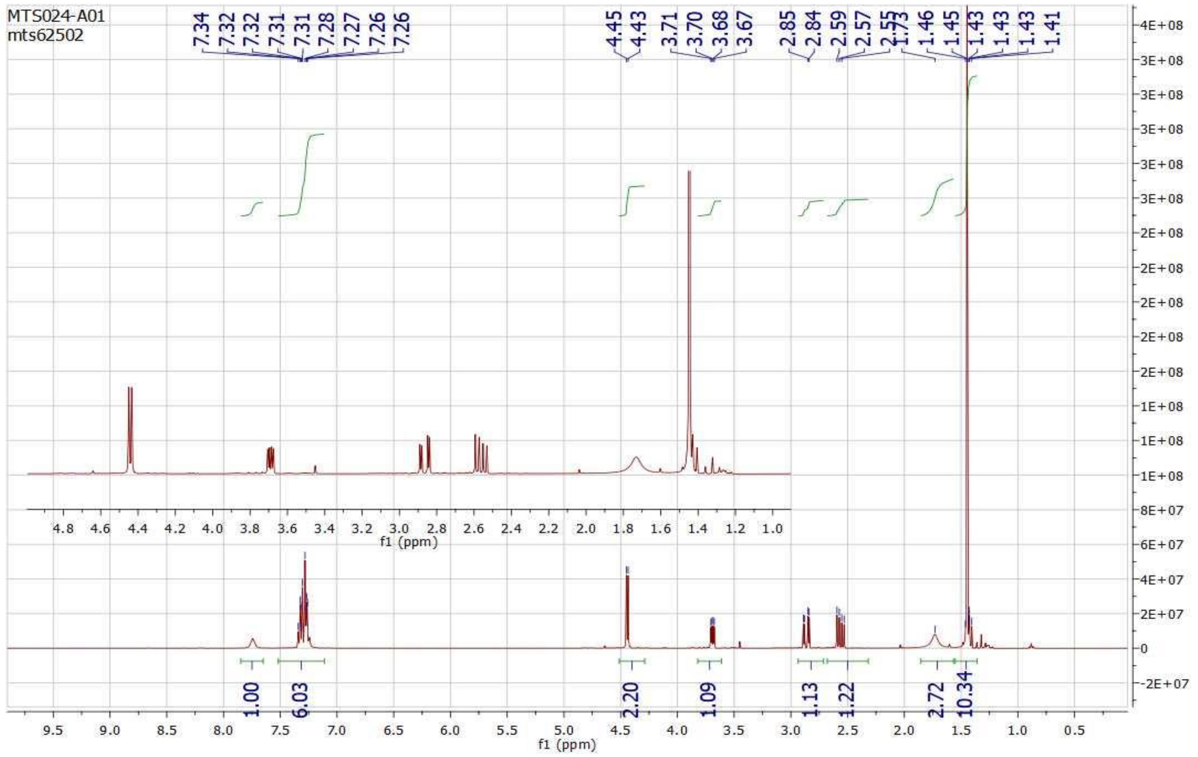

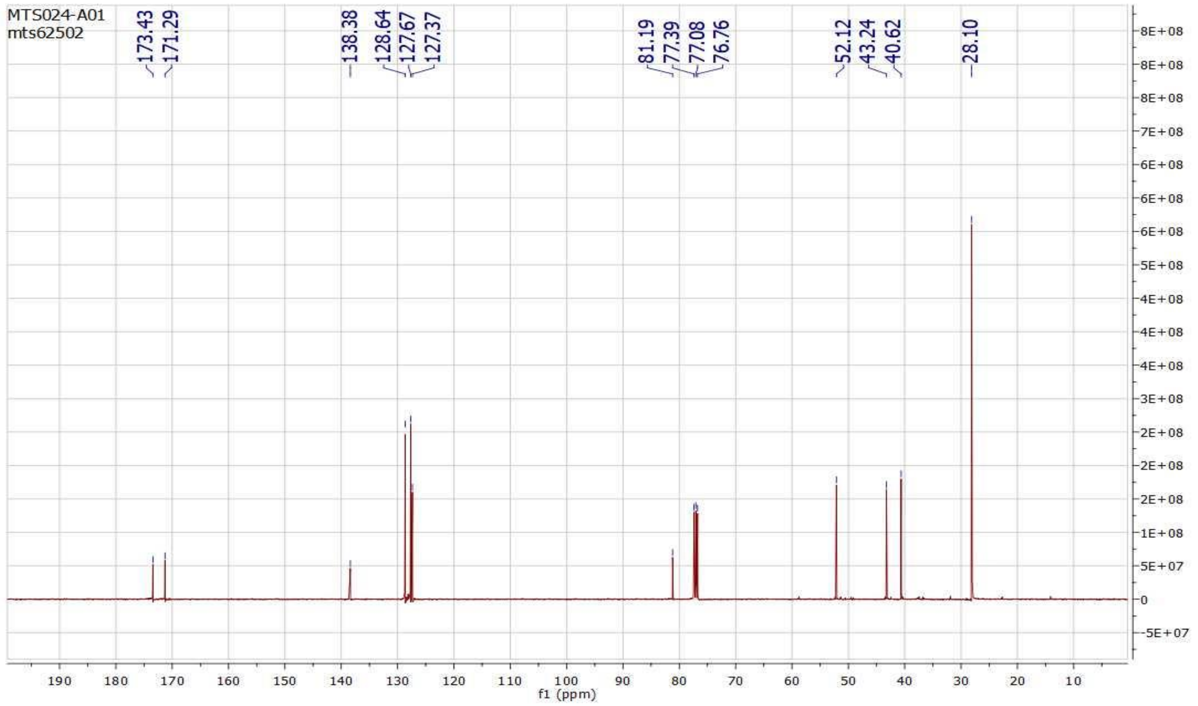

(*S*)-2-Amino-*N*-benzyl-3-methoxypropanamide (1z)

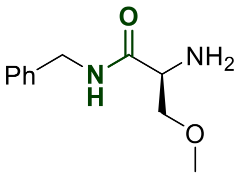

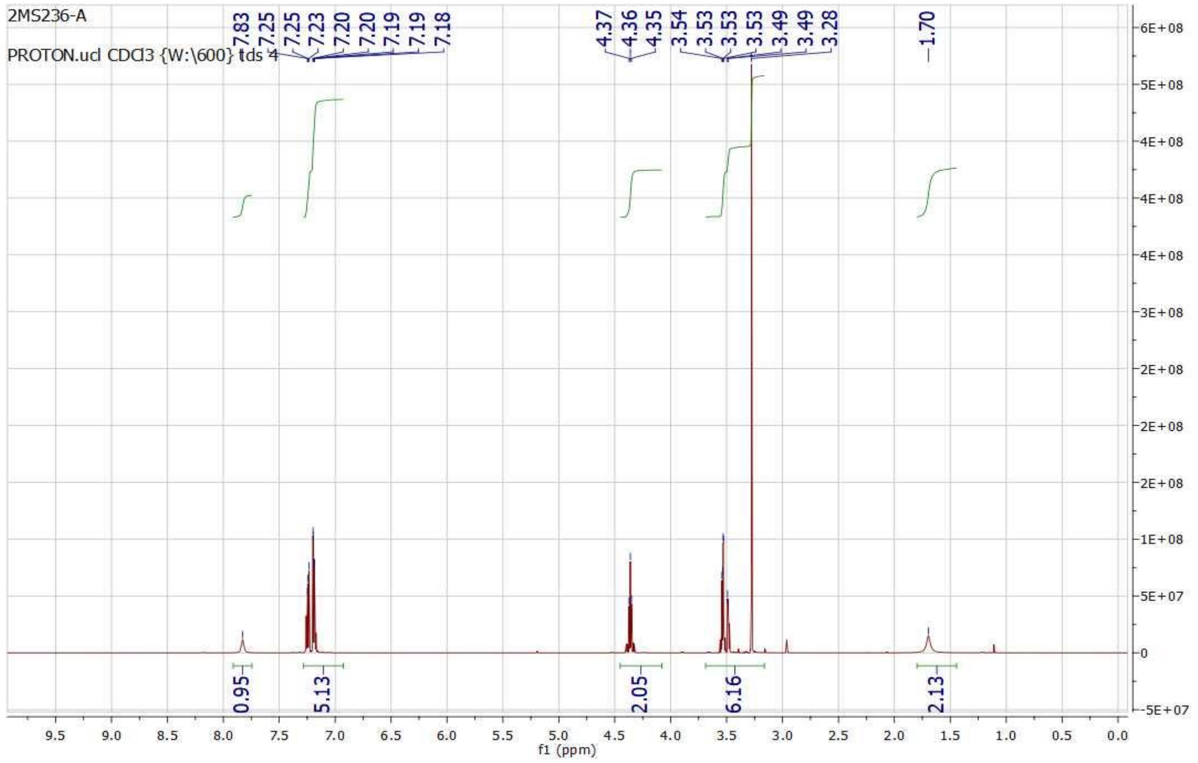

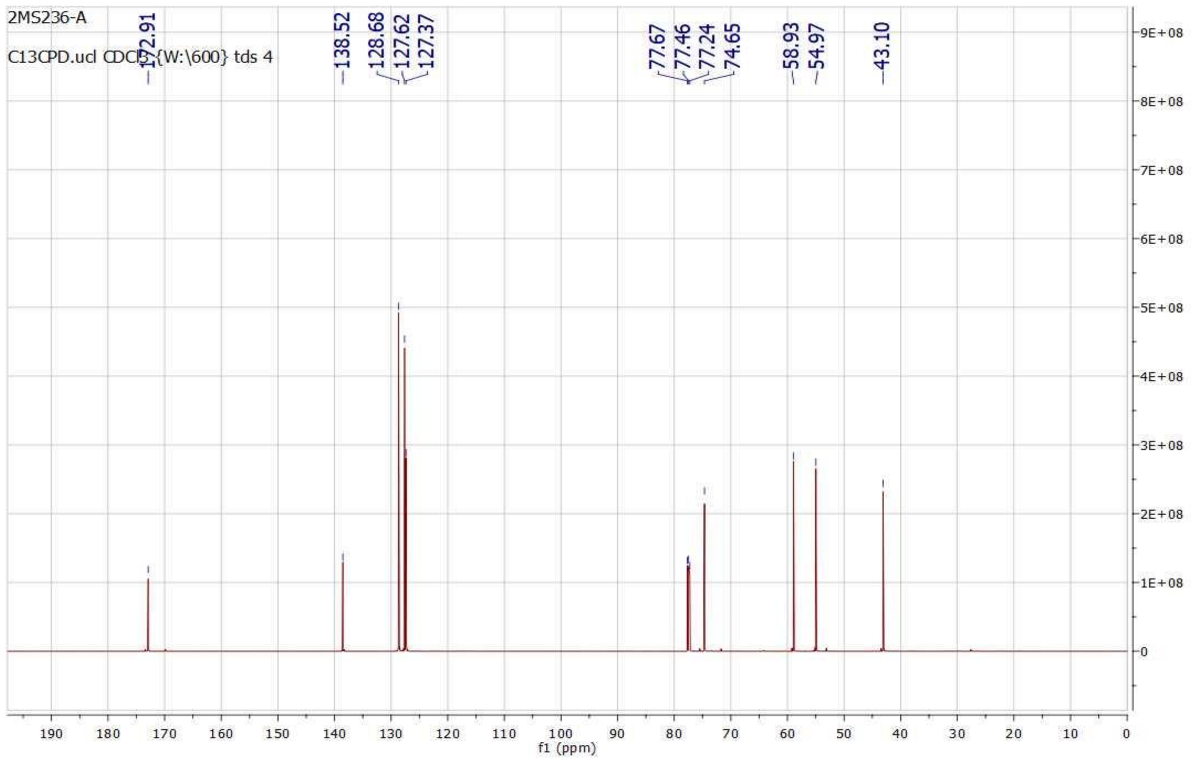

***N*-benzylthiazolidine-4-carboxamide (1aa)**

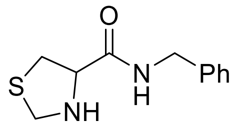

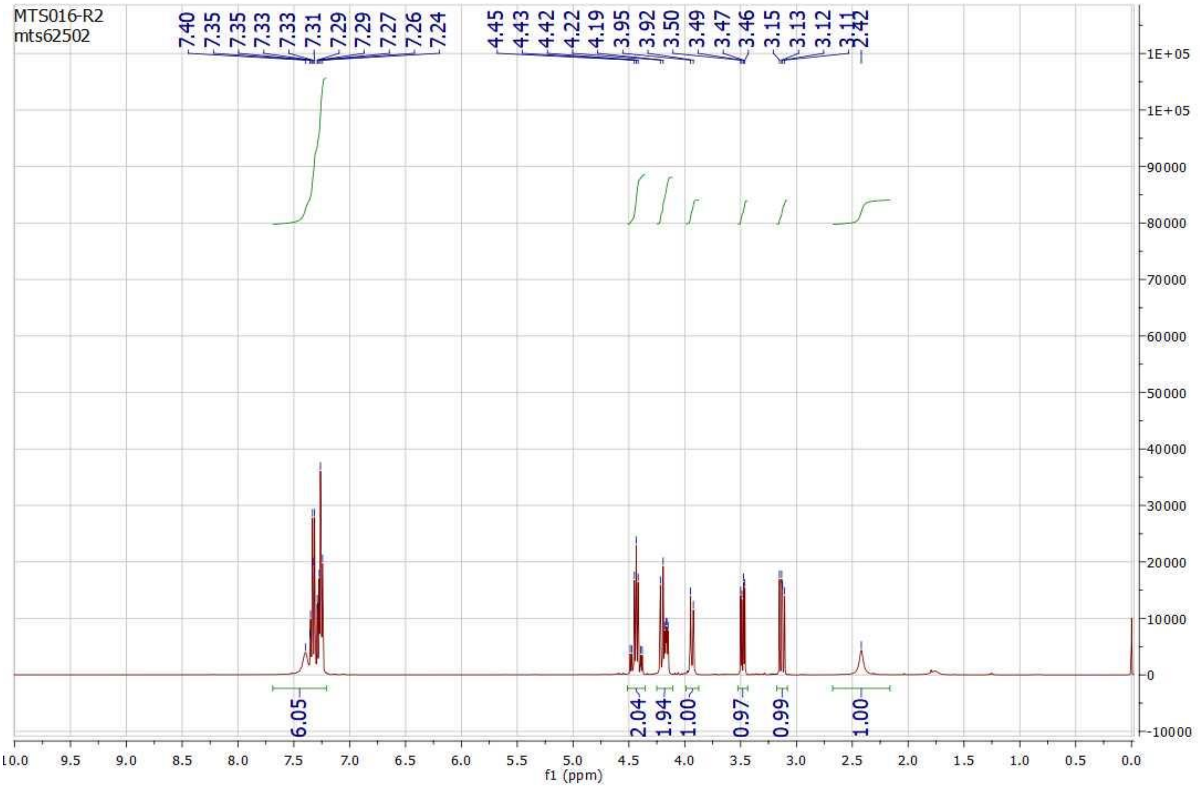

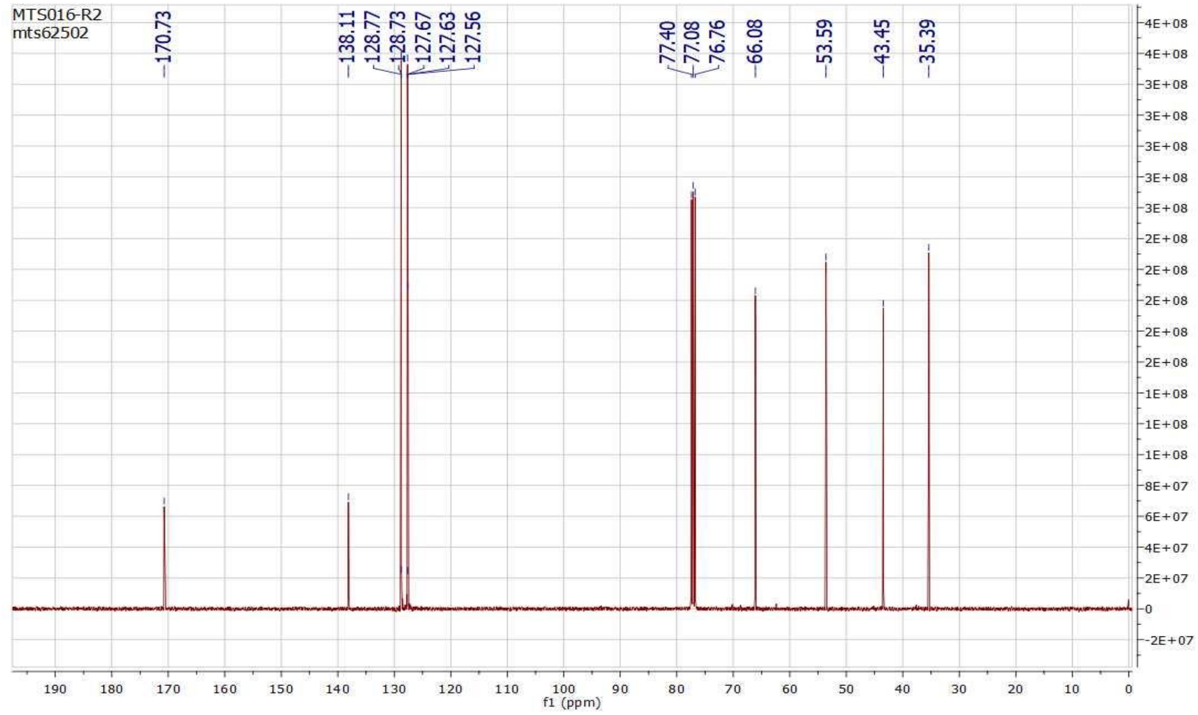

5-Amino-3-benzyl-1,3-thiazinan-4-one (SI.1ab)

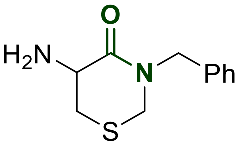

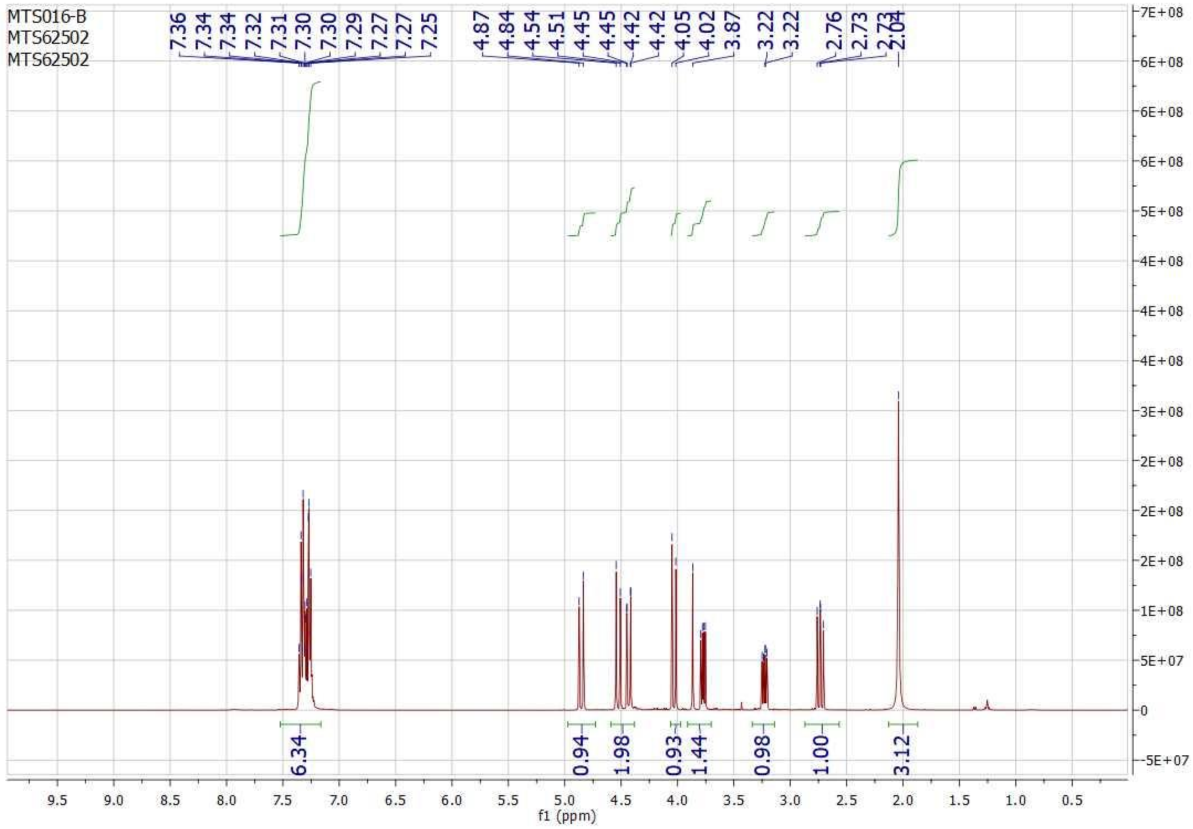

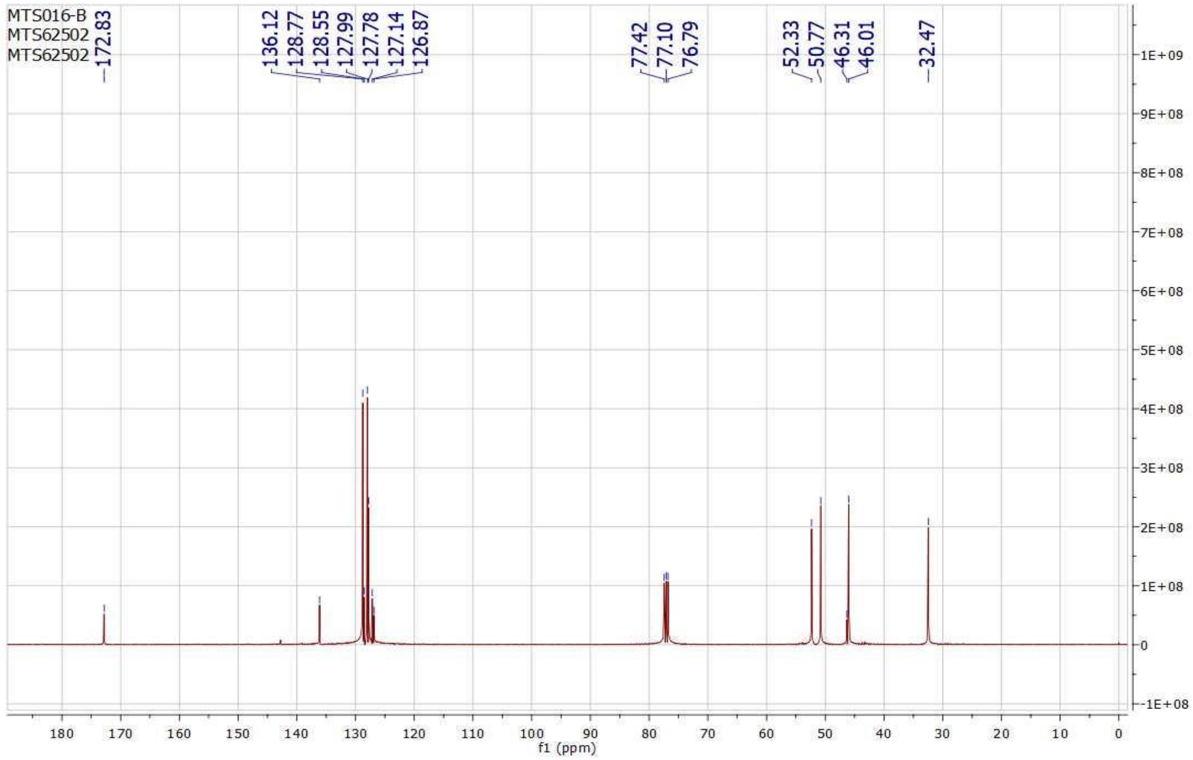

(*R*)-2-Amino-*N*-benzyl-3-(methylthio)propanamide (1bb)

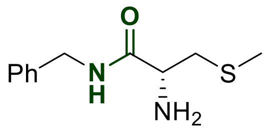

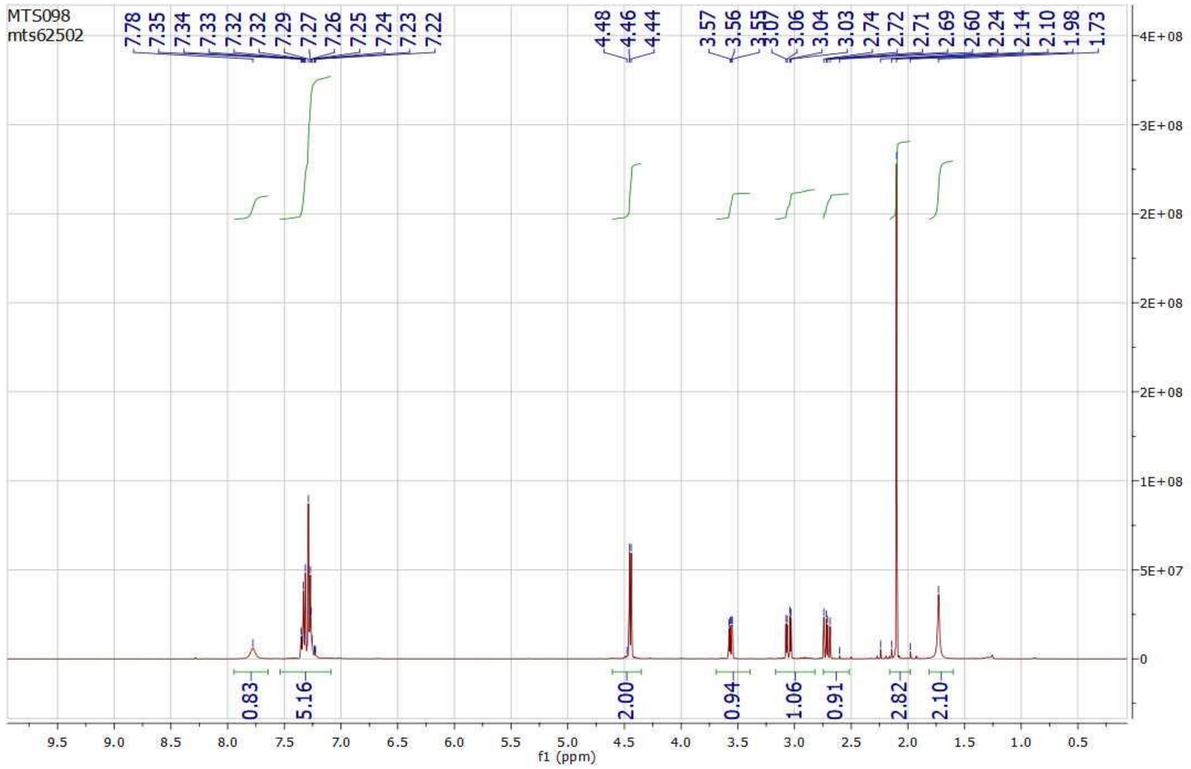

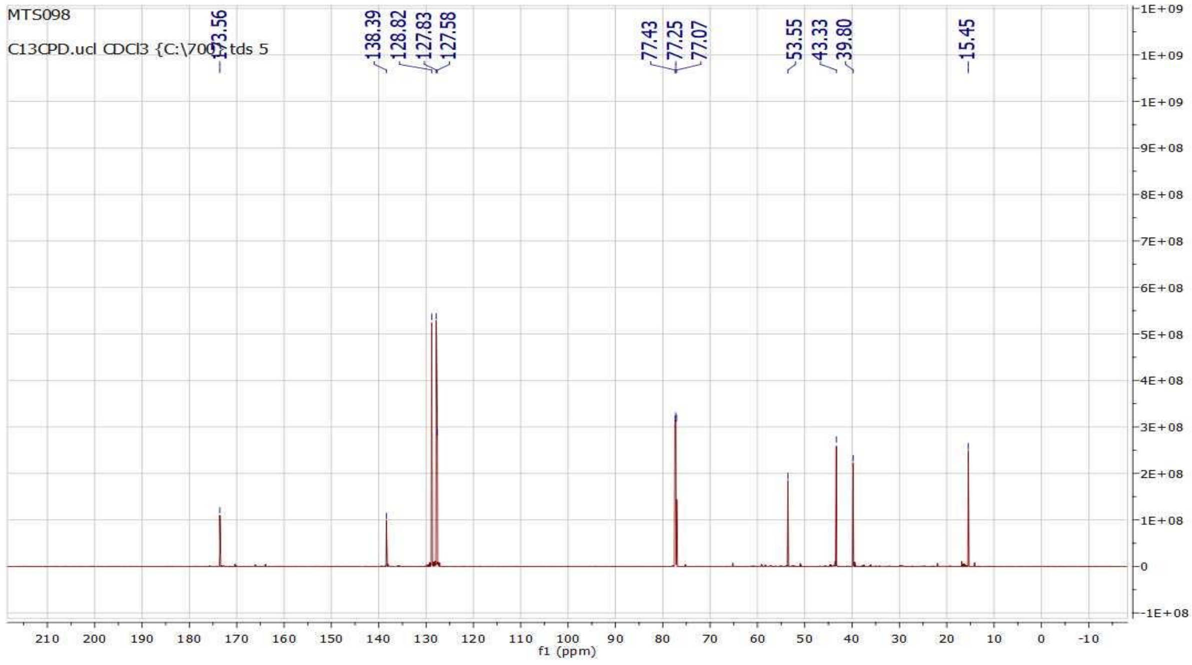

***N*-Benzyl-2-(methylamino)acetamide (1cc)**

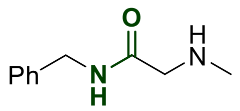

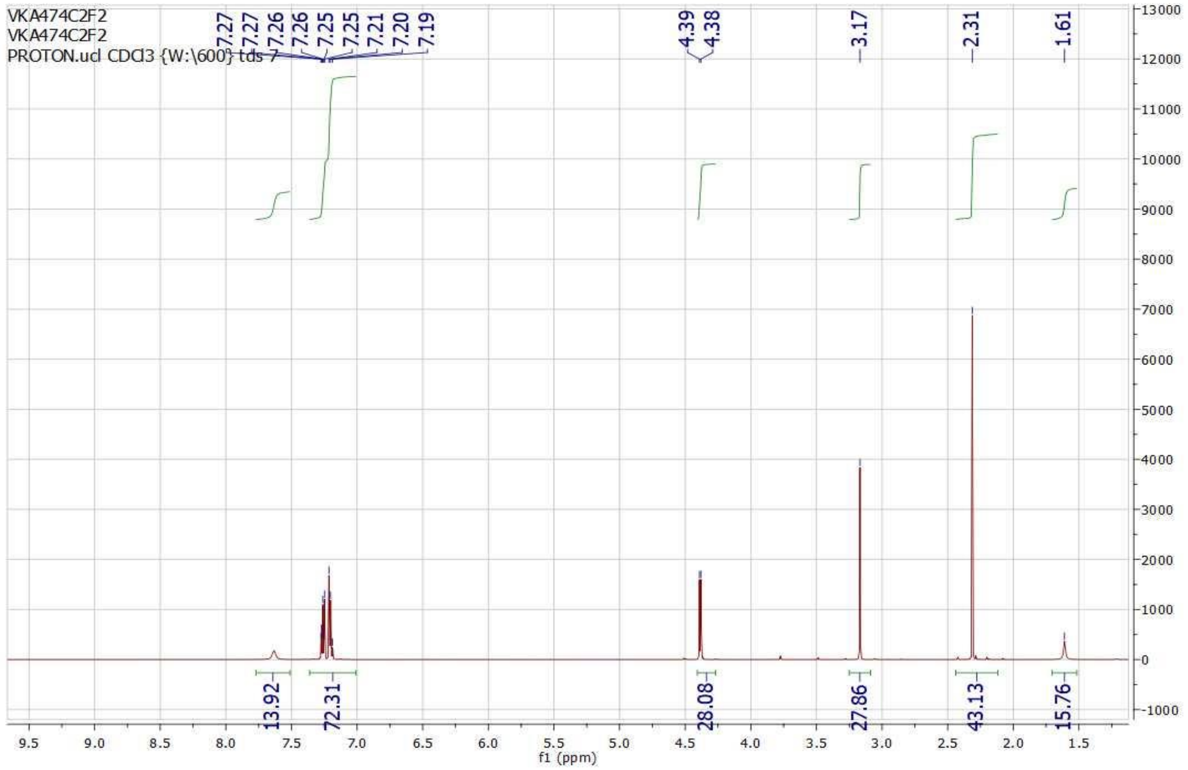

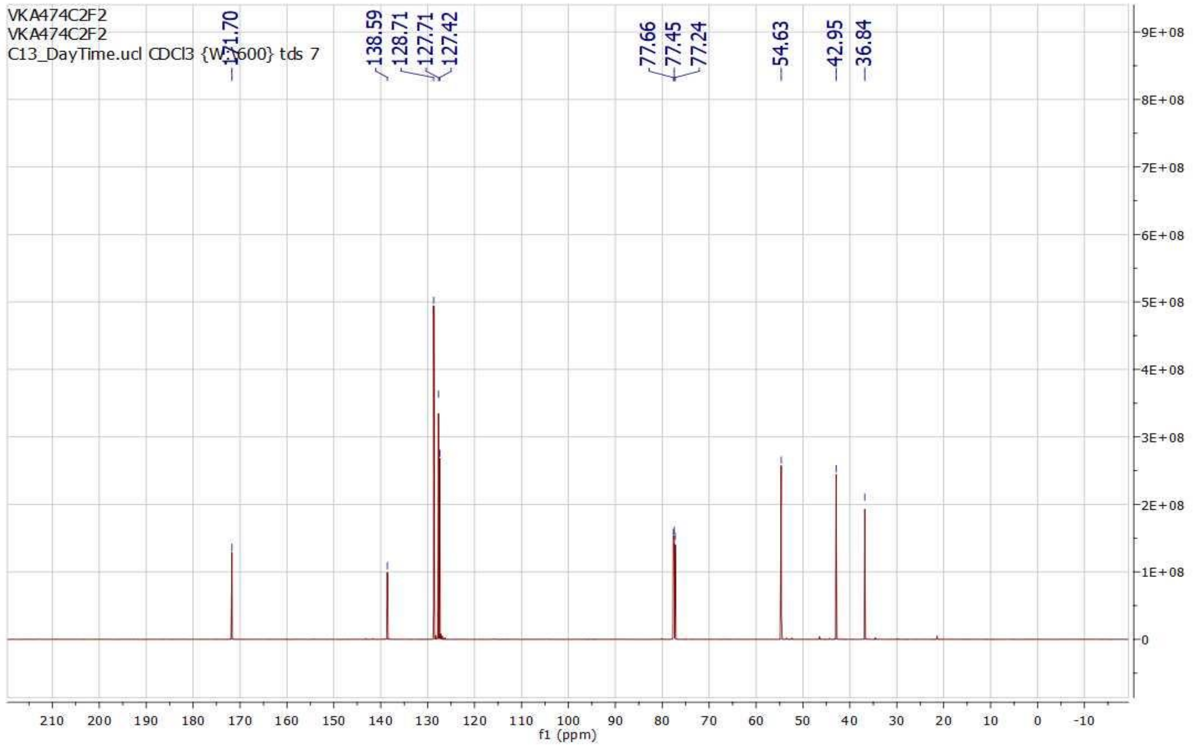

(*S*)-2-Amino-N-benzyl-2-phenylacetamide (1ee)

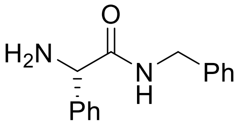

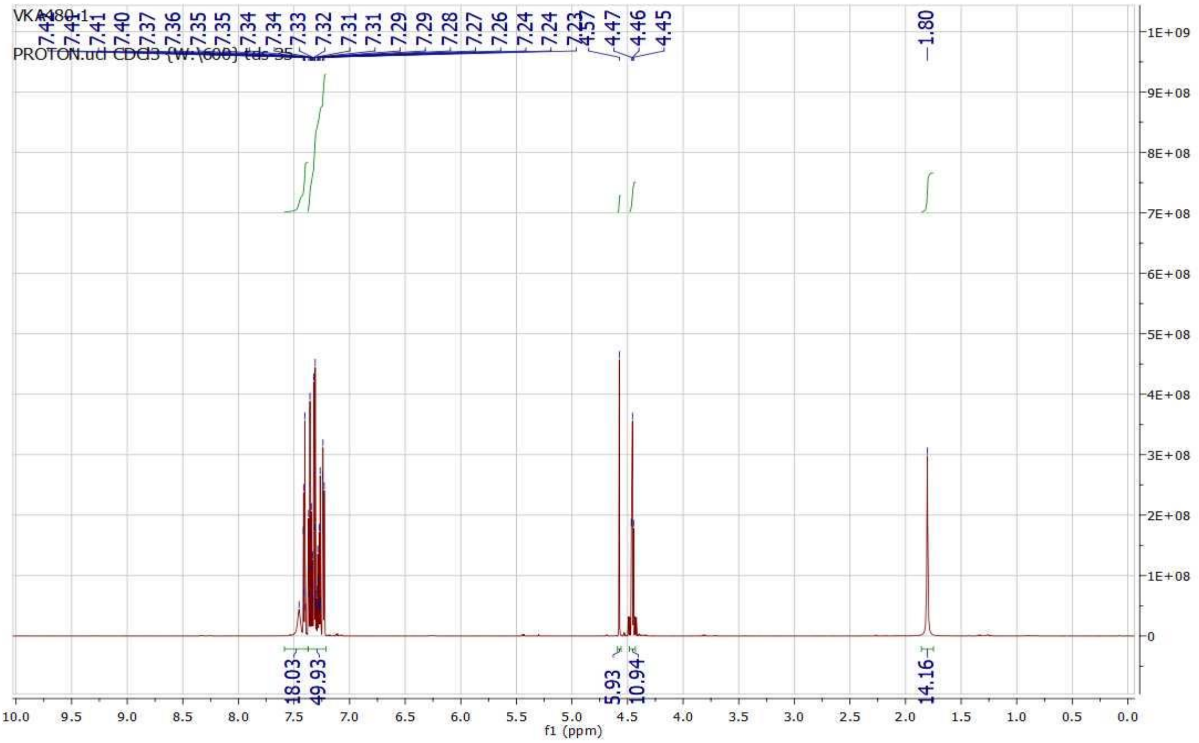

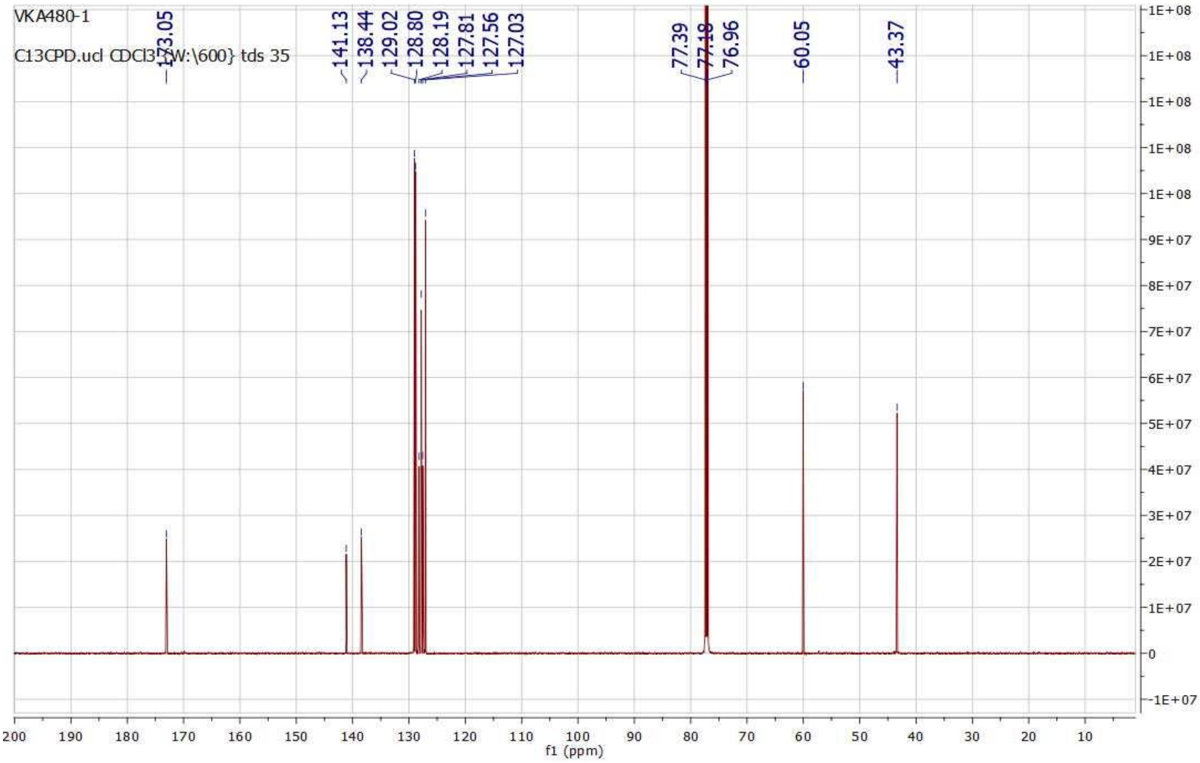

(*R*)-2-Amino-*N*-(cyclohexylmethyl)-4-(methylthio)butanamide (6g)

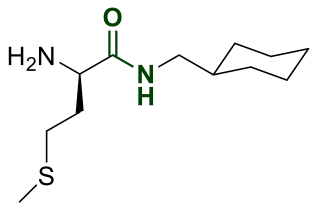

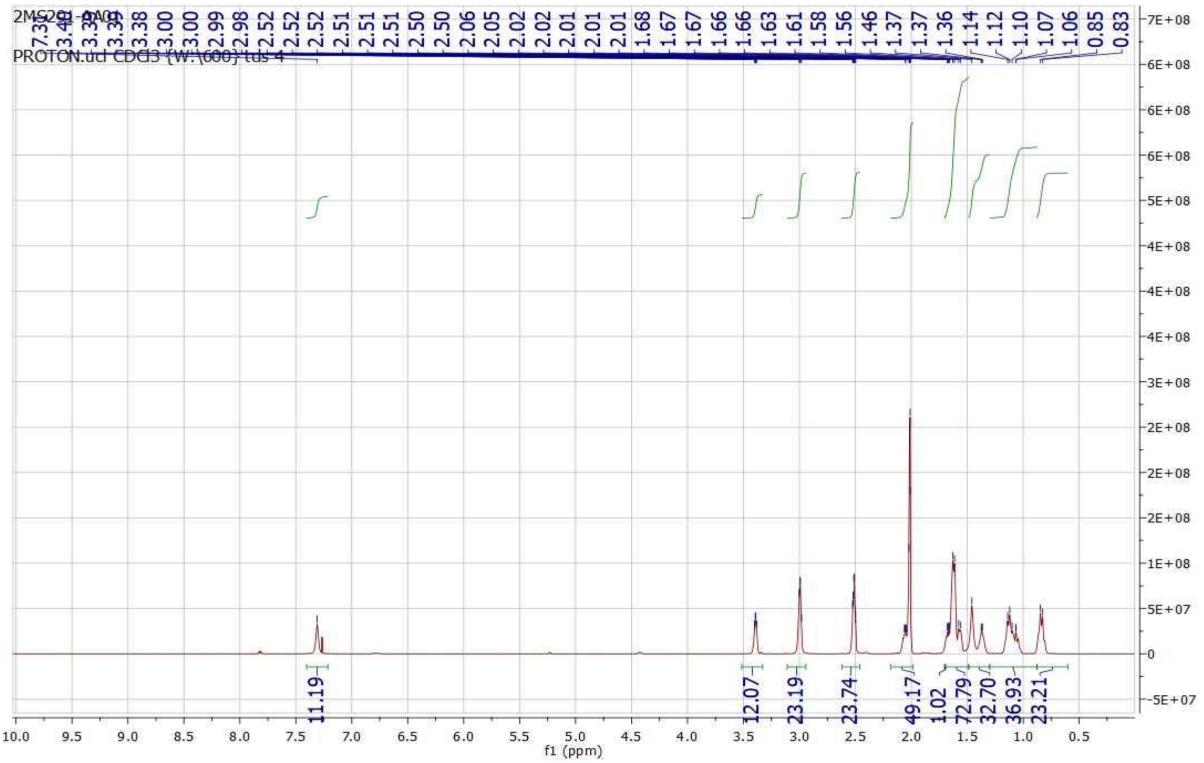

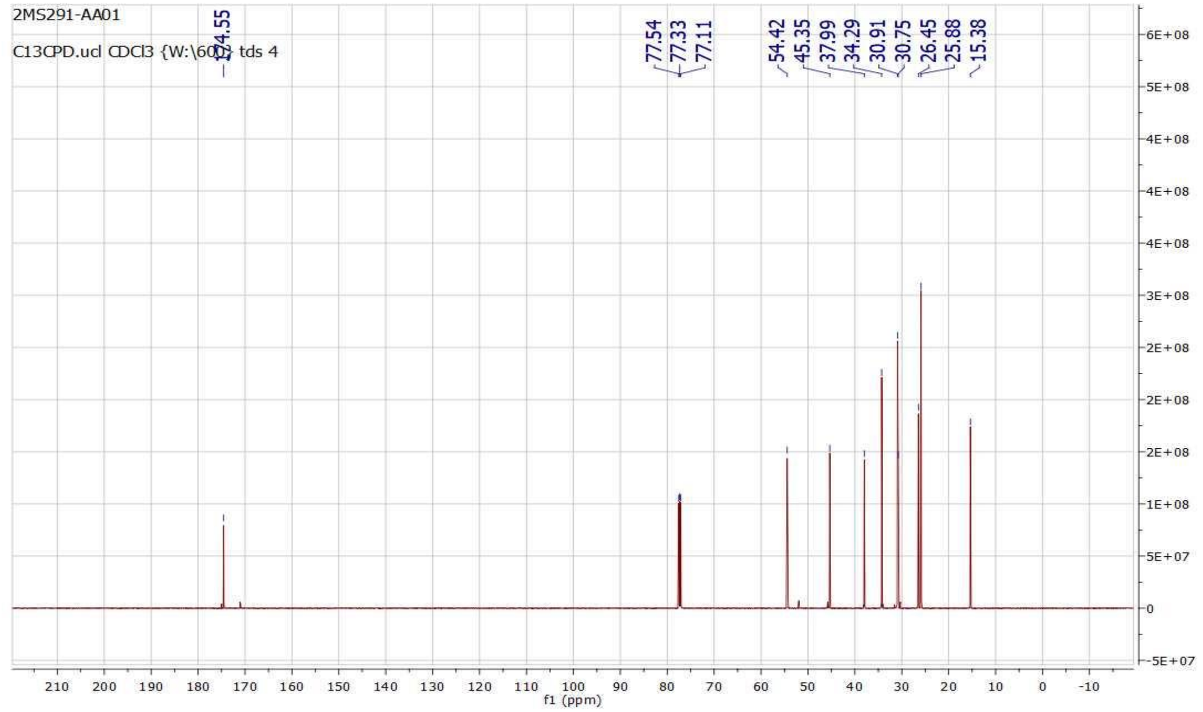

(*S*)-2-Amino-*N*-cyclohexyl-3-phenylpropanamide (6h)

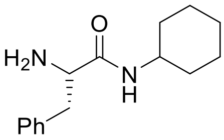

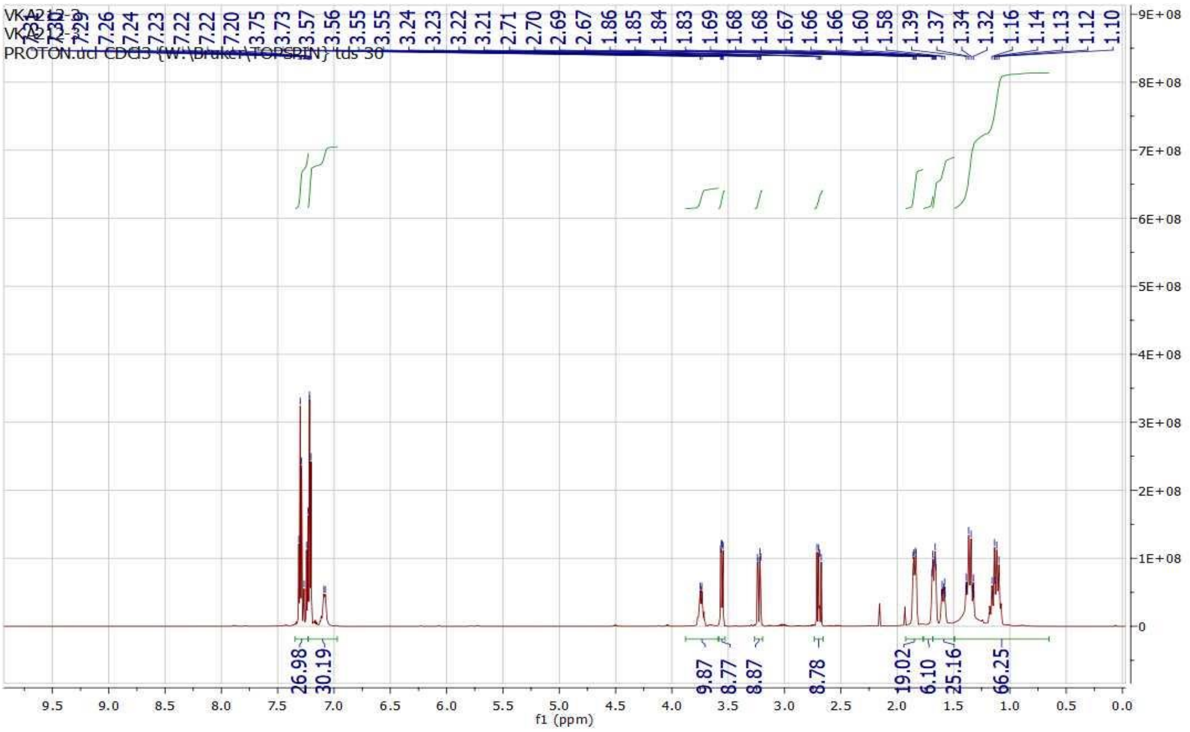

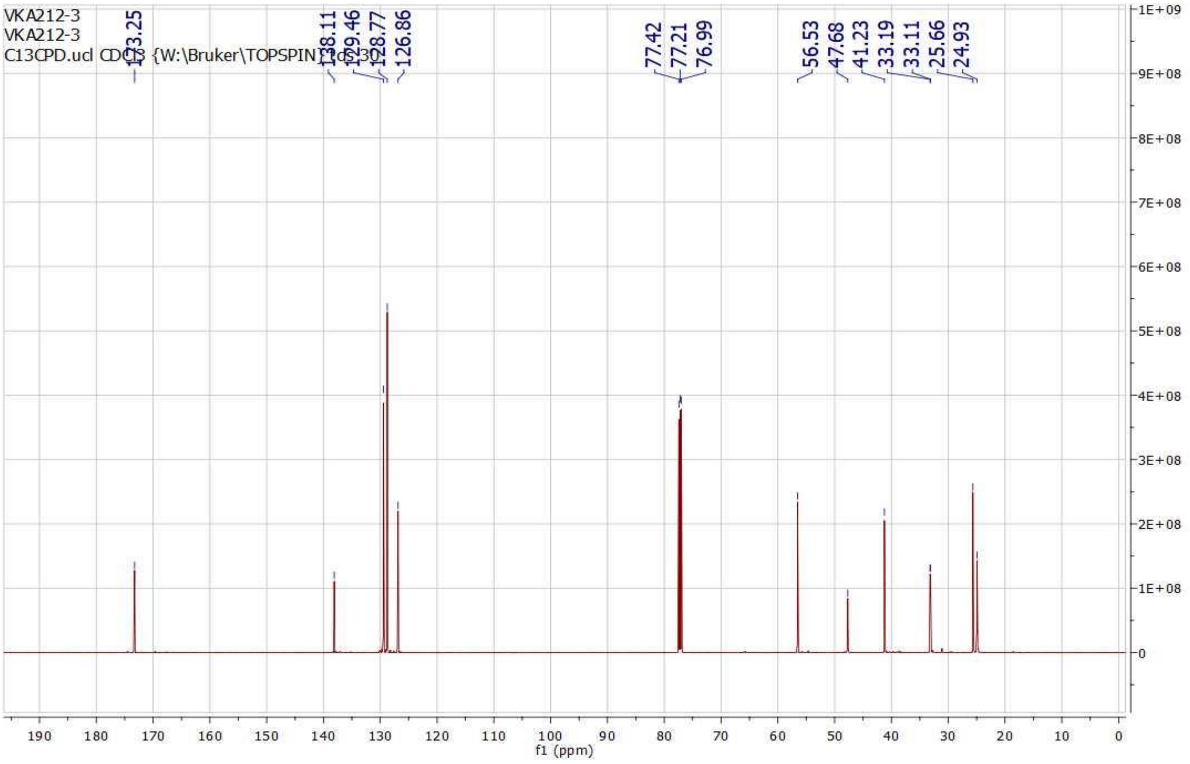

(S)-2-Amino-N-(4-methoxybenzyl)-3-phenylpropanamide (6i)

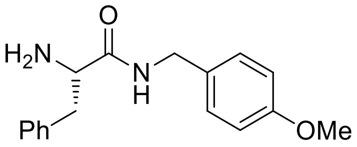

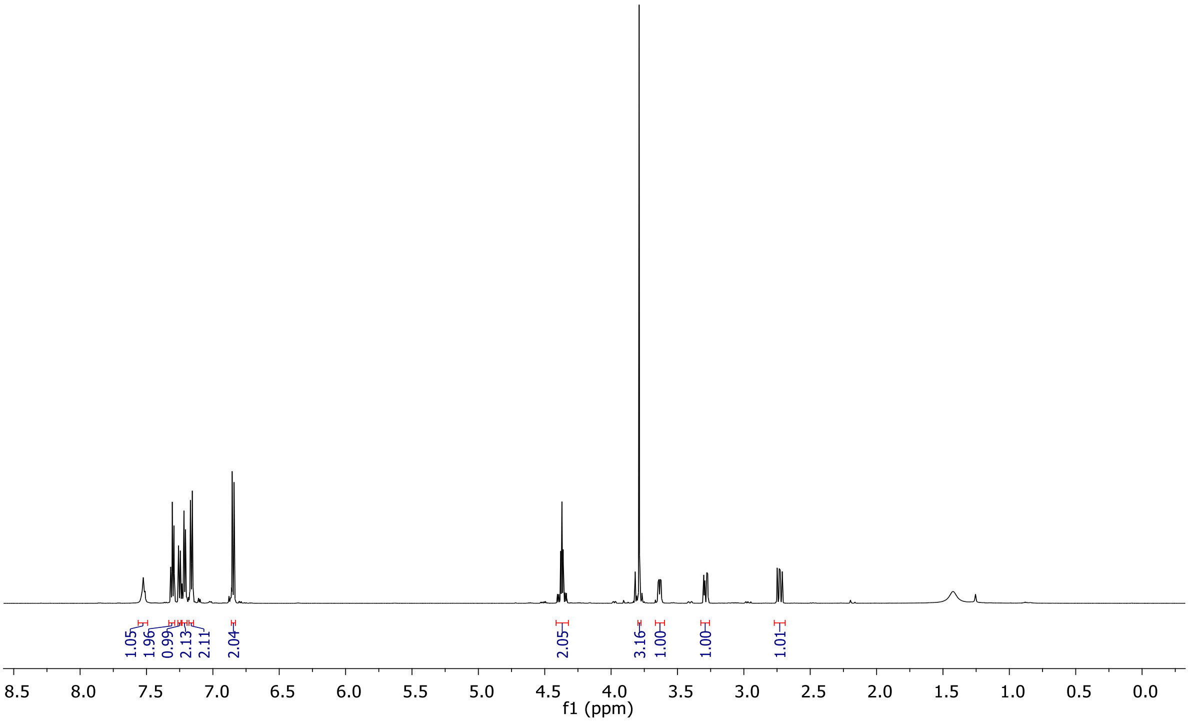

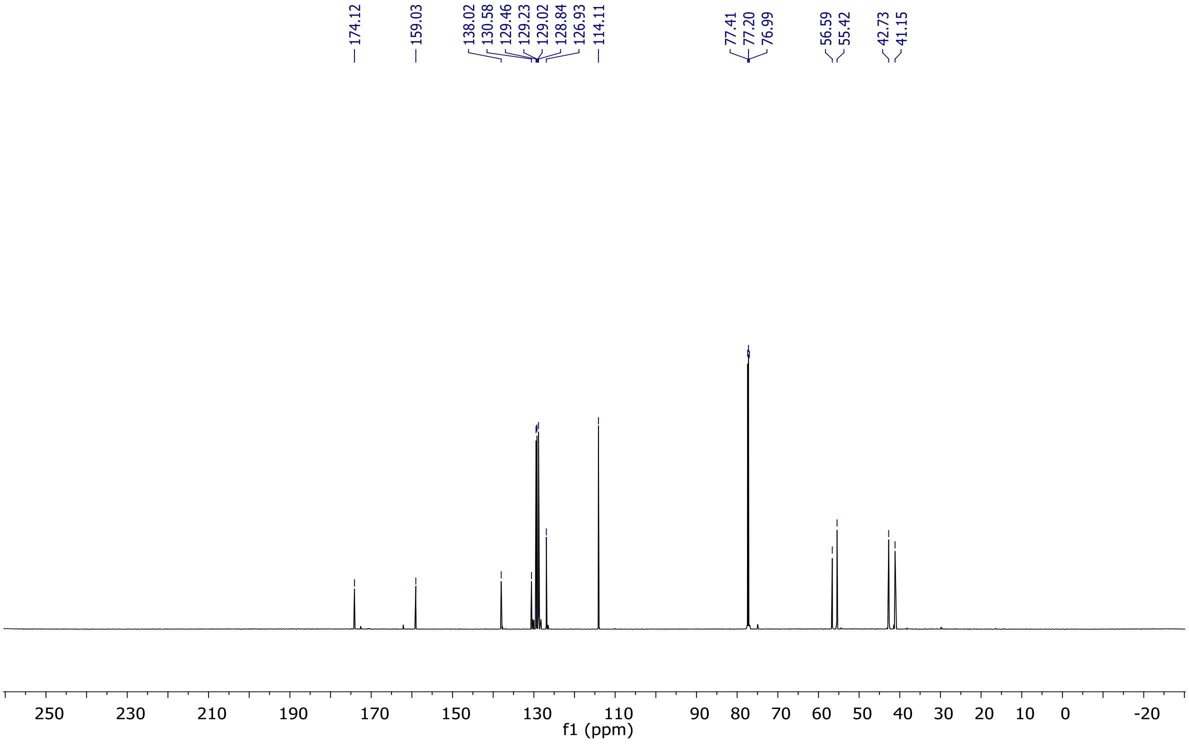

(S)-2-Amino-N-(4-fluorobenzyl)-3-phenylpropanamide (6j)

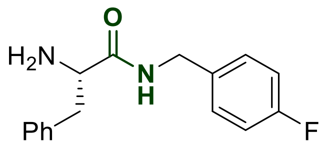

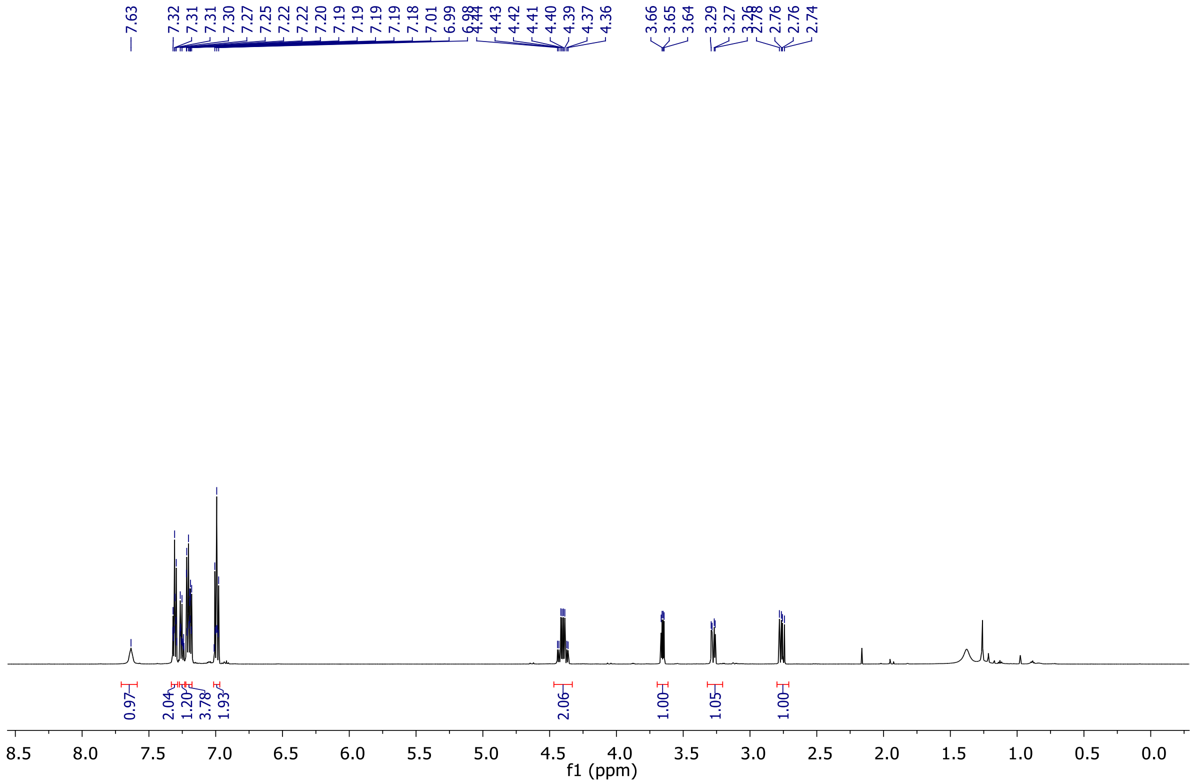

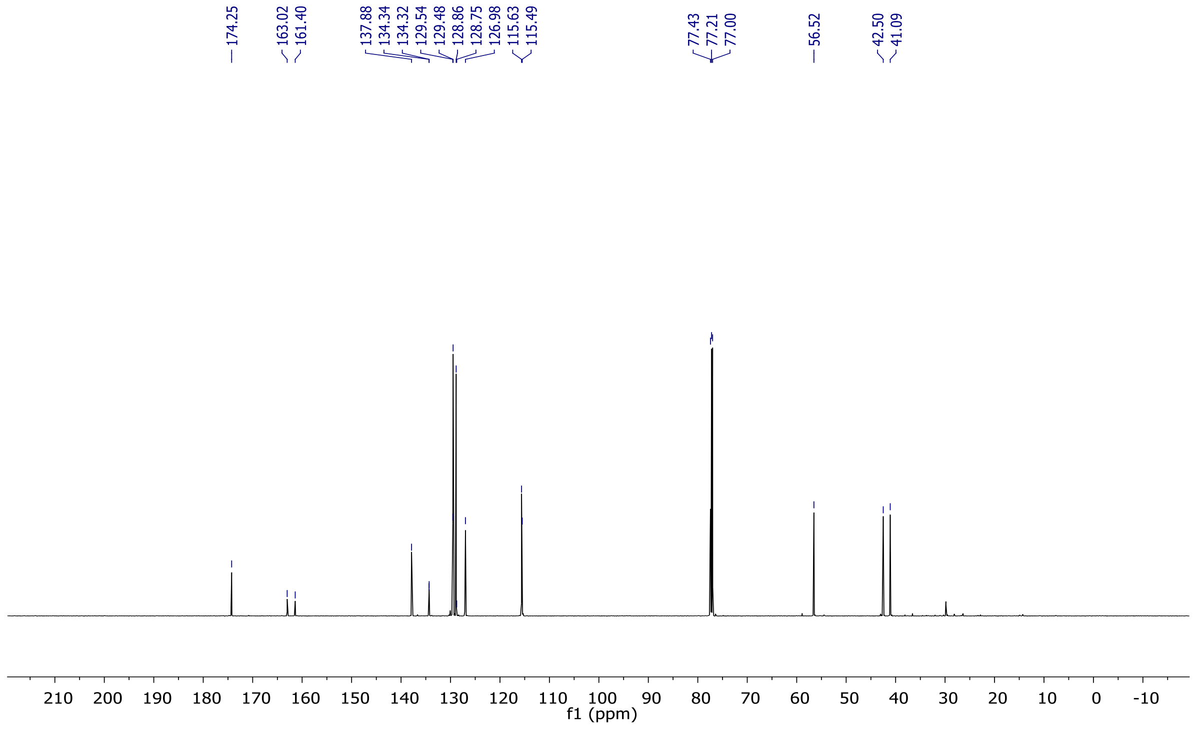

(*S*)-*N*-(4-Methoxybenzyl)pyrrolidine-2-carboxamide (6l)

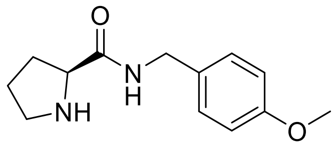

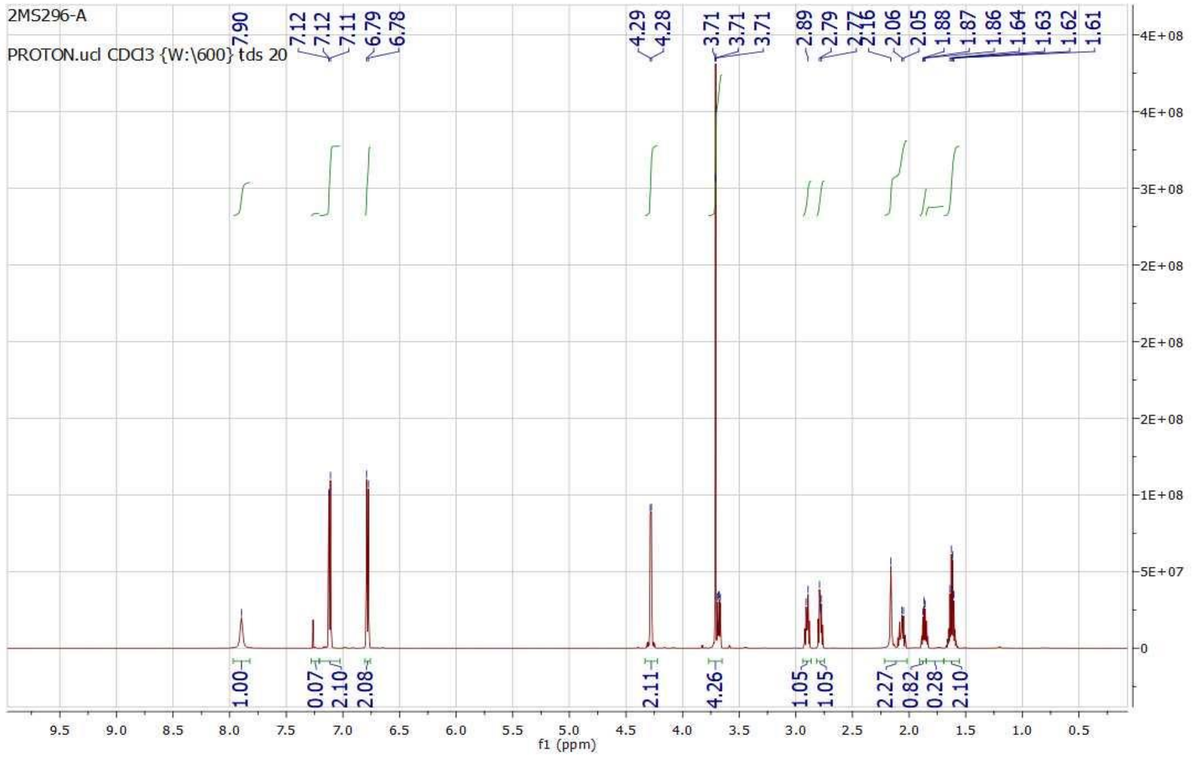

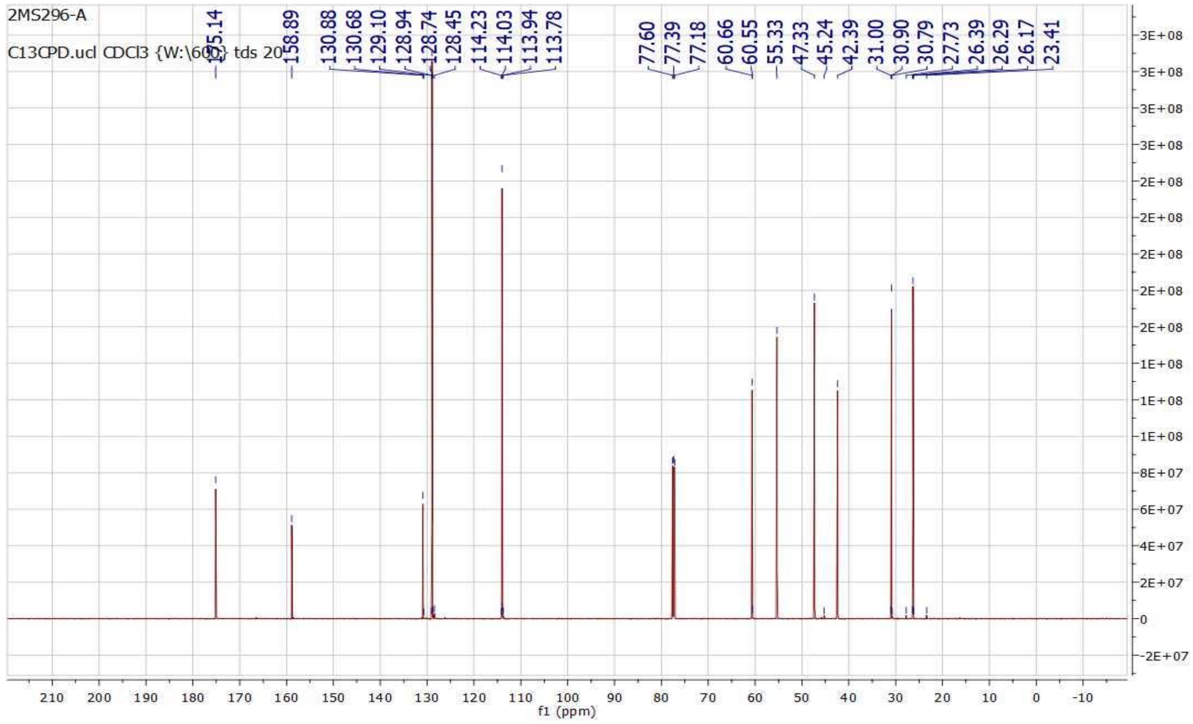

(*S*)-*N*-(2-(1*H*-Indol-3-yl)ethyl)-2-aminopropanamide (6m)

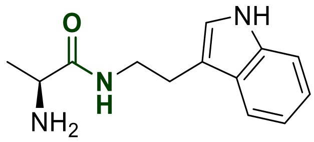

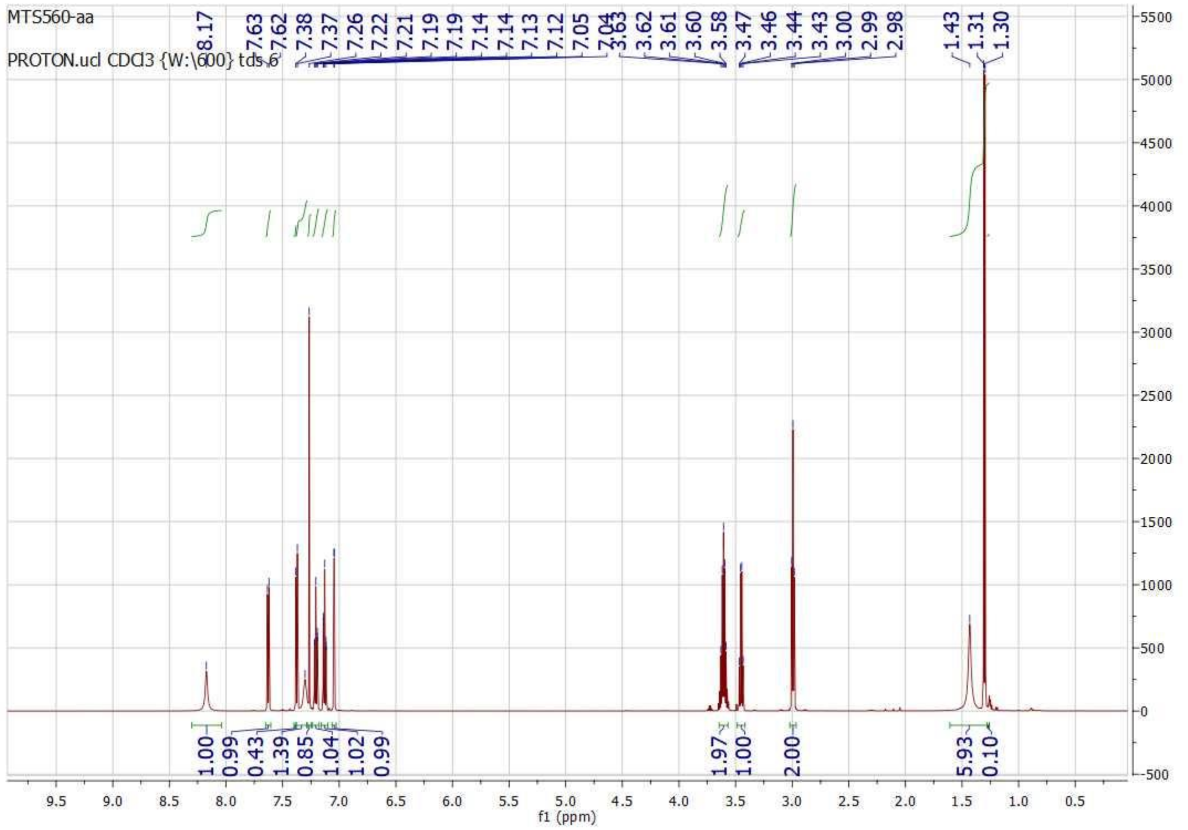

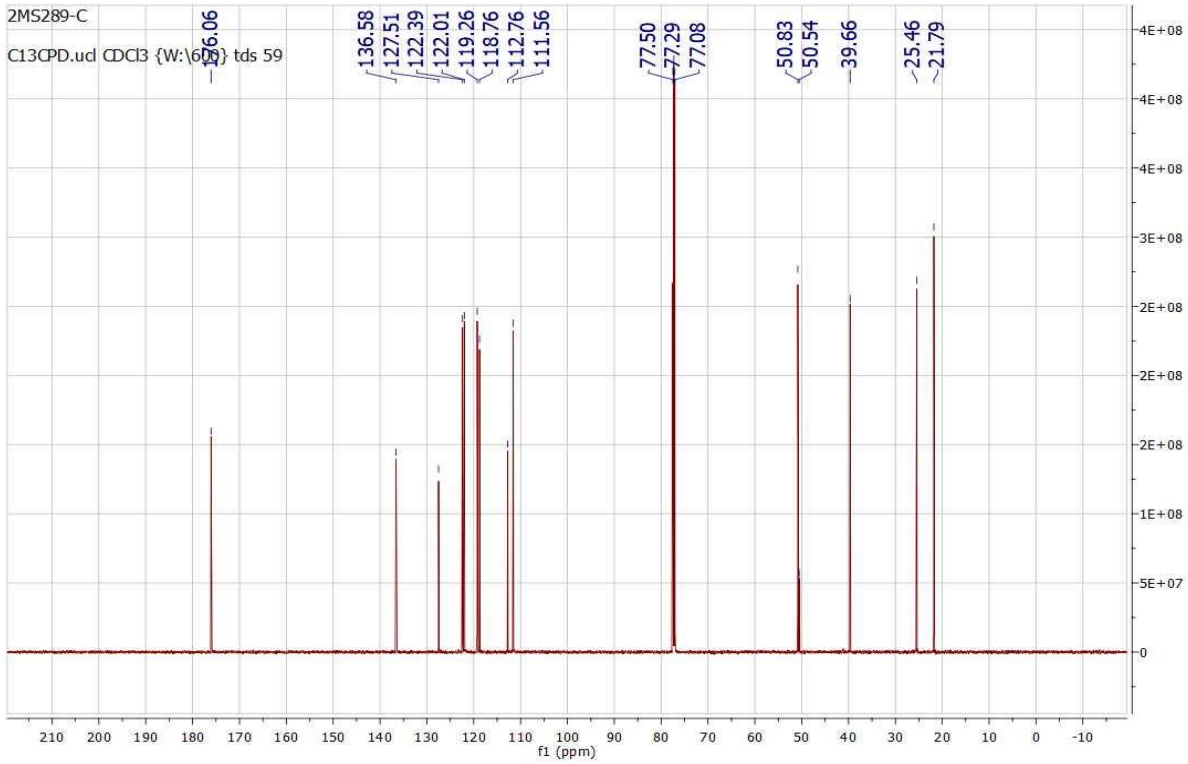

(*S*)-2-Amino-*N*-(4-(3,3,4,4,5,5,6,6,7,7,8,8,9,9,10,10,10-heptadecafluorodecyl)benzyl)-3-phenylpropanamide (6n)

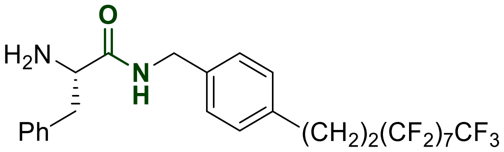

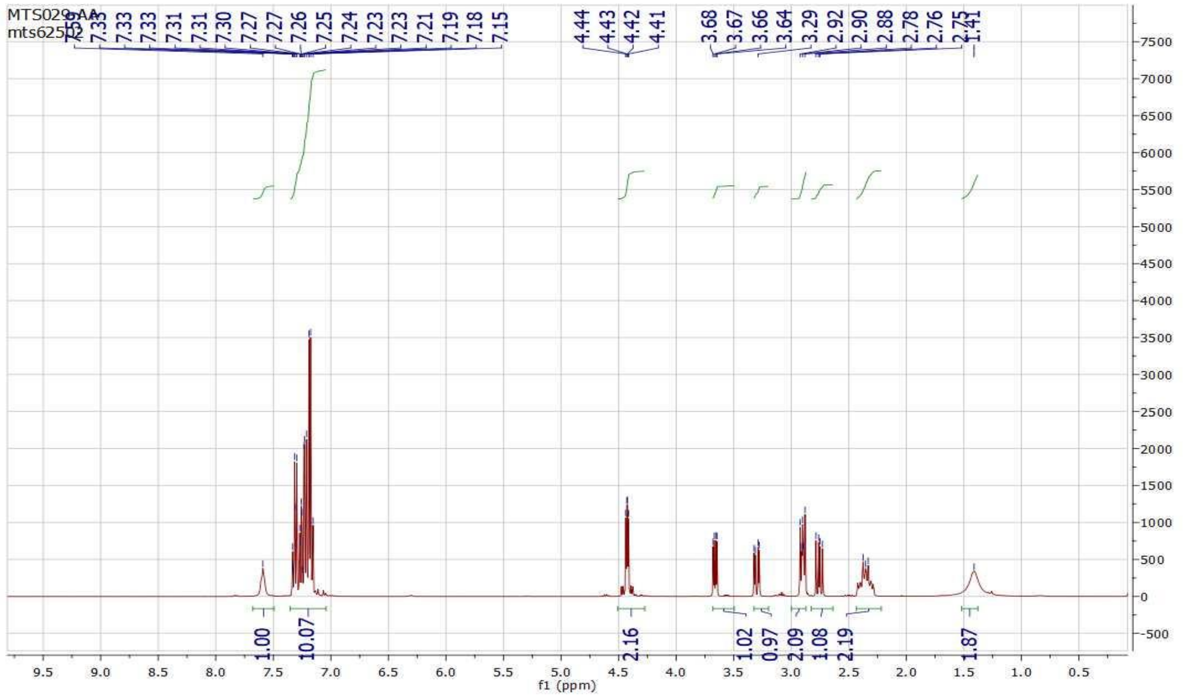

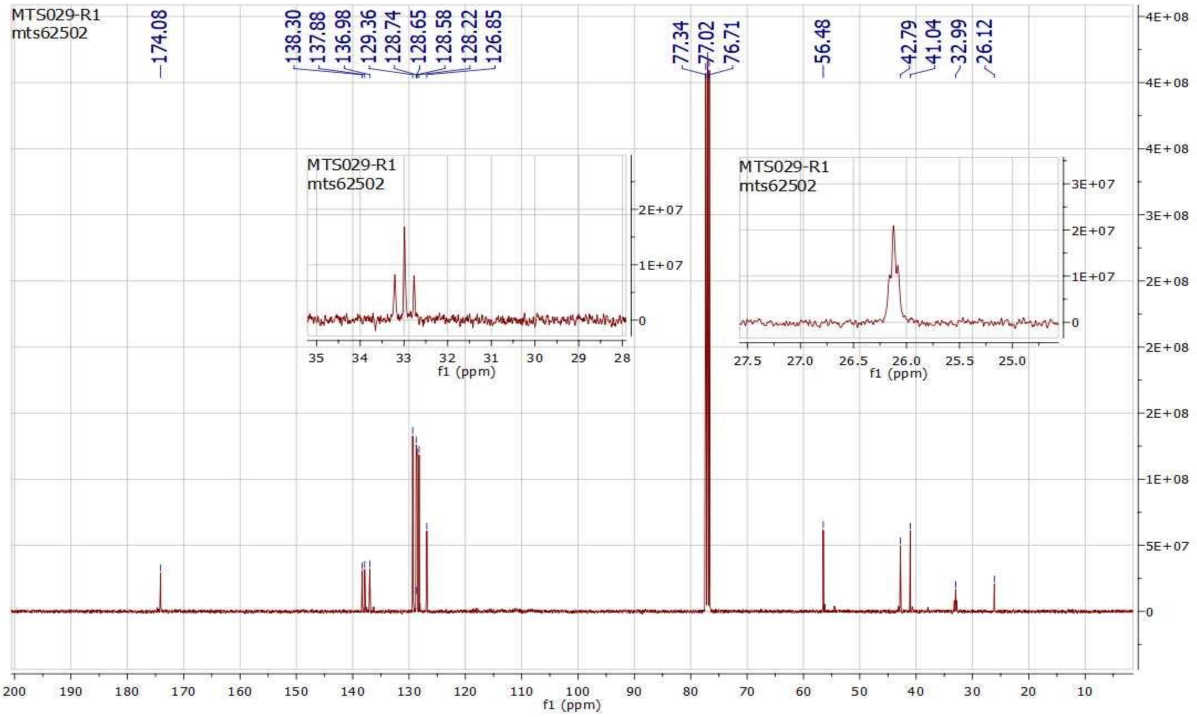

^{19}F NMR

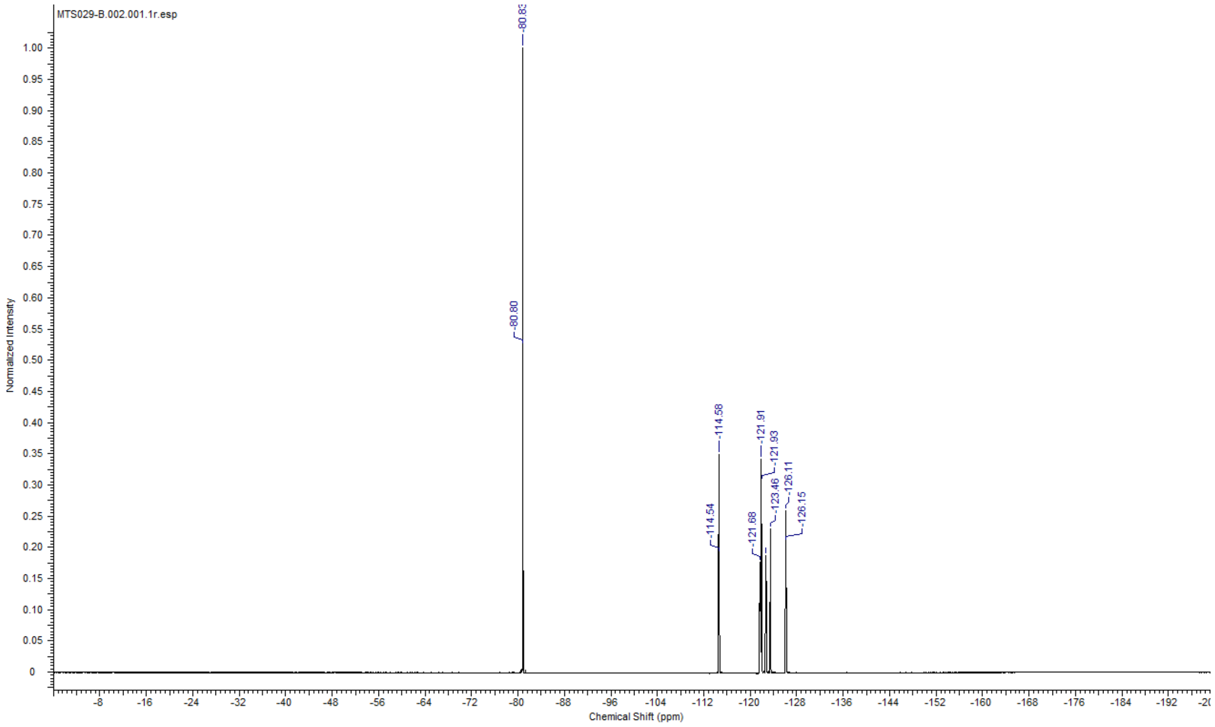
MTS029-B.002.001.1r.esp
-80.83
-80.80
-114.58
-114.54
-121.91
-121.93
-121.68
-123.46
-126.11
-126.15
Normalized Intensity
Chemical Shift (ppm)

***(S)*-2-Amino-3-phenyl-*N*-(pyridin-2-ylmethyl)propanamide (6o)**

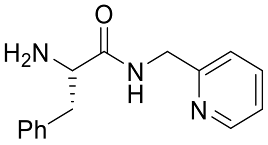

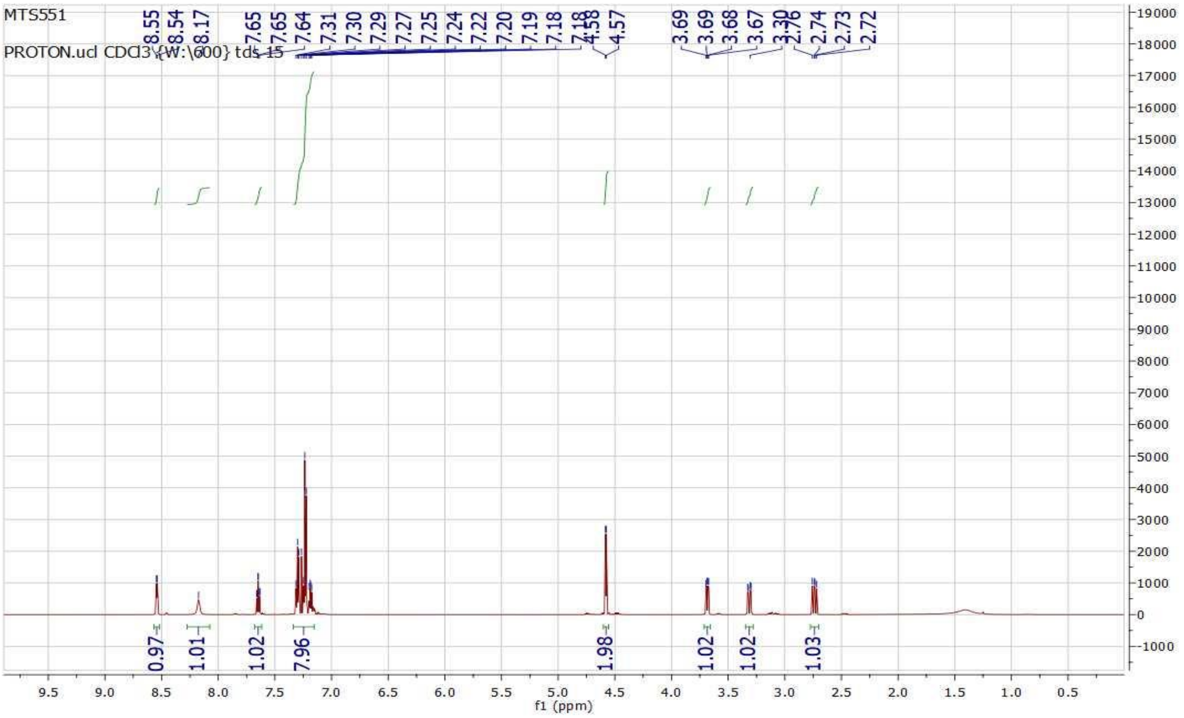

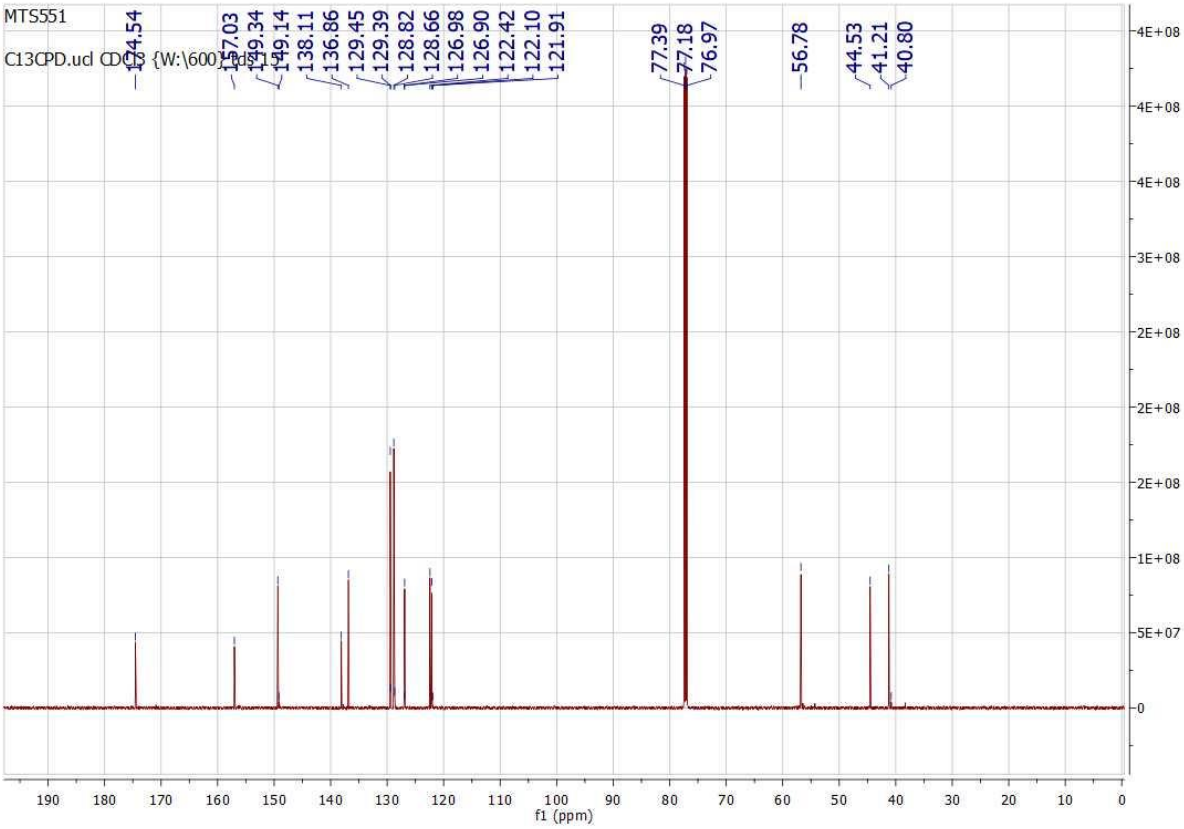

(*S*)-2-Amino-*N*-(4-methoxyphenyl)-3-phenylpropanamide (6p)

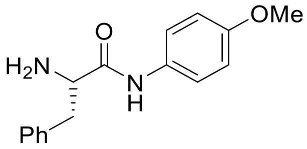

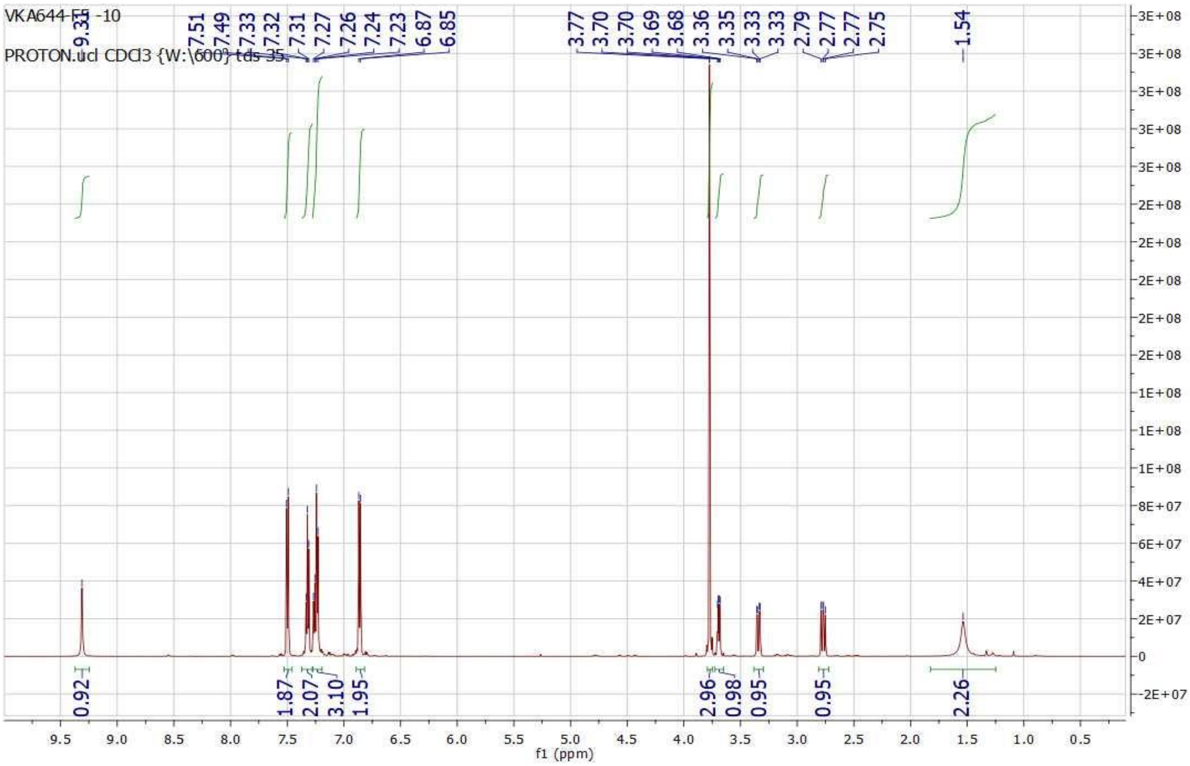

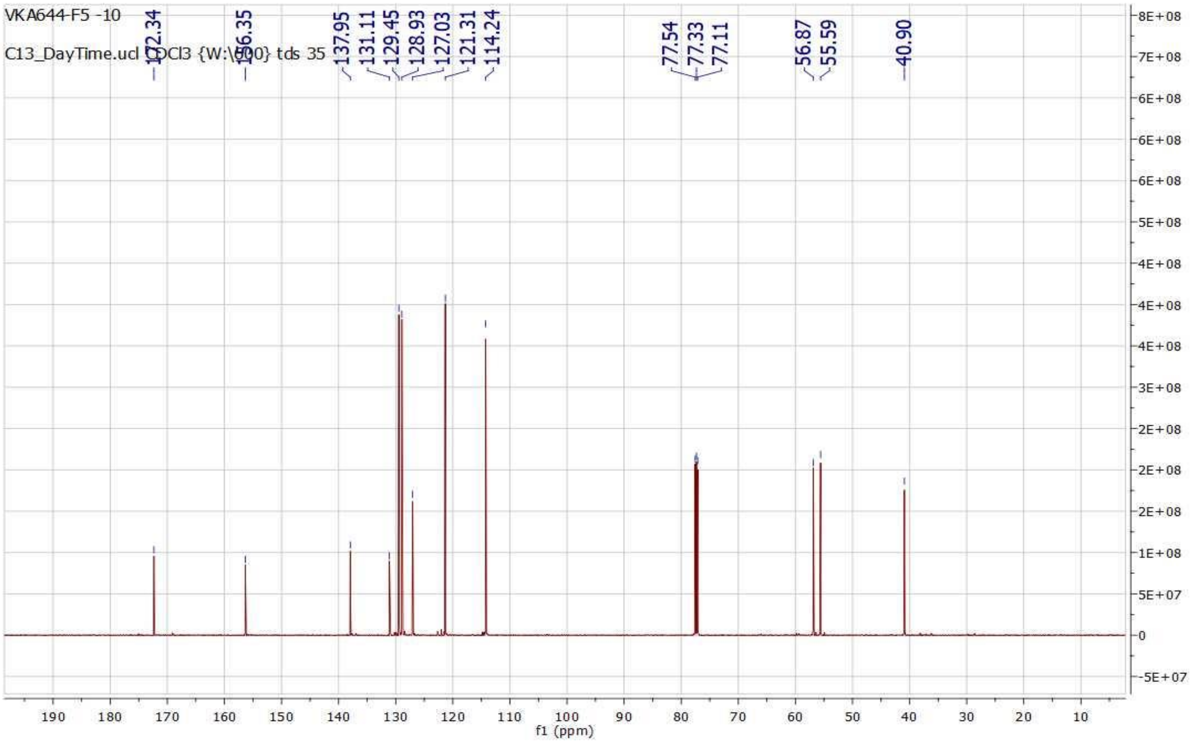

(*S*)-2-Amino-4-methyl-1-(pyrrolidin-1-yl)pentan-1-one (6s)

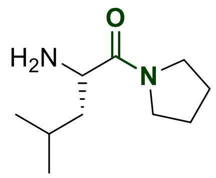

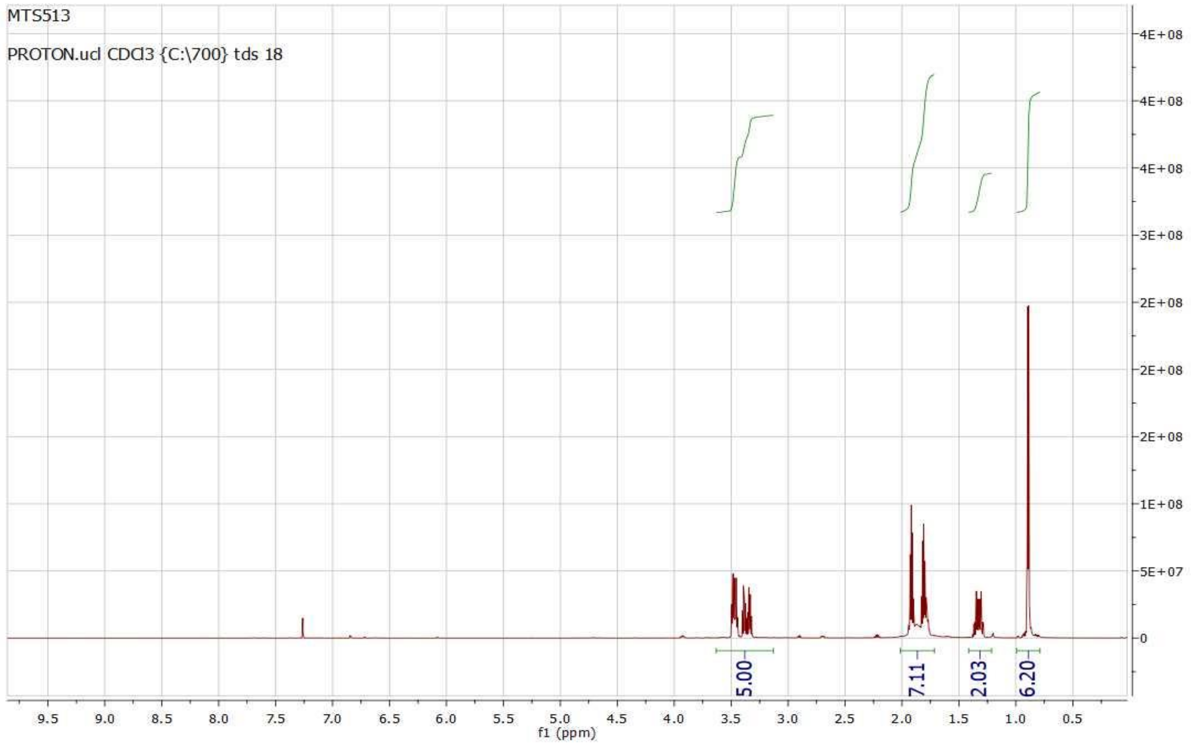

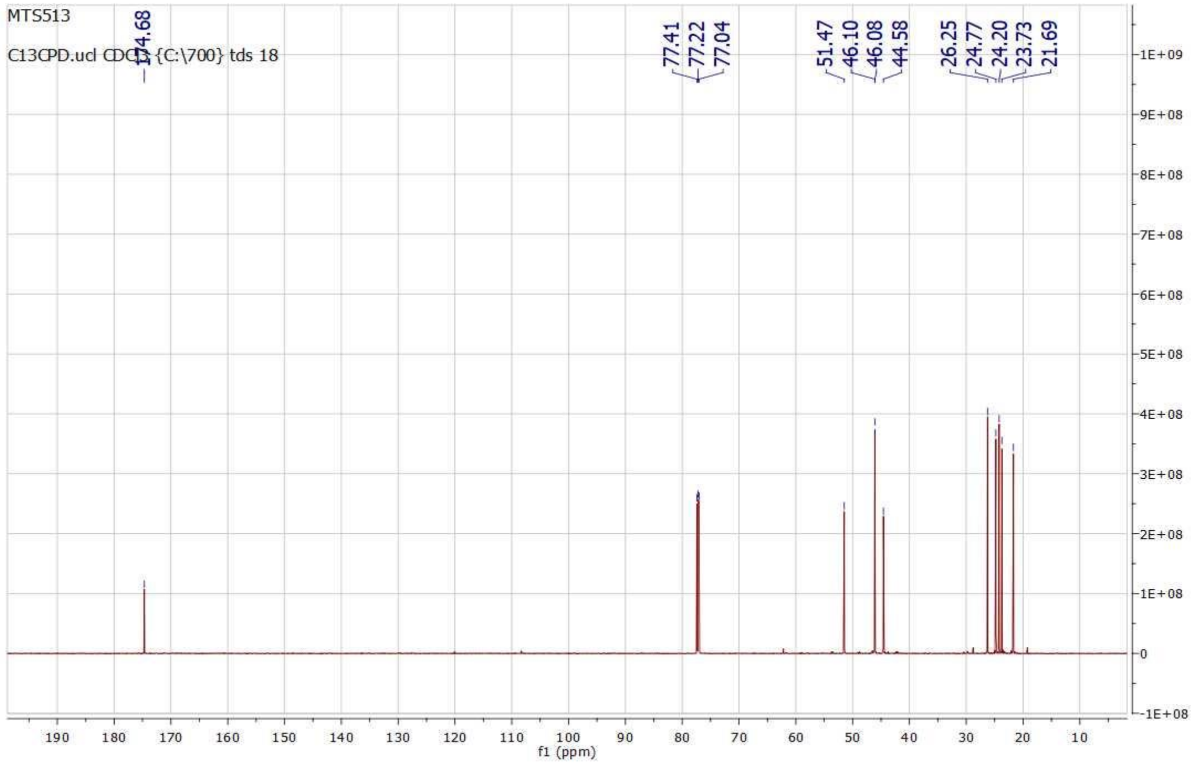

(*S*)-2-Amino-3-phenyl-1-(pyrrolidin-1-yl)propan-1-one (6u)

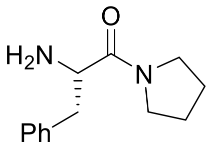

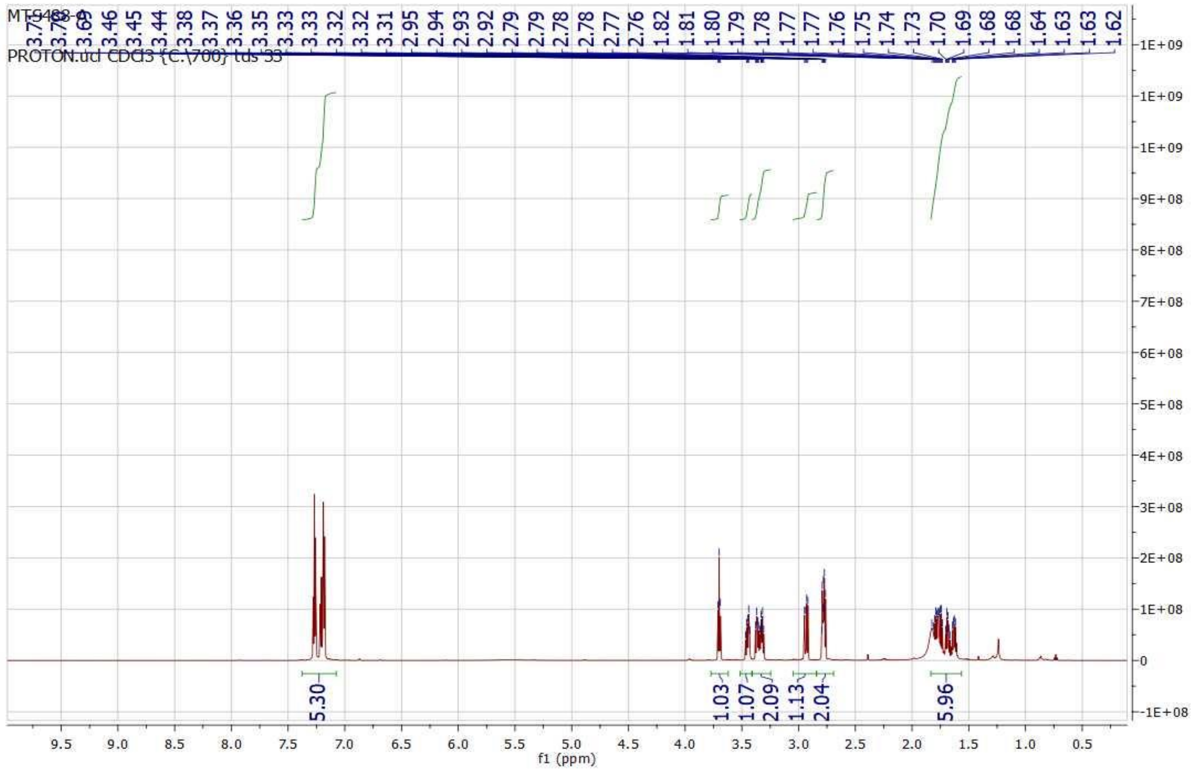

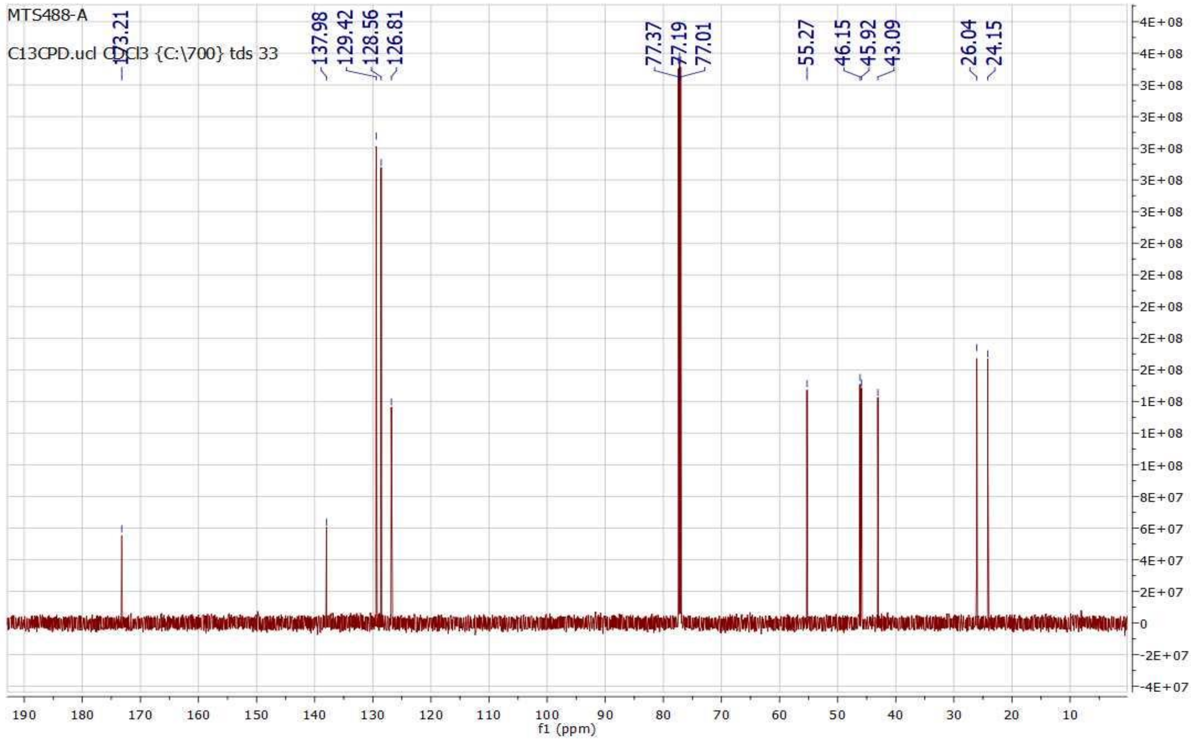

tert-Butyl L-phenylalanylglycinate (6y)

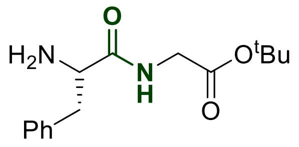

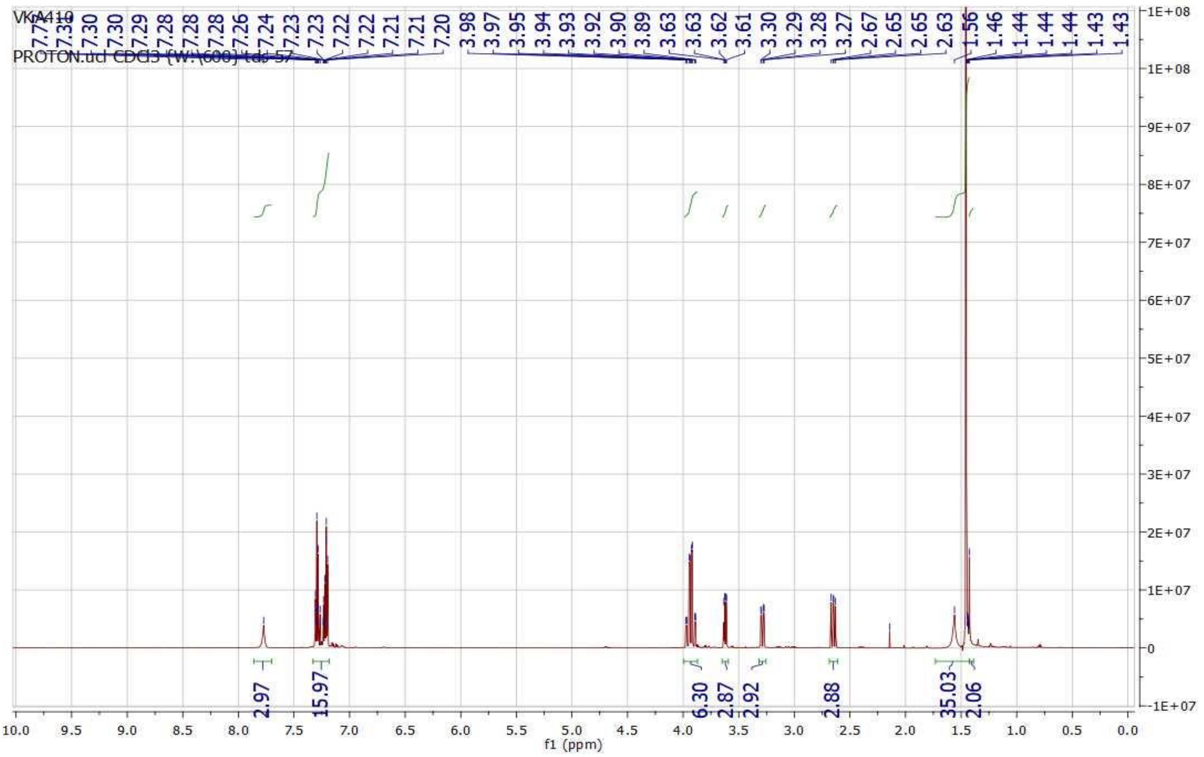

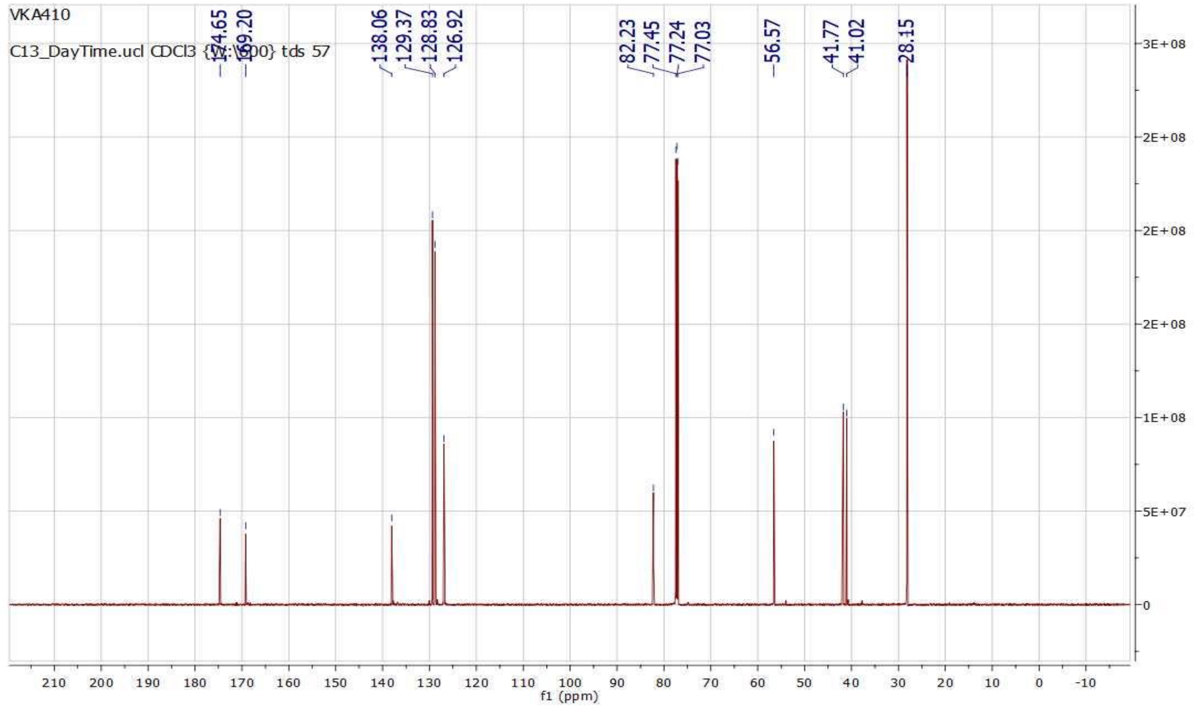

(*R*)-3-Amino-3-(2-chlorophenyl)-*N*,*N*-dimethylpropanamide (6cc)

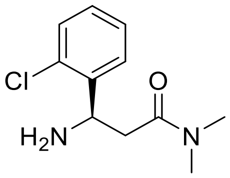

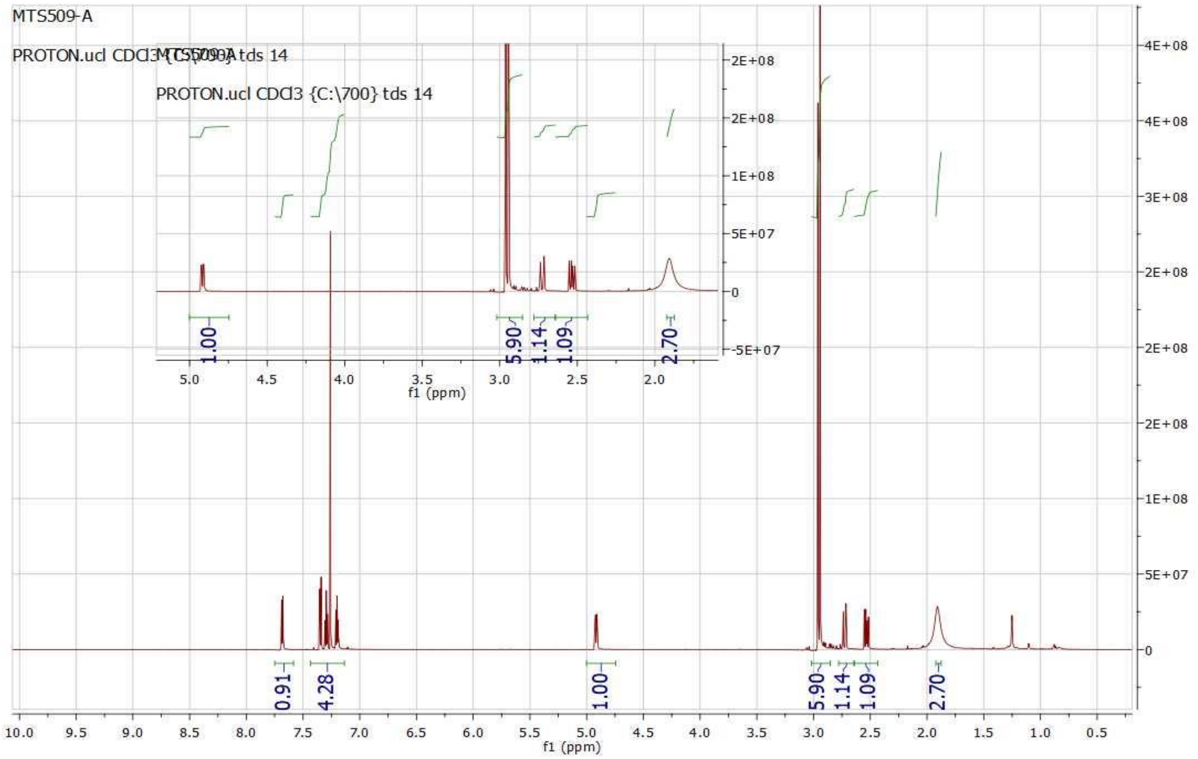

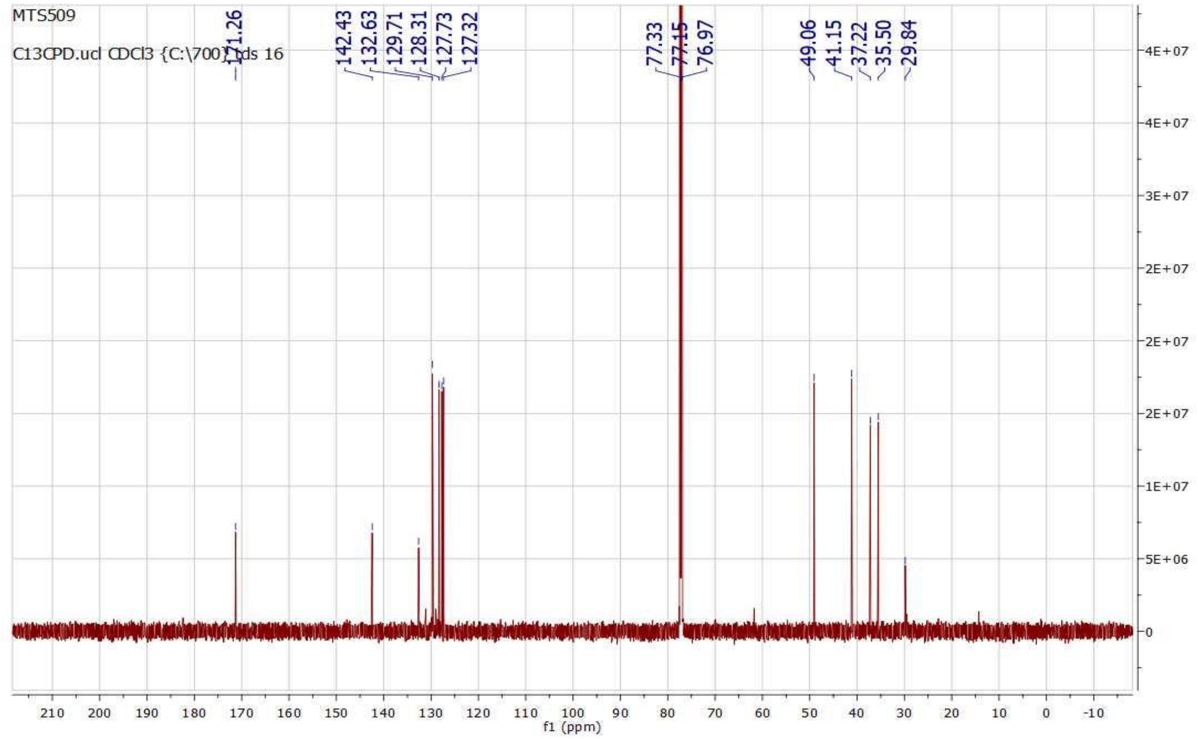

(*S*)-2-Amino-*N*,*N*-4-trimethylpentanamide (6dd) (95:5 dr)

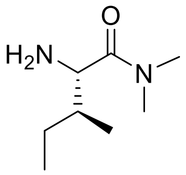
O
H2N
N

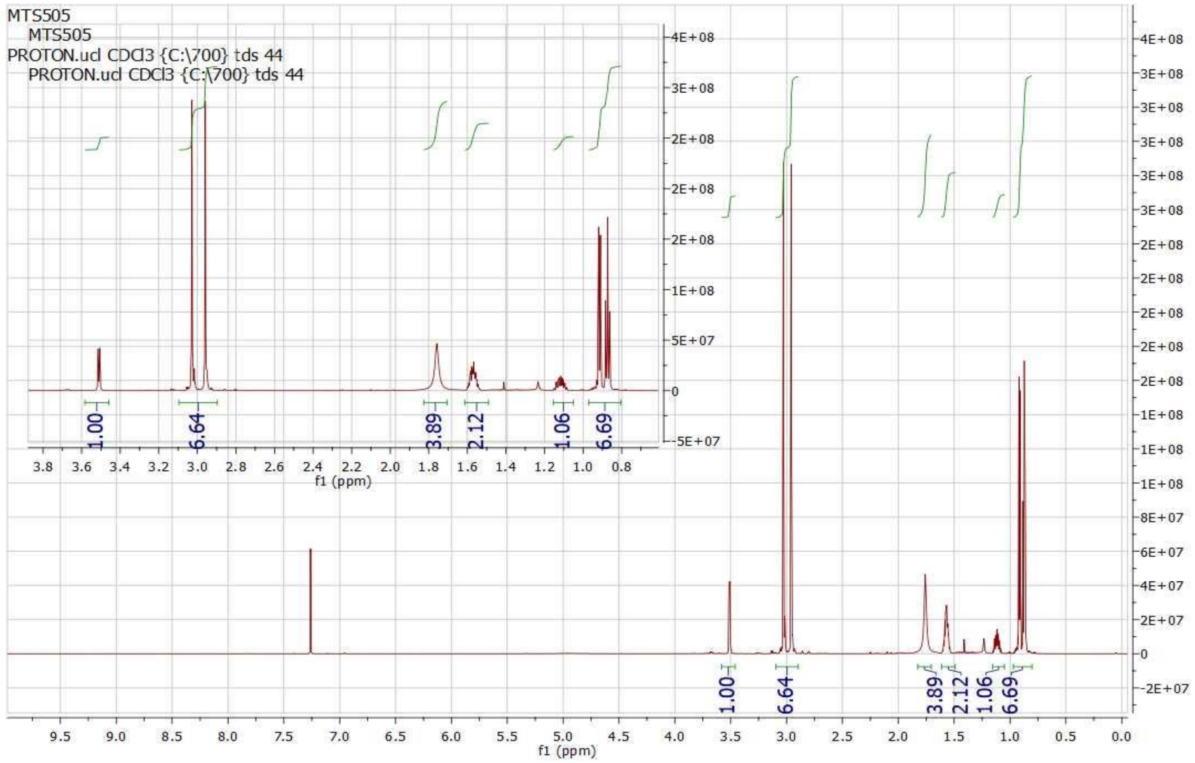
MTS505
MTS505
PROTON.ucl CDCl3 {C:\700} tds 44
PROTON.ucl CDCl3 {C:\700} tds 44
1.00
6.64
3.89
2.12
1.06
6.69
f1 (ppm)

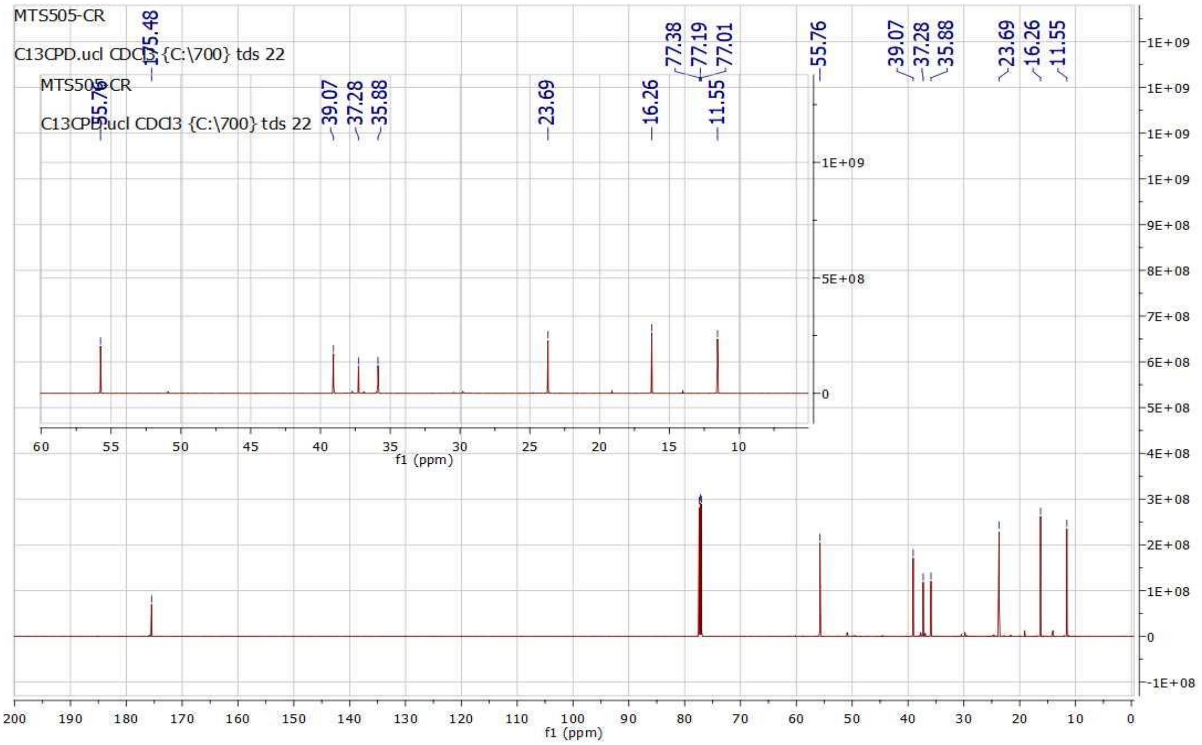
MTS505-CR
C13CPD.ucl CDCl3 {C:\700} tds 22
MTS505-CR
C13CPD.ucl CDCl3 {C:\700} tds 22
175.48
77.38
77.19
77.01
55.76
39.07
37.28
35.88
23.69
16.26
11.55
f1 (ppm)

(*S*)-2-Amino-*N*,*N*-dimethyl-3-phenylpropanamide (6ee)

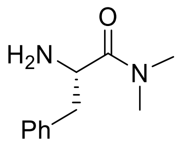

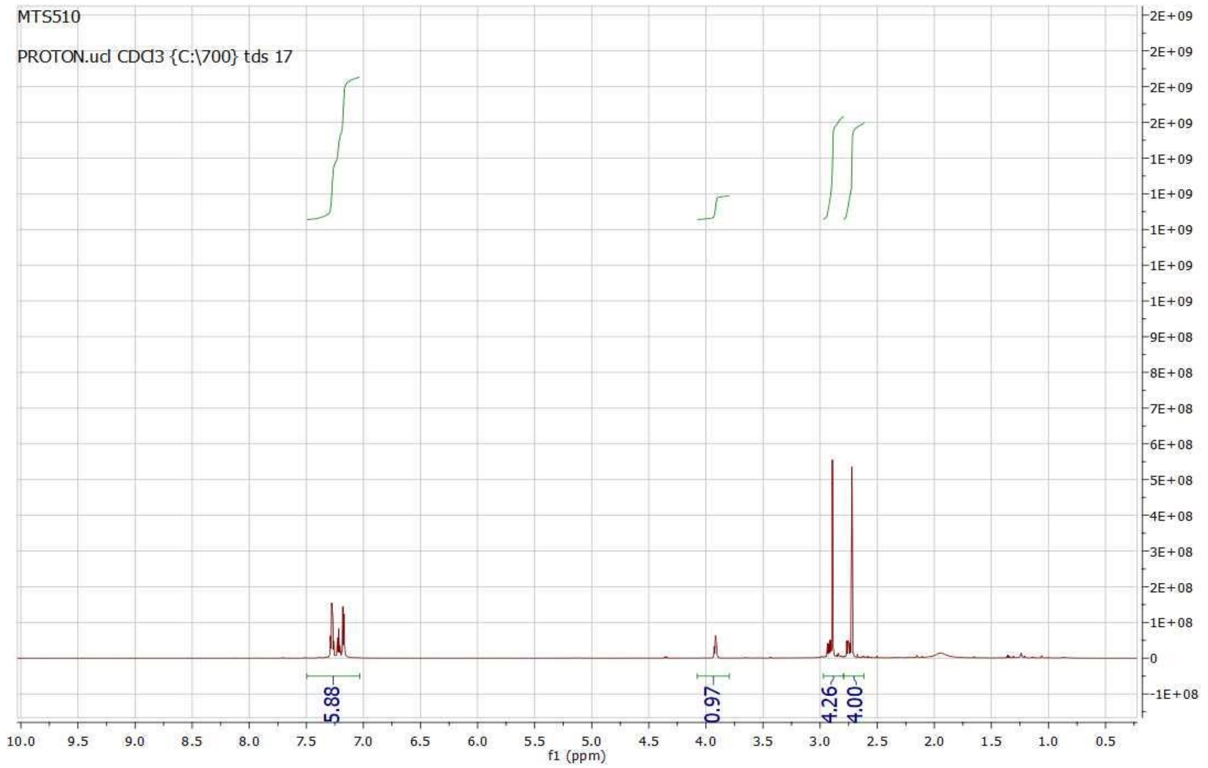

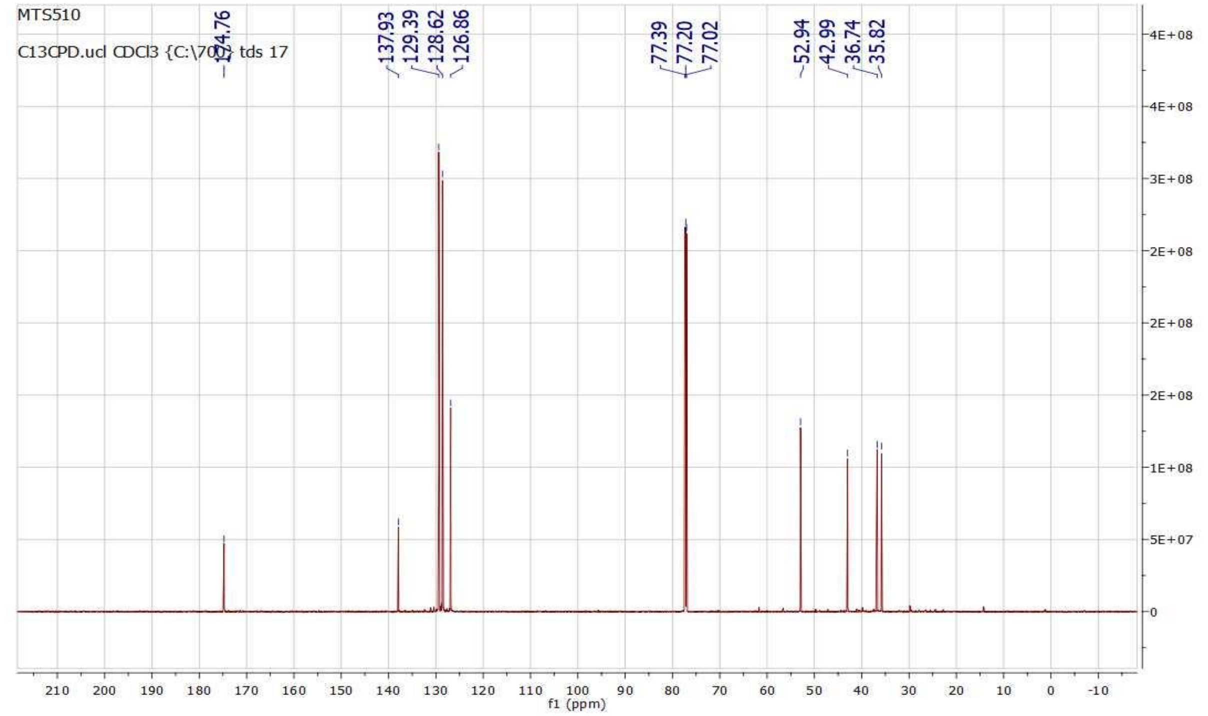

(*S*)-2-Amino-3-(1*H*-indol-3-yl)-*N,N*-dimethylpropanamide (6ff)

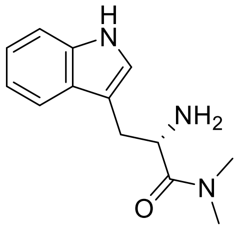

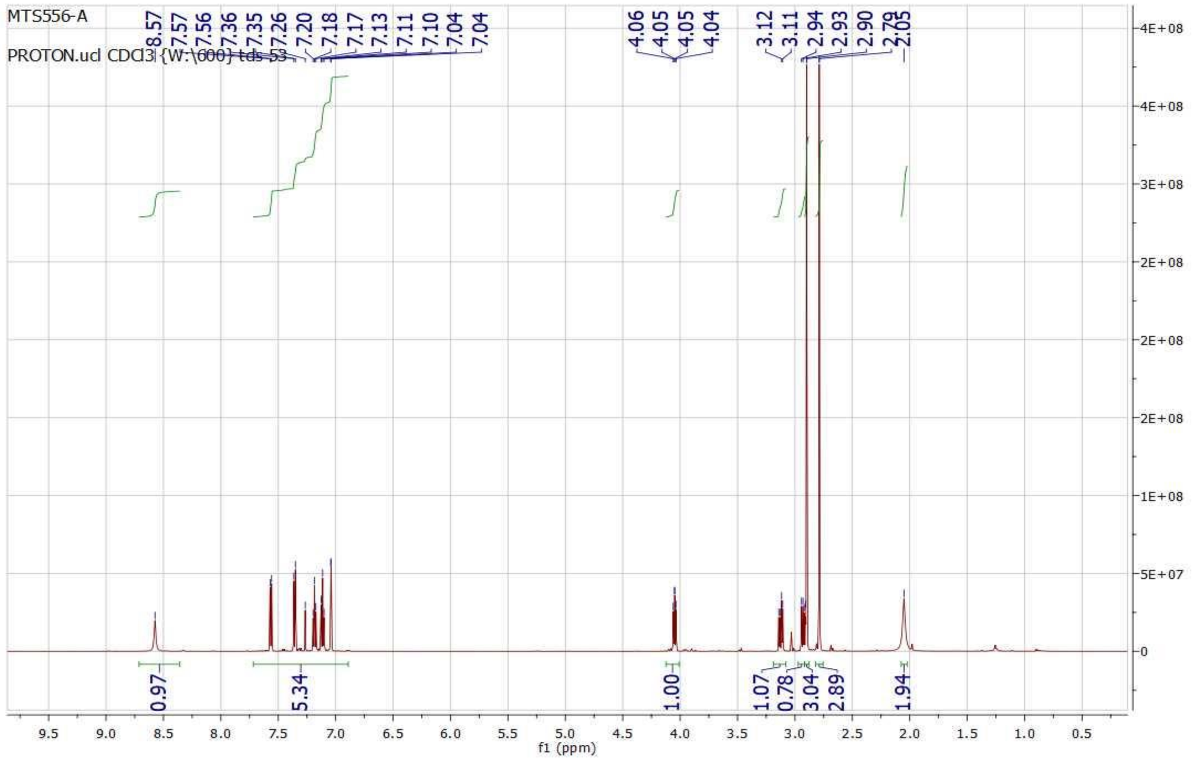

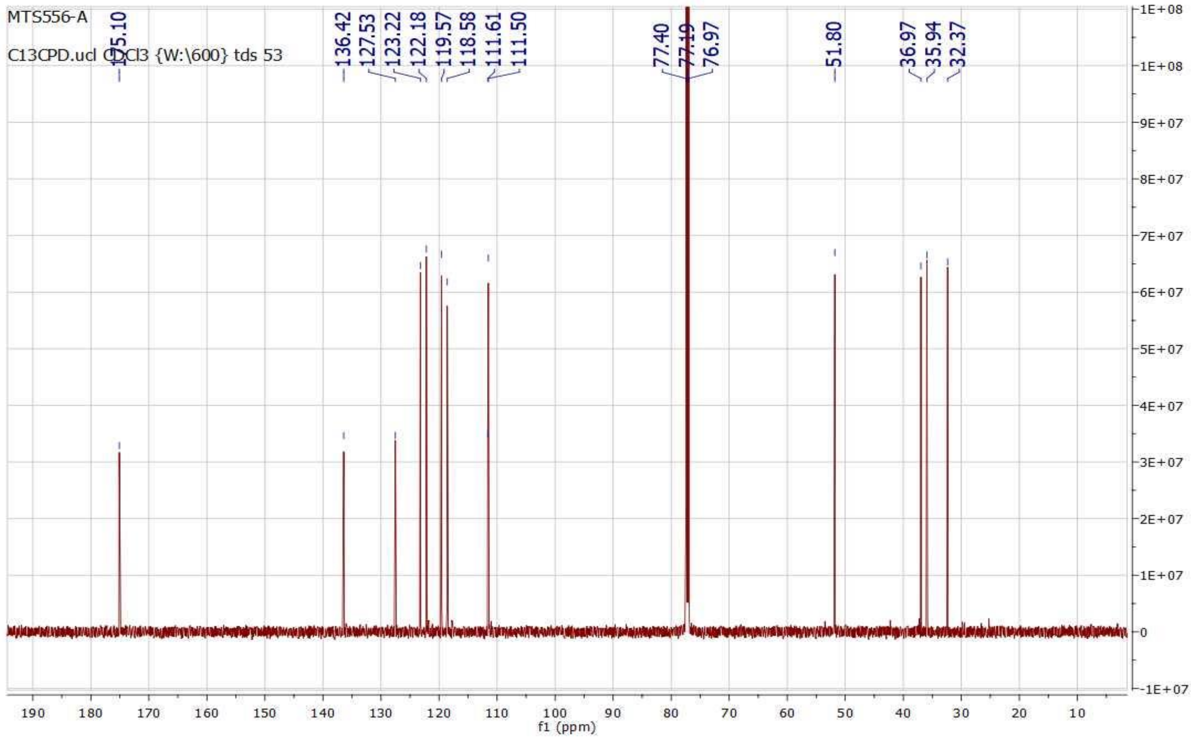

(*S*)-2-Amino-*N*,*N*-dimethyl-4-(methylthio)butanamide (6gg)

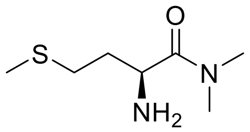

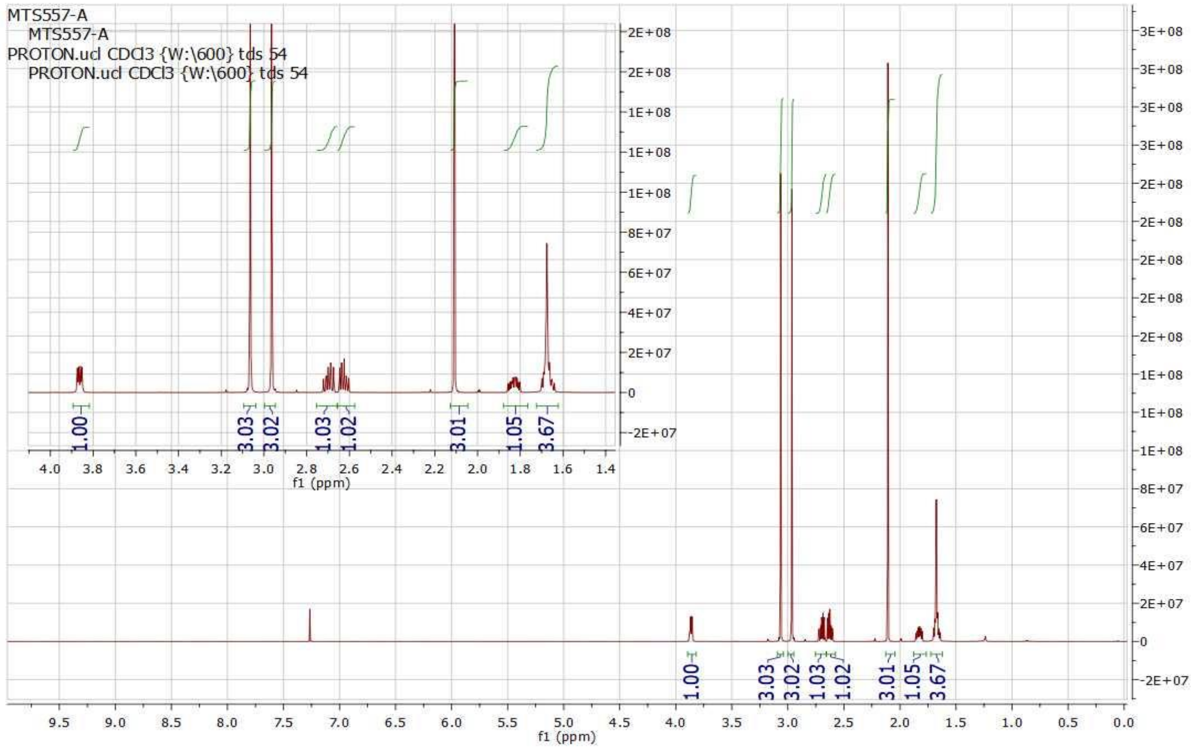

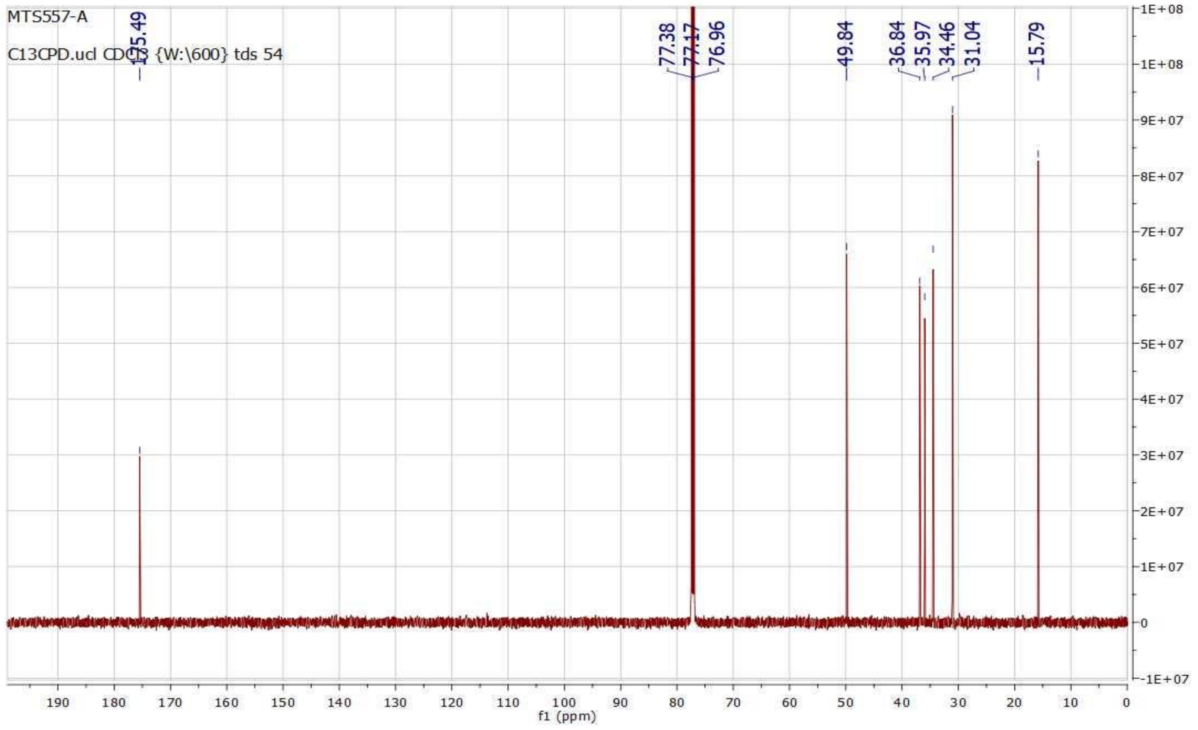

(*S*)-3-Benzyl-5-phenyl-1,3-dihydro-2H-benzo[e][1,4]diazepin-2-one (8)

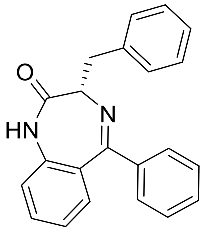

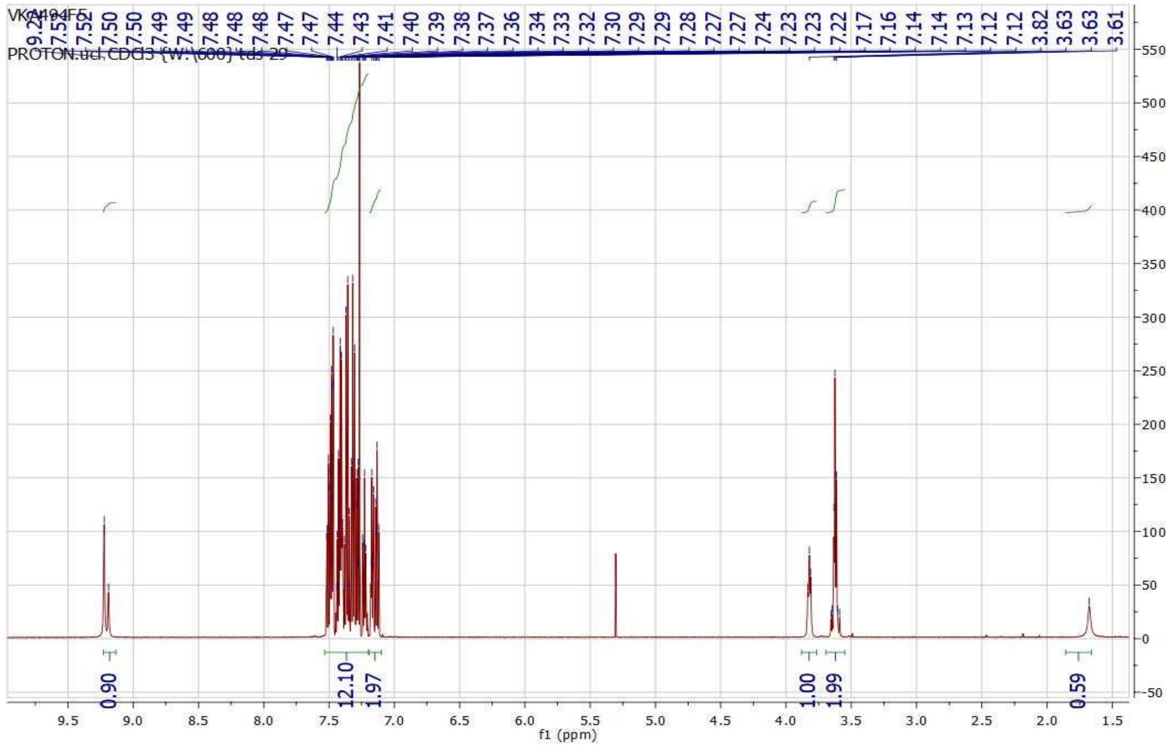

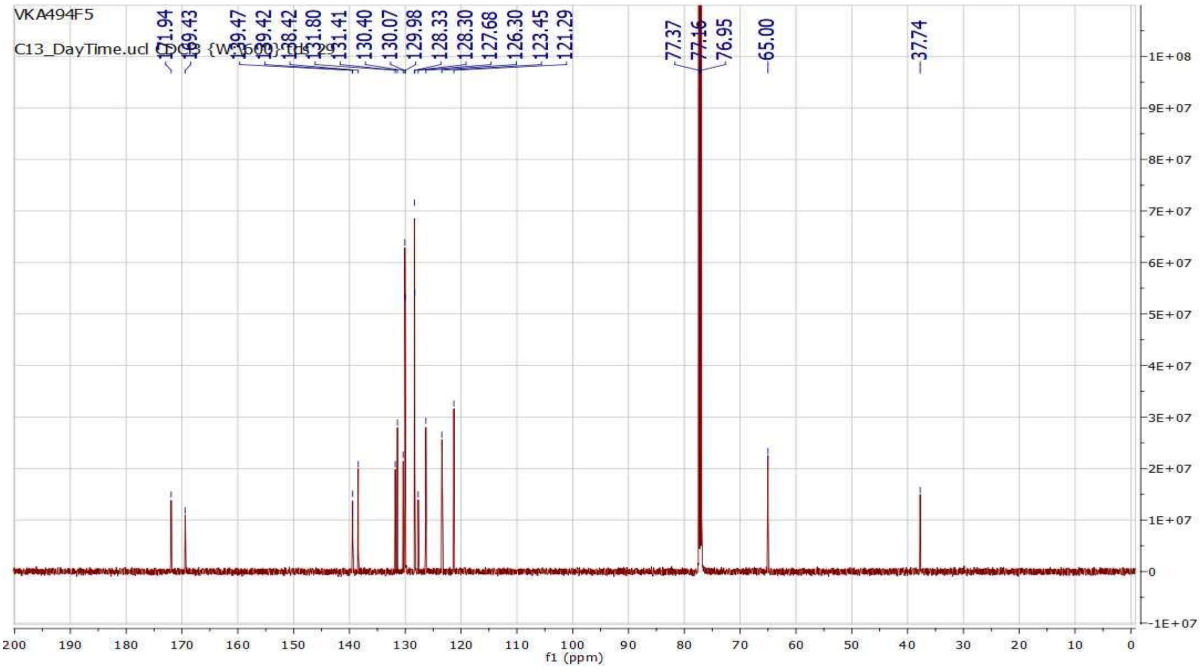

(*S*)-2-(2-(4-Bromophenyl)acetamido)-3-phenyl-*N*-propylpropanamide (10a)

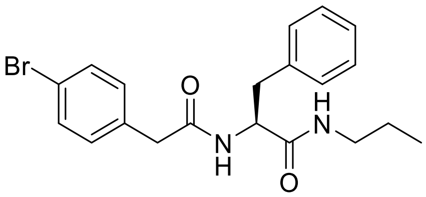

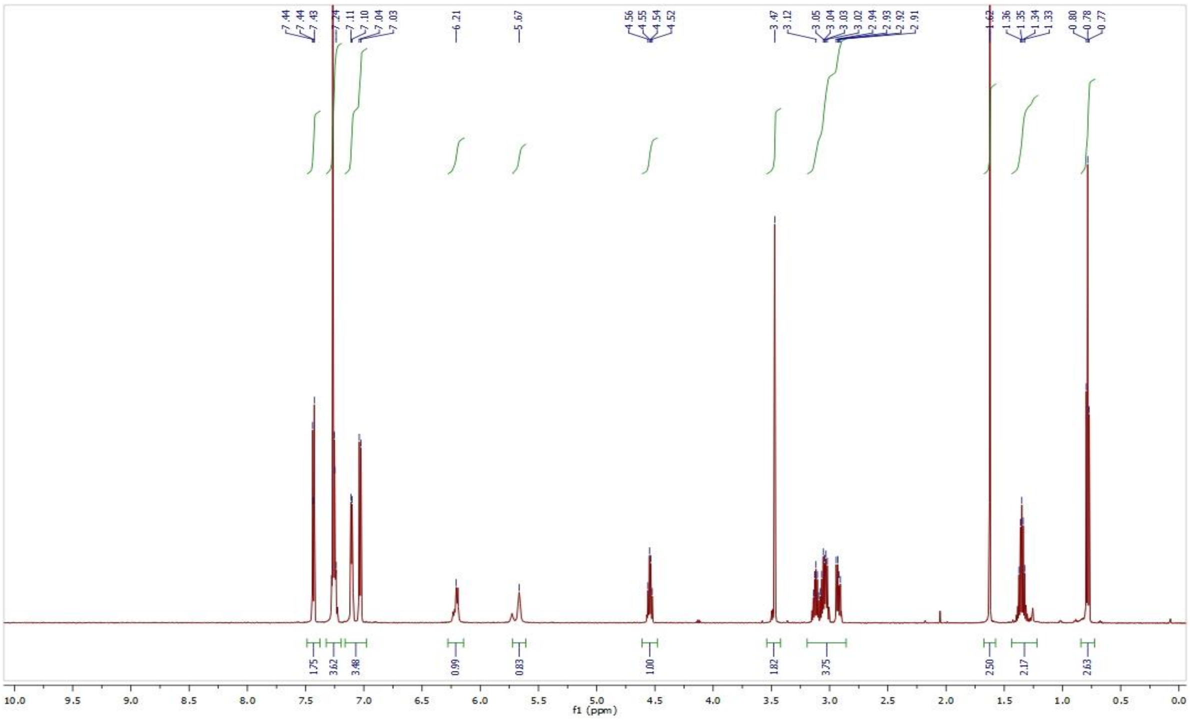

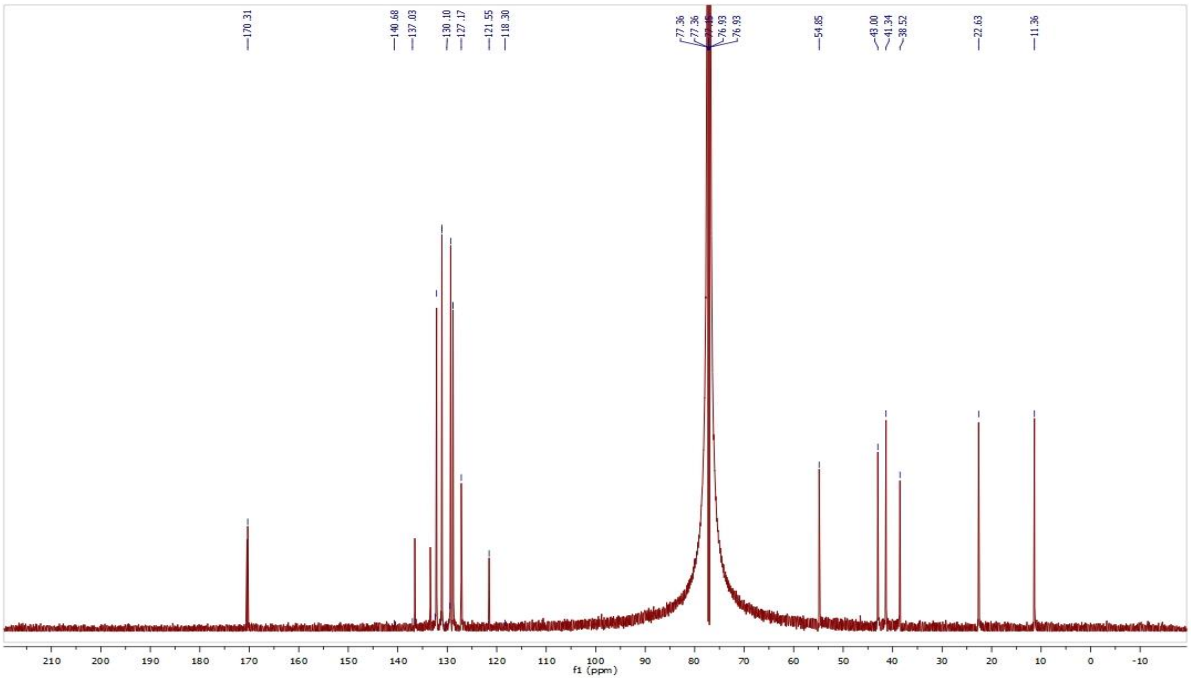

***tert*-Butyl (*R*)-methyl(2-oxo-2-((1-oxo-3-phenyl-1-(propylamino)propan-2-yl)amino)ethyl)carbamate (10b)**

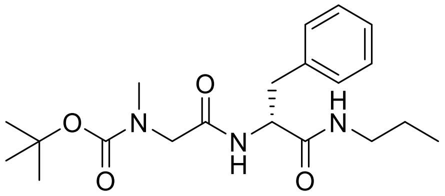

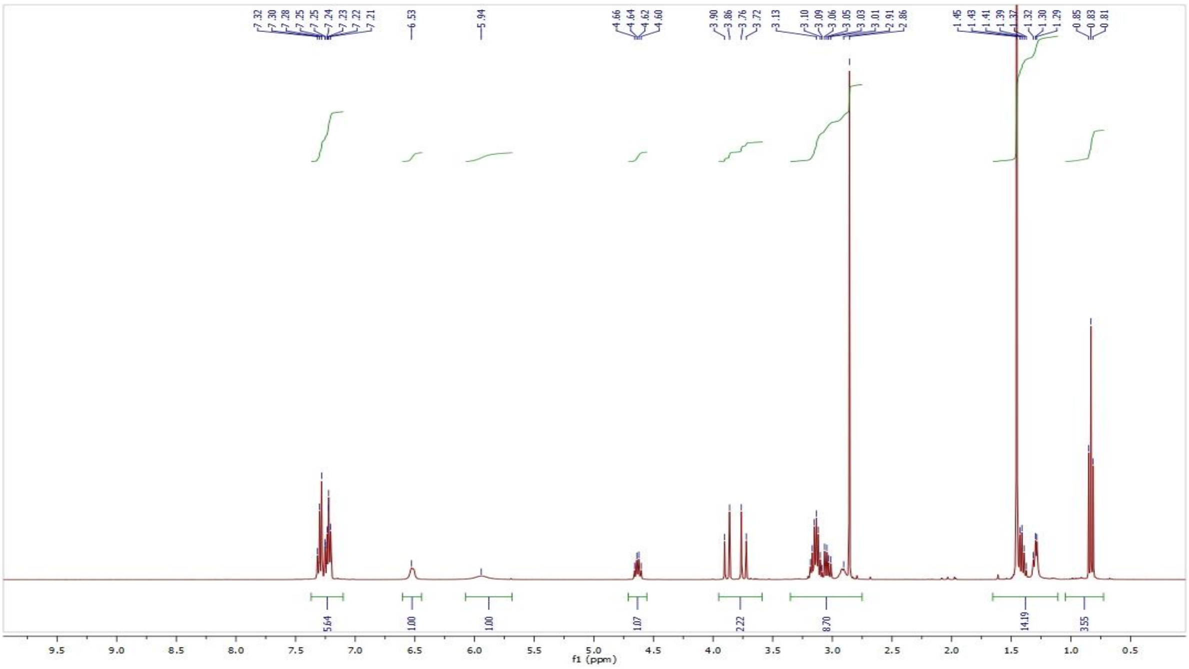

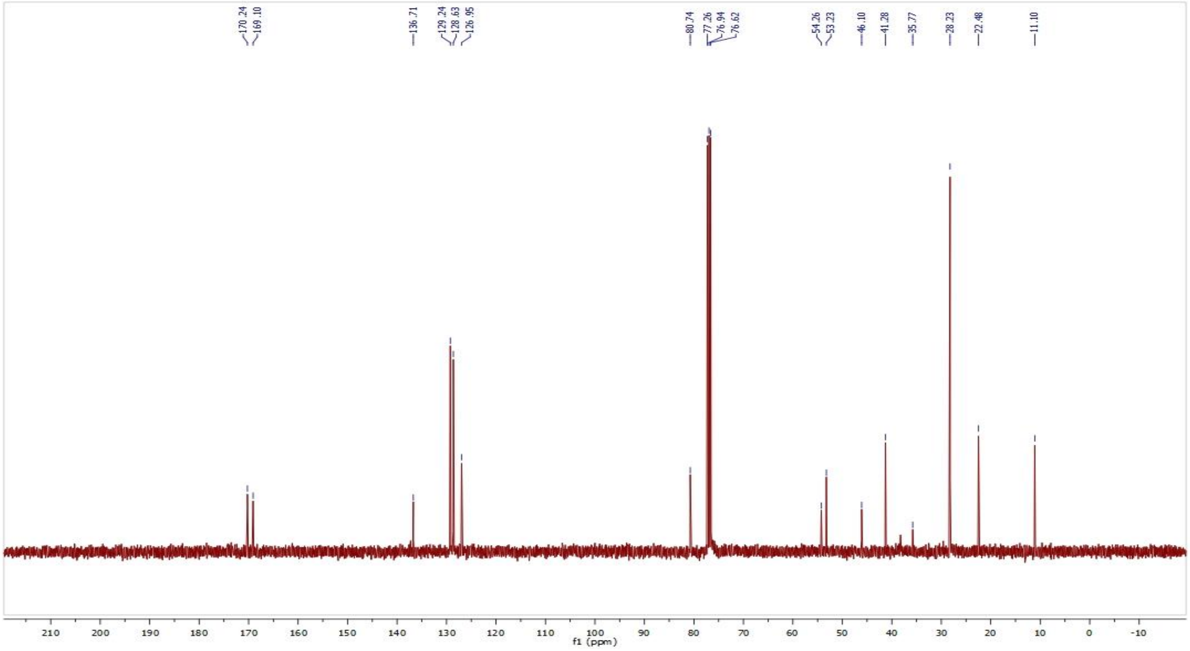

***N*-(2-Oxo-2-(propylamino)ethyl)picolinamide (10c)**

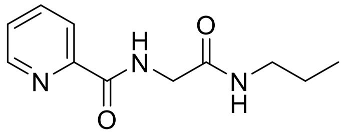

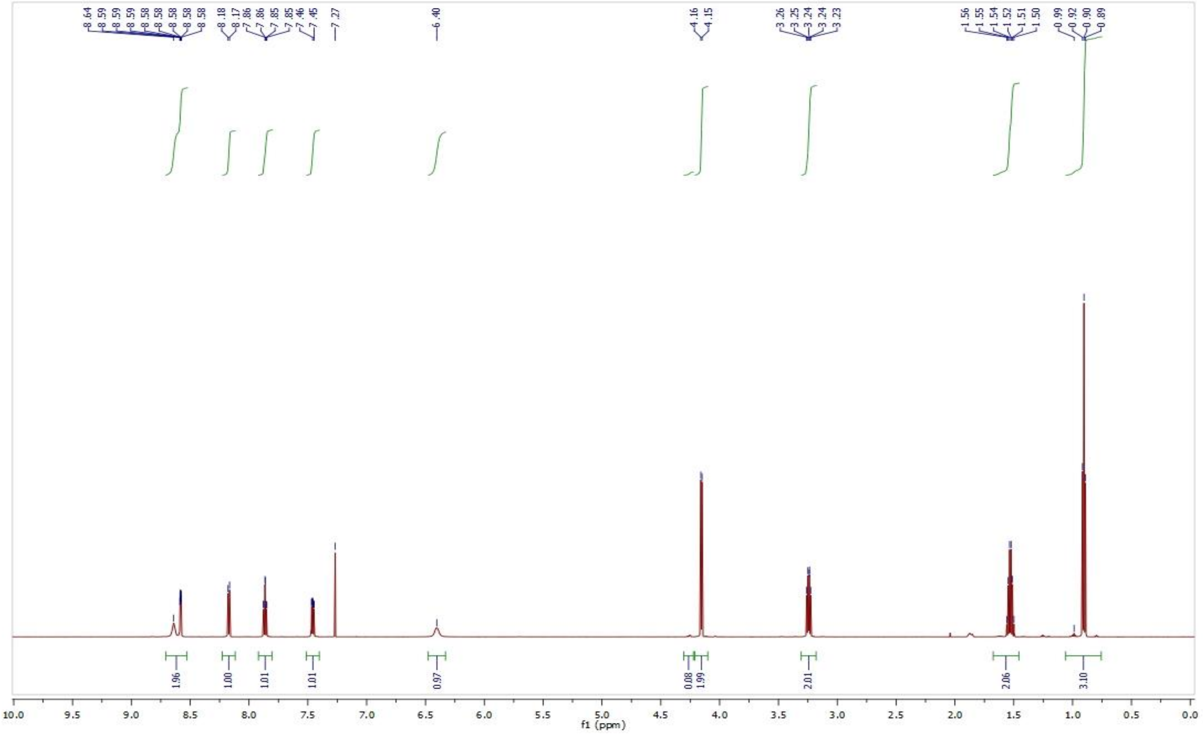

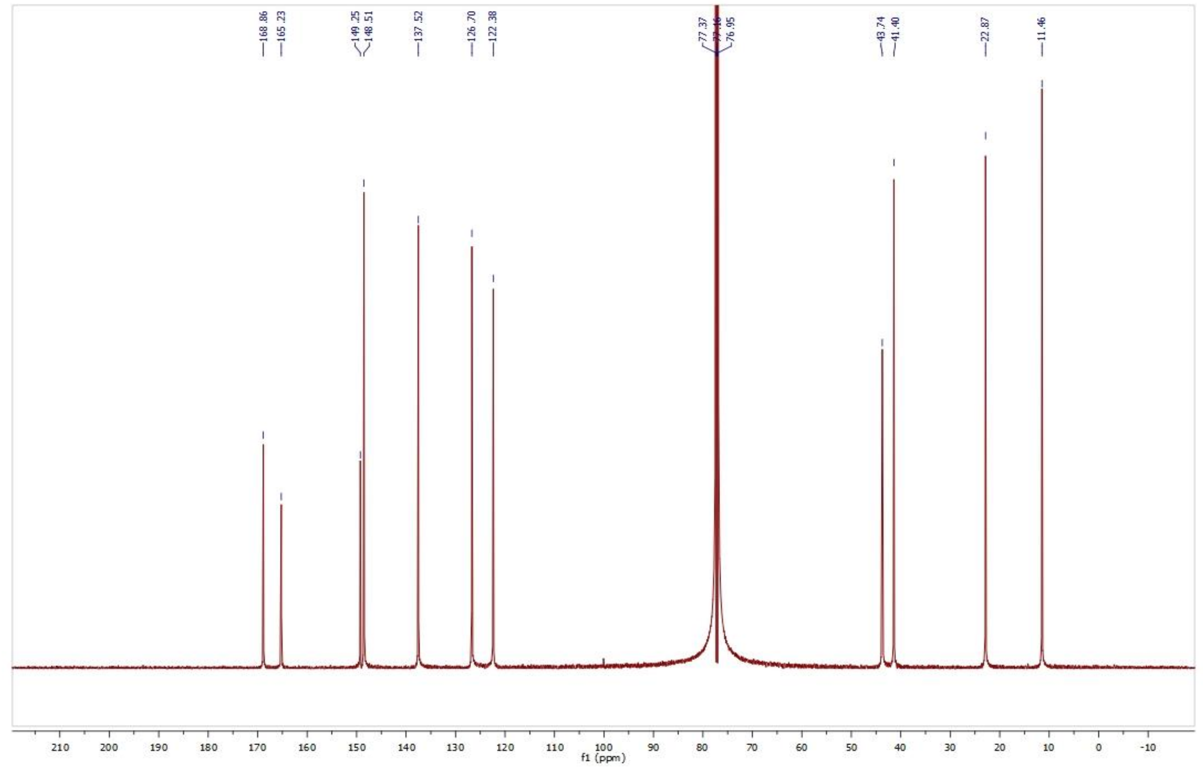

***N*-(2-Oxo-2-(propylamino)ethyl)-2-phenylacetamide (10d)**

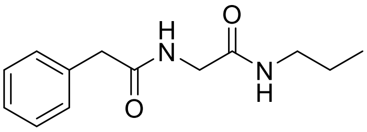

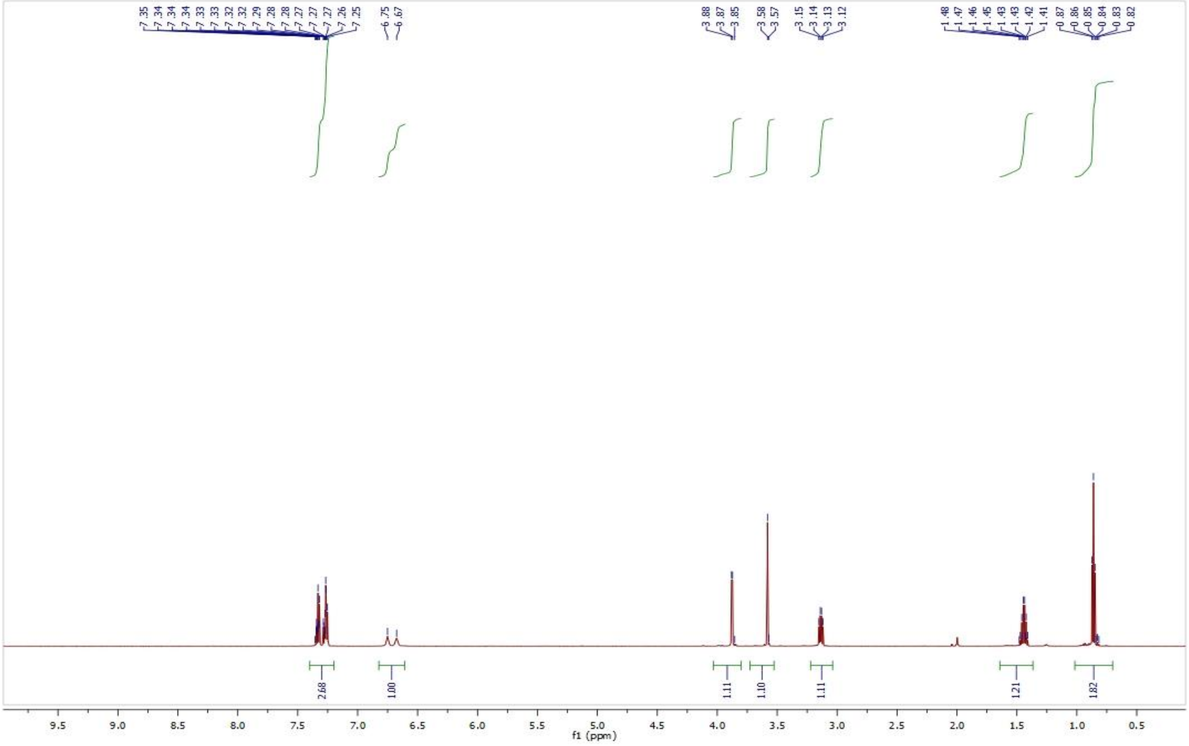

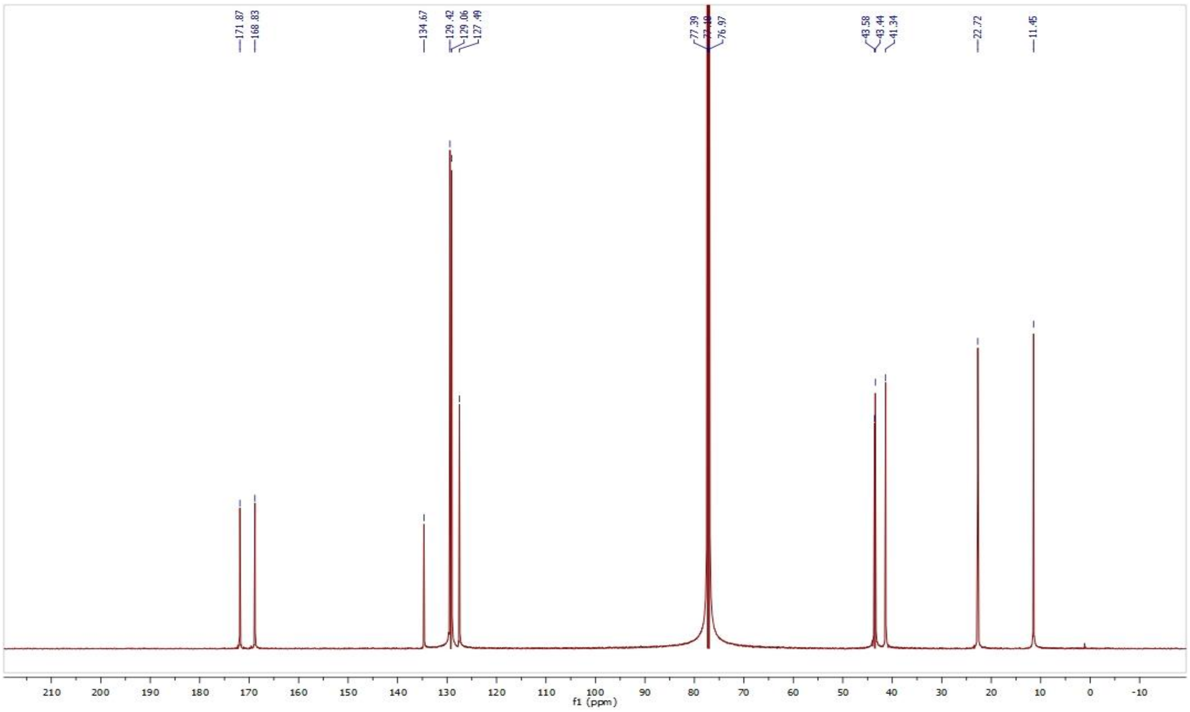

3-Hydroxy-*N*-(2-Oxo-2-(propylamino)ethyl)-2-phenylpropanamide (10e)

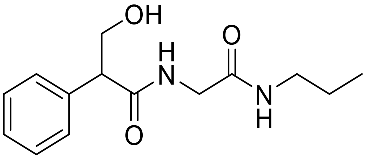

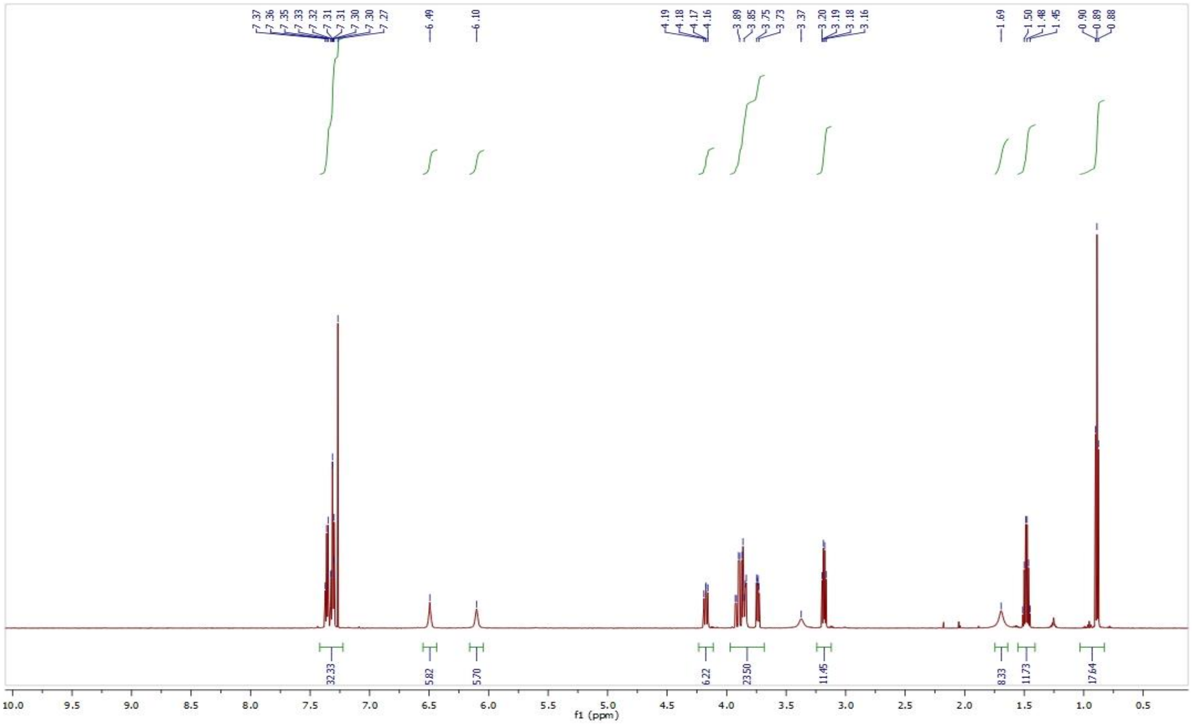

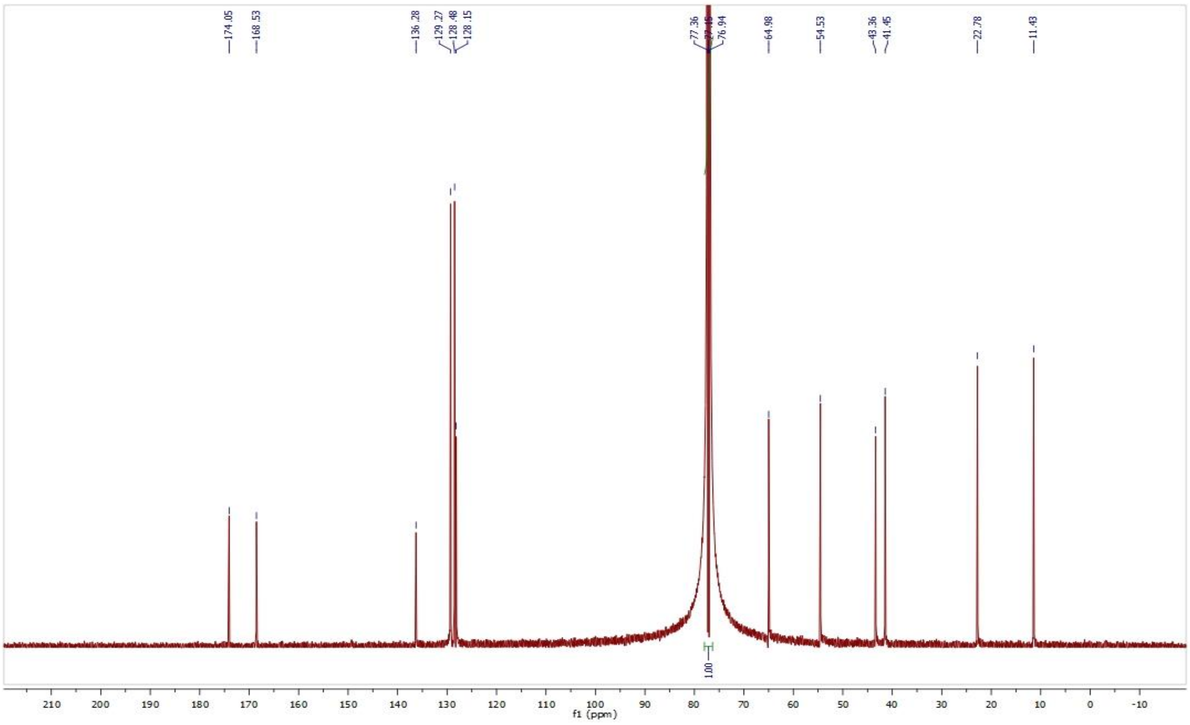

***tert*-Butyl (2-((2-(benzylamino)-2-oxoethyl)(methyl)amino)-2-oxoethyl)(methyl) carbamate (11a)**

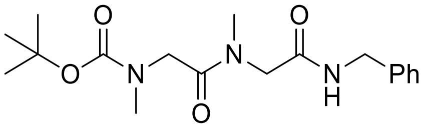

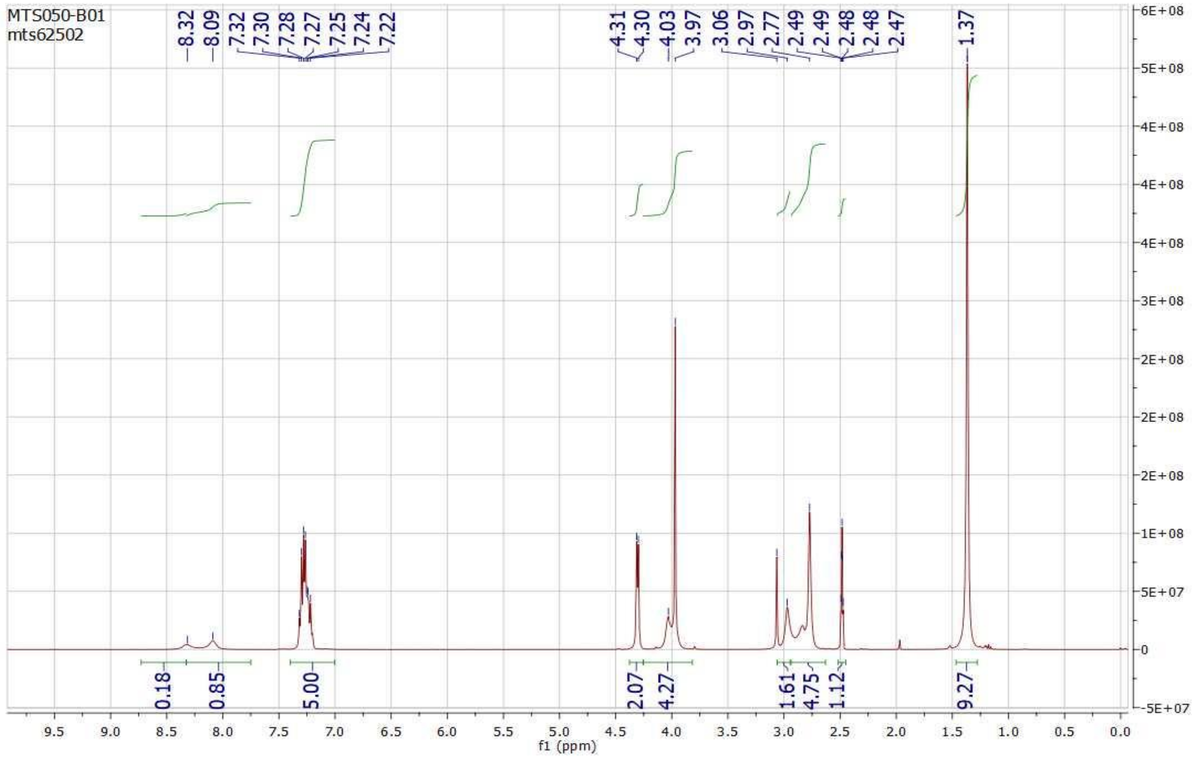

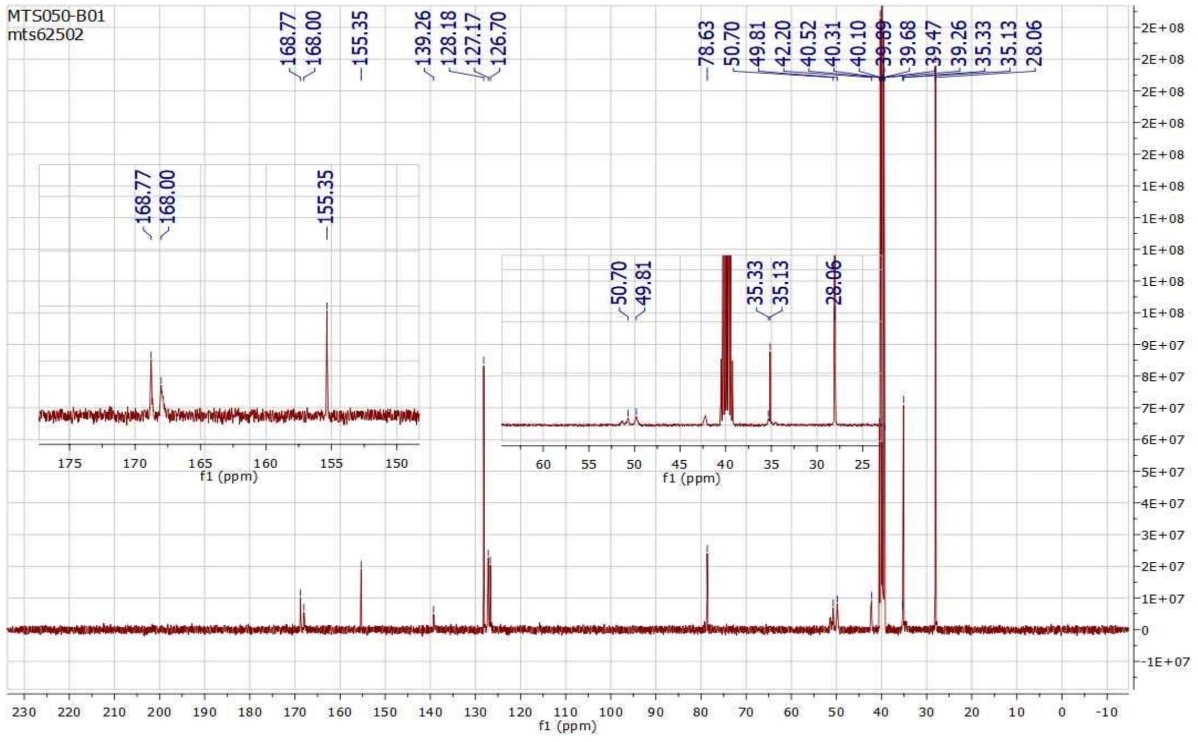

tert-Butyl ((*S*)-1-(((*S*)-1-(benzylamino)-4-methyl-1-oxopentan-2-yl)amino)-1-oxo-3-phenylpropan-2-yl)carbamate (11b)

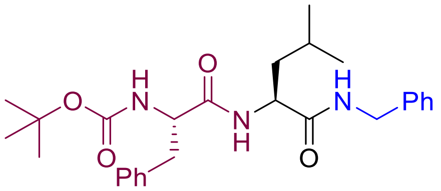

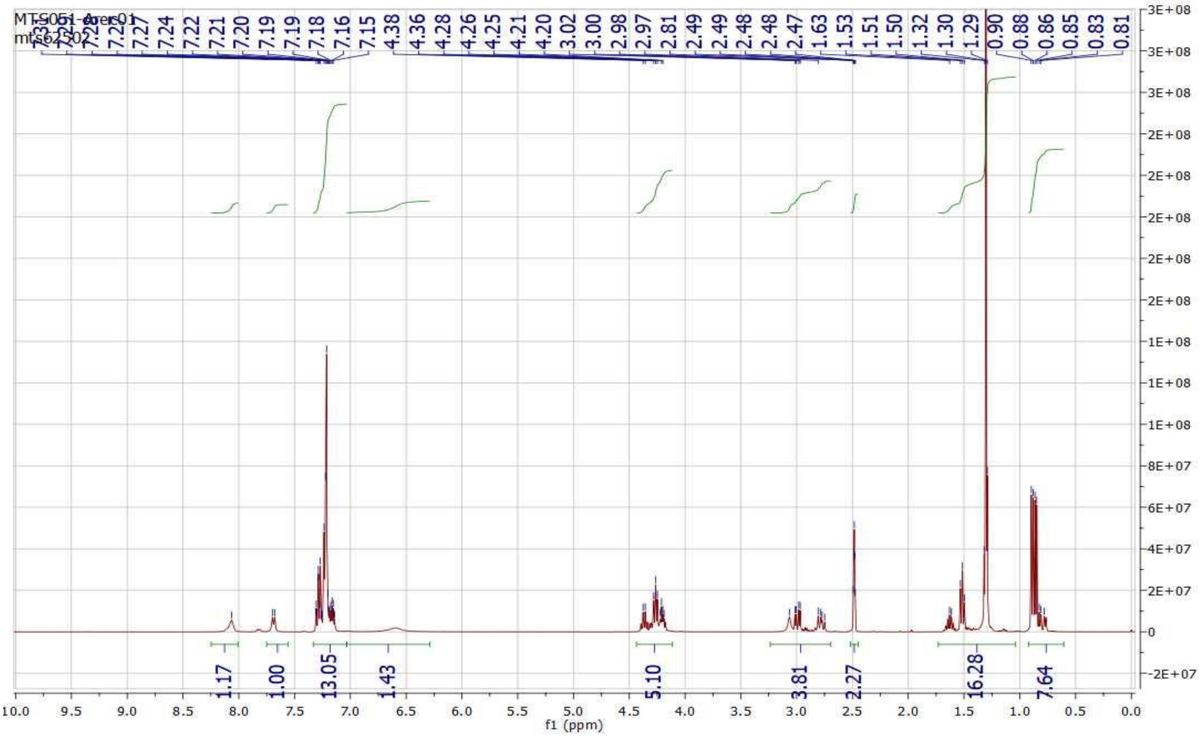

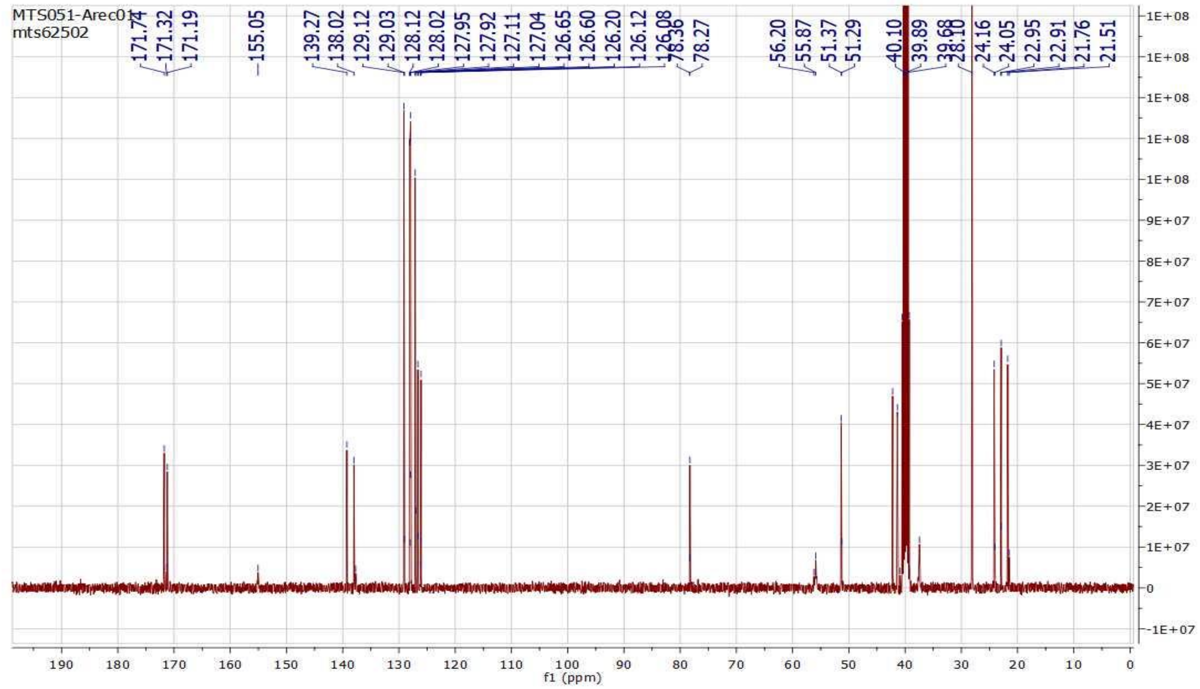

(*S*)-*N*-Benzyl-3-(1*H*-indol-3-yl)-2-(2-phenylacetamido)propenamide (11c)

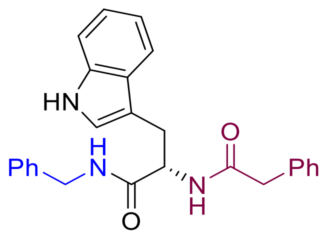

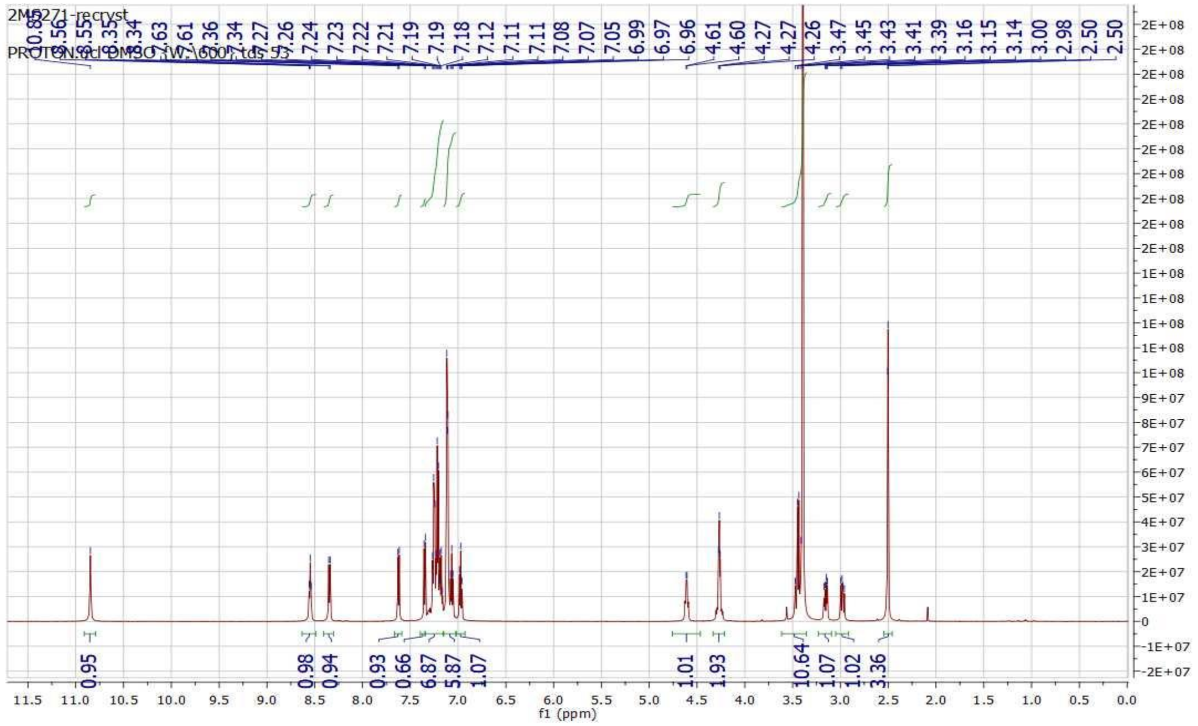

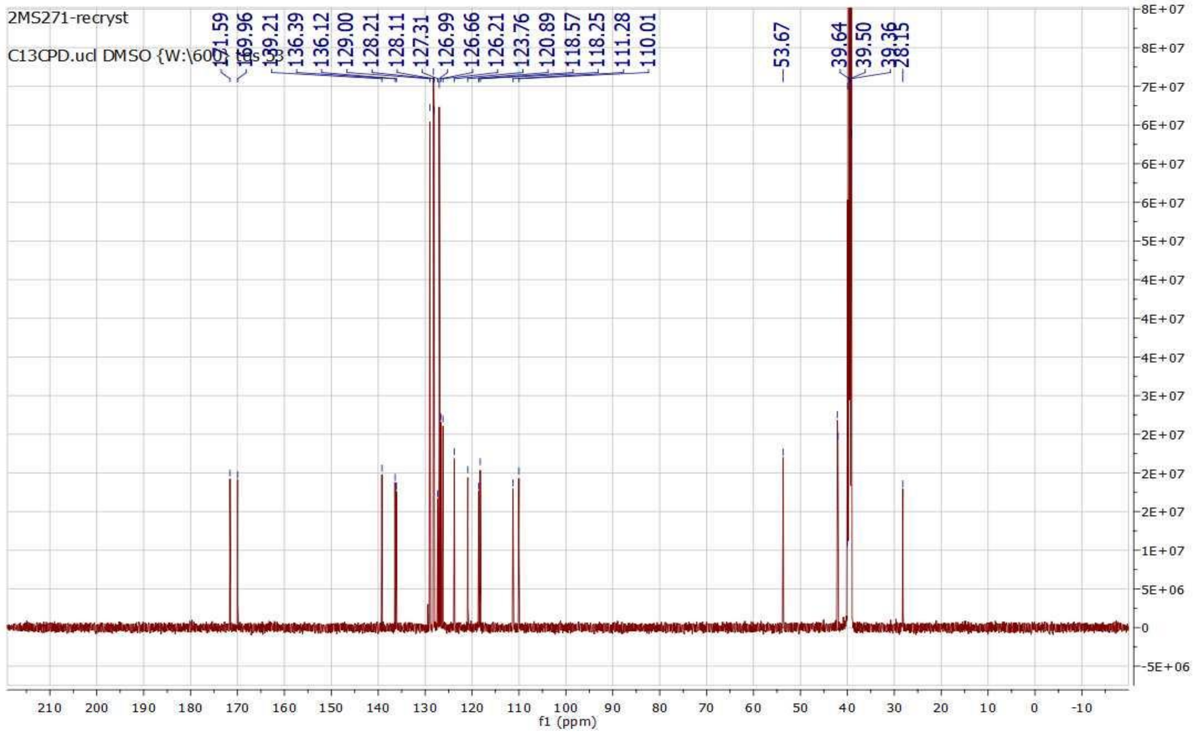

3-Benzyl-2-(4-hydroxy-3-methoxyphenyl)-1-methylimidazolidin-4-one (12a)

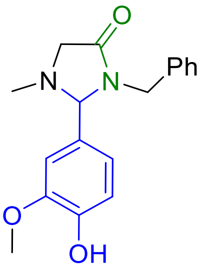

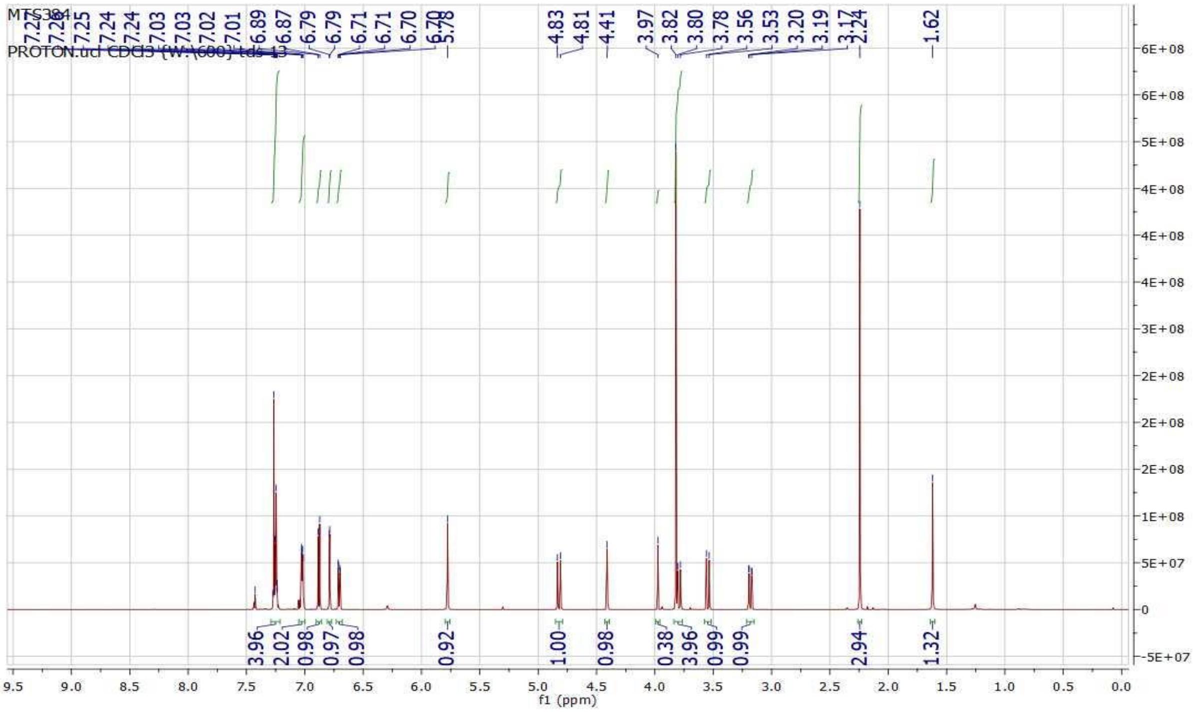

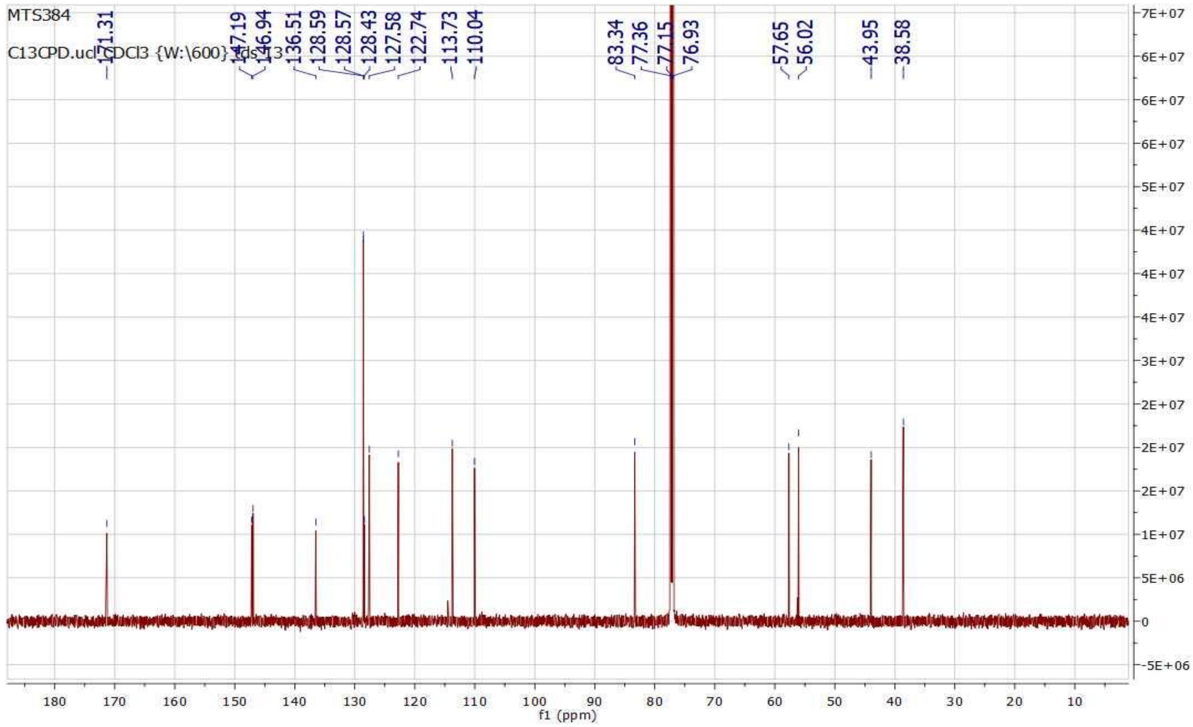

3-Benzyl-1-methyl-2-(1*H*-pyrrol-2-yl)imidazolidin-4-one (12b)

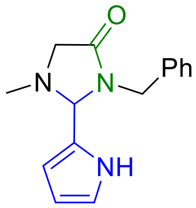

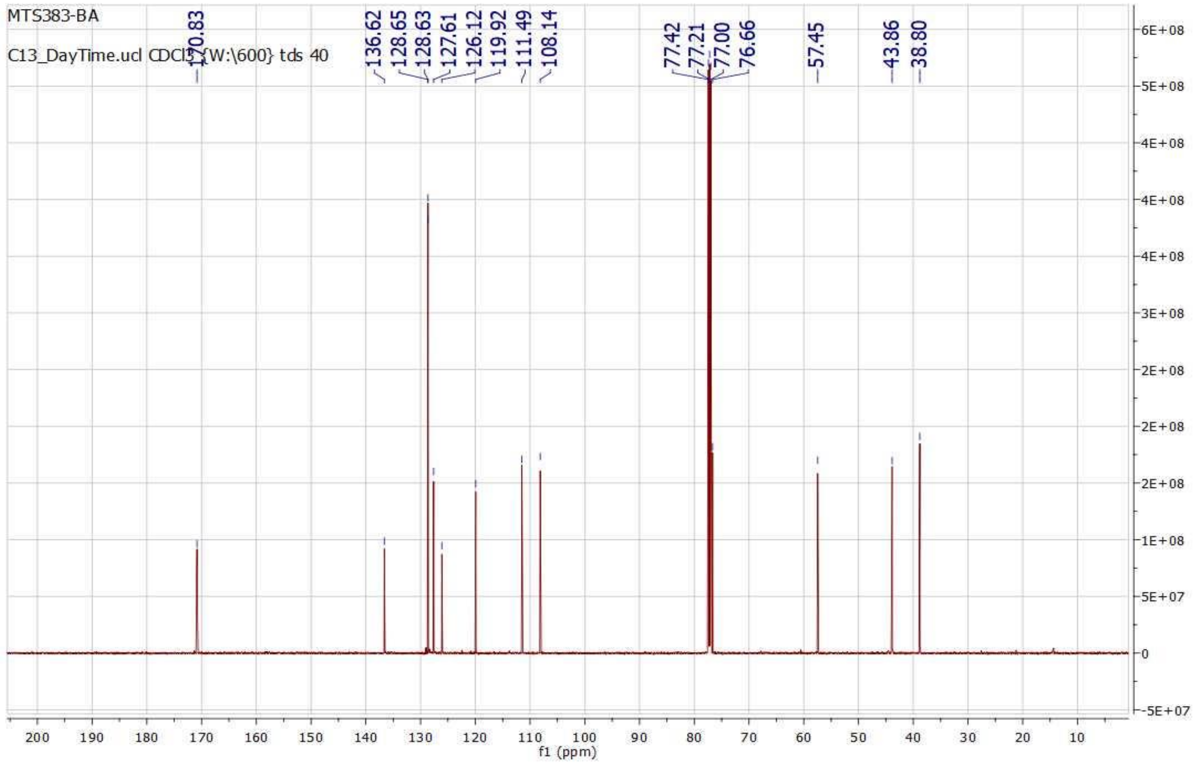

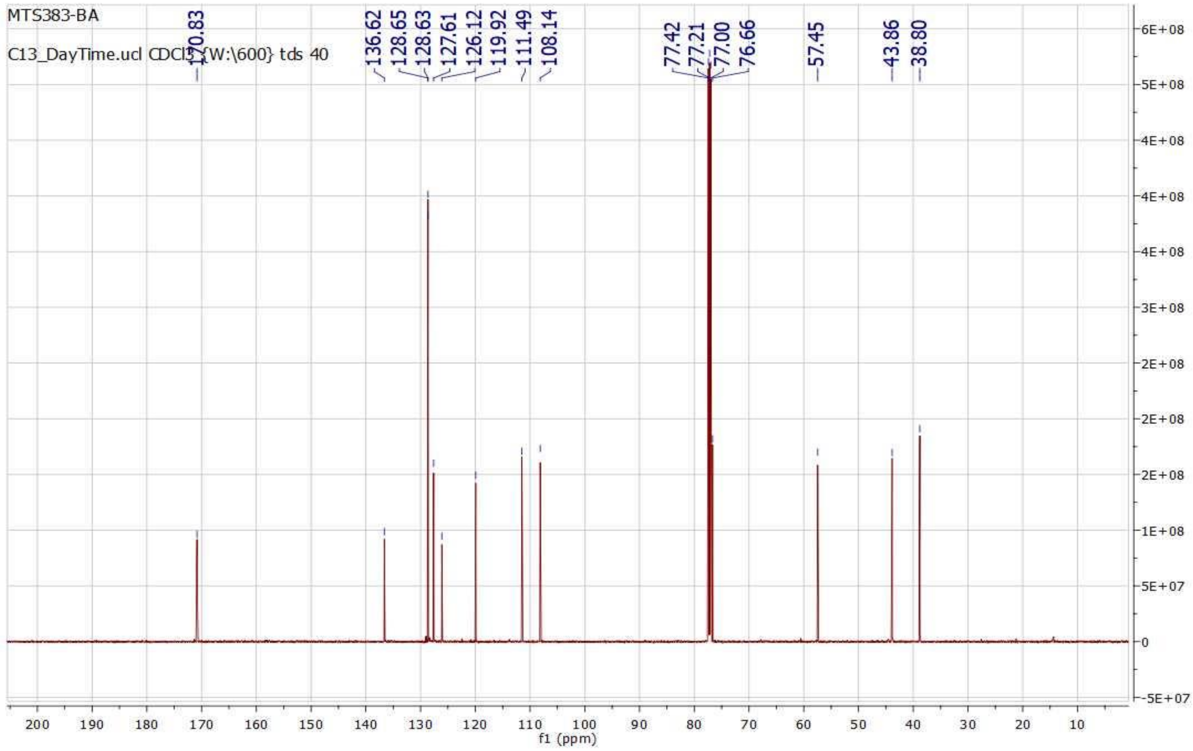

3-Benzyl-2,2-diethyl-1-methylimidazolidin-4-one (12d)

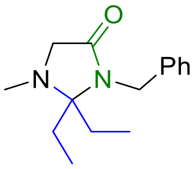

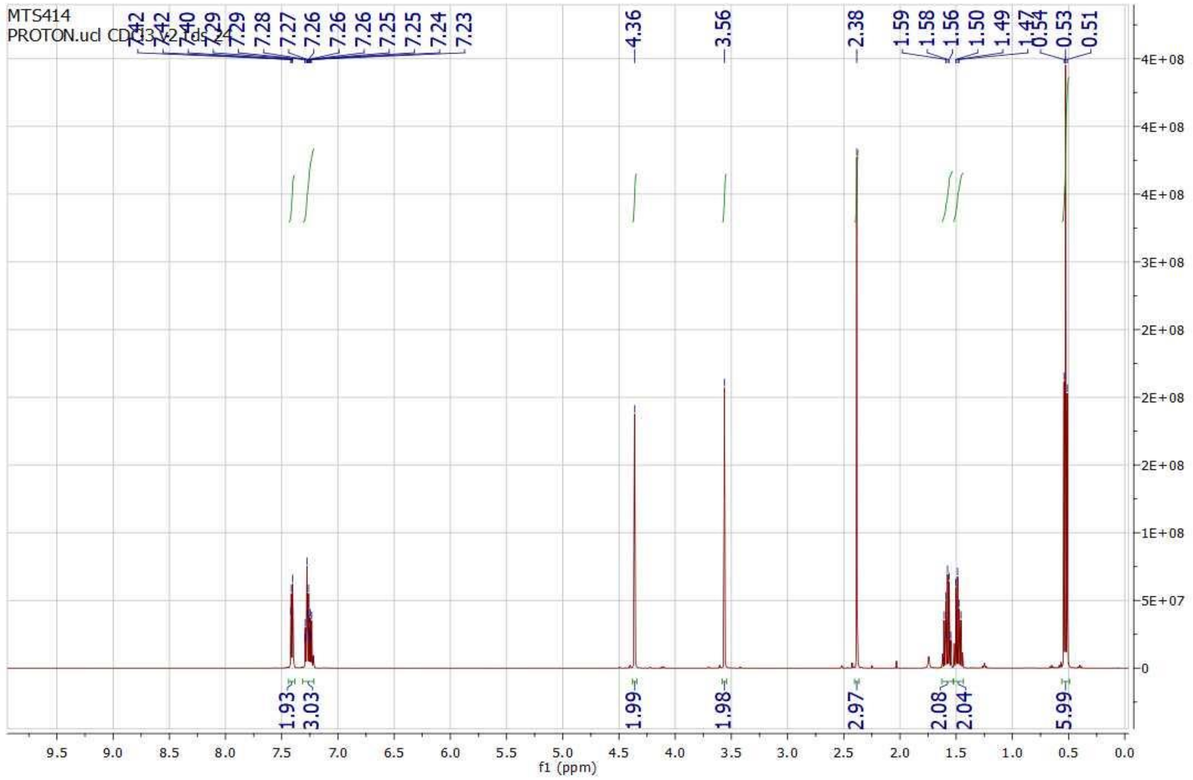

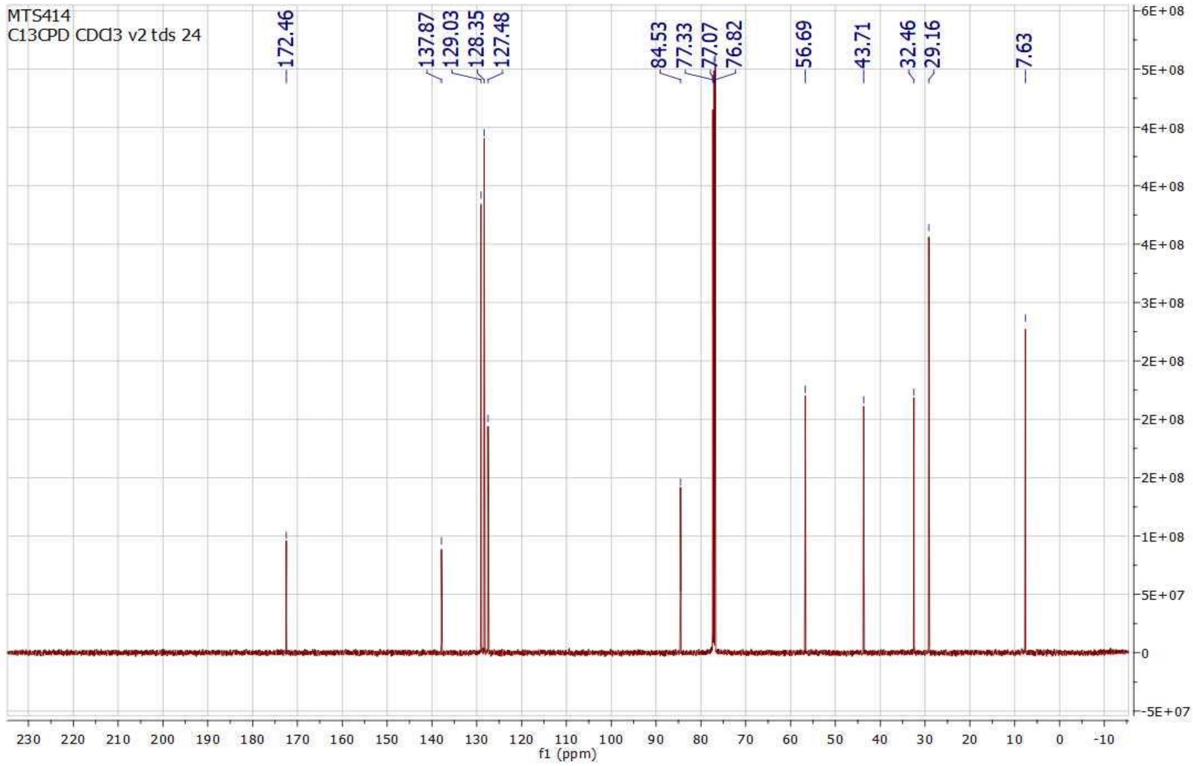

tert-Butyl (*S*)-1,3-dibenzyl-2-oxo-1,4,8-triazaspiro[4.5]decane-8-carboxylate (12f)

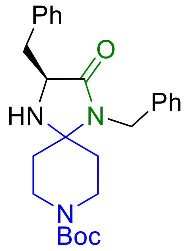

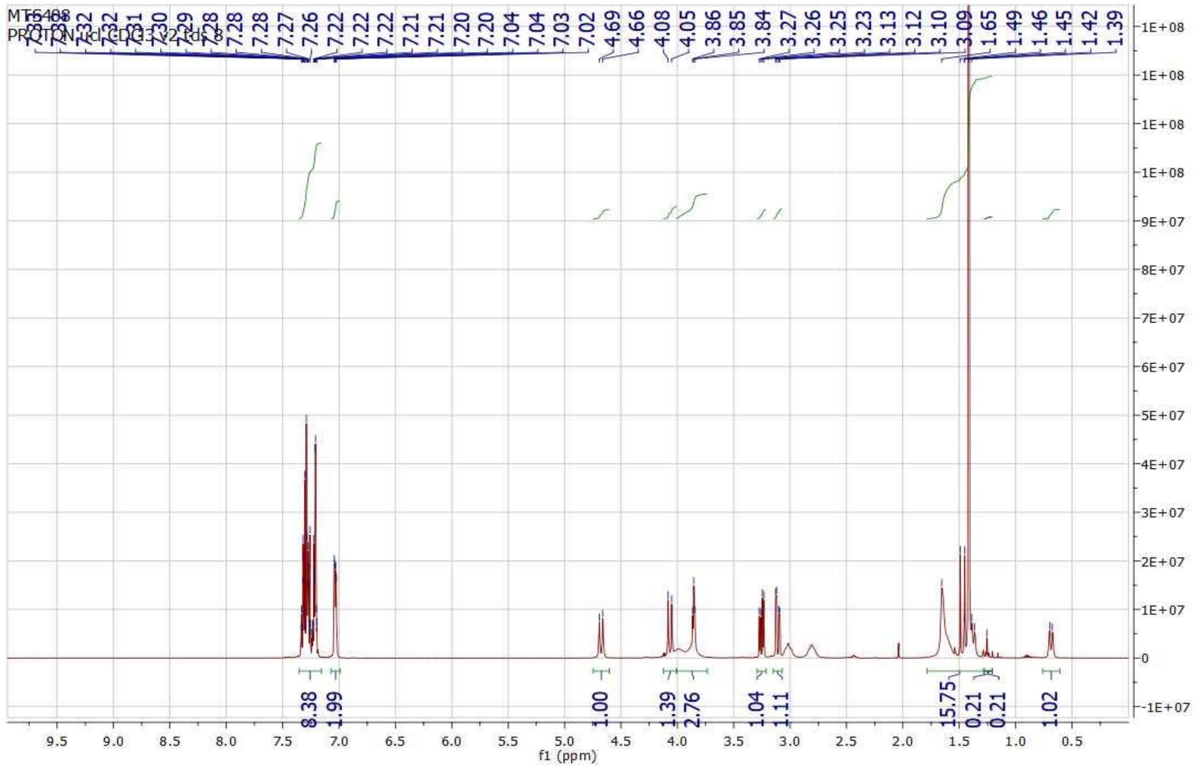

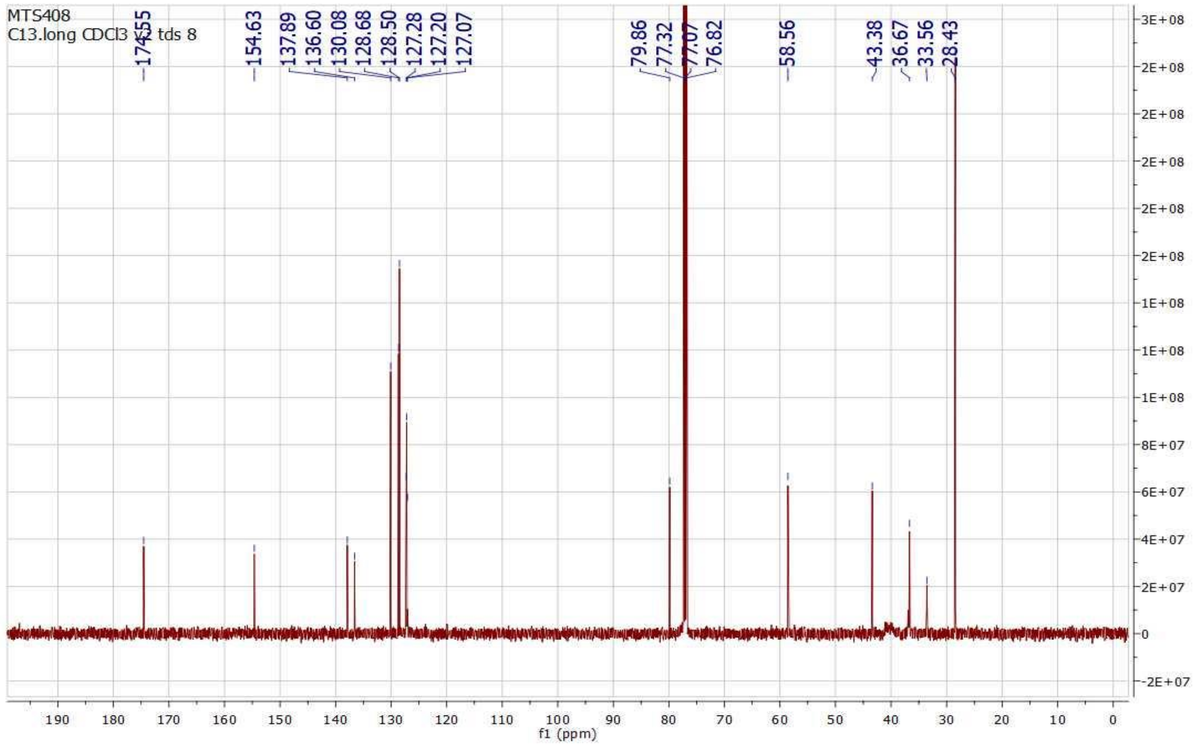

1-Benzyl-8-(tert-butyl)-3-phenyl-1,4-diazaspiro[4.5]decan-2-one (12i)

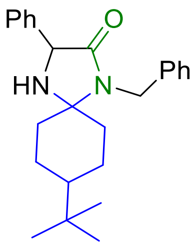

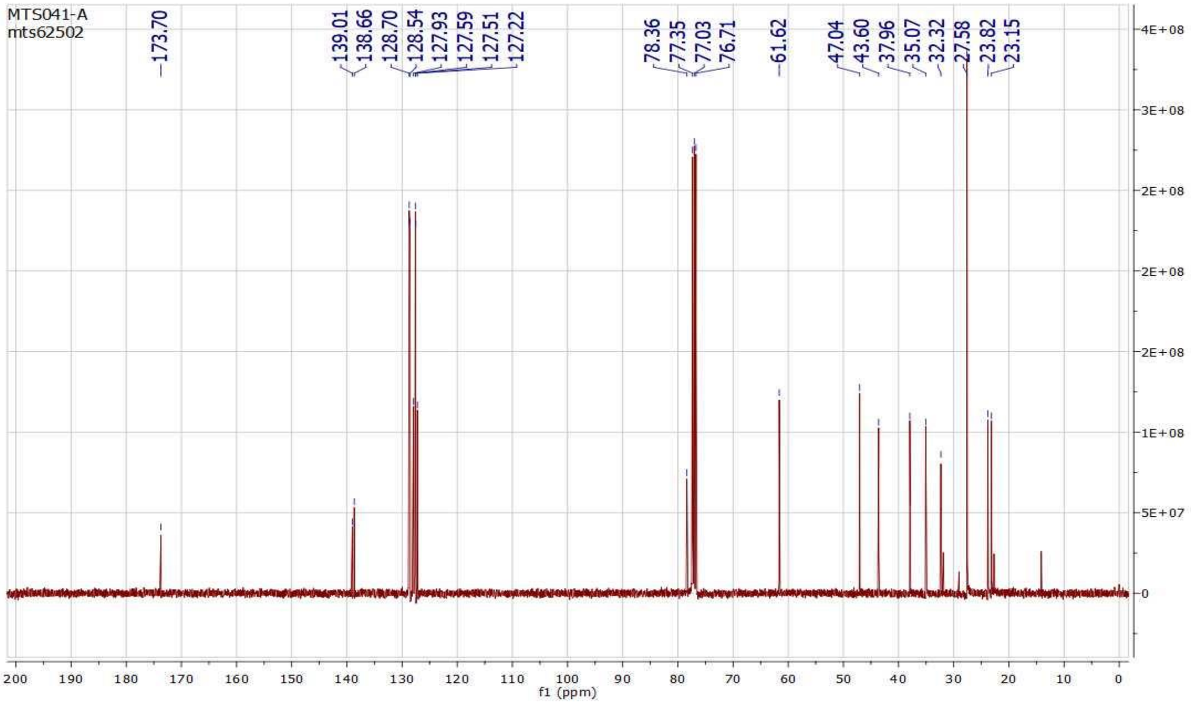

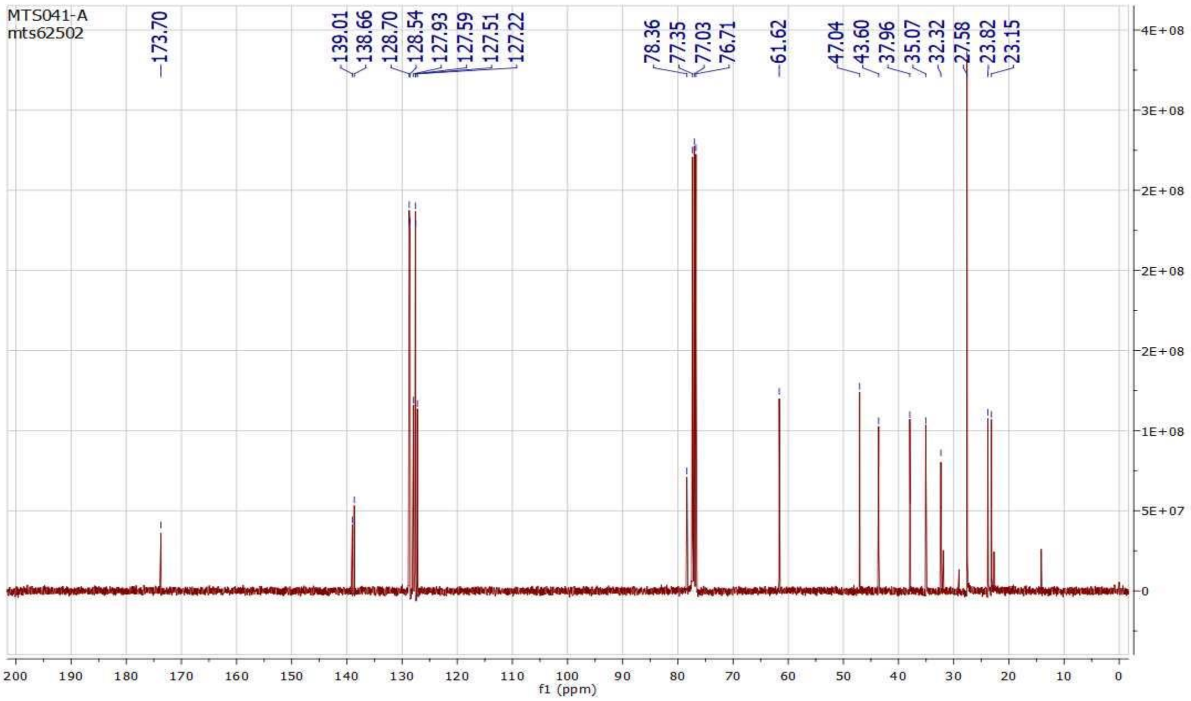

3-(2-(1*H*-Indol-3-yl)ethyl)-2-ethyl-2,5-dimethylimidazolidin-4-one (12j)

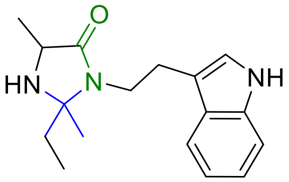

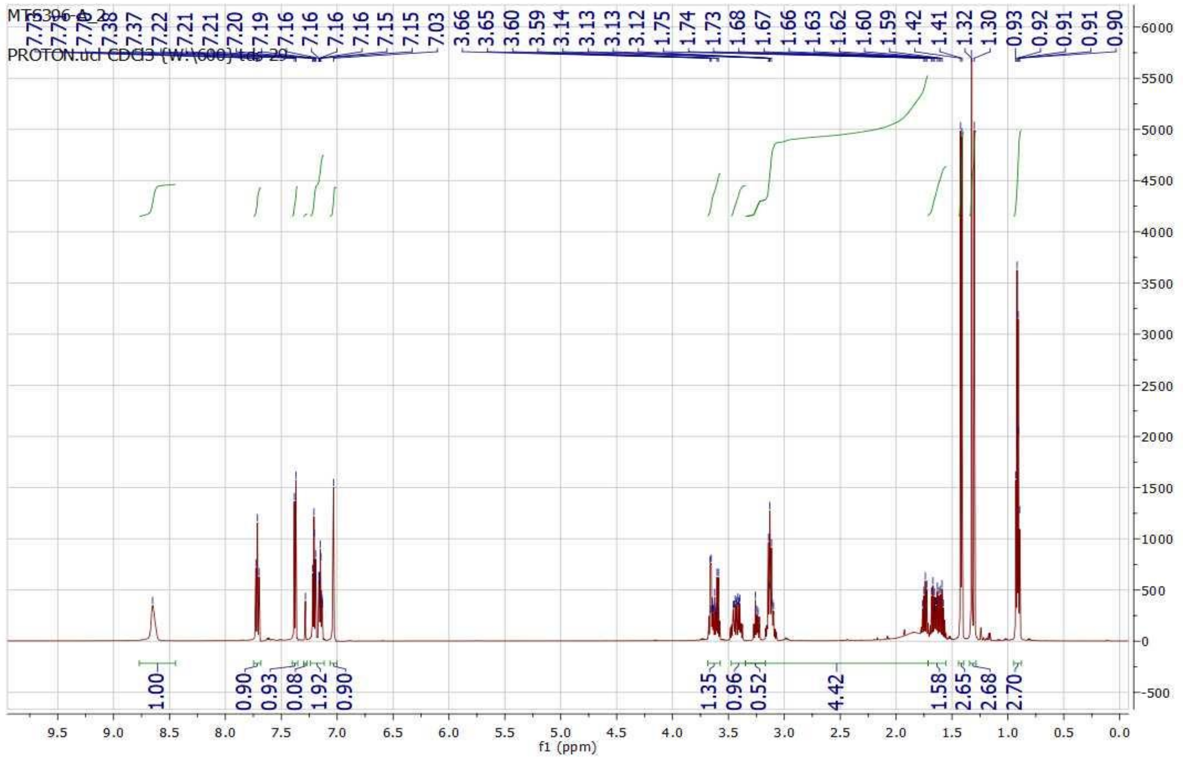

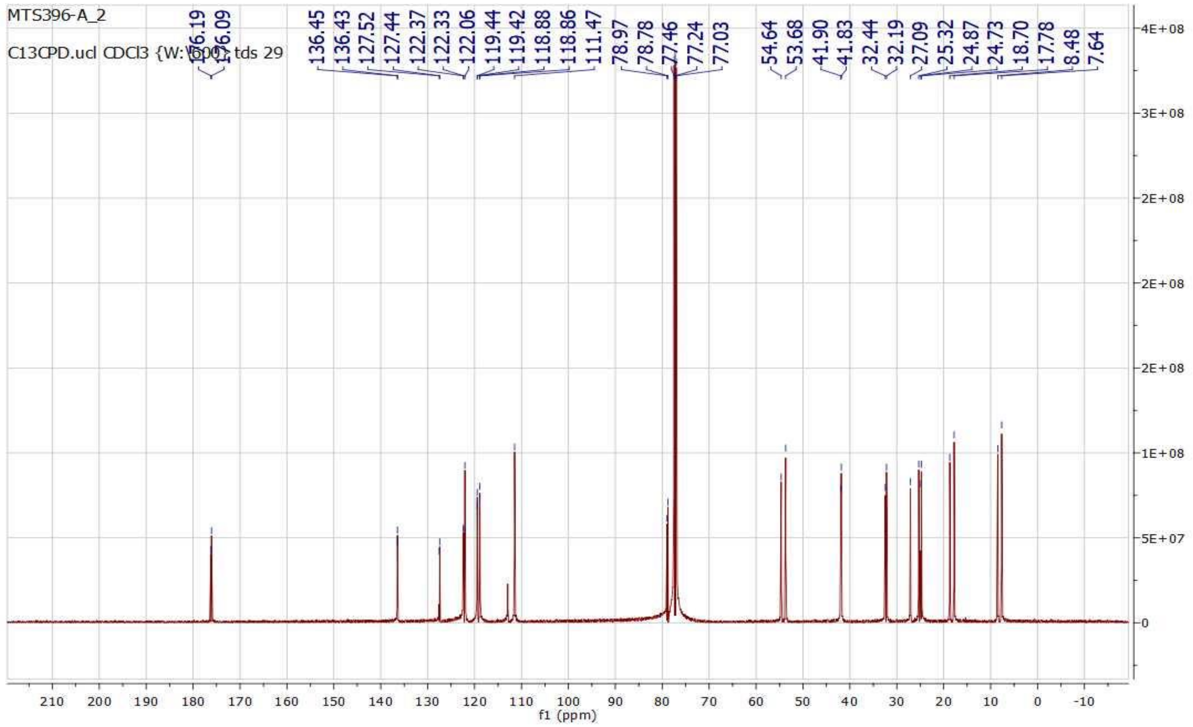

2,2-Diethyl-3-hexyl-1-methylimidazolidin-4-one (12m)

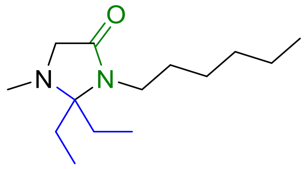

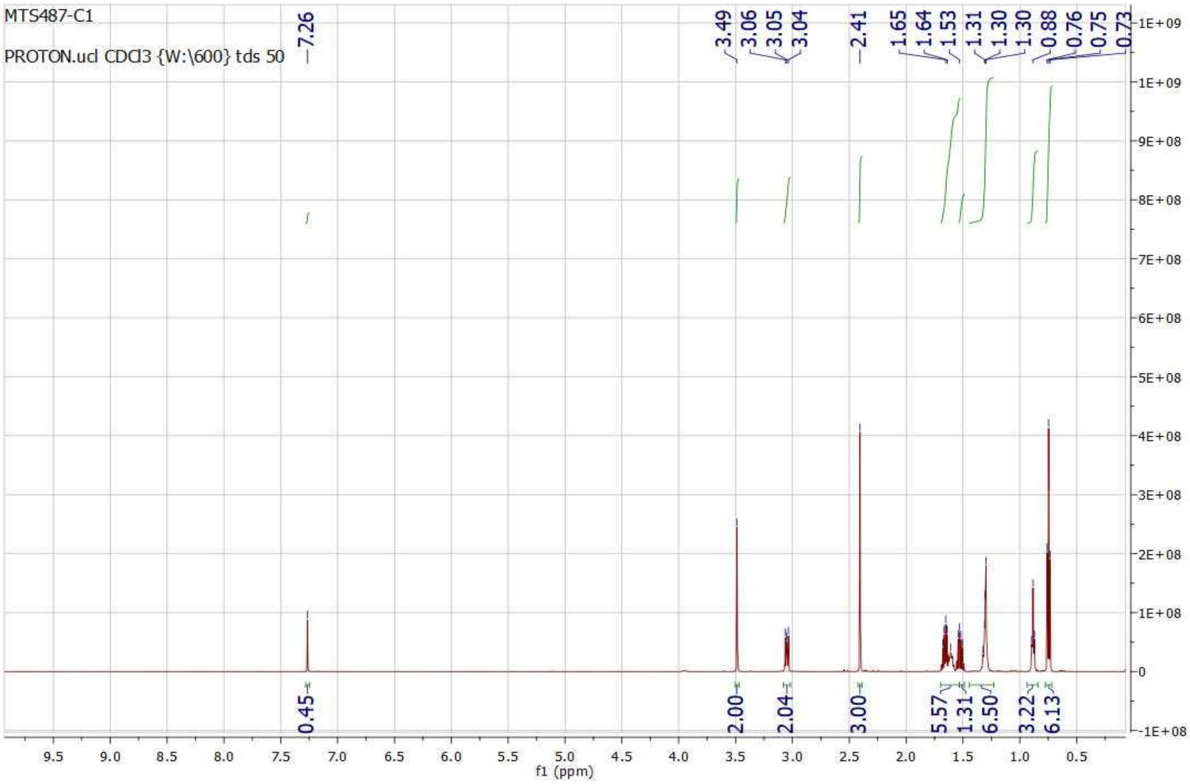

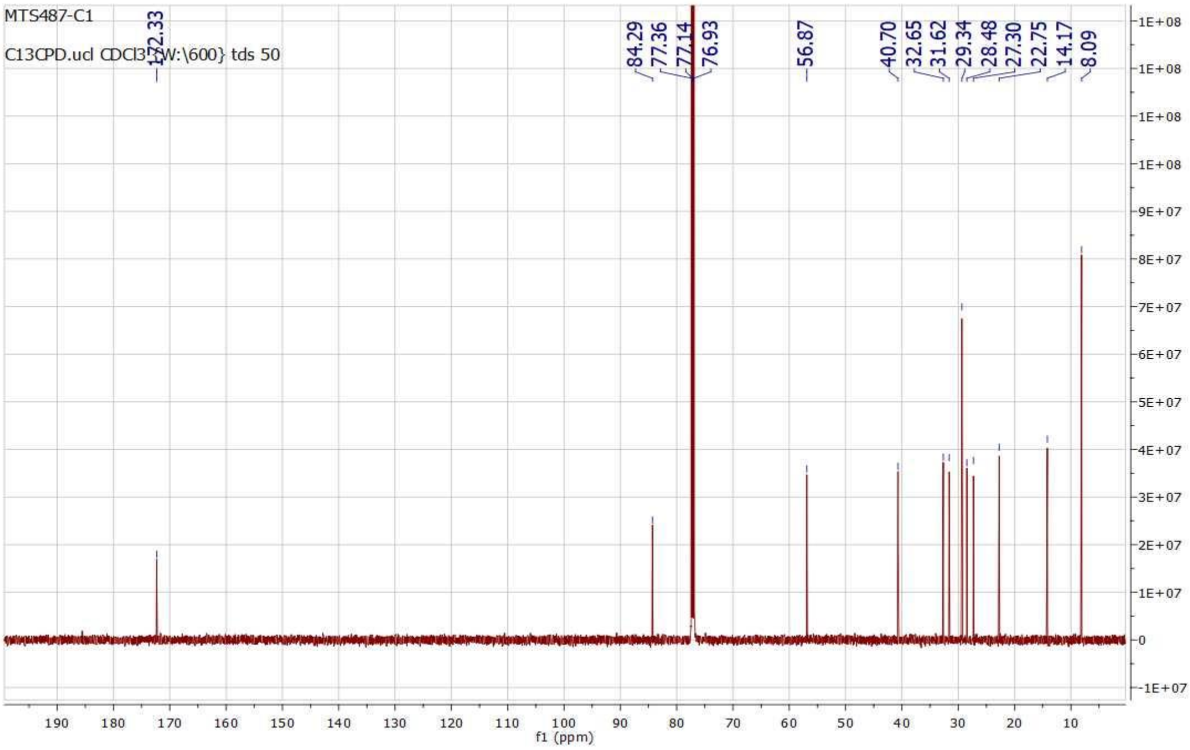

9. ^{1}H and ^{13}C NMR spectra for enantiopurity and HPLC traces

Spectrum of Marfey-derived **1a, 1d, 1f, 1b, 1g, 1h, 1j, 1dd, 6e, 6aa** from catalytic $B(OCH_2CF_3)_3$ presented in previous publication.[16]

Spectrum of Marfey-derived **6a, 6c, 6f, 6j, 6h, 6aa** from stoichiometric $B(OCH_2CF_3)_3$ presented in previous publication.[16]

Chiral HPLC traces for **6z, 12g, 12h** from $B(OCH_2CF_3)_3$ catalysed amidations presented in previous publication.[16]

Spectrum of Marfey-derived **1a** from $Ti(O^{i}Pr_4)$ (Figure 4), >95:5 er

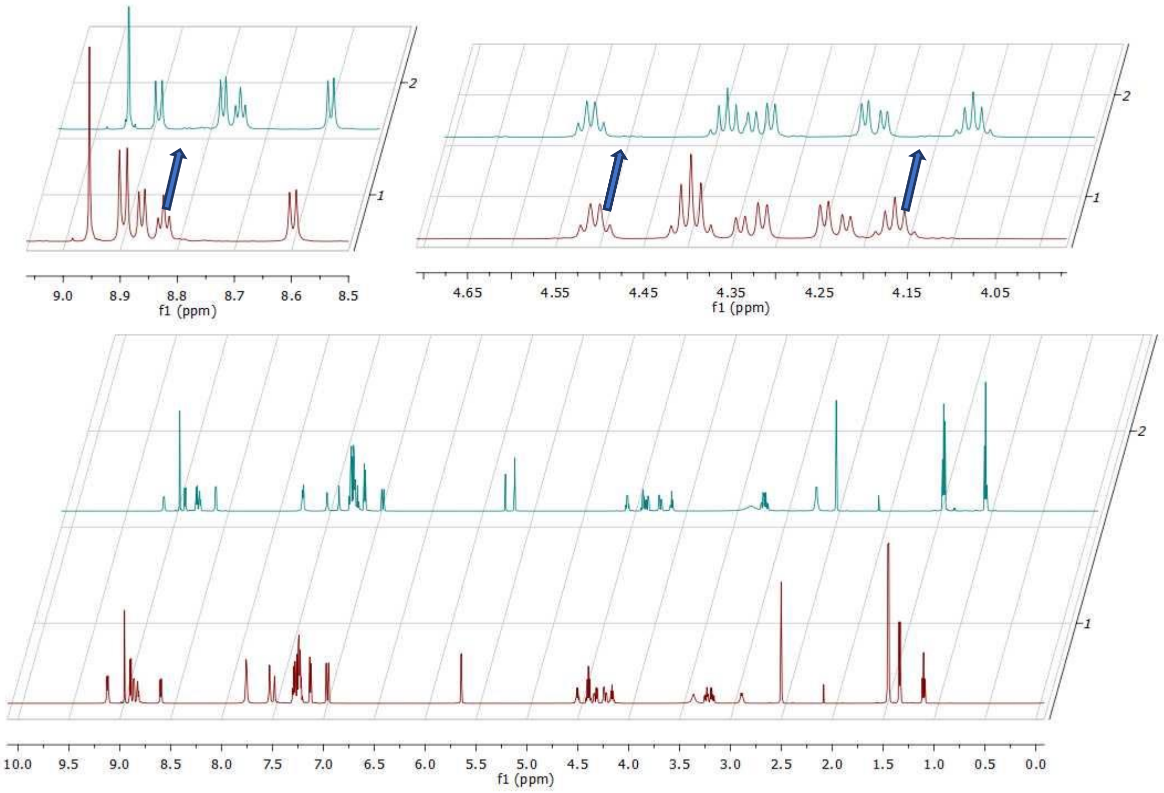

Spectrum of Marfey-derived **1a [scale up],** (Figure 9) >95:5 er

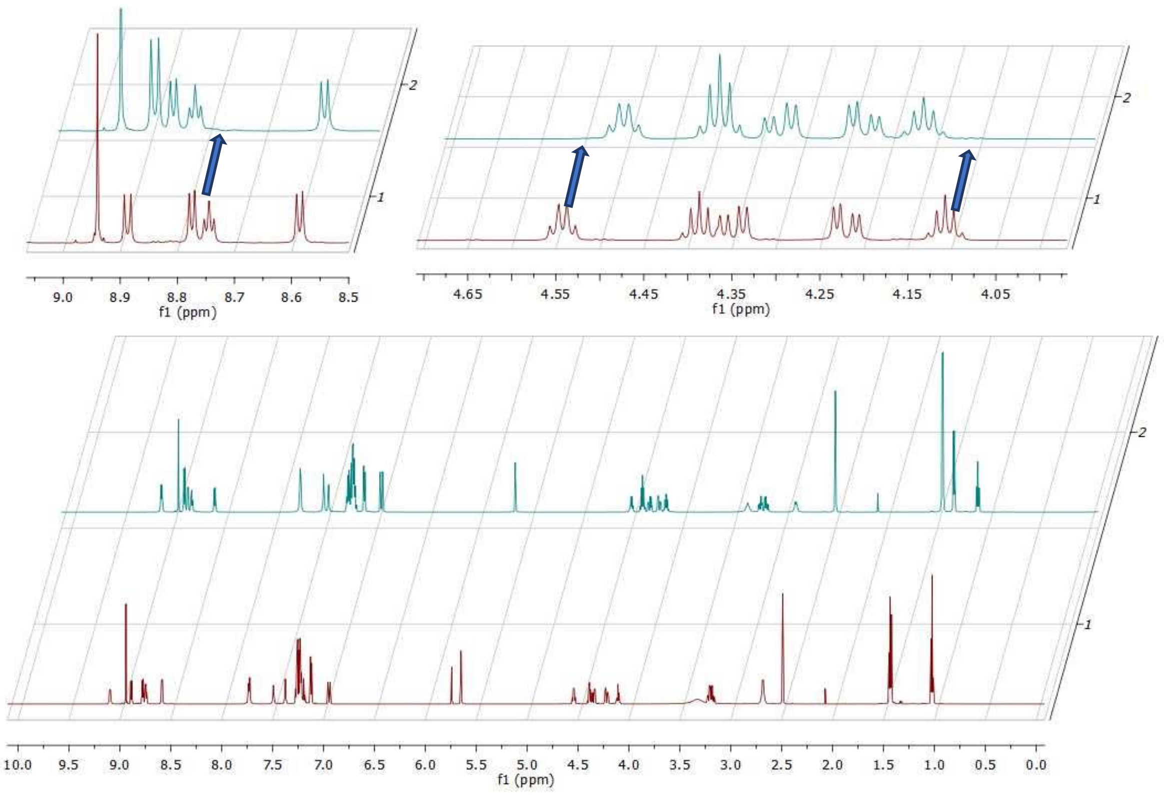

Spectrum of Marfey-derived **1b** from Ti(O^{i}Pr$_4$), (Figure 4) >95:5 er

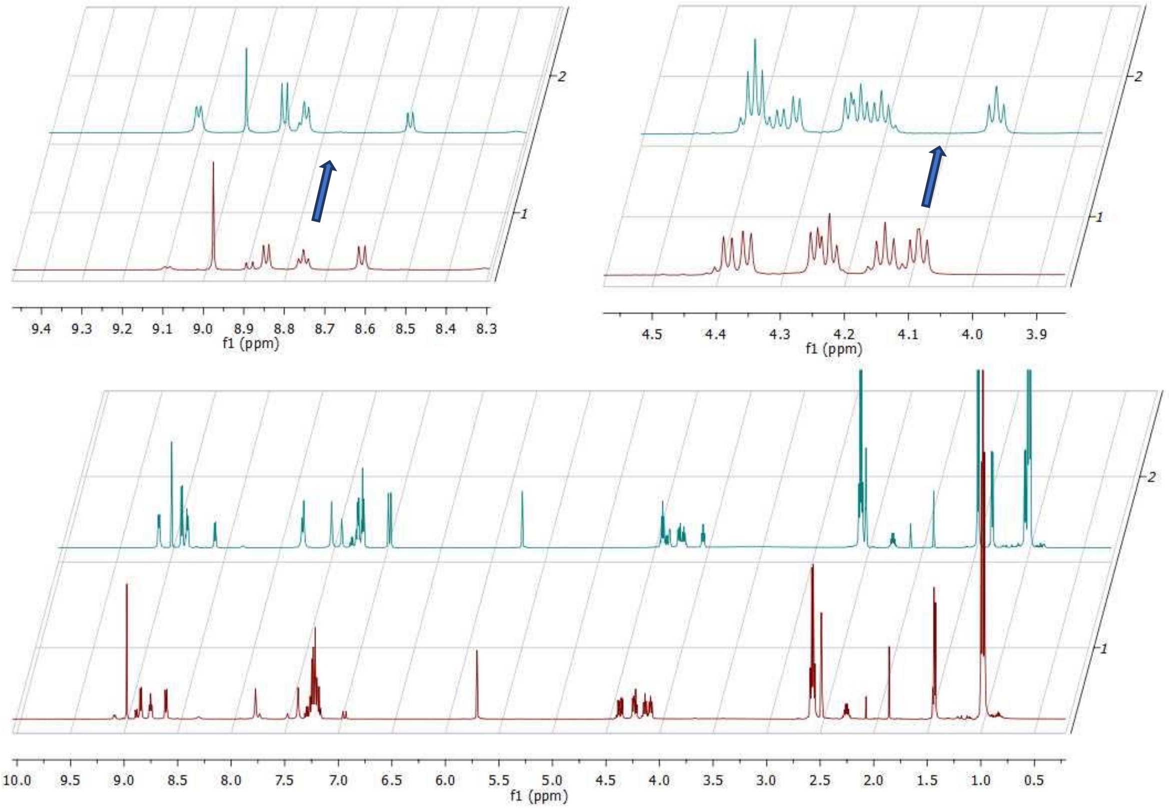

Spectrum of Marfey-derived **1b** [scale-up], (Figure 9) >95:5 er

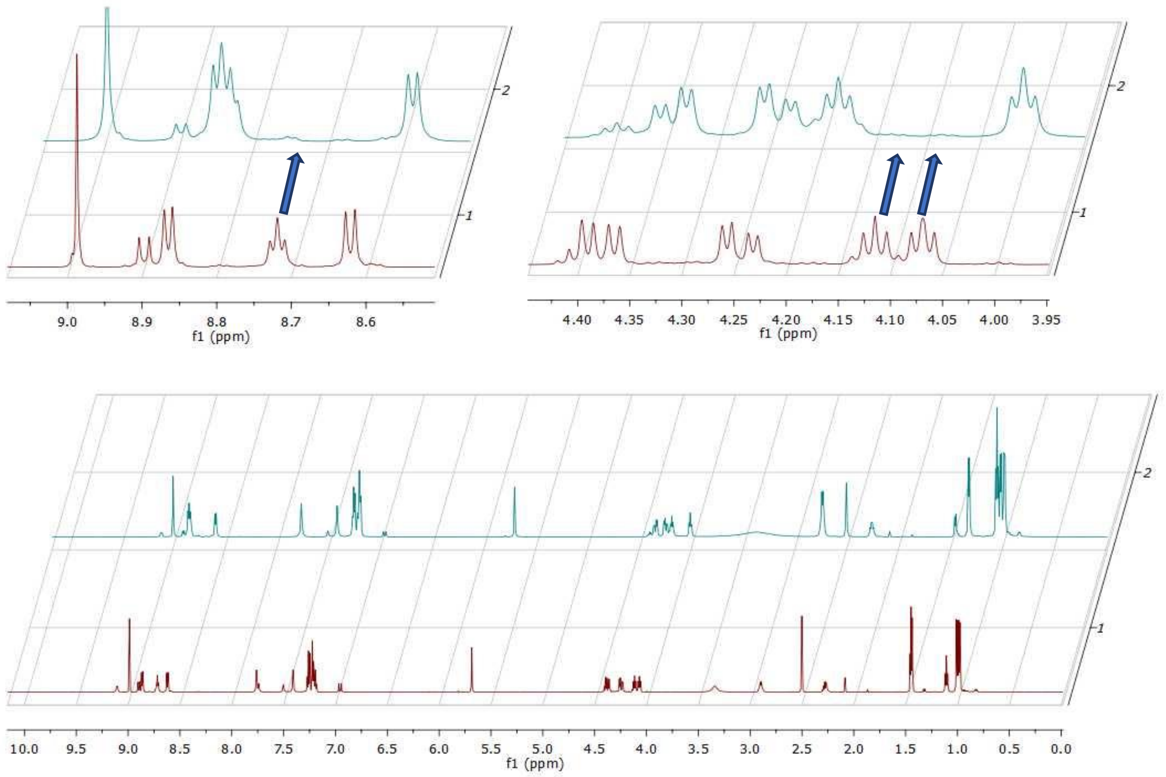

(S)-2-Amino-*N*-benzyl-3-(4-hydroxyphenyl)propanamide (1c) 95:5 er

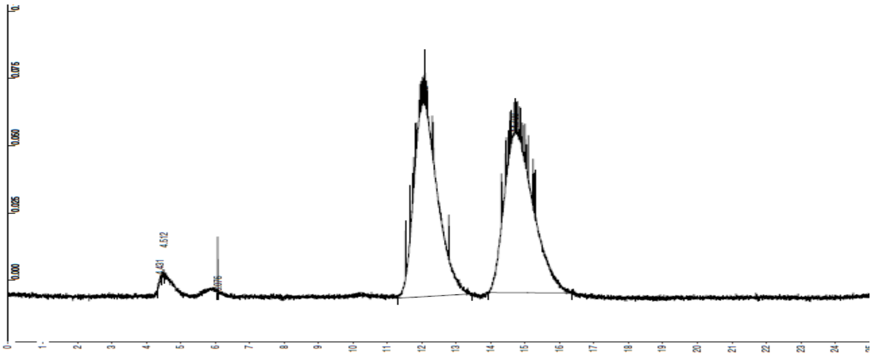

Peak No.	Peak Name	Result ()	Ret. Time (min)	Time Offset (min)	Area (counts)	Sep. Code	Width 1/2 (sec)	Status Codes
1		0.1	4.431	0.000	8230	BB	7.8	
2		0.1	4.512	0.000	5250	BB	0.0	
3		0.1	6.076	0.000	6877	BB	0.2	
4		45.0	12.092	0.000	3536530	BB	35.5	
5		45.6	14.708	0.000	3587629	BB	46.0	
	Totals:	99.3		0.000	7862404			

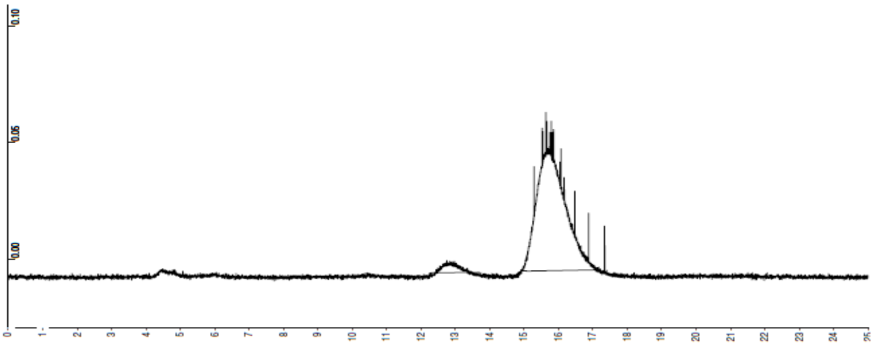

Peak No.	Peak Name	Result ()	Ret. Time (min)	Time Offset (min)	Area (counts)	Sep. Code	Width 1/2 (sec)	Status Codes
1		3.6056	12.752	0.000	115483	BB	15.8	
2		96.1135	15.635	0.000	3078409	BB	42.3	
3		0.2809	64.348	0.000	8998	BB	0.2	
	Totals:	100.0000		0.000	3202890			

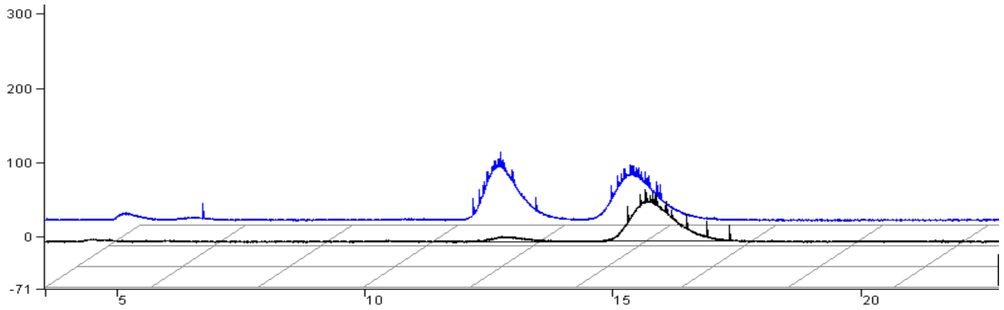

(*S*)-*N*-Benzyl-5-oxopyrrolidine-2-carboxamide 1n

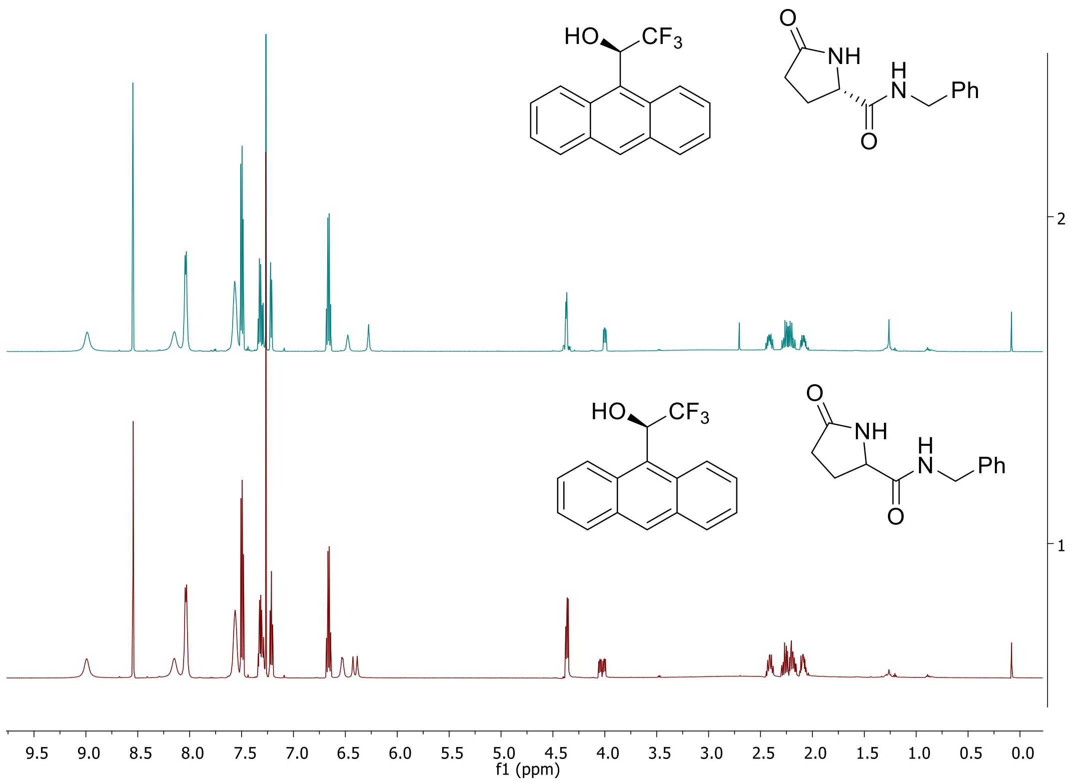

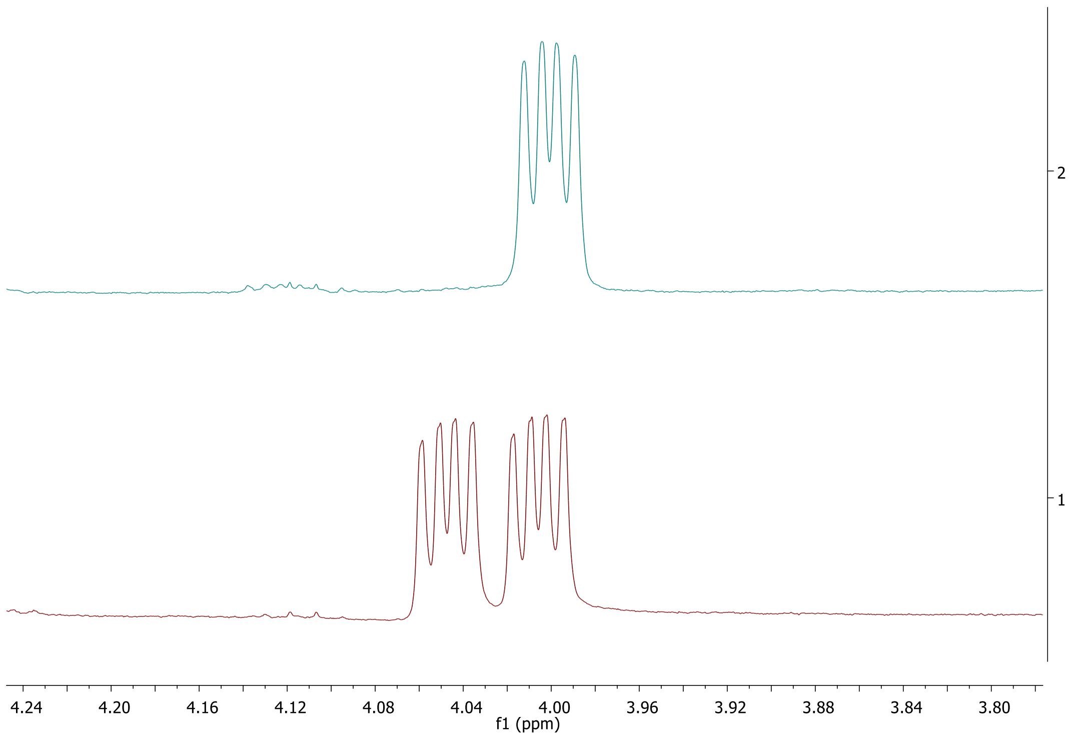

Spectrum of Marfey-derived **1d** [scale-up], (Figure 9) 95:5 er

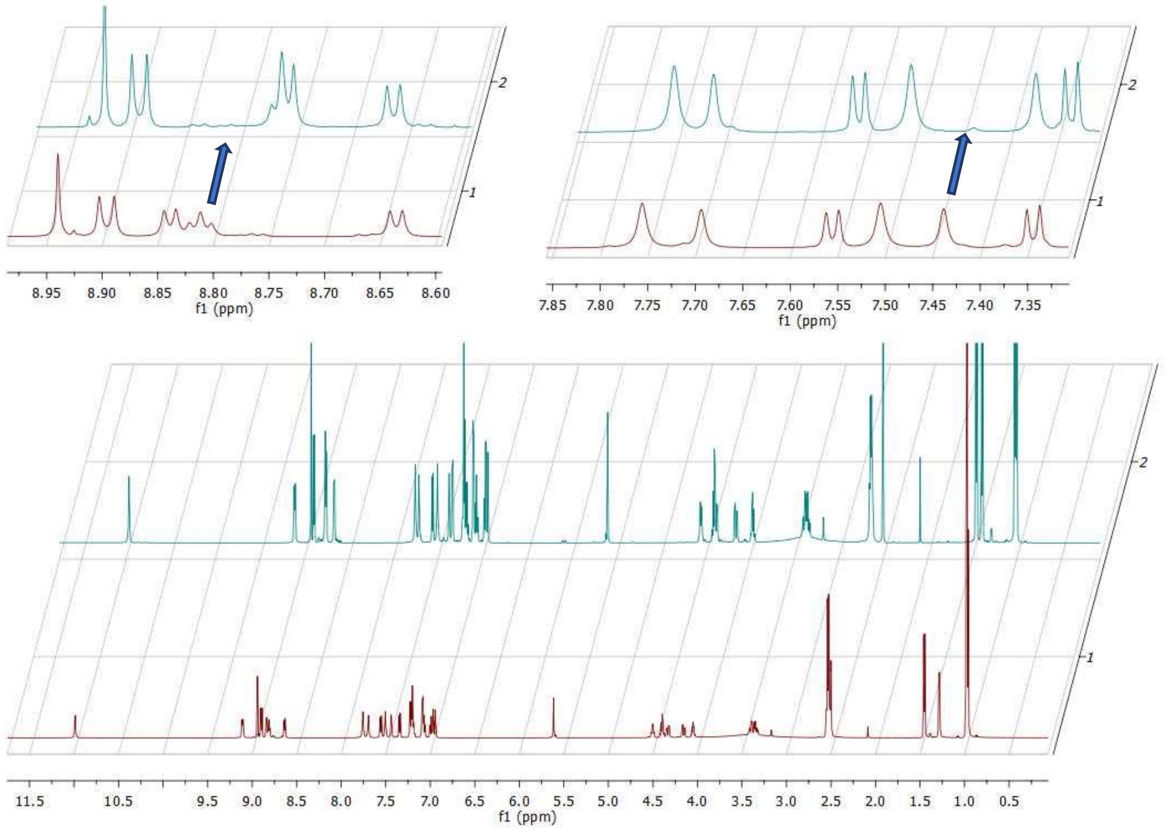

Spectrum of Marfey-derived **1o** 73:27 er

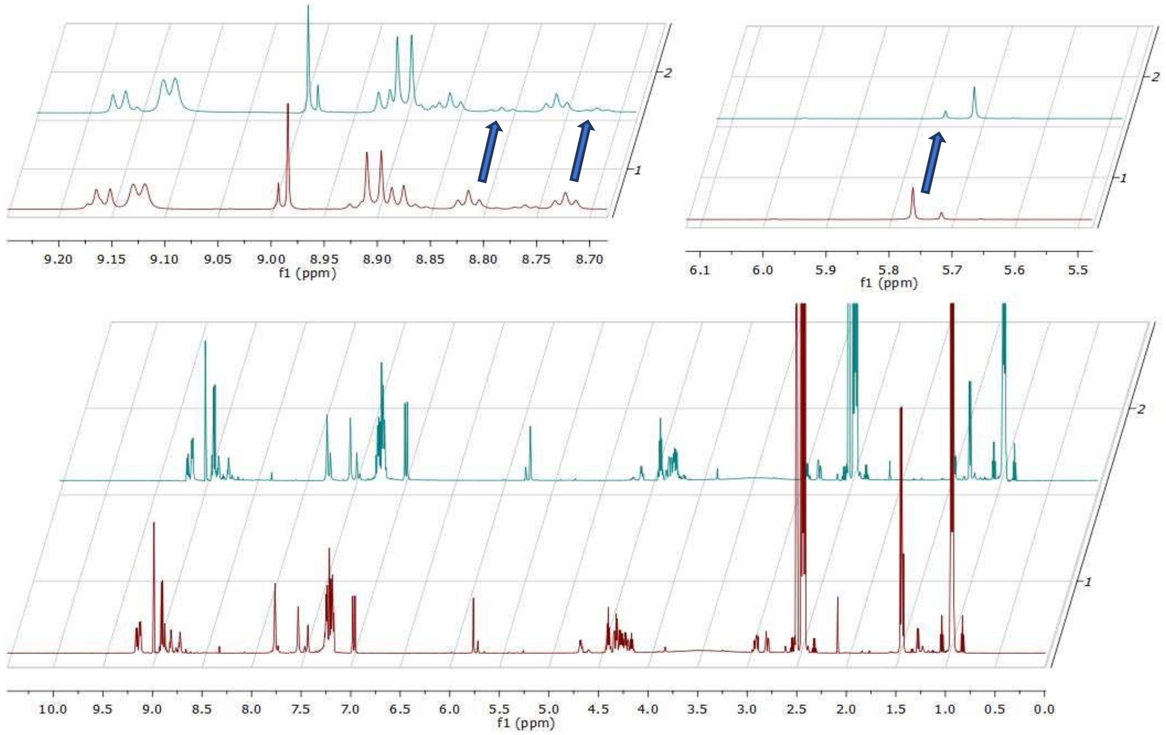

Spectrum of Marfey-derived **1y**, 79% yield, 75:25 er

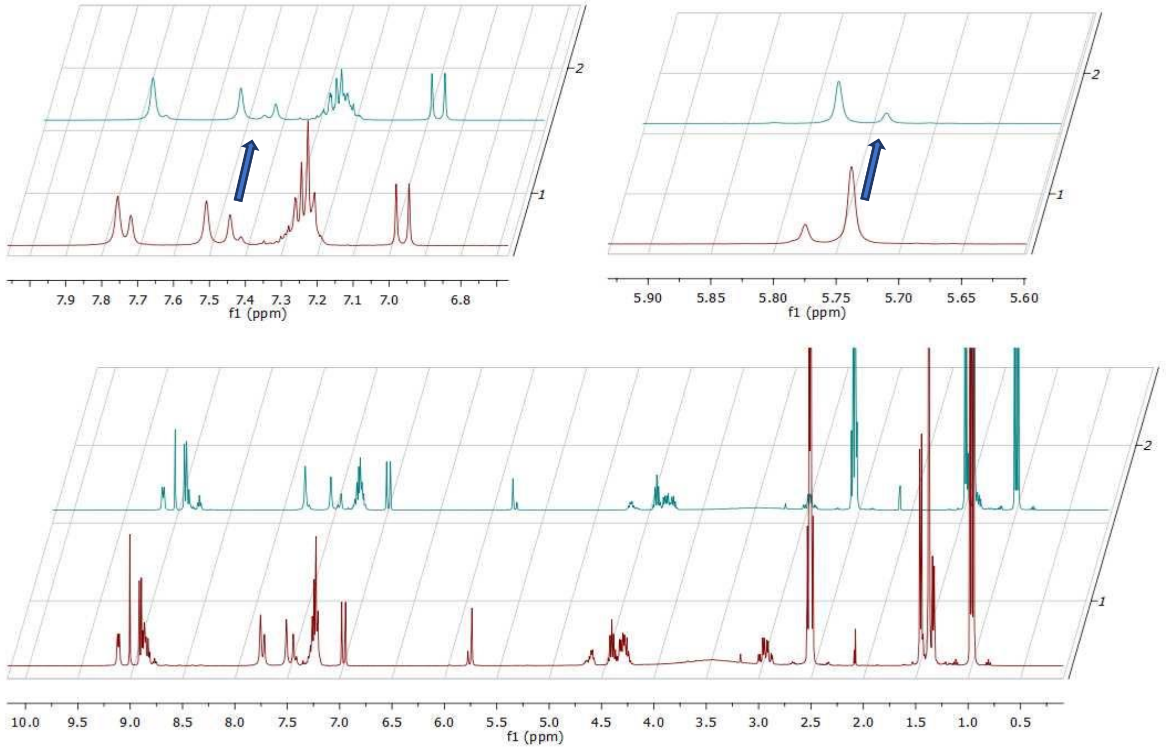

Spectrum of Marfey-derived **1y**, with 10 mol% $B(OCH_2CF_3)_3$, 65% yield, 87:13 er

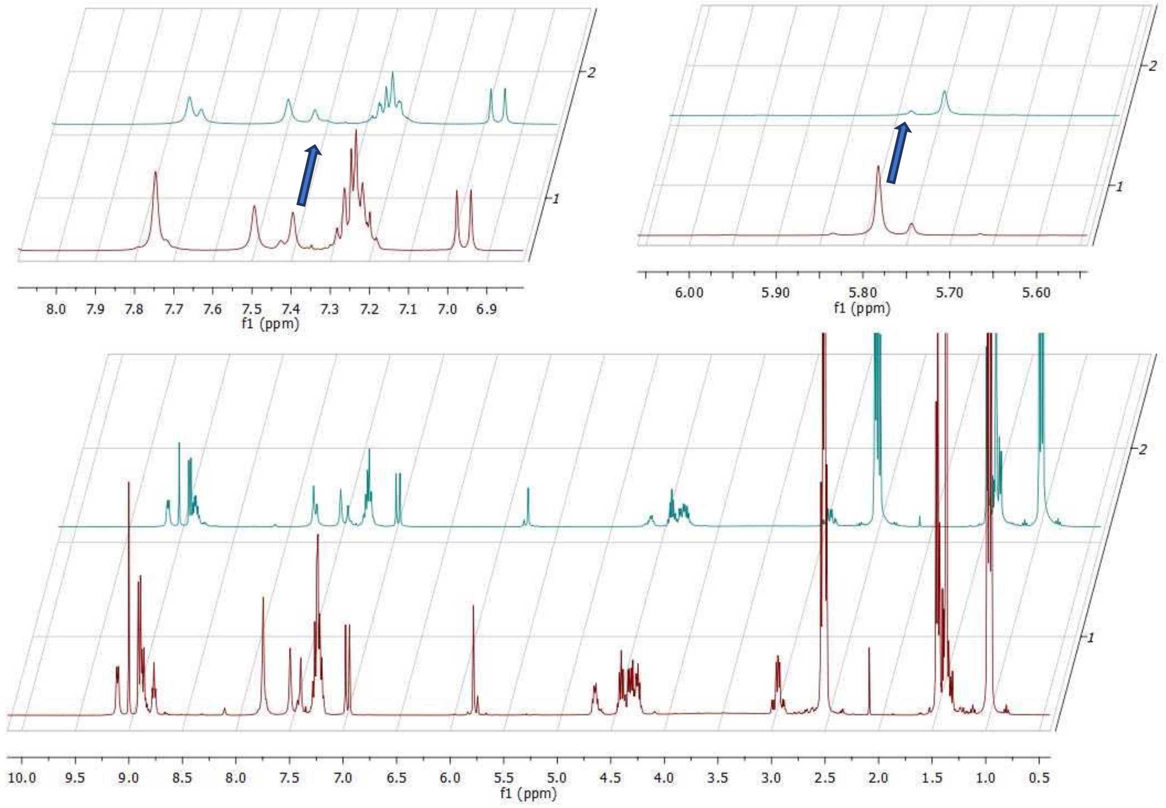

Spectrum of Marfey-derived **1y**, with 8 h reaction time, 77%, 84:16 er

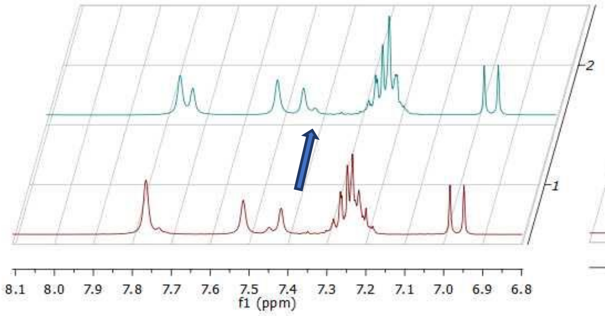

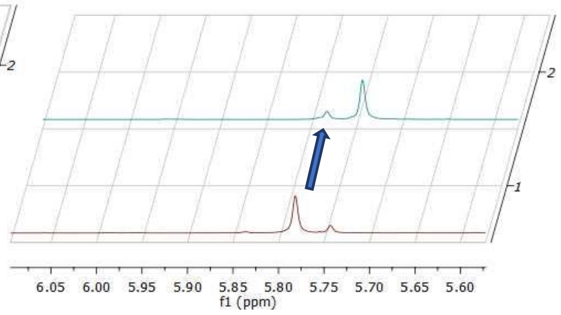

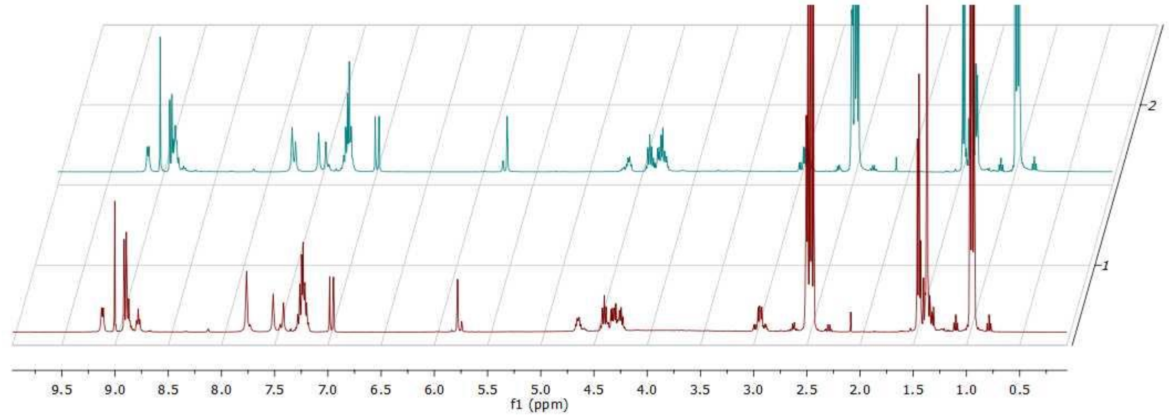

Spectrum of Marfey-derived **1z**, 91%, 74:26 er

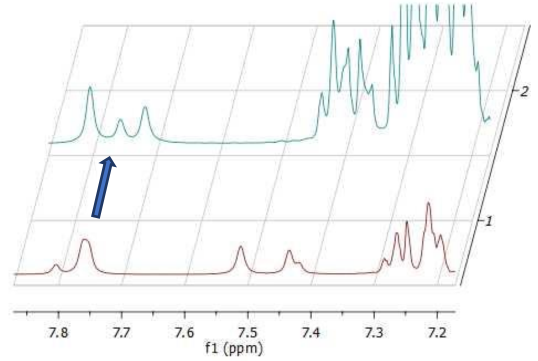

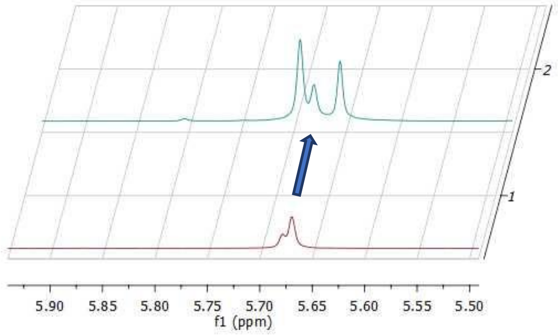

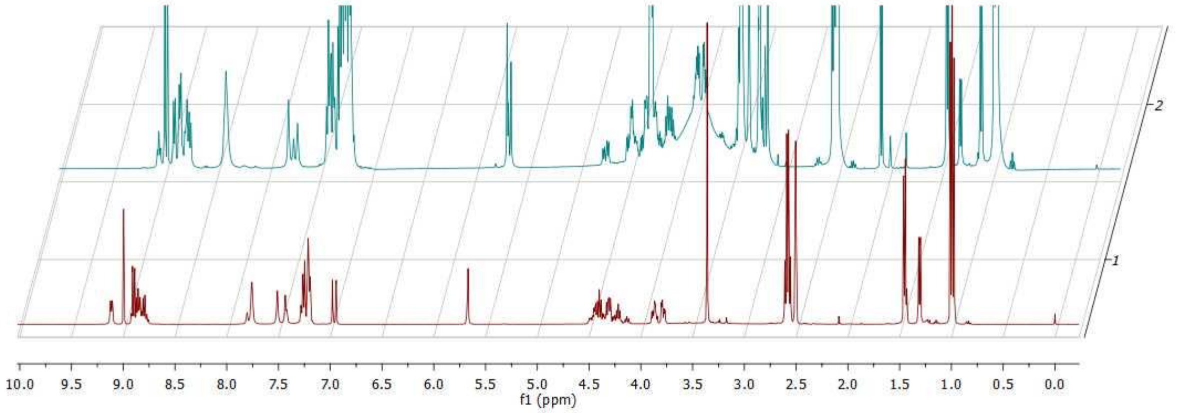

Spectrum of Marfey-derived **1z**, with 10 mol% $B(OCH_2CF_3)_3$, 84%, 75:25 er

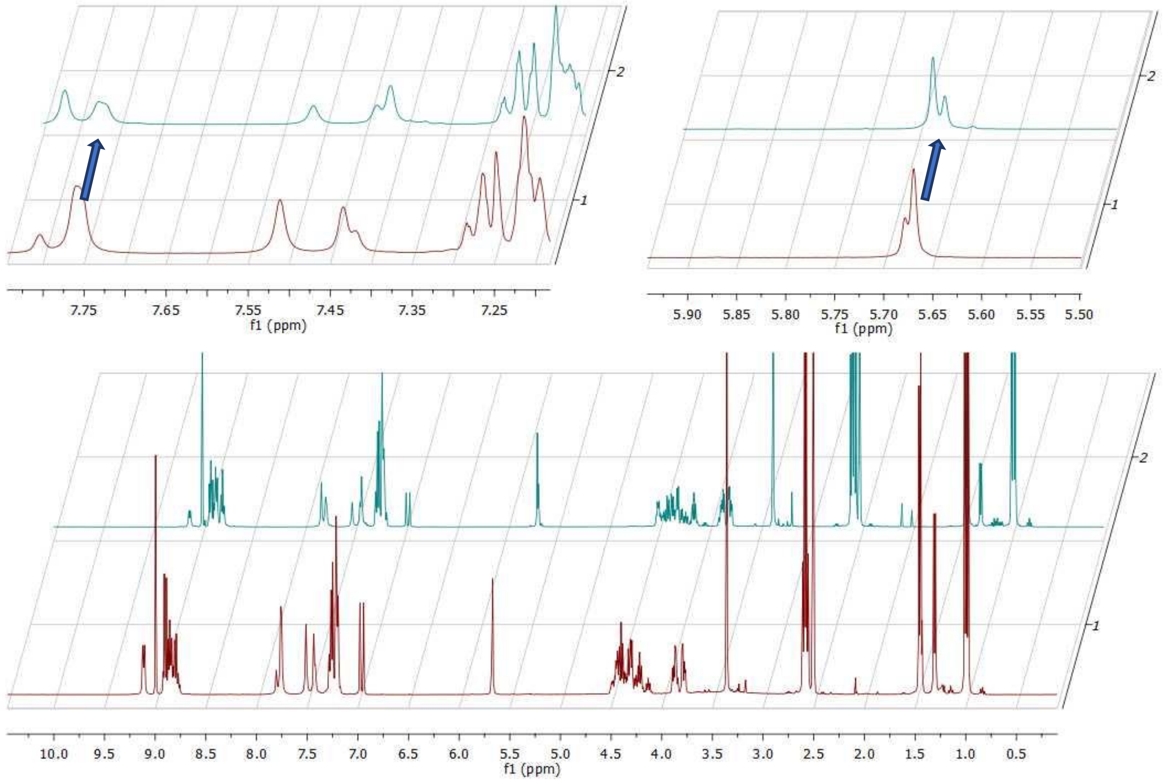

Spectrum of Marfey-derived **starting material of 1z,** 94:6 er

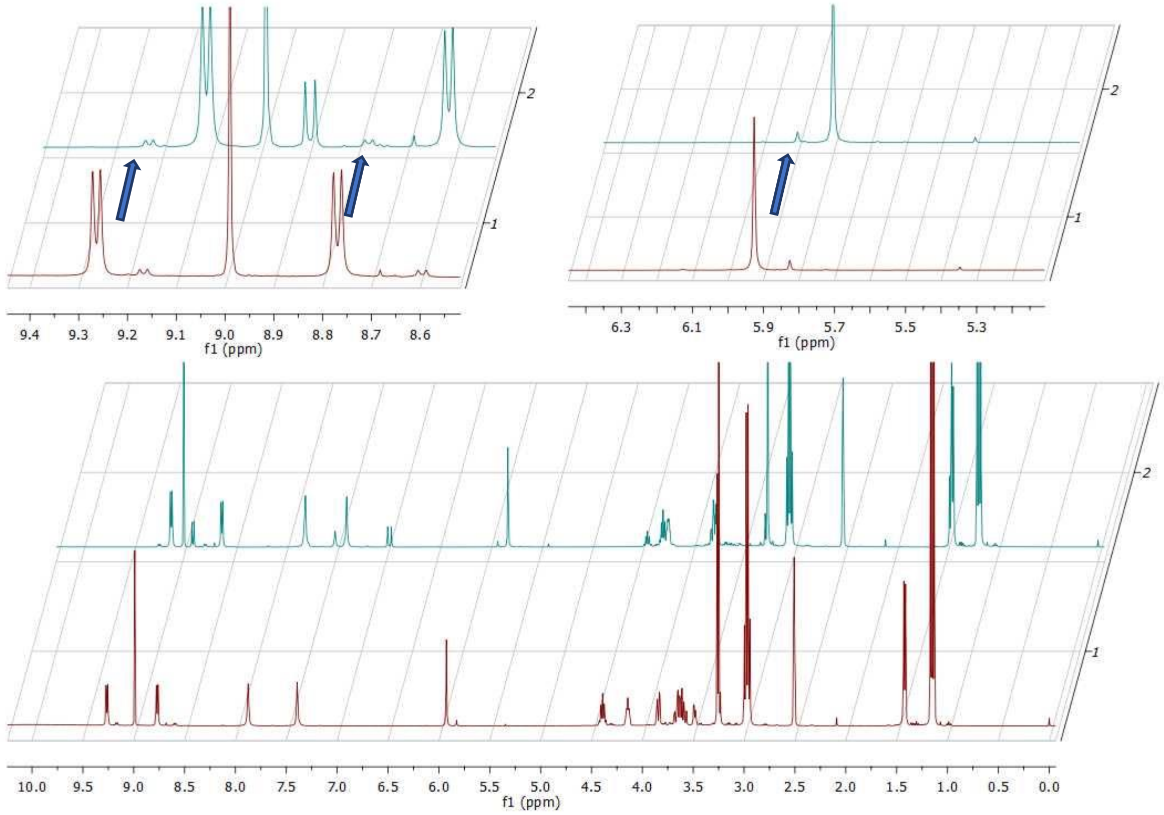

Spectrum of Marfey-derived **1bb**, er 67:33

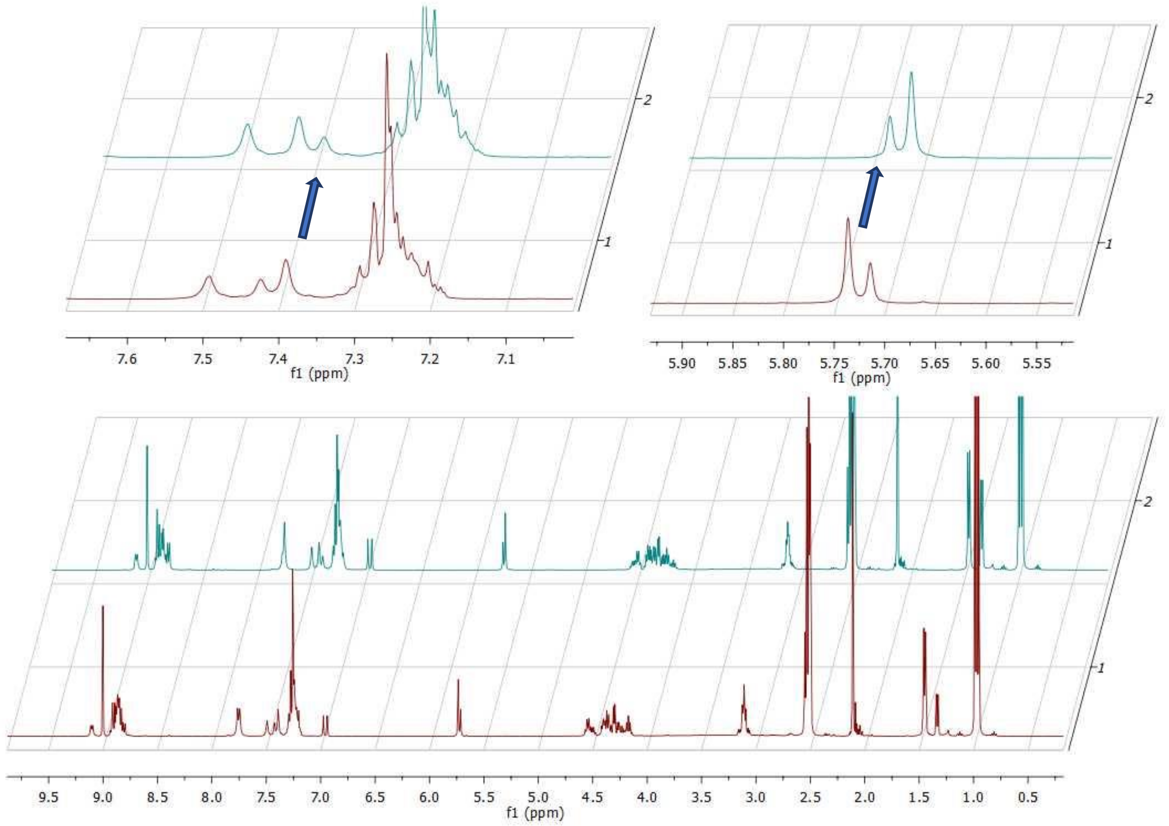

Spectrum of Marfey-derived **1ee**, 50:50 er

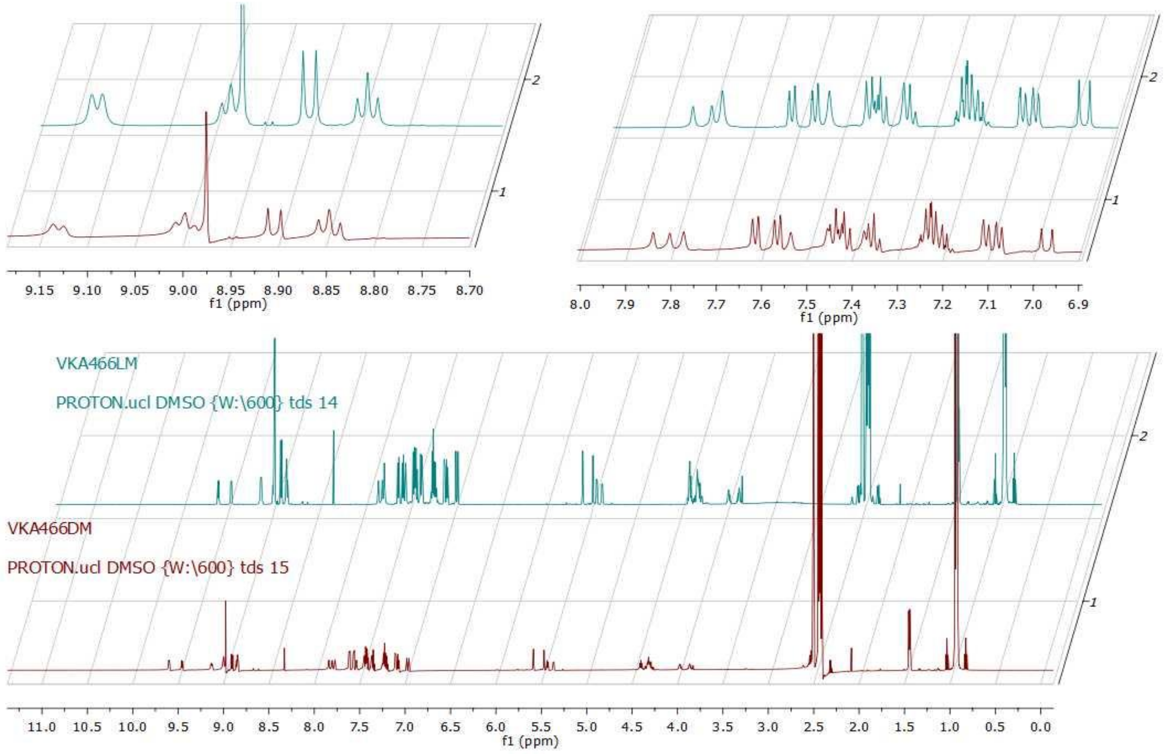

Spectrum of Marfey-derived **2n** from $Ti(OiPr)_4$ (Table 4), 80:20 er

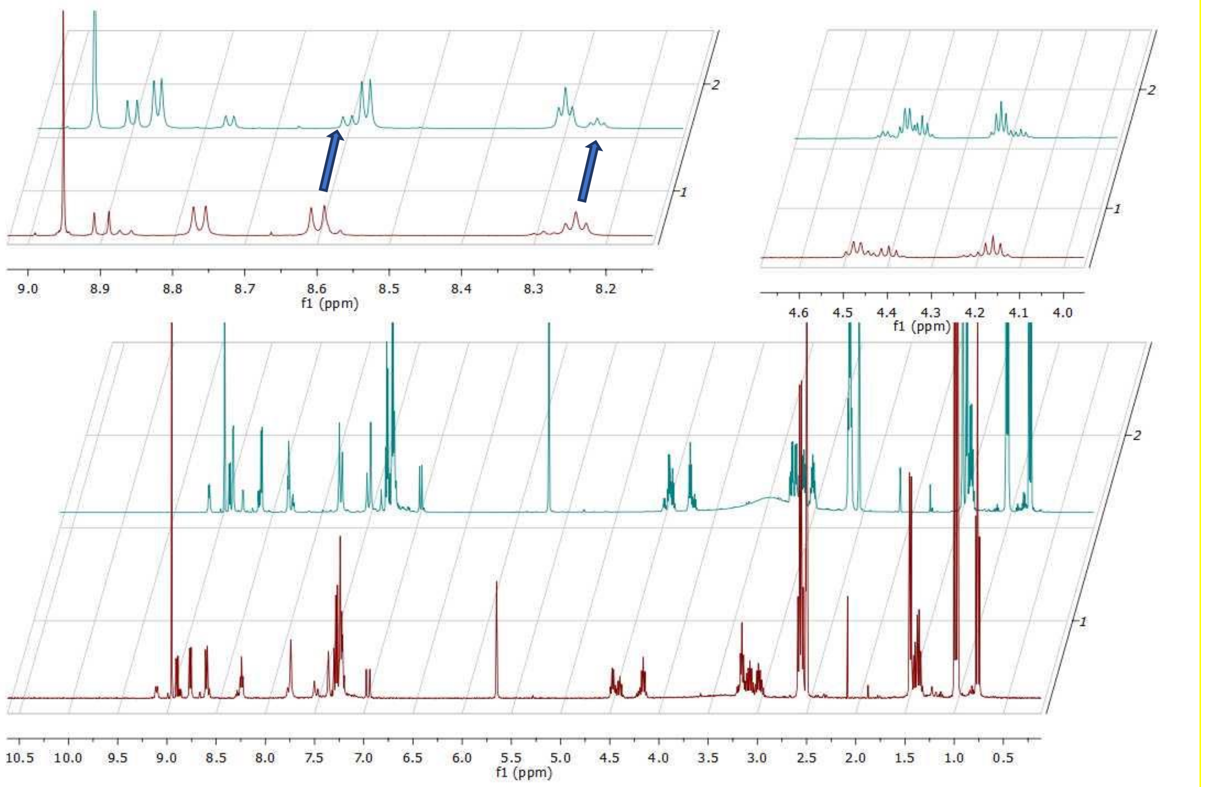

Spectrum of Marfey-derived **2r** from $Ti(OiPr)_4$ (Table 4), 95:5 er

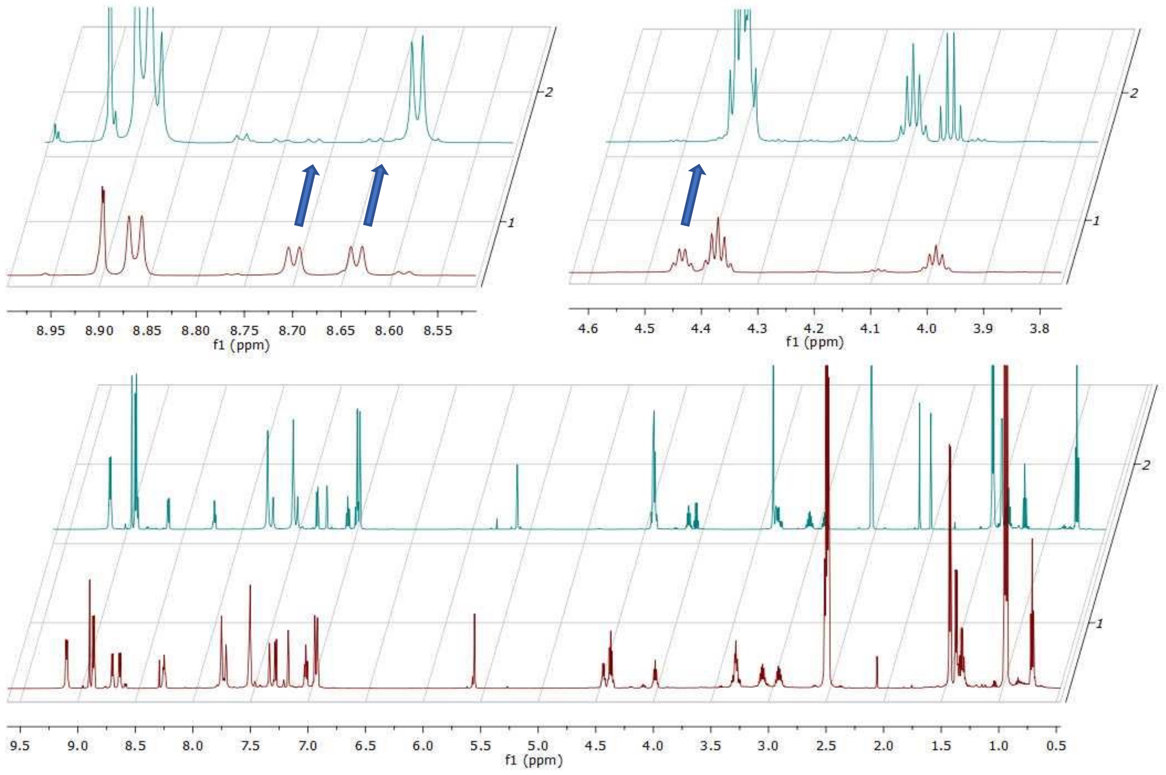

Spectrum of Marfey-derived **2t** from $Ti(OiPr)_4$ (Table 4), >95:5 er

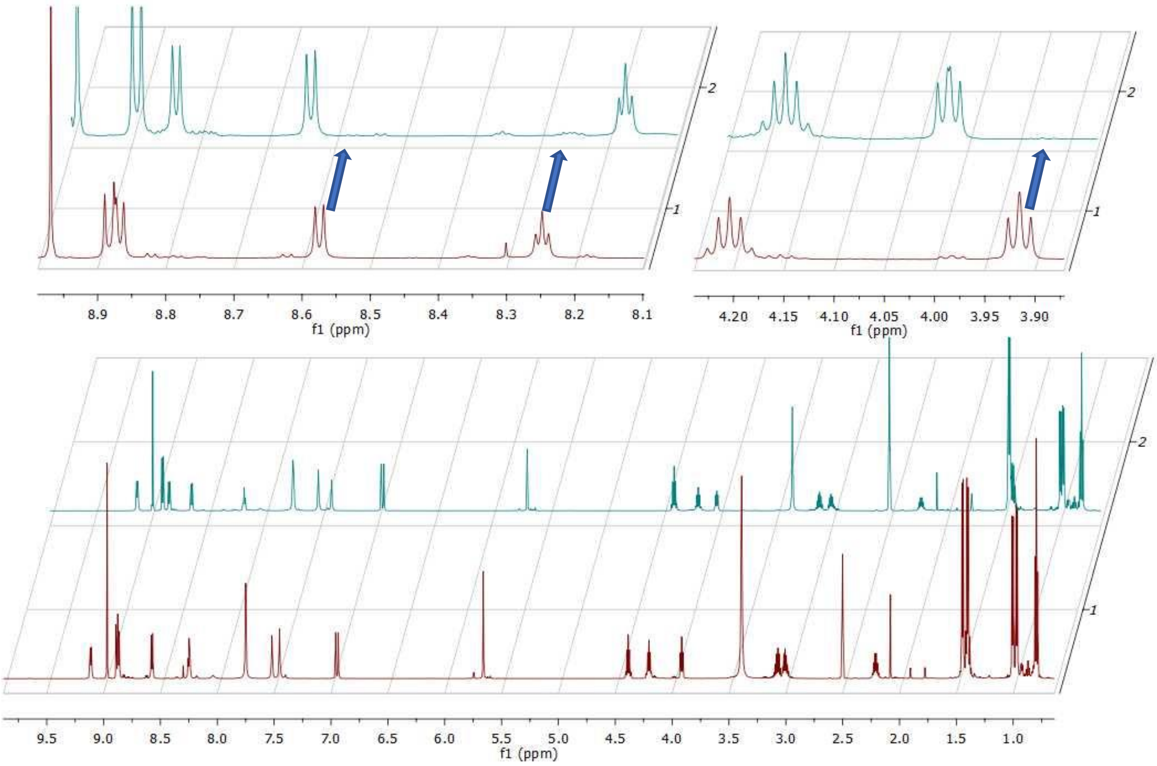

Spectrum of Marfey-derived **6c**, with 20 mol% $B(OCH_2CF_3)_3$, >95:5 er

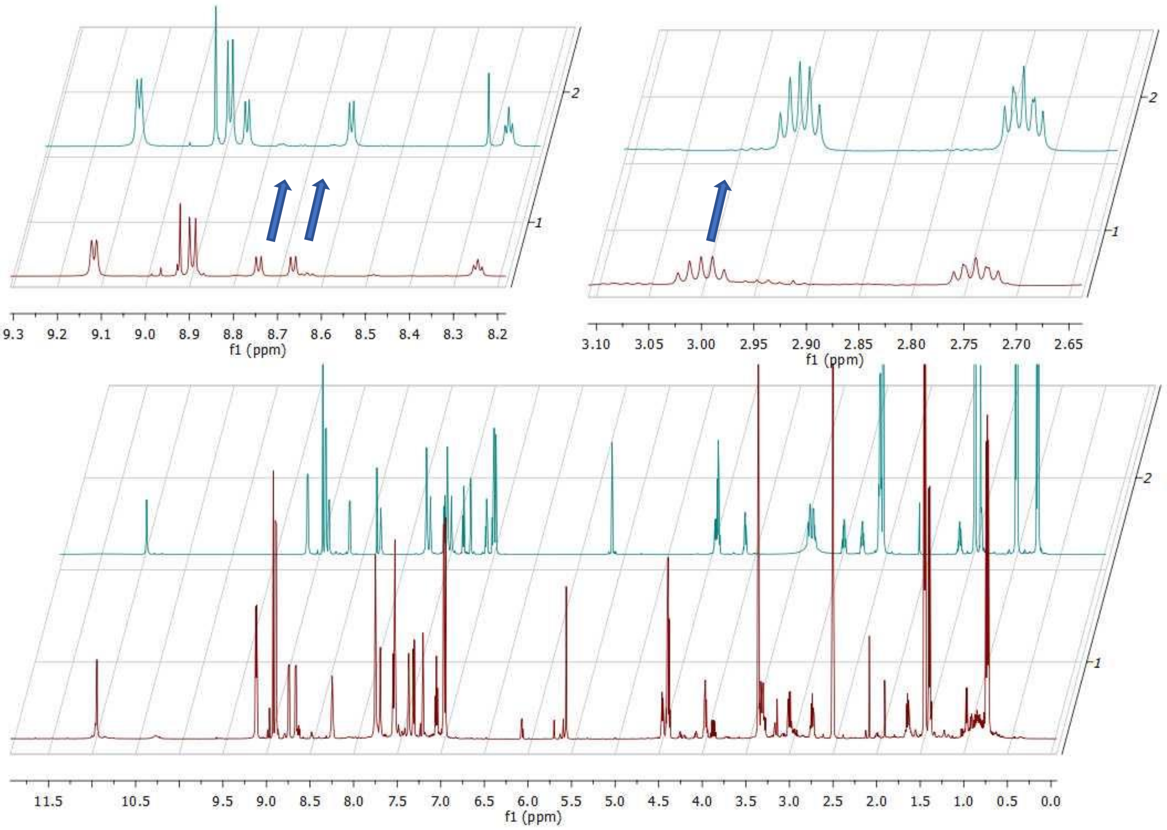

Spectrum of Marfey-derived **6g**, >95:5 er

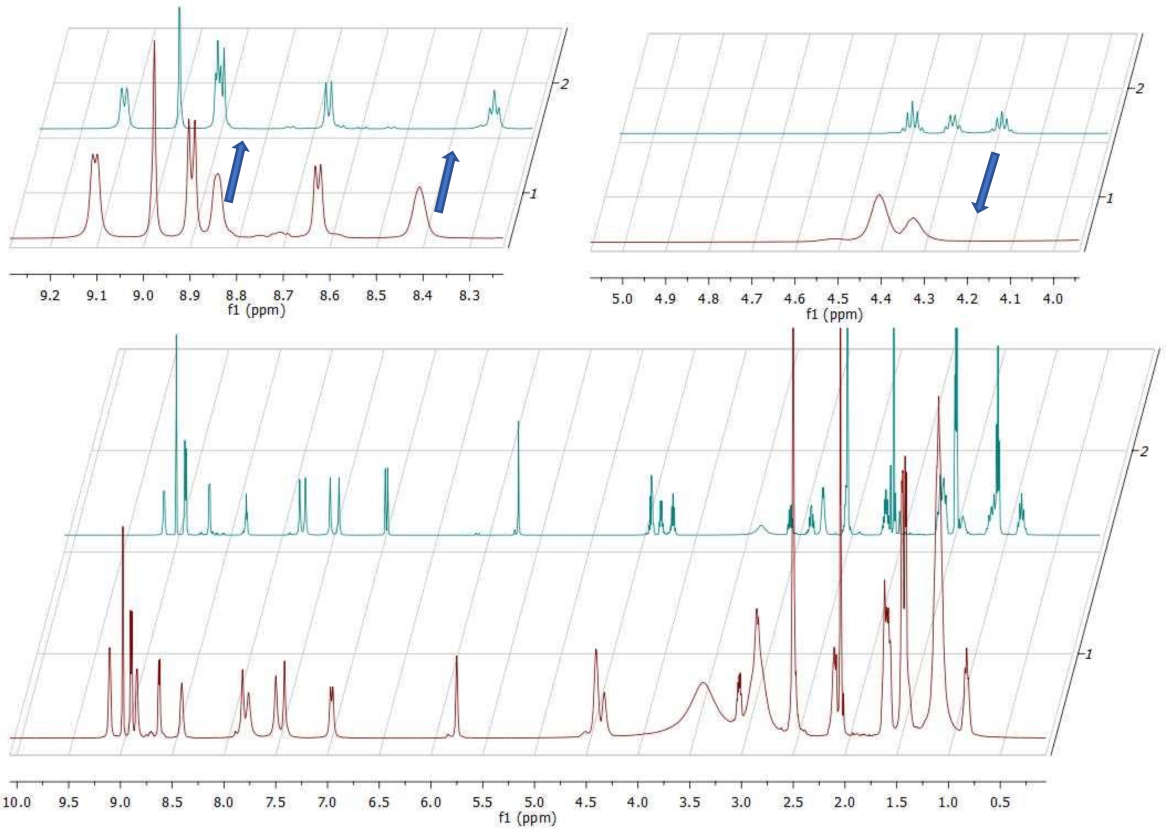

Spectrum of Marfey-derived **6h**, 60:40 er

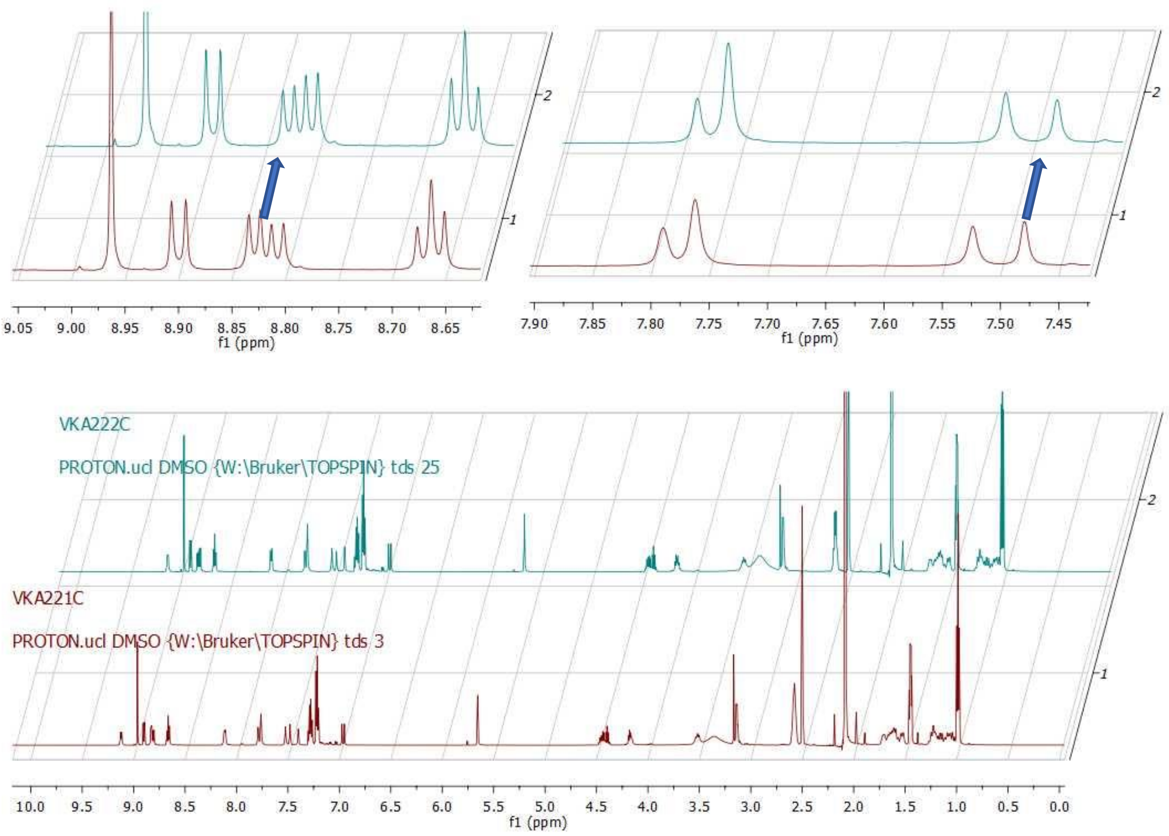

Chiral HPLC trace of **6i**, 90:10 er

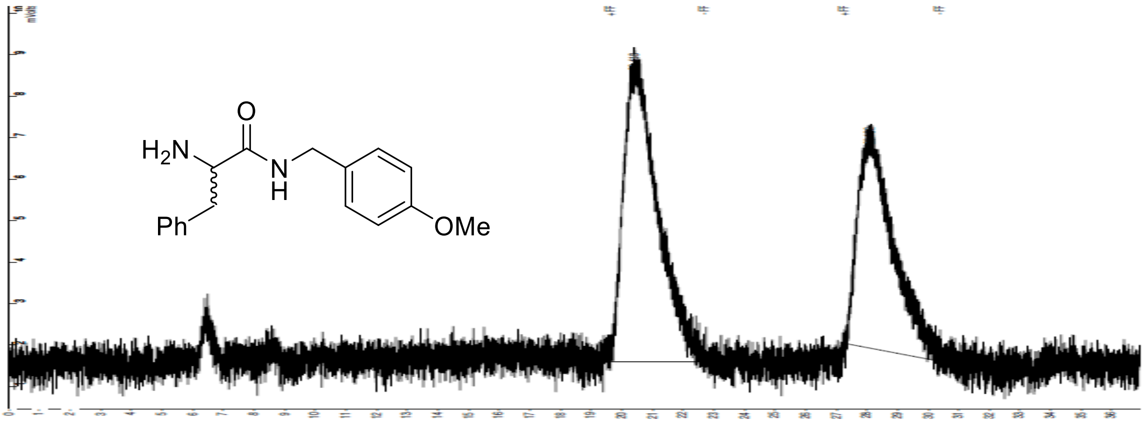

Peak No.	Peak Name	Result ()	Ret. Time (min)	Time Offset (min)	Area (counts)	Sep. Code	Width 1/2 (sec)	Status Codes
1		57.1505	20.418	0.000	559287	BB	63.1	
2		42.8495	28.132	0.000	419334	BB	63.4	
	Totals:	100.0000		0.000	978621			

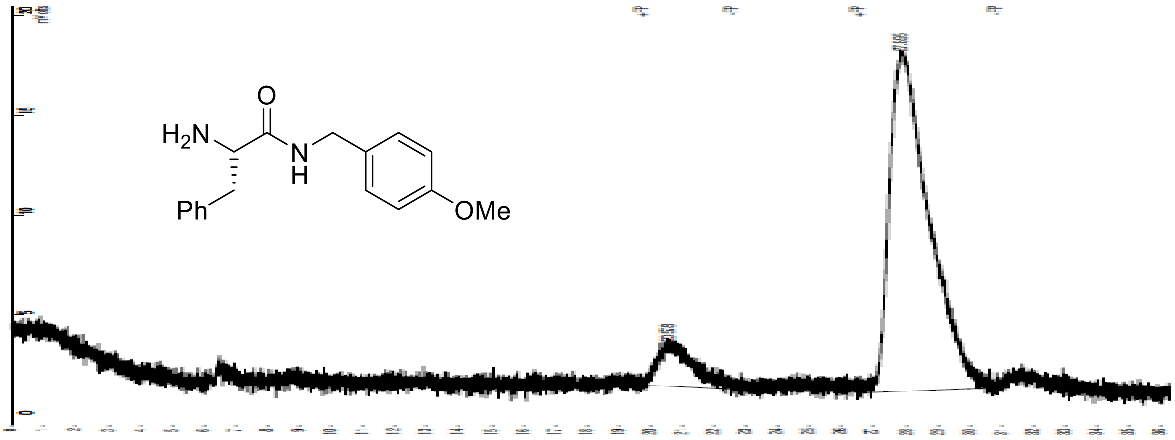

Peak No.	Peak Name	Result ()	Ret. Time (min)	Time Offset (min)	Area (counts)	Sep. Code	Width 1/2 (sec)	Status Codes
1		10.3881	20.578	0.000	170323	BB	32.8	
2		89.6119	27.895	0.000	1469275	BB	77.7	
	Totals:	100.0000		0.000	1639598			

Spectrum of Marfey-derived **6j**, from stoichiometric $B(OCH_2CF_3)_3$, 85:15 er

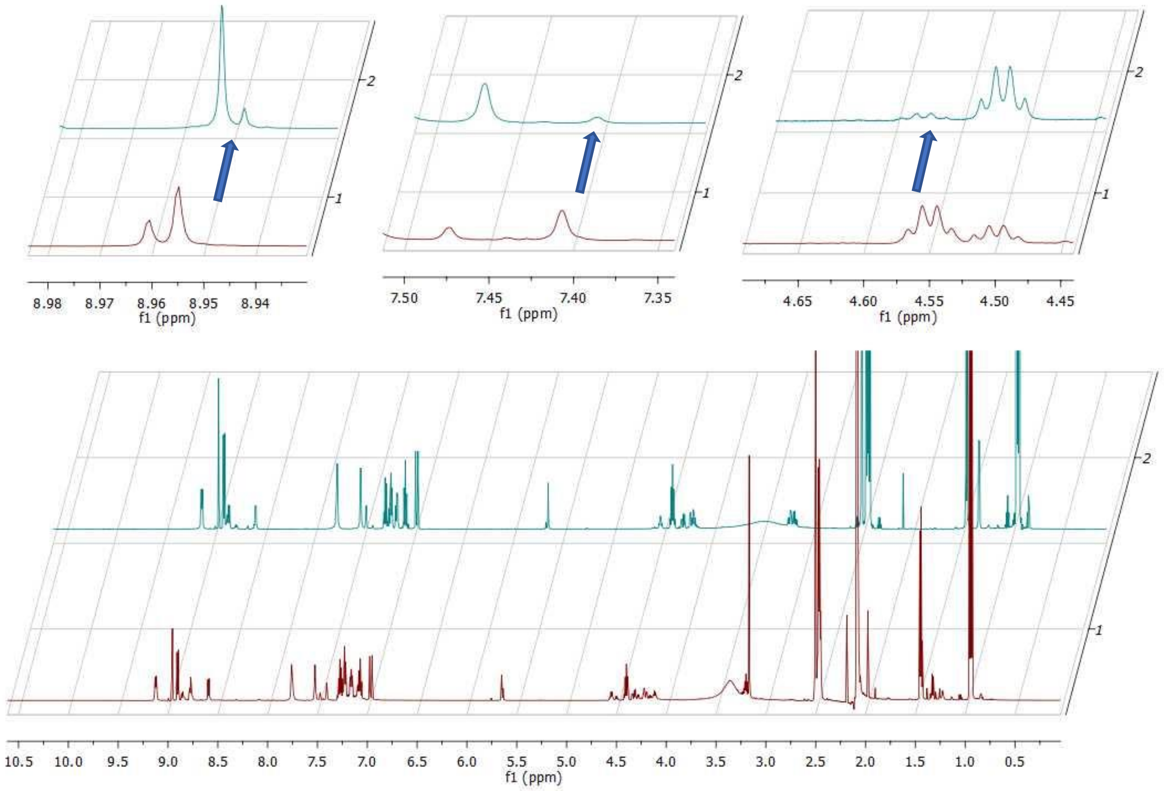

Spectrum of Marfey-derived **6j**, from catalytic $B(OCH_2CF_3)_3$, 89:11 er

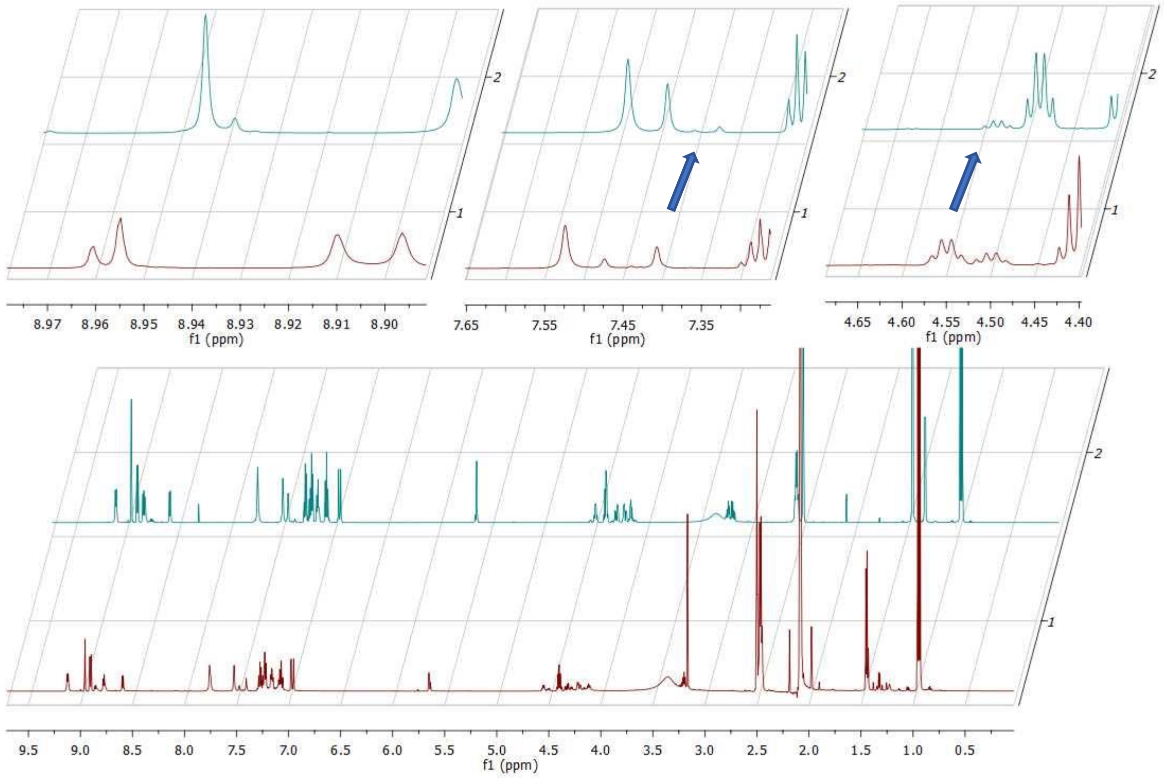

Spectrum of Marfey-derived **6l**, from catalytic $B(OCH_2CF_3)_3$, >95:5 er

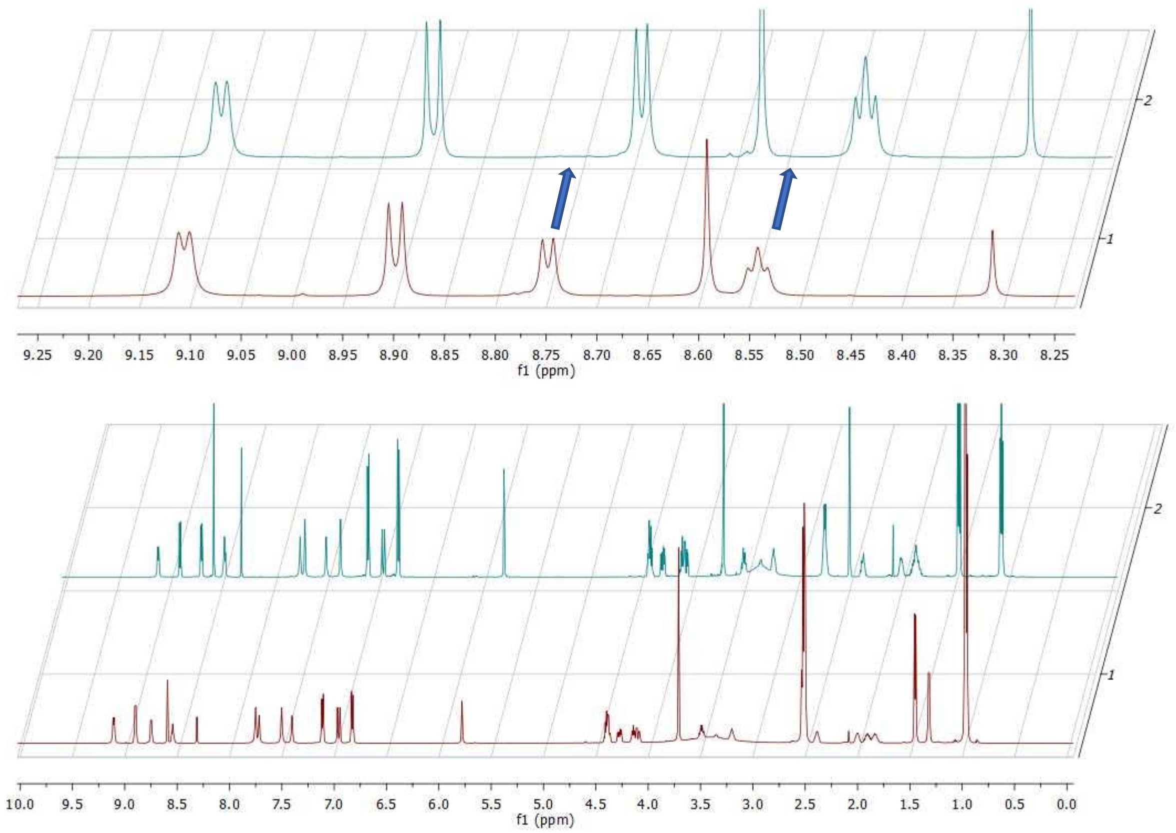

Spectrum of Marfey-derived **6m**, from catalytic $B(OCH_2CF_3)_3$, 88:12 er

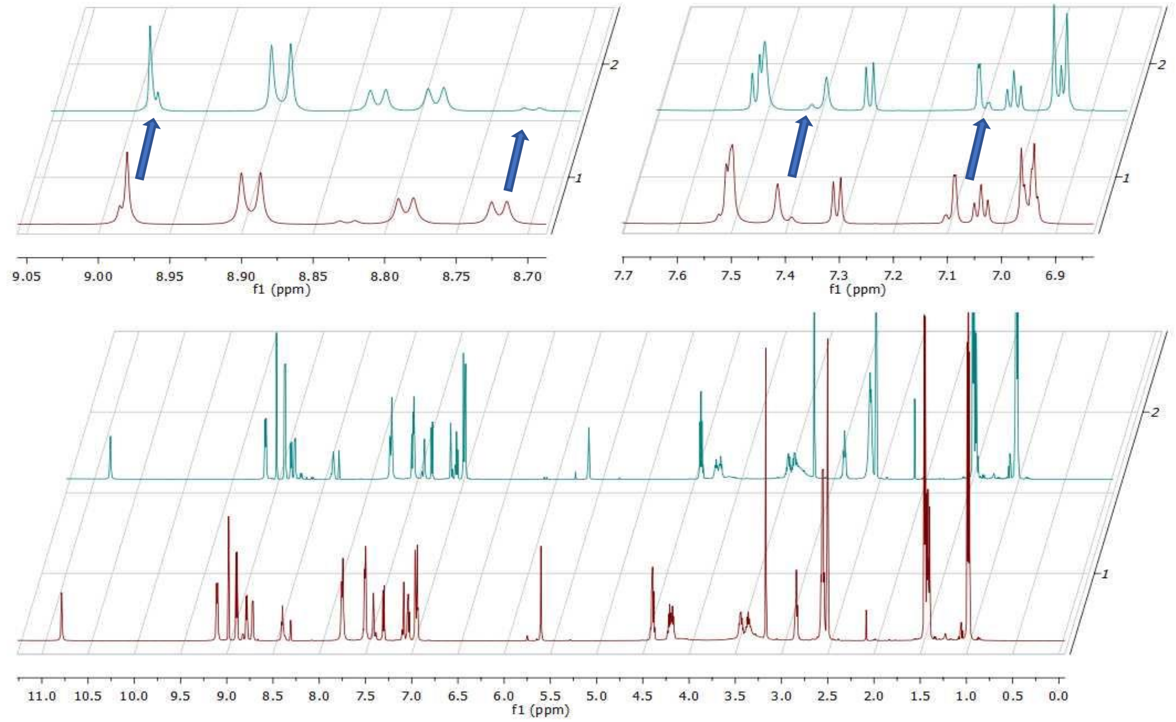

Spectrum of Marfey-derived **6n**, from catalytic $B(OCH_2CF_3)_3$, >95:5 er

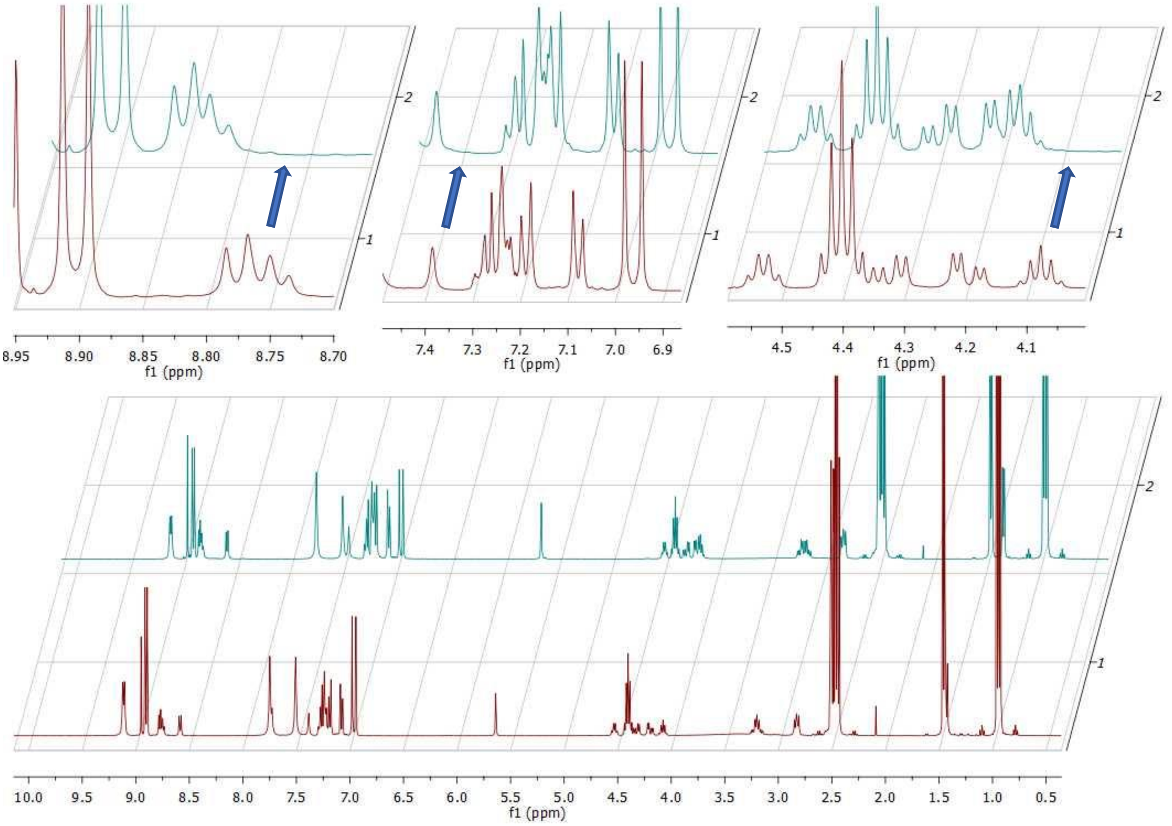

Spectrum of Marfey-derived **6o**, from stoichiometric $B(OCH_2CF_3)_3$, 60:40 er

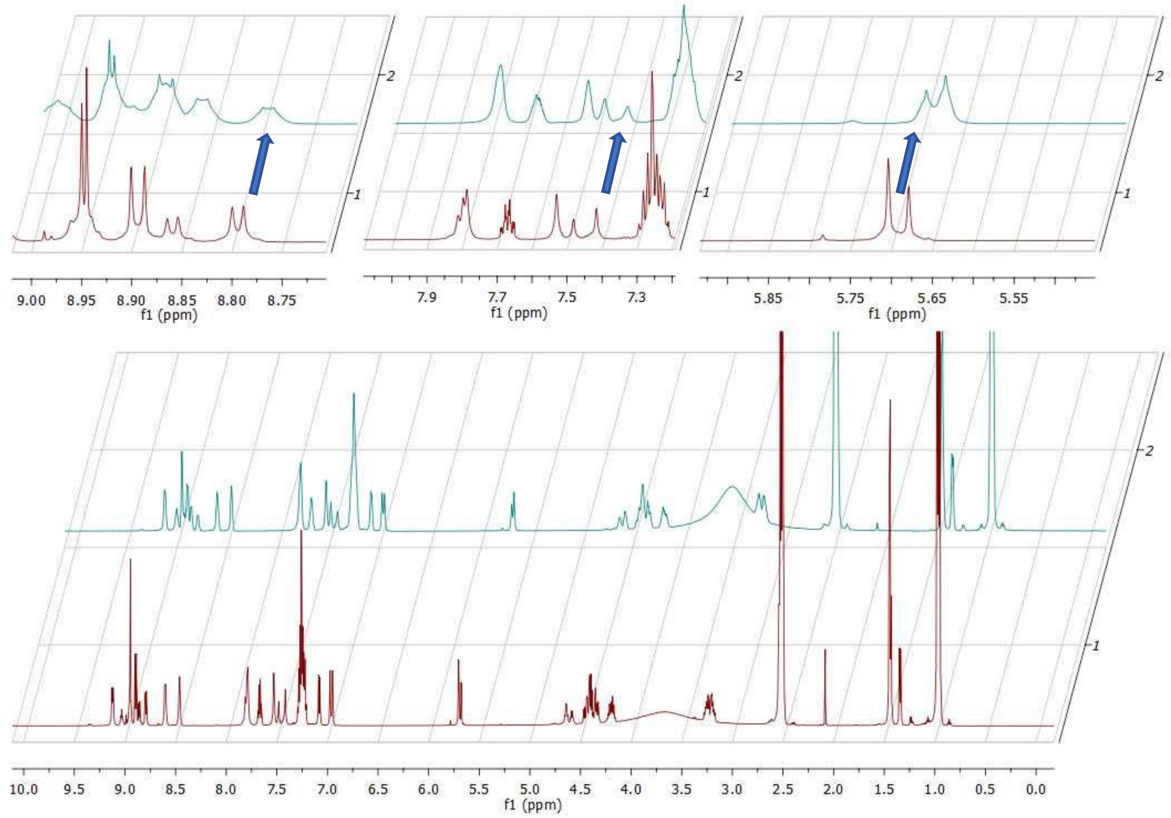

Spectrum of Marfey-derived **6o**, from catalytic $B(OCH_2CF_3)_3$, 92:8 er

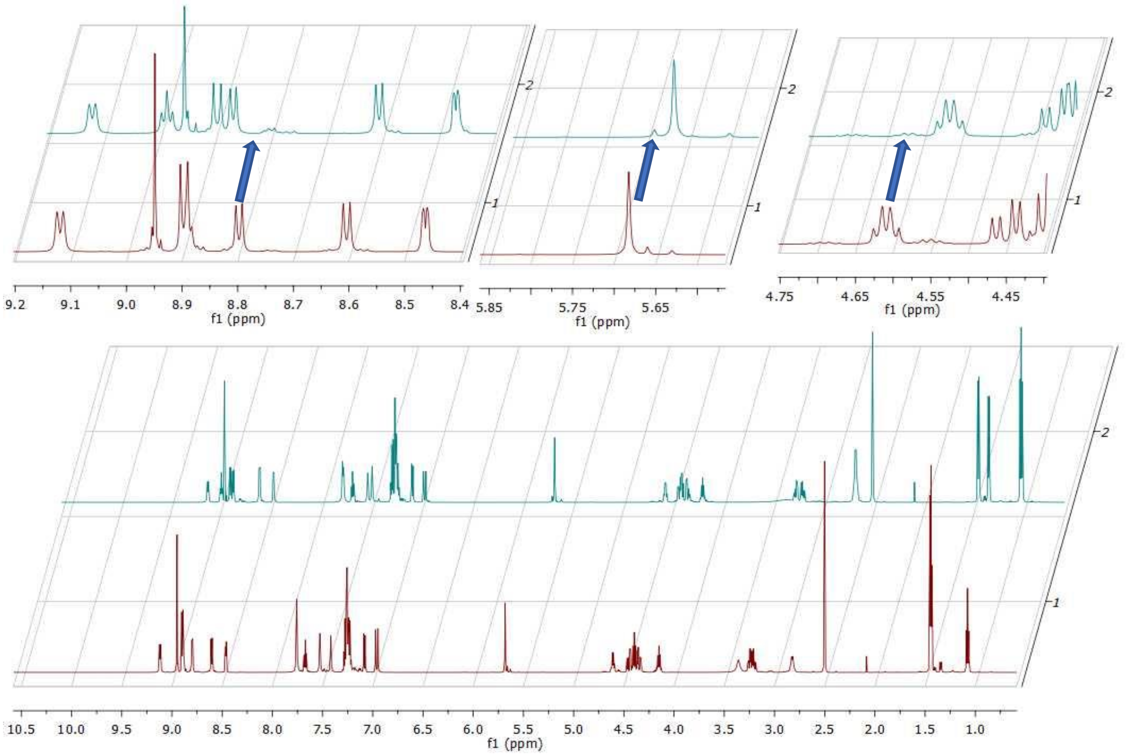

Spectrum of Marfey-derived **6p**, 50:50 er

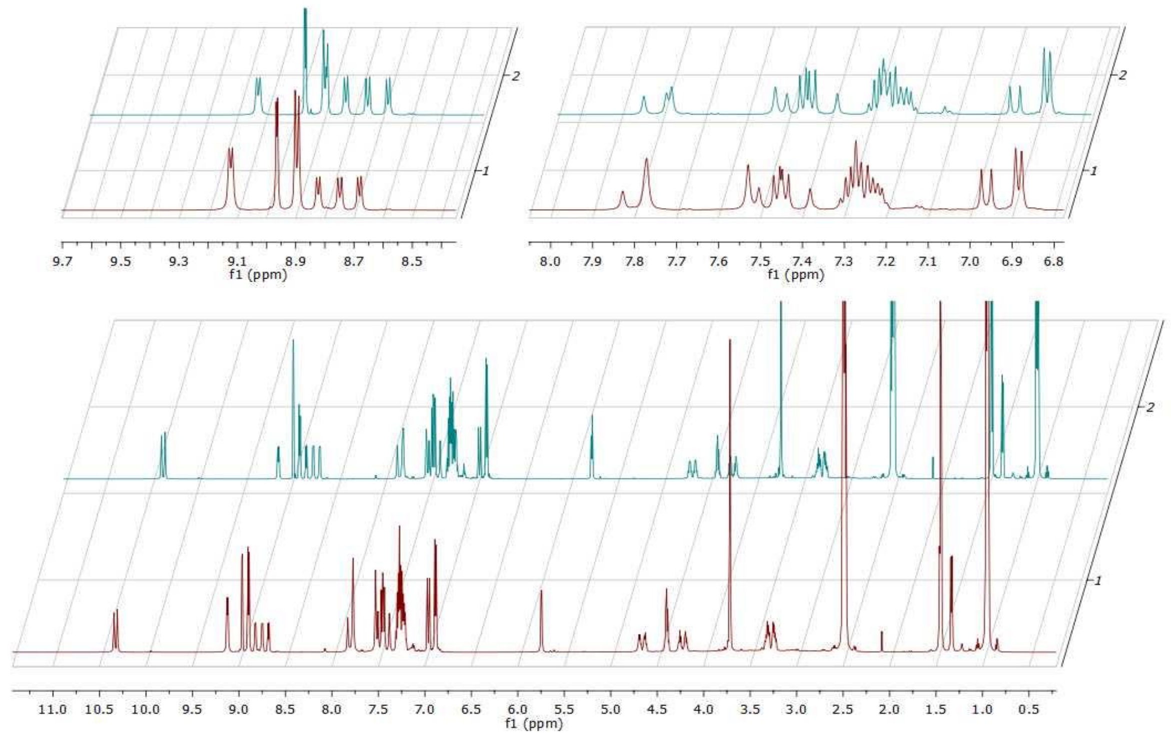

Spectrum of Marfey-derived **6s**, 60:40 er

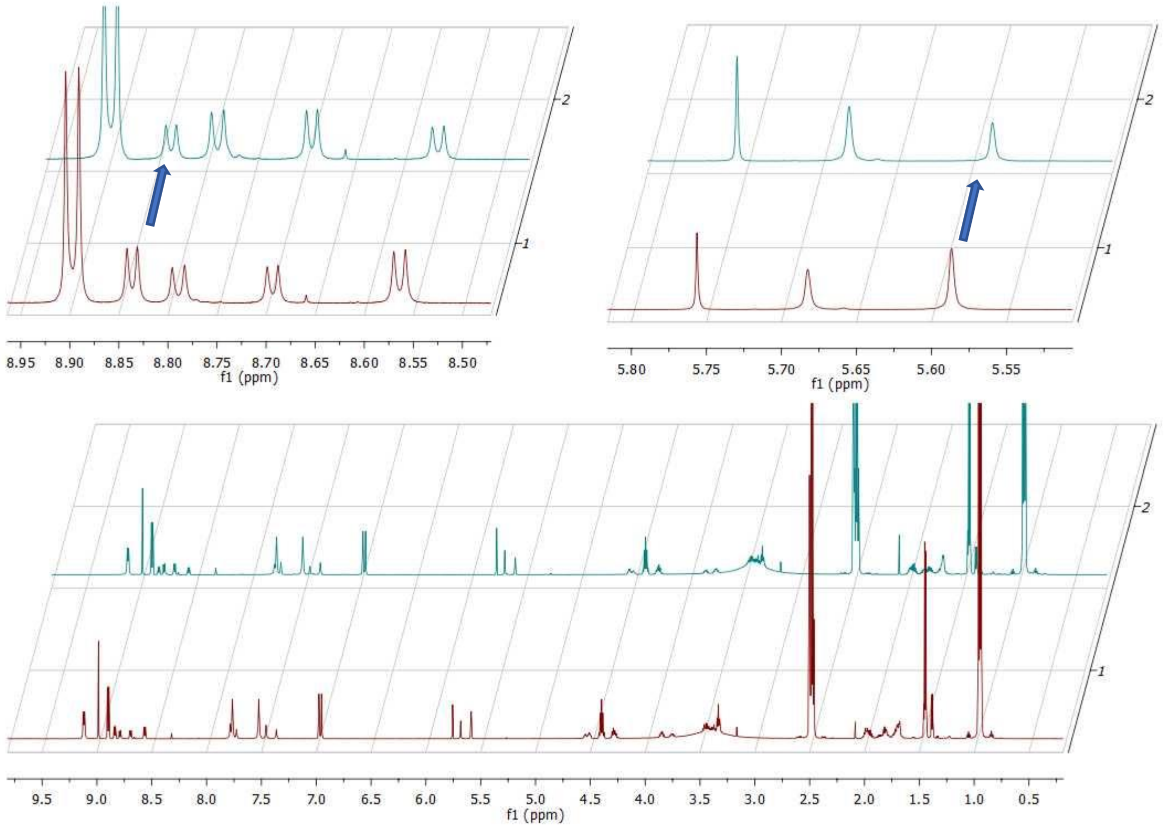

Spectrum of Marfey-derived **6s**, from tris(pyrrolidino)borane 93:7 er

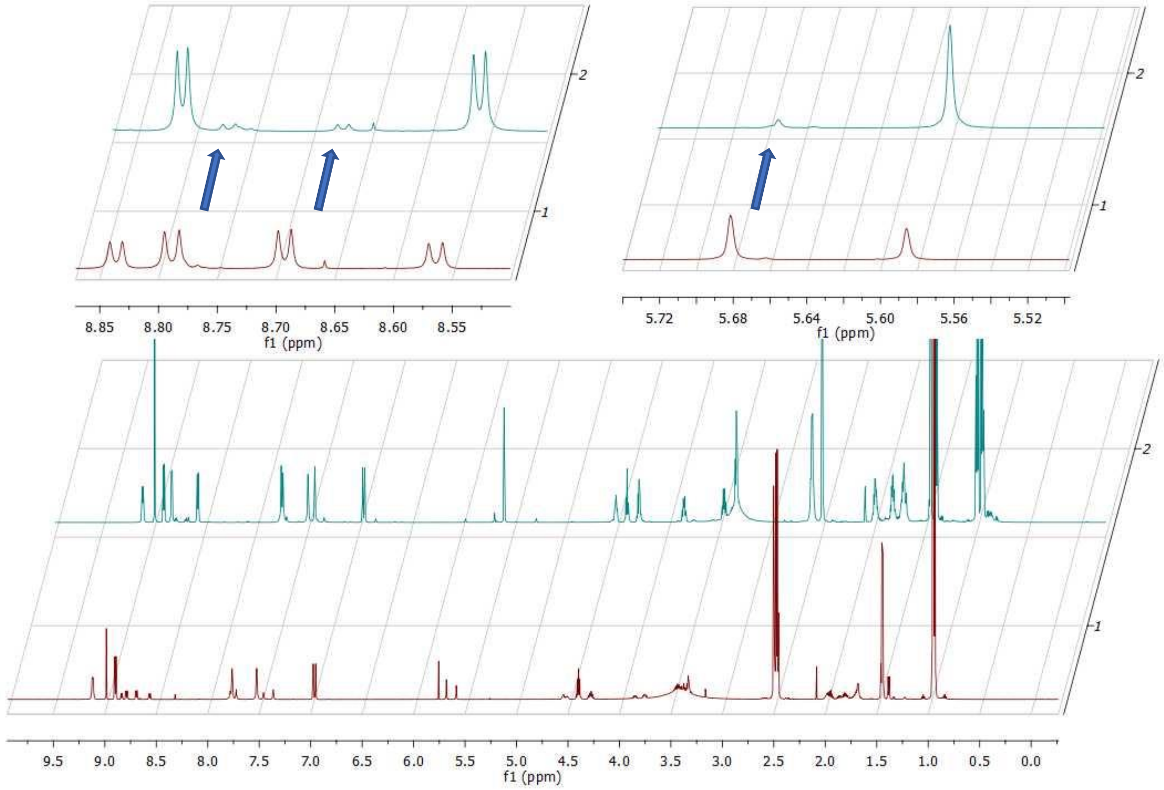

Spectrum of Marfey-derived **6u**, 53:47 er

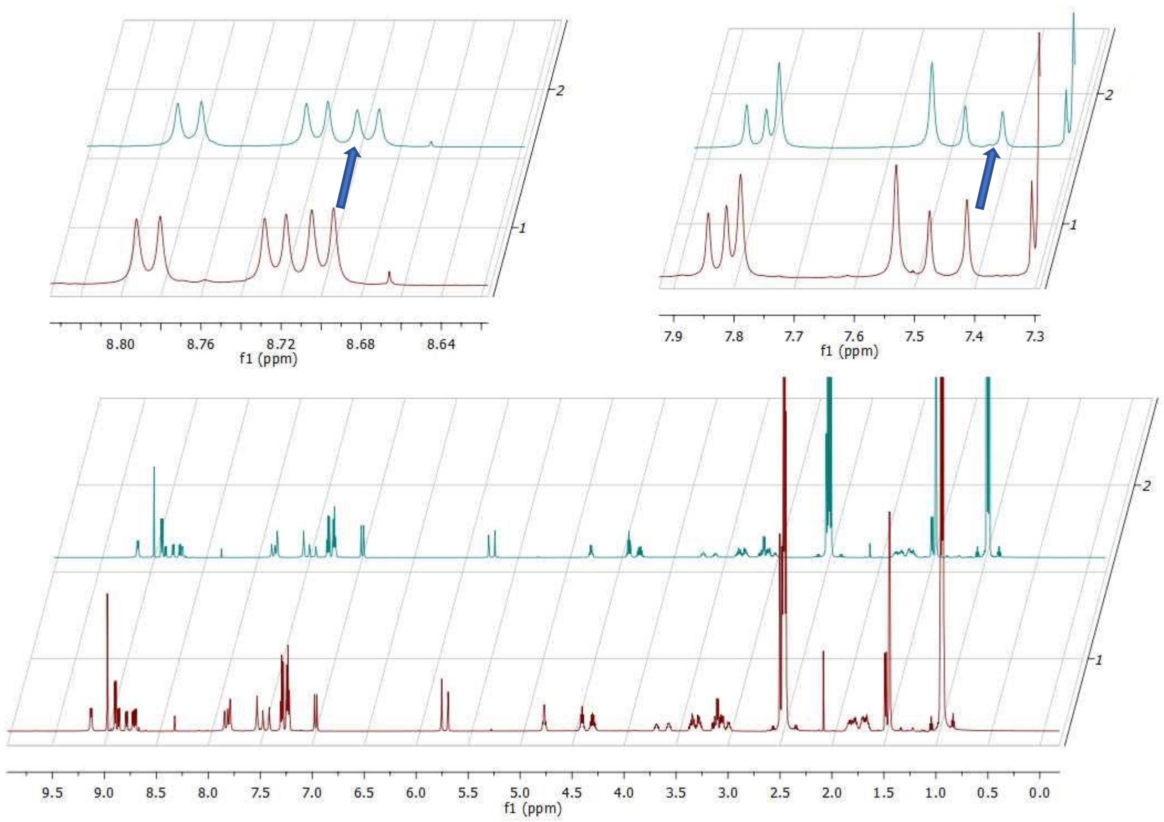

Spectrum of Marfey-derived **6u**, from tris(pyrrolidino) borane 94:6 er

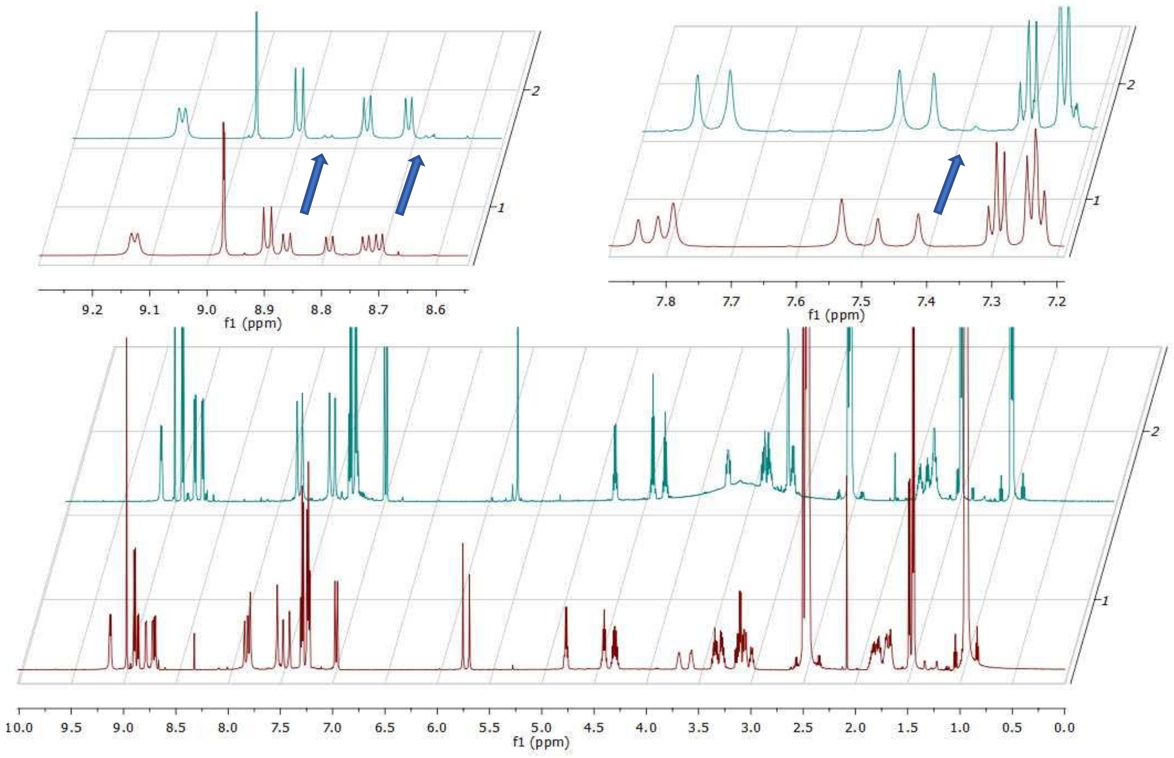

Spectrum of Marfey-derived **6y**, 2:1 er

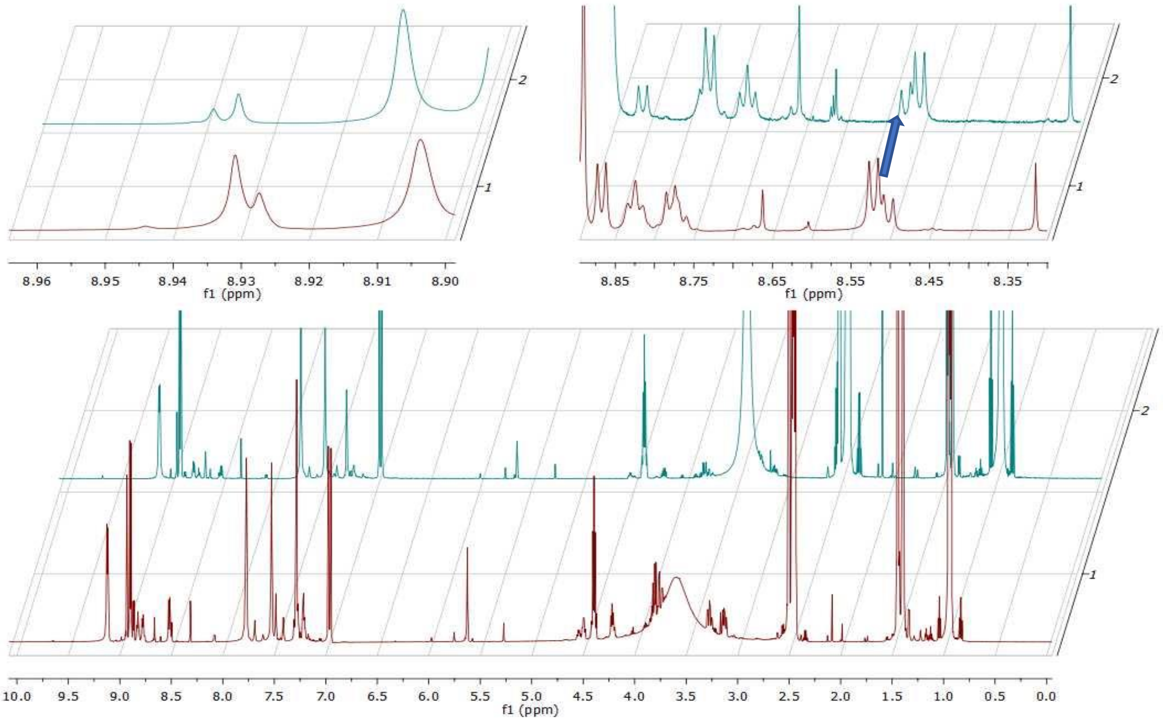

Spectrum of Marfey-derived **6ee**, with 1 eq $B(NMe_2)_3$, >95:5 er

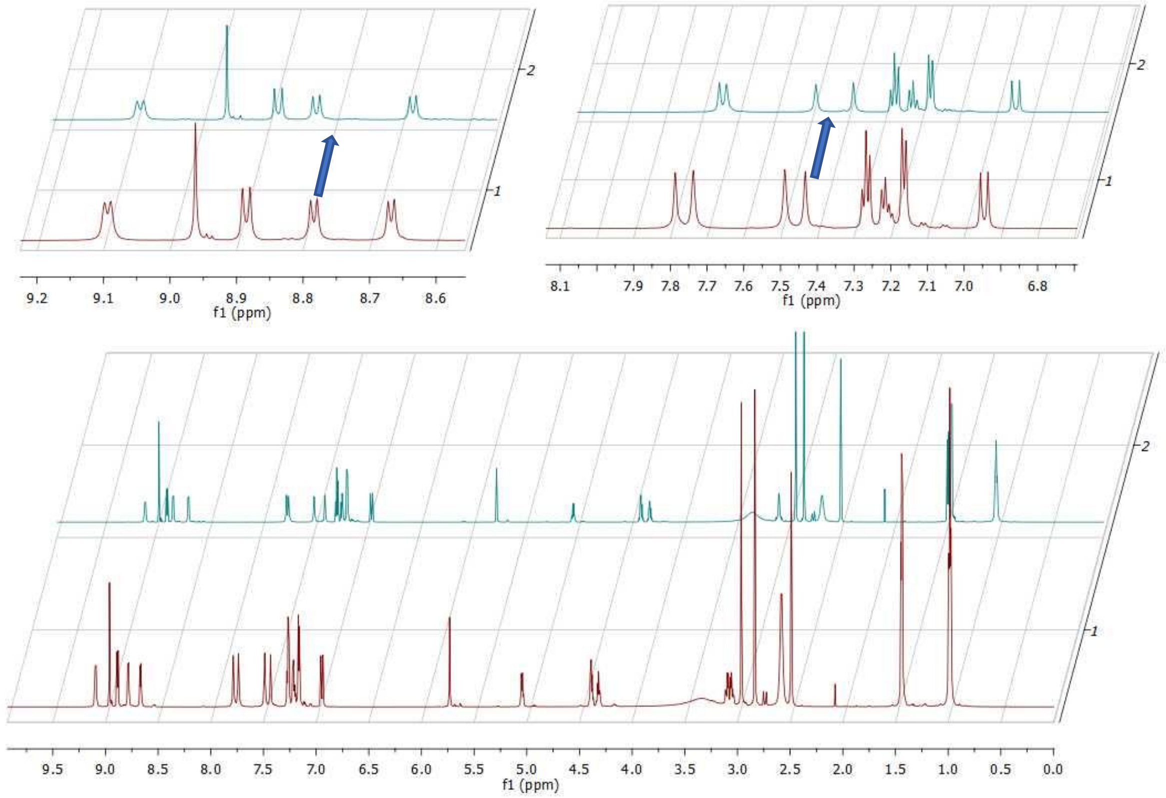

Spectrum of Marfey-derived **6ee**, with 1.5 eq $B(NMe_2)_3$, 92:8 er

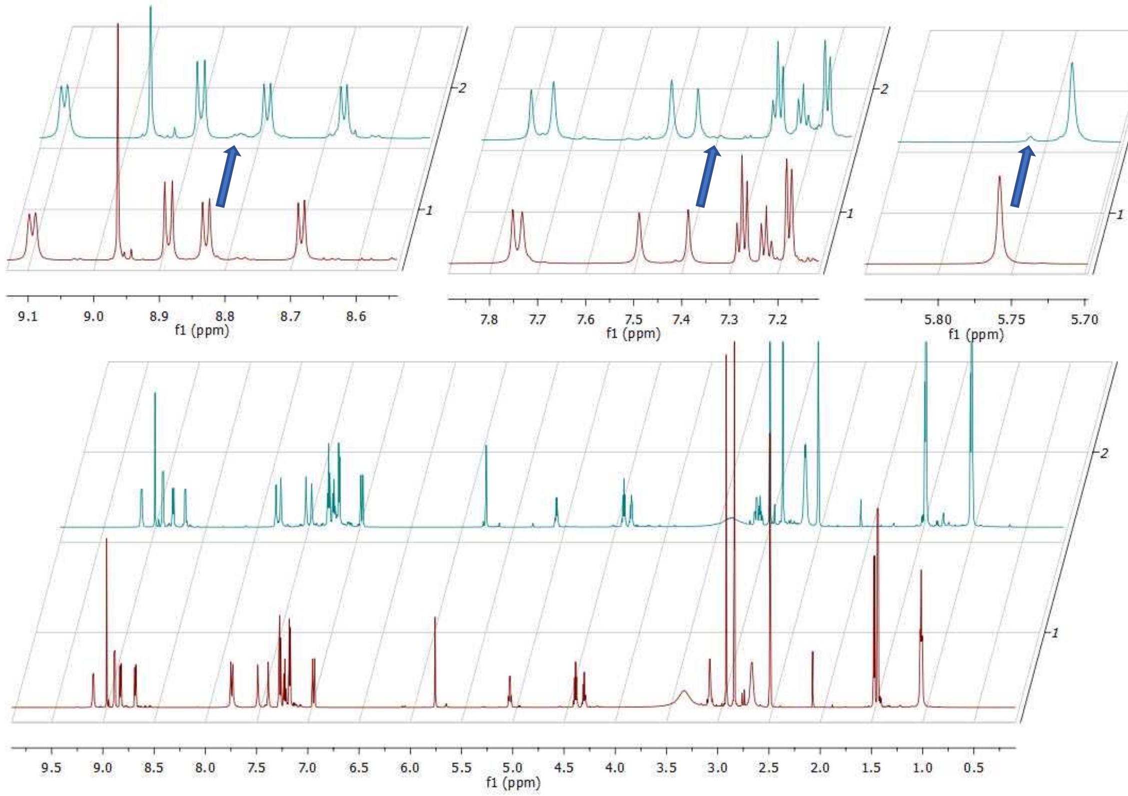

Spectrum of Marfey-derived **6ff**, with 1.5 eq $B(NMe_2)_3$, >95:5 er

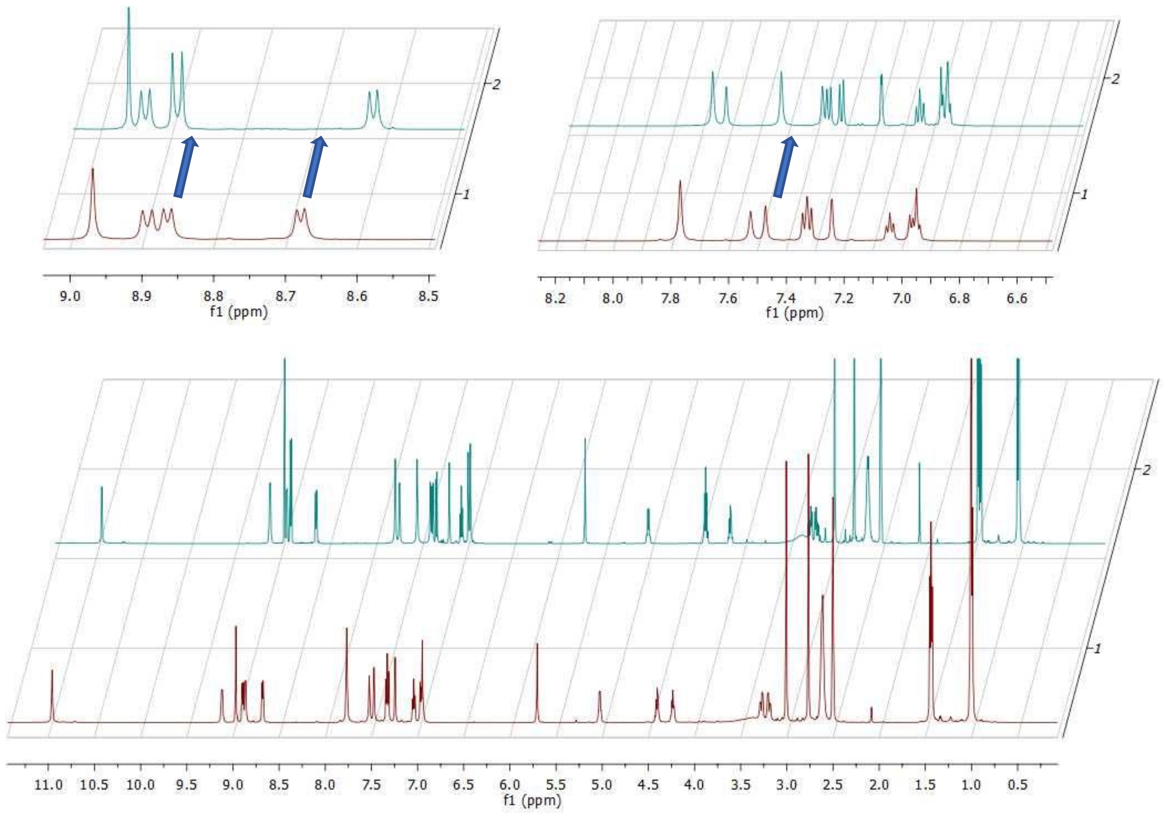

Spectrum of Marfey-derived **6gg**, with 1.5 eq $B(NMe_2)_3$, >95:5 er

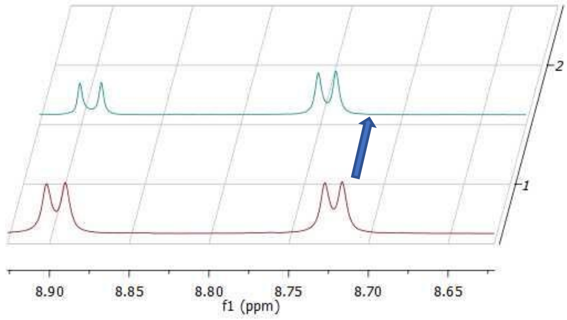

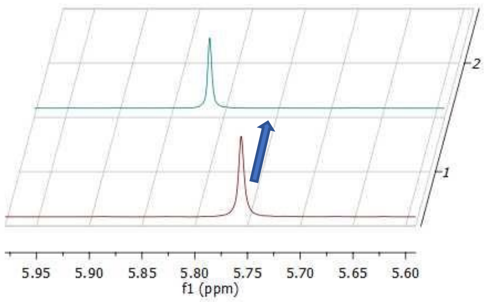

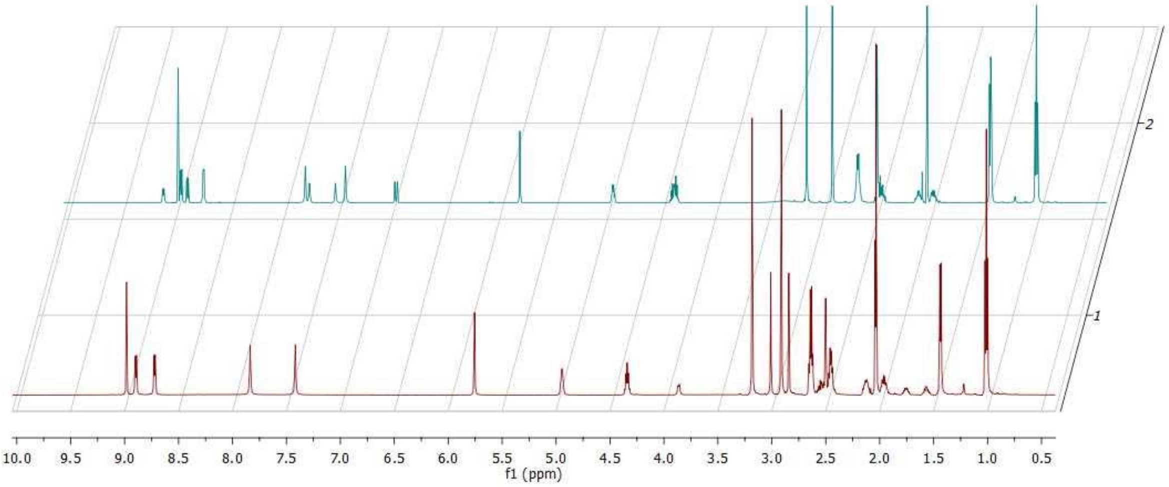

(±)-2-(2-(4-Bromophenyl)acetamido)-3-phenyl-N-propylpropanamide rac-10a

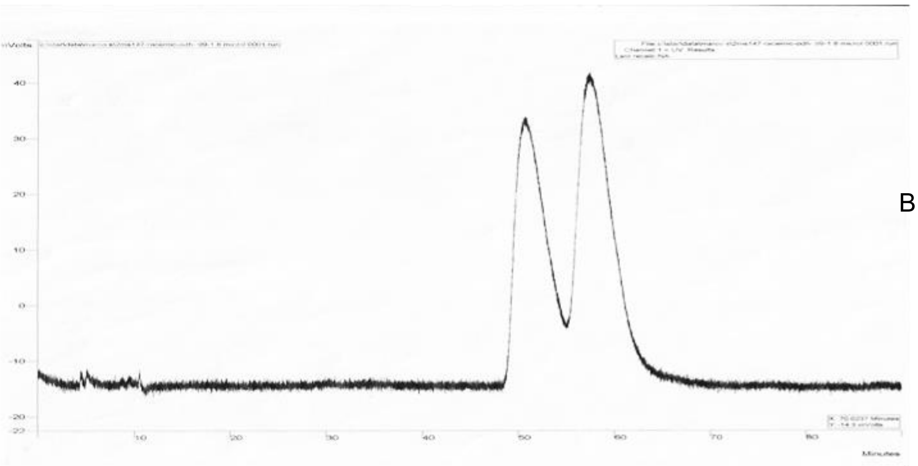

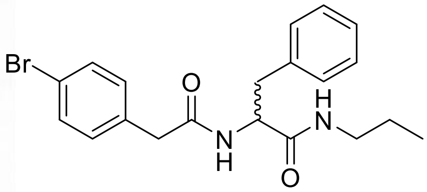

(S)-2-(2-(4-Bromophenyl)acetamido)-3-phenyl-N-propylpropanamide (S)-10a

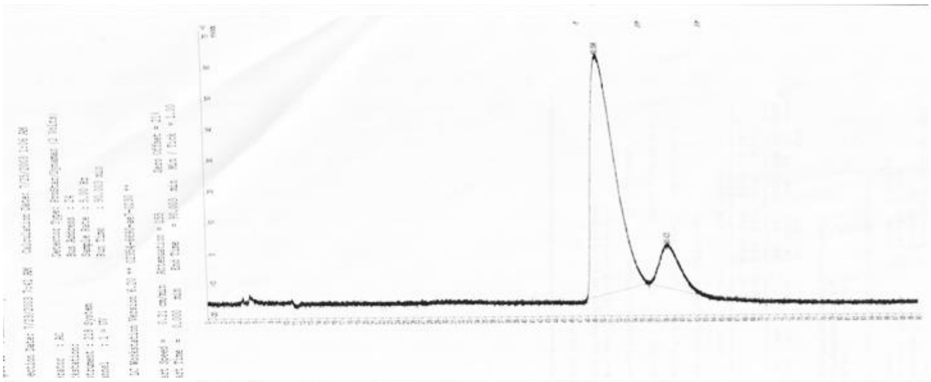

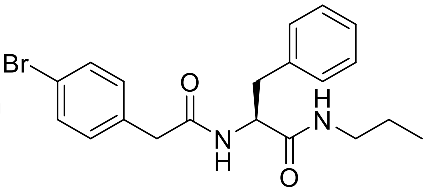

Run Mode : Analysis
Peak Measurement: Peak Area
Calculation Type: Percent (Normalized Results)

Peak No.	Peak Name	Result ()	Ret. Time (min)	Time Offset (min)	Area (counts)	Sep. Code	Width 1/2 (sec)	Status Codes
1		86.2912	50.098	0.000	16028991	BB	199.1	
2		13.7088	58.425	0.000	2546479	BB	135.9	
	Totals:	100.0000		0.000	18575470			

tert-butyl (±)-methyl(2-oxo-2-((1-oxo-3-phenyl-1-(propylamino)propan-2-yl)amino)ethyl)carbamate rac-10b

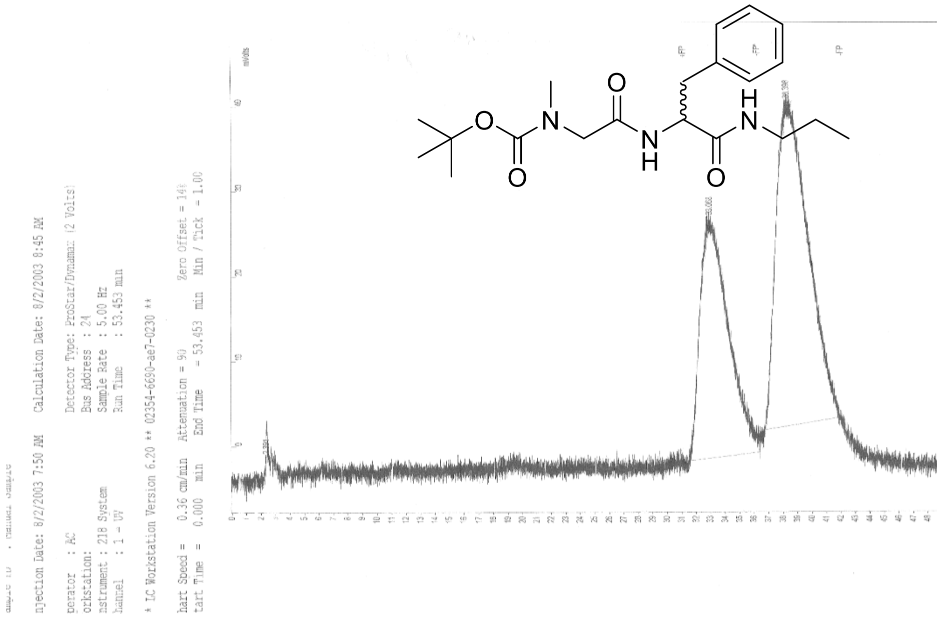

tert-butyl (*R*)-methyl(2-oxo-2-((1-oxo-3-phenyl-1-(propylamino)propan-2-yl)amino)ethyl)carbamate (R)-10b

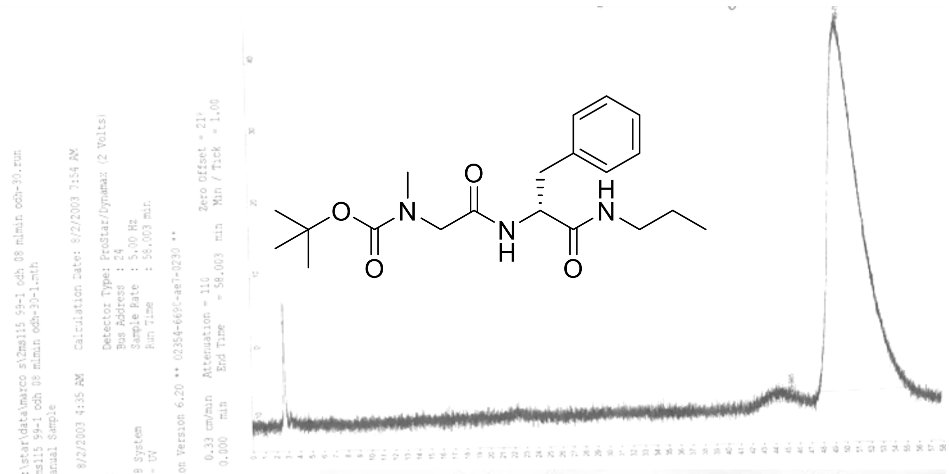

Result ()	Ret. Time (min)	Time Offset (min)	Area (counts)	Sep. Code	Width 1/2 (sec)	Status Codes
1.9279	45.385	0.000	183675	BB	0.2	
98.0721	49.792	0.000	9343647	BB	157.6	
100.0000		0.000	9527322			

Rac-*N*-Benzyl-3-(1*H*-indol-3-yl)-2-(2-phenylacetamido)propenamide (rac-11c)

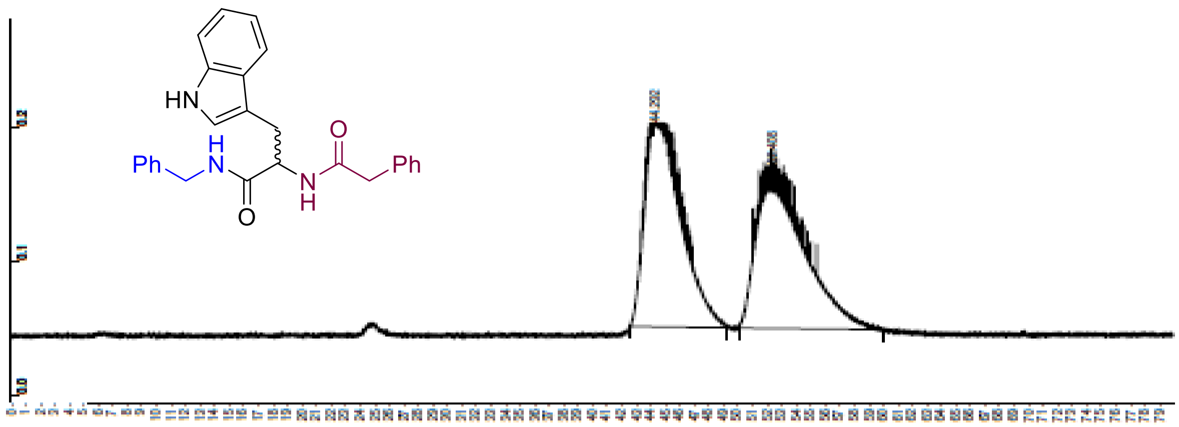

(S)-*N*-Benzyl-3-(1*H*-indol-3-yl)-2-(2-phenylacetamido)propenamide (S)-11c

Peak No.	Peak Name	Result ()	Ret. Time (min)	Time Offset (min)	Area (counts)	Sep. Code	Width 1/2 (sec)	Status Codes
1		2.1111	44.325	0.000	998448	BB	105.8	
2		97.8889	50.898	0.000	46297352	BB	211.2	
	Totals:	100.0000		0.000	47295800			

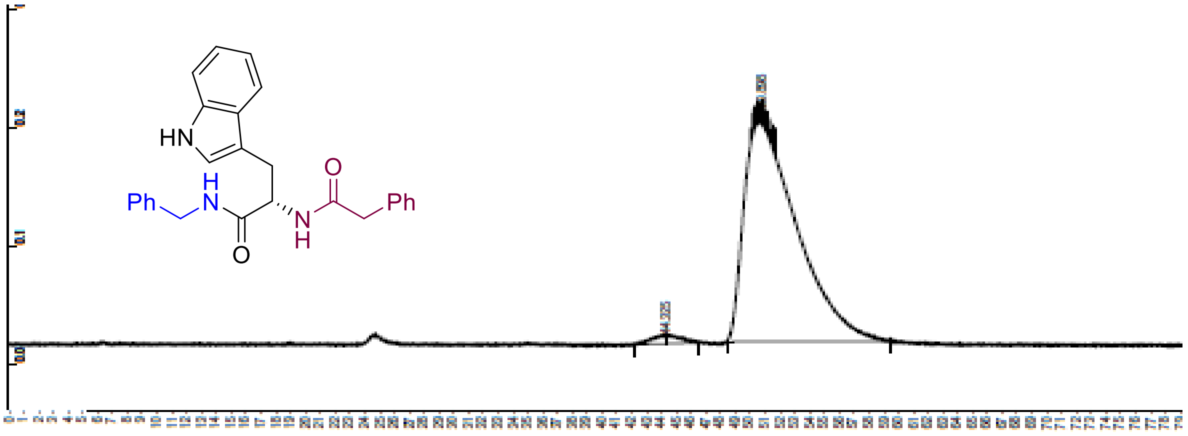

[1] Y. J. Pu, R. K. Vaid, S. K. Boini, R. W. Towsley, C. W. Doecke, D. Mitchell. *Org. Proc. Res. Dev.,* 2009, **13**, 310–314

[2] G.-J. Ho, K. M. Emerson, D. J. Mathre, R. F. Shuman, E. J. J. Grabowski. *J. Org. Chem.,* 1995, **60**, 3569-3570

[3] A. Leggio, E. L. Belsito, G. De Luca, M. L. Di Gioia, V. Leotta, E. Romio, C. Siciliano, A. Liguori. *RSC Adv.*, 2016, **6**, 34468-34475

[4] R. K. Sharma, R. Jain. *Synlett*, **2007**, 603-606

[5] A. M. King, C. Salomé, J. Dinsmore, E. Salomé-Grosjean, M. De Ryck, R. Kaminski, A. Valade, H. Kohn. *J. Med. Chem.*, 2011, **54**, 4815–4830

[6] B. Ramalingam, M. Neuburger, A. Pfaltz. *Synthesis*, **2007**, 572-582

[7] R. M. Lanigan, V. Karaluka, M. T. Sabatini, P. Starkov, M. Badland, L. T. Boulton, T. D. Sheppard. *Chem. Commun*., 2016, **52**, 8846-8849

[8] M. Amedjkouh, P. Ahlberg. *Tetrahedron Asymmetry*, 2002, **13**, 2229 - 2234

[9] V. Karaluka, R. M. Lanigan, P. M. Murray, M Badland, T. D. Sheppard. *Org. Biomol. Chem*., 2015, **13**, 10888-10894

[10] S. Sun, Md. A. Fazal, B. C. Roy, S. Mallik. *Org. Lett*., 2000, **2**, 911–914

[11] E. Tarkin-Tas, C. A. Lange, L. J. Mathias. *J. Polym. Sci., Part A: Polym. Chem.,* 2011, **49**, 2451 - 2460

[12] S. B. Wadavrao, A. Narikimalli, A. V. Narsaiah. *Synthesis*, 2013, **45**, 3383 - 3386

[13] T. Mimoto, R. Kato, H. Takaku, S. Nojima, K. Terashima, S. Misawa, T. Fukazawa, T. Ueno, H. Sato, M. Shintani, Y. Kiso, H. Hayashi. *J. Med. Chem.*, 1999, **42**, 1789 -1802

[14] A. J. A.Watson, R. J. Wakeham, A C. Maxwell, J. M. J. Williams. *Tetrahedron*, 2014, **70**, 3683-3690.

[15] J. Escorihuela, M. I. Burguete, G. Ujaque, A. Lledós, S. V. Luis. *Org. Biomol. Chem.*, 2016, **14**, 11125-11136

[16] M. T. Sabatini, L. T. Boulton, T. D. Sheppard. *Sci. Adv.,* 2017, **9**, 1058-1072

[17] E. J. Corey, R. L. Dawson. *J. Am. Chem. Soc.,* 1962, **84**, 4899-4904

[18] H. Zhao, Z. Huang, W. Chen. *J. Org. Chem.*, 2014, **79**, 11290 - 11294

[19] Y. Knobler, S. Bittner, M. Frankel. *J. Chem. Soc.,* 1964, **35**, 3941-3951

[20] W. Zhenyu, M. Liguang, H. Qun; Z. Weicheng. Patent number CN103319367 A (2012)

[21] J. Christoffers, A. Mann. *Chem. Eur. J.*, 2001, **7**, 1014-1027

[22] P. S. Fier, A. M. Whittaker. *Org. Lett*., 2017, **19**, 1454–1457
